# Supplementary material for: The mechanosensitive TRPV2 calcium channel promotes human melanoma invasiveness and metastatic potential
Source: EMBO Rep. 2023 Feb 6;24(4):e55069. doi: 10.15252/embr.202255069 (PMC10074106; doi:10.15252/embr.202255069)
Supplement: Supplementary file 1 — Appendix [file EMBR-24-e55069-s010.pdf]

Appendix for "The mechanosensitive TRPV2 calcium channel promotes human melanoma invasiveness and metastatic potential"by Kenji F. Shoji *et al.*

Table of content

**Appendix Figure S1 (related to Figure 1).**	**p.1**
**Appendix Figure S2 (related to Figure 2).**	**p.3**
**Appendix Figure S3 (related to Figure 3).**	**p.4**
**Appendix Figure S4.**	**p.5**
**Appendix Figure S5 (related to Figure 4).**	**p.6**
**Appendix Figure S6 (related to Figure 5).**	**p.7**
**Appendix Figure S7 (related to Figure 6).**	**p.9**
**Appendix Figure S8 (related to Figure 6).**	**p.10**
**Appendix Figure S9 (related to Figure 6).**	**p.12**
**Appendix Figure S10 (related to Figure 7).**	**p.13**
**Appendix Figure S11 (related to Figure 7).**	**p.14**
**Appendix Table S1.**	**p.15**
**Appendix Table S2.**	**p.16**
**Appendix Table S3.**	**p.16**

Appendix Figure S1 (related to Figure 1). TRPV2 is predominantly expressed in melanoma tumors as compared to nevi tissues or to cancers from other origin

(A) TRPV2 mRNA expression analysis within the Broad-Novartis Cancer Cell Line Encyclopedia (CCLE) project RNAseq data. The box plot is sorted and colored by the average distribution of TRPV2 expression in a lineage (The highest average distribution is in red on the left and the red arrow points to the melanoma lineage). Lineages are composed of cell lines from the same area/organ or system of the body with the number of corresponding cell lines indicated in the figure. Data is represented as whisker-box plots with outliers plotted as individual points (Boxes extend from the 25th to 75th percentiles, whiskers from the 10th to 90th percentiles, the solid horizontal line in each box is plotted at the median and the dashed one at the mean).

(B) GEPIA (Gene expression profiling interactive analysis) generated dot plot profiling of TRPV2 gene expression across multiple cancer types and paired normal samples (TCGA tumors *vs* TCGA normal) based on TCGA RNAseq data directly extracted from tissue samples. Cancer types are as follow (T, tumor sample; N, matched normal sample): ACC, Adrenocortical carcinoma (T n=77; N n=-); BLCA, Bladder carcinoma (T n=404; N n=19); BRCA, Breast invasive carcinoma (T n=1085; N n=112); CESC, Cervical squamous cell carcinoma and endocervical adenocarcinoma (T n=306; N n=3); CHOL, Cholangiocarcinoma (T n=36; N n=9); COAD, Colon adenocarcinoma (T n=275; N n=41); DLBC, Diffuse Large B-cell Lymphoma (T n=47; N n=-); ESCA, Esophageal carcinoma (T n=182; N n=13); GBM, Glioblastoma multiform (T n=163; N n=-); HNSC, Head and neck squamous cell carcinoma (T n=519; N n=44); KICH, Kidney chromophobe (T n=66; N n=25); KIRC, Kidney renal clear cell carcinoma (T n=523; N n=72); KIRP, Kidney renal papillary cell carcinoma (T n=286; N n=32); LAML, Acute Myeloid Leukemia (T n=173; N n=-); LGG, Lower grade glioma (T n=518; N n=207); LIHC, Liver hepatocellular carcinoma (T n=369; N n=50); LUAD, Lung adenocarcinoma (T n=483; N n=59); LUSC, Lung squamous cell carcinoma (T n=486; N n=50); MESO, Mesothelioma (T n=87; N n=-); OV, Ovarian serous cystadenocarcinoma (T n=426; N n=-); PAAD, Pancreatic adenocarcinoma (T n=179; N n=4); PCPG, Pheochromocytoma and Paraganglioma (T n=182; N n=3); PRAD, Prostate adenocarcinoma (T n=492; N n=52); READ, Rectum adenocarcinoma (T n=92; N n=10); SARC, Sarcoma (T n=262; N n=2); SKCM, Skin cutaneous melanoma (T n=461; N n=1); STAD, Stomach adenocarcinoma (T n=408; N n=36); TGCT, Testicular germ cell tumor (T n=137; N n=-); THCA, Thyroid carcinoma (T n=512; N n=59); THYM, Thymoma (T n=118; N n=2); UCEC, Uterine corpus endometrial carcinoma (T n=174; N n=13); UCS, Uterine carcinosarcoma (T n=57; N n=-); UVM; Uveal melanoma (T n=79; N n=-).

(C) TCGA_SKCM based GEPIA2 generated dot plot profiling of TRPV2 gene expression across the main melanoma mutational status: BRAF_Hotspot_Mutants (n=149), NF1_Any_Mutants (n=27), RAS_Hotspot_Mutants (n=92), Triple_WT (n=45).

For B and C the box plot shows TRPV2 expression levels in different cancers and normal tissues or melanoma subtypes as whisker-box plots with outliers plotted as individual points (Boxes extend from the 25th to 75th percentiles, whiskers from the 5th to 95th percentiles, the solid horizontal line in each box is plotted at the median).

(D-E) TRPV2 expression was correlated with POU3F2(BRN2) levels, based on mRNA data presented in figure 1G for the cell lines used in this study (E) or based on publicly available expression data for an

extended panel of 83 melanoma cell lines from the CCLE cohort (F).

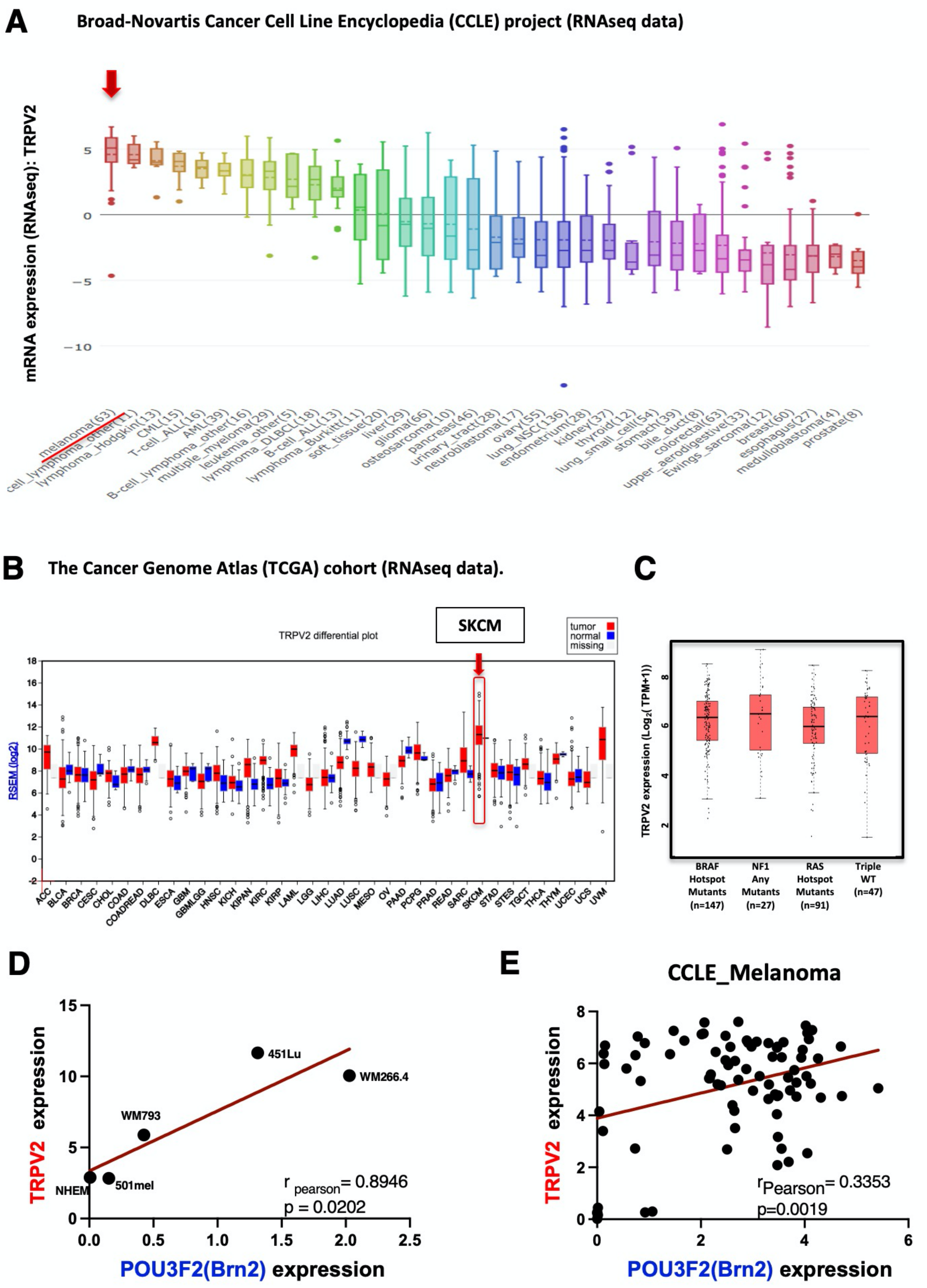

Appendix Figure S2 (related to Figure 2). TRPV2 functional expression and plasma membrane localization correlate with the invasive phenotype of melanoma cells

(A) Representatives Ca^{2+} influx responses to 40 μM Cannabidiol (CBD) stimulation. Data represents means ± SD from at least 15 cells per cell line.

(B) Confocal microphotographs showing the distribution of TRPV2 (red) and stained nuclei (blue) in the indicated melanoma cell lines. The dotted line highlights the membrane edge in the WM793 cell line. Note that TRPV2 distribution at the plasma membrane correlates with the invasive potential of the melanoma cell lines. (Scale bar = 10μm).

(C) Representative kinetics of CBD induced Ca^{2+} influx in melanoma cell lines modified for TRPV2 expression. Non-invasive 501mel cells (left) overexpressing GFP or GFP-TRPV2. WM266.4 (middle) and 451Lu (right) metastatic cells transduced with either control (Ctrl) or TRPV2-targeting sequences V2-1 and V2-2 shRNAs. Data represents means ± SEM of the number of cells indicated on the figures and are representative of at least 3 independent experiments.

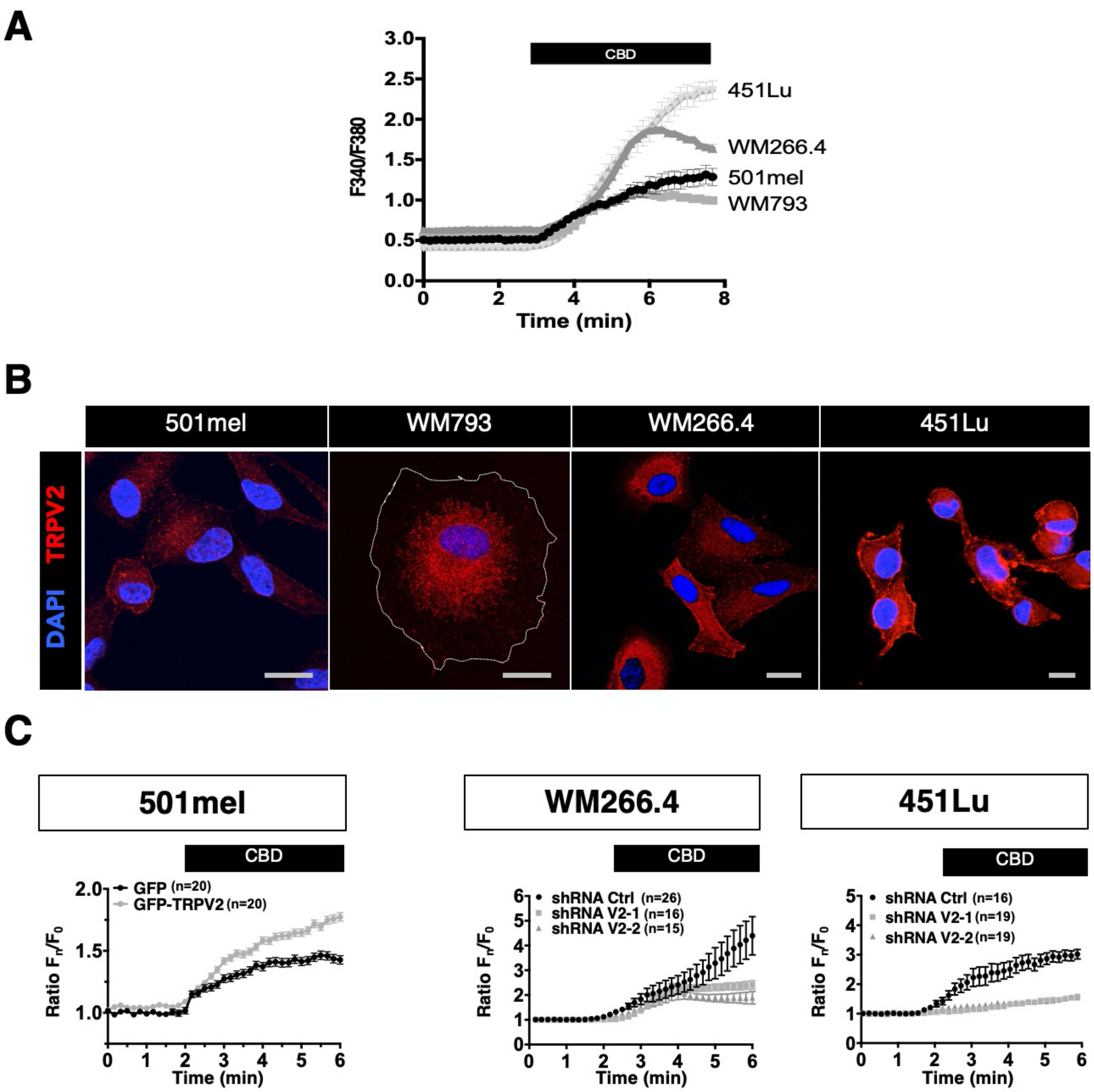

Appendix Figure S3 (related to Figure 3). TRPV2 Ca^{2+}-conducting activity is essential for melanoma tumor cell migration

(A) WM266.4 metastatic melanoma cells migration behavior upon TRPV2 pharmacological inhibition with Tranilast (1mM) assessed in scratch wound healing assays. Left panel shows representative images. Right panel shows gap closure quantification at 24h presented as mean ± SEM (n=5 biological replicates; ****P <0.0001, Unpaired t-test).

(B) Schematic representation of 2D cell migration assays using WM266.4 cells transduced with control (red) or TRPV2 (green) shRNAs, and placed in a 0-5% FCS gradient using an Ibidi migration chamber. Representative fluorescent image showing a typical field of seeded cells being evaluated for 12h, under temperature and CO_2 controlled conditions. (Scale bar = 30µm).

(C) Invasion kinetics of collagen-embedded spheroids based on Figure 3 D-E experiments. Data are presented as mean ± SEM of n>4 independent spheroids for each condition.

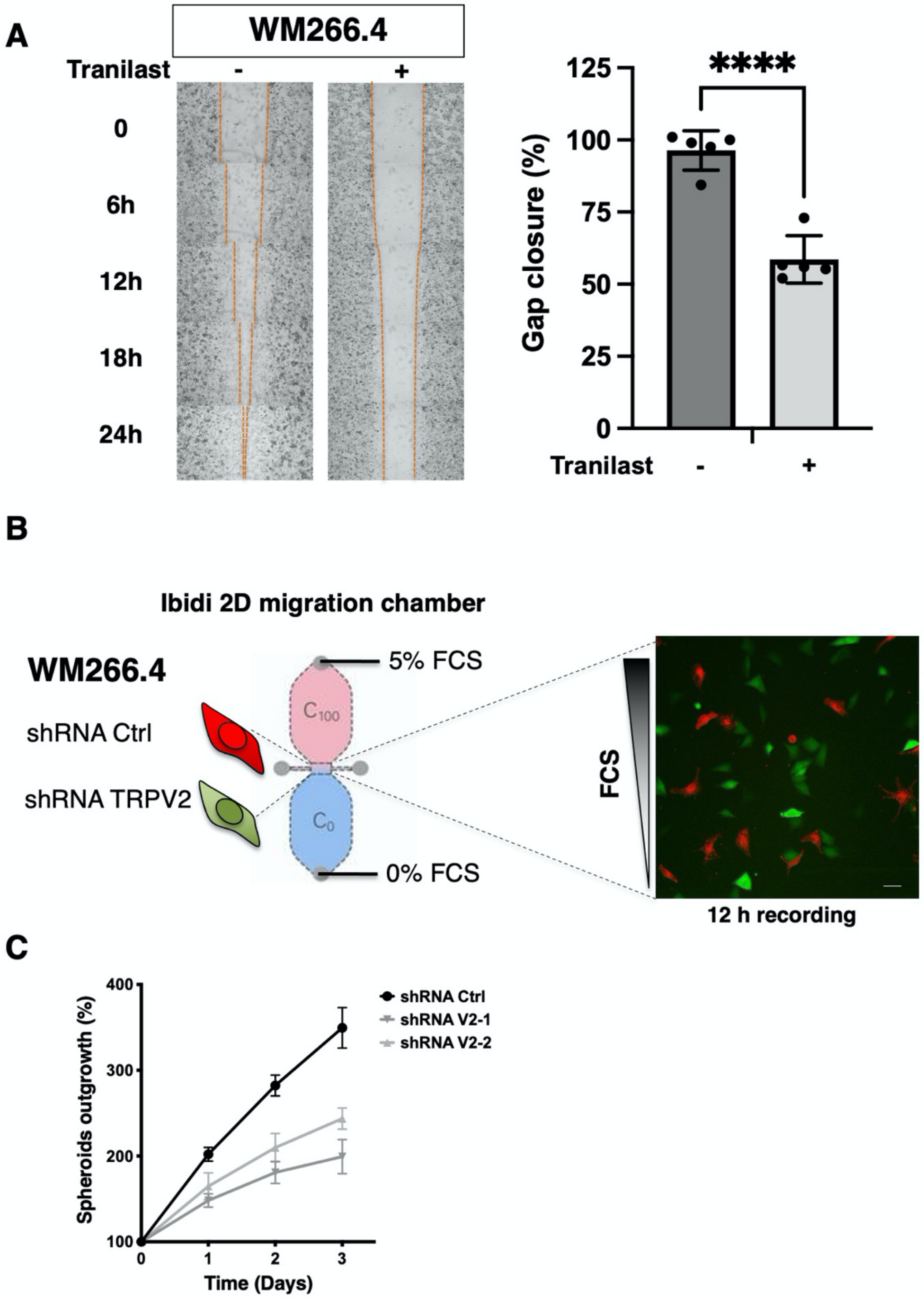

Appendix Figure S4. Modulating TRPV2 expression does not affect pseudo-EMT markers

Representative immunoblots analysis of the indicated “epithelial” and “mesenchymal” markers in control (GFP) or GFP-TRPV2 overexpressing 501mel cells, and in WM266.4 and 451Lu cells expressing either control (ctrl) or TRPV2-targeting (V2-1 or V2-2) shRNAs. HSP60 was used as a loading control. MITF= Microphthalmia-associated transcription factor; E-CAD= E-Cadherin; FN1= Fibronectin; N-CAD= N-Cadherin; SPARC= secreted protein acidic and rich in cysteine; a-SMA= alpha-smooth muscle actin; VIM= Vimentin.

The star indicates the detection of non-phospho, active β-catenin (β-CAT) (non-phosphorylated on Ser33, Ser37 and Thr41 residues, D13A1 Rabbit mAb, Cell Signalling). Note that 501mel cells display high levels of active β-catenin, acting as a suppressor of cell invasion in melanoma (Arozarena *et al*, 2011). WM266.4 cells strongly express the mesenchymal markers tested, suggesting that these cells have completed a mesenchymal transition, while 451lu cells present an intermediate phenotype.

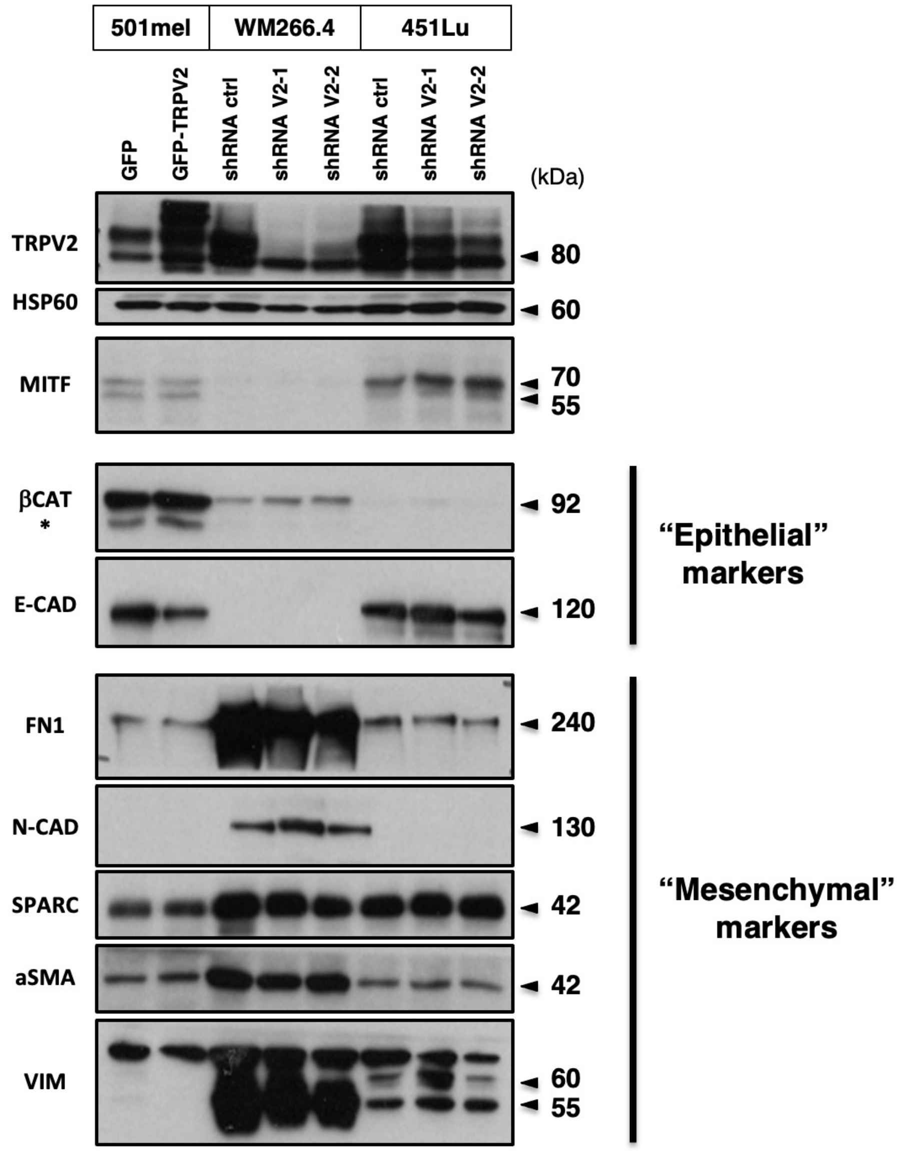

Appendix Figure S5 (related to Figure 4). TRPV2 associates with Vimentin in metastatic melanoma cells

(A) Representative confocal images of low confluence WM266.4 and 451Lu melanoma cells seeded on fibronectin-coated coverslips. Cell nuclei are depicted in blue (DAPI), TRPV2 in red and vimentin (VIM) in green. Bottom panels show magnification of the indicated area and arrows indicate sites of co-localization. (Scale bar = 20μm for top panels and 3μm for bottom panels).

(B) Representative immunoblotting (IB) of reciprocal co-immunoprecipitation (IP) of TRPV2 with vimentin in WM266.4 and 451Lu cells. Experiments were performed three times.

A

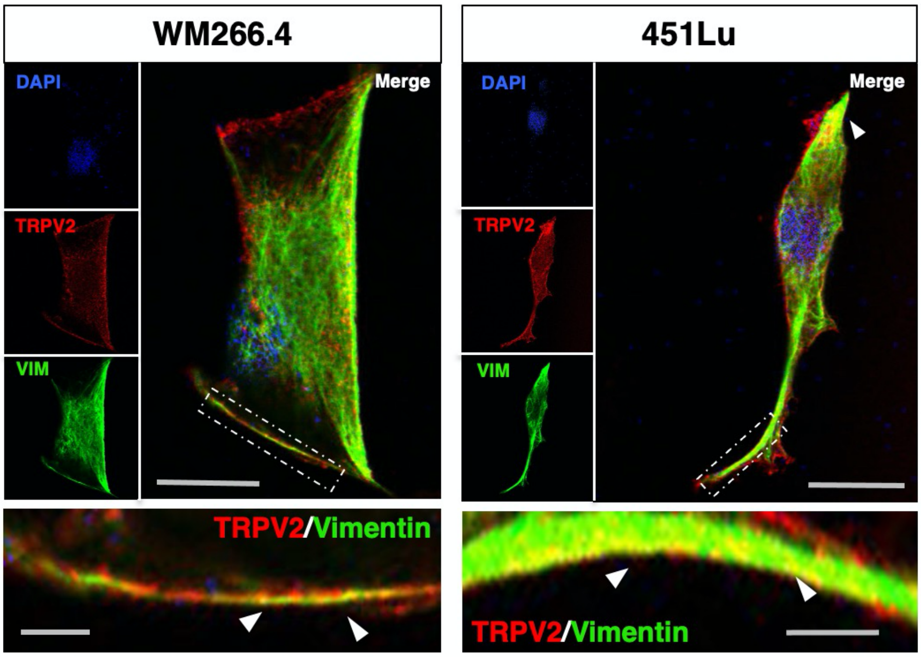

B

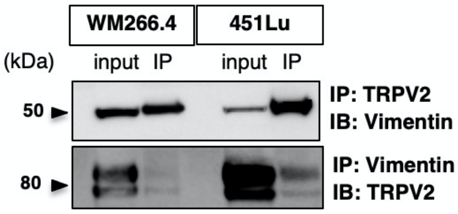

Appendix Figure S6 (related to Figure 5). TRPV2 controls adhesions maturation and turnover through the Ca^{2+}-dependent activation of calpain

(A) Representative vinculin (red) and nuclei (DAPI, blue) staining, used to quantify mature FAs per cell, in cell lines where TRPV2 expression was modulated. (Scale bar = 10μm).

(B) Immunoblotting (IB) assessing the total amount of vinculin following TRPV2 genetic manipulation. β-actin was used as a loading control.

(C & D) IB assessment of the Ca^{2+}-dependency of talin cleavage, an early adhesion protein proteolyzed by calpain. Transfected 501mel cells (C), or wild-type WM266.4 and 451Lu cells (D) were bathed in nominally Ca^{2+}-free (0) or 2 mM Ca^{2+} (2) HBSS external solution. Five minutes prior lysis, cells were treated with either 5 μM Ionomycin, a selective potent Ca^{2+} ionophore allowing normalization of the intracellular [Ca^{2+}] with the extracellular [Ca^{2+}], or with cannabidiol (CBD, 40 μM for 10 min) eliciting a TRPV2-mediated Ca^{2+} influx. Note that following either of these treatments (both resulting in an increased cytosolic [Ca^{2+}]), talin was majoritarly present under its full-length form (Black Arrows) in 0 mM Ca^{2+} cultured cells, whereas a strong occurrence of the 190 kDa calpain-cleaved isoform (Grey Arrows) was observed in the 2 mM Ca^{2+} external solution bathed cells. In the context of TRPV2-dependent Ca^{2+} influx mediated by CBD stimulation, 501mel cells overexpressing GFP-TRPV2 were used instead of GFP cells lacking TRPV2. In all TRPV2-expressing melanoma cells, CBD-induced Ca^{2+} influx leads to talin proteolysis reflecting calpain activation.

(E) Representative immunoblots showing the effect of calpain pharmacological inhibition on the relative levels of full-length (230 kDa; Black Arrow) and the calpain-mediated degradation product (190 kDa, Grey Arrow) of talin in control or TRPV2 overexpressing 501mel cells. Tubulin was used as loading control.

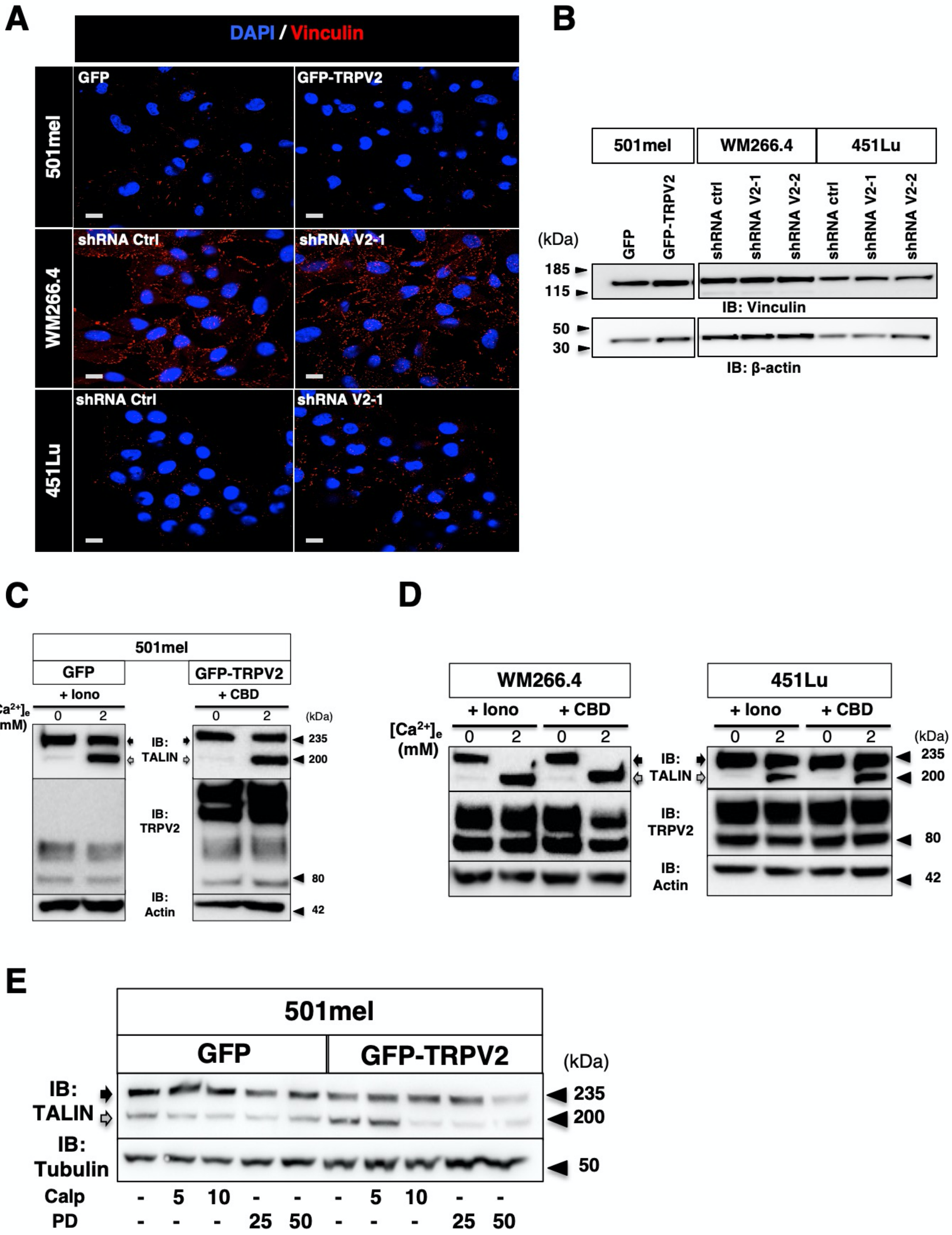
A
DAPI / Vinculin
501mel
GFP
GFP-TRPV2
WM266.4
shRNA Ctrl
shRNA V2-1
451Lu
shRNA Ctrl
shRNA V2-1
B
501mel
WM266.4
451Lu
(kDa)
GFP
GFP-TRPV2
shRNA ctrl
shRNA V2-1
shRNA V2-2
shRNA ctrl
shRNA V2-1
shRNA V2-2
185
115
IB: Vinculin
50
30
IB: β-actin
C
501mel
GFP
GFP-TRPV2
+ Iono
+ CBD
[Ca2+]e (mM)
0 2
0 2
(kDa)
IB: TALIN
235
200
IB: TRPV2
80
IB: Actin
42
D
WM266.4
451Lu
+ Iono
+ CBD
[Ca2+]e (mM)
0 2 0 2
(kDa)
IB: TALIN
235
200
IB: TRPV2
80
IB: Actin
42
E
501mel
GFP
GFP-TRPV2
(kDa)
IB: TALIN
235
200
IB: Tubulin
50
Calp - 5 10 - - - 5 10 - -
PD - - - 25 50 - - - 25 50

Appendix Figure S7 (related to Figure 6). TRPV2 knockdown impairs the lung extravasation of melanoma tumor cells.

Lung extravasation/colonization analysis 24 h after inoculation of 451Lu-LUC cells expressing either ctrl shRNA or TRPV2 targeting shRNA. Scatter plots (Box and whiskers) of normalized BLI photon flux in lungs (Boxes extend from the 25th to 75th percentiles, whiskers from min to max, the horizontal line in each box is plotted at the median and each dot correspond to a single mouse (n=7); ***P* = 0.0082, unpaired *t*-test)

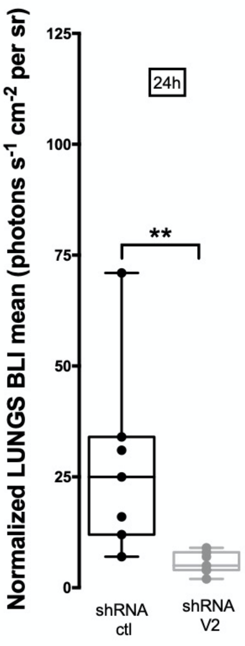

Appendix Figure S8 (related to Figure 6). *Ex vivo* characterization of lung metastasis colonized by human melanoma cells

(A & B) Immunofluorescence analyses of lung sections from mice injected with 451Lu-LUC cells expressing either control or TRPV2 targeting shRNAs. Representative microscopy images of lung sections labeled with DAPI (blue), antibodies against the mouse endothelial cells marker CD31/PECAM-1 (red) and the human melanoma marker MEL (A) or TRPV2 (B) (yellow). (Scale bar = 5 mm).

(C) High magnification pictures of lung metastatic foci showing that human melanoma cells (a, b insets) have colonized the lung parenchyma, as well as the strong expression and plasma membrane localization of endogenous TRPV2 (c, d insets) in these tumor xenografts.

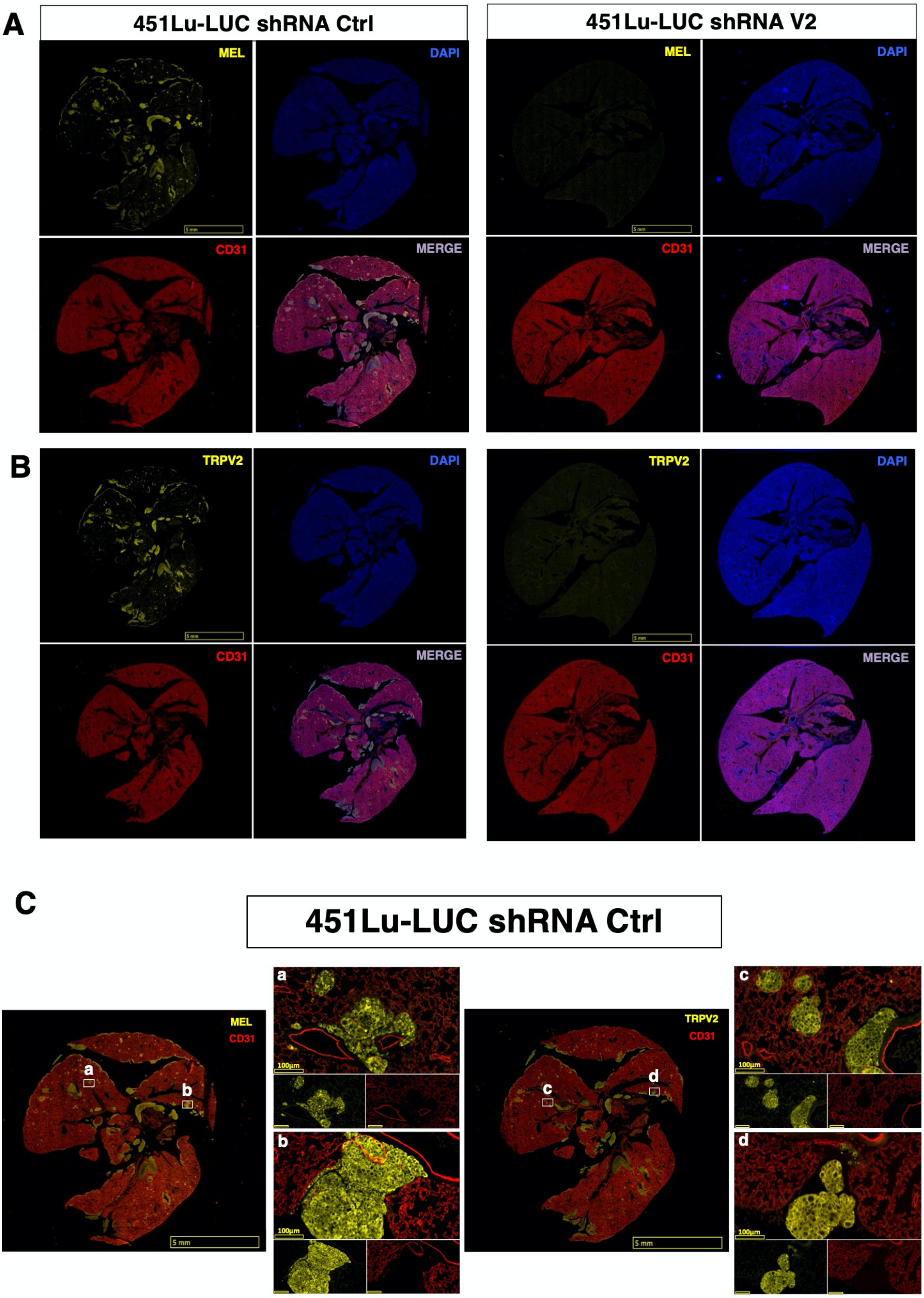
A
451Lu-LUC shRNA Ctrl
451Lu-LUC shRNA V2
MEL
DAPI
CD31
MERGE
B
TRPV2
C
451Lu-LUC shRNA Ctrl
MEL
CD31
TRPV2
a
b
c
d
100µm
5 mm

Appendix Figure S9 (related to Figure 6). Direct comparison of the *in vivo* metastatic potential of TRPV2-modulated melanoma cells in xenografted zebrafish

(A) Schematic representation of the experimental strategy used to evaluate the impact of TRPV2 repression in human melanoma cells on micrometastasis formation in xenografted zebrafish. As control- and TRPV2-shRNA expressing WM266.4 cells were both GFP-labeled, control cells were double-stained with the CM-DiI red fluorescent dye, and equal amounts of both populations were co-injected in the duct of Cuvier of 2 days-old zebrafish embryos. 36h post-transplantation, selected xenografted zebrafish were imaged.

(B) Representative fluorescent microscopy images of zebrafish larvae co-injected with shRNA control (orange) and shRNA TRPV2 (green) melanoma cells. Quantification of tumor burden per embryo shows a significant reduction of the metastatic potential of TRPV2-silenced cells compared to control cells. Eyes autofluorescence was not considered in the quantification. Comparison was done in 9 individual zebrafish larvae. Scatter plots show mean ± SD. ****P* <0.001, Unpaired *t*-test.

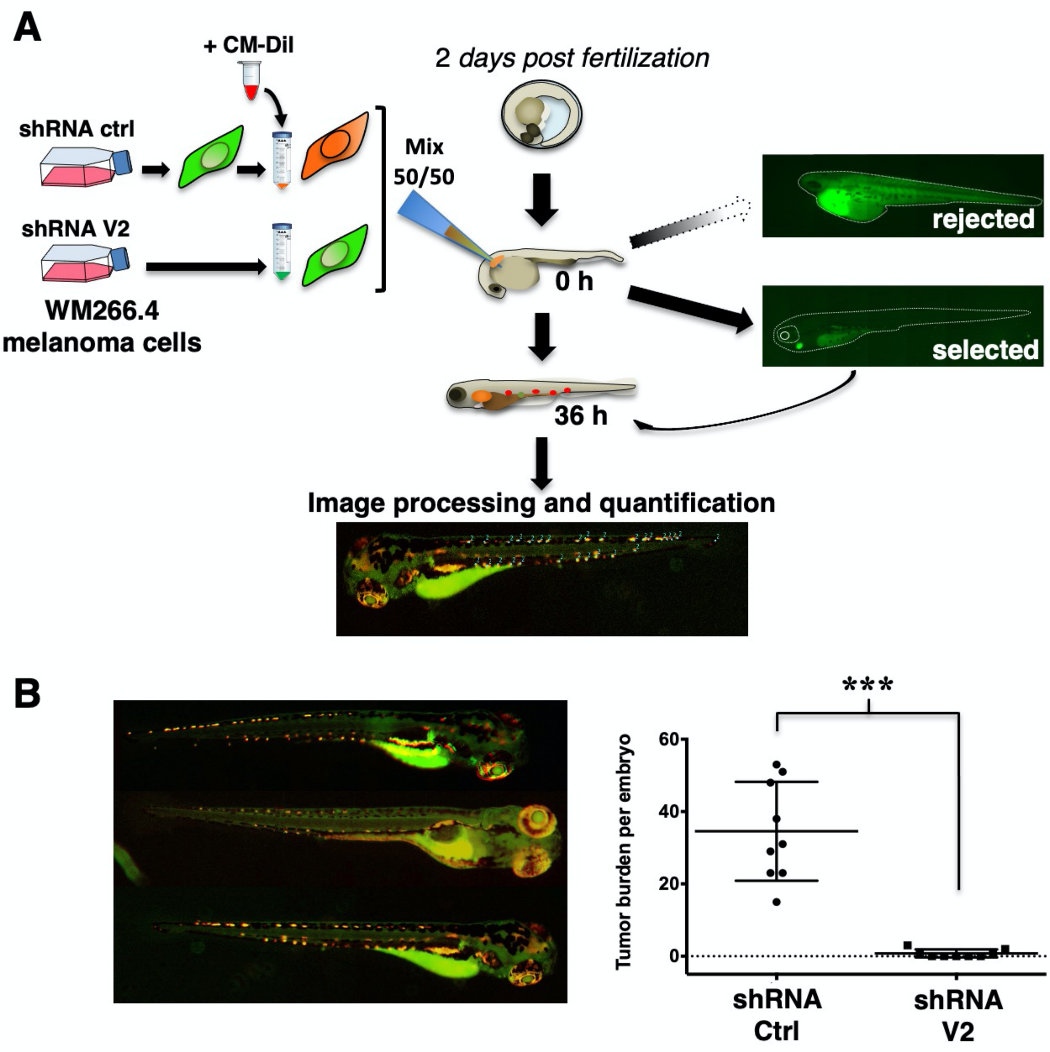

Appendix Figure S10 (related to Figure 7). Antibody validation and quantification method of TRPV2 expression in melanoma patient samples

(A & B) Specificity of the HPA044993 anti-TRPV2 polyclonal antibody (*Sigma*). Non-transfected HEK cells were used as negative control and human TRPV2 transfected HEK cells as positive control. TRPV2 antibody was tested (A) by immunoblotting on WM266.4 and 451Lu melanoma cells either endogenously expressing high levels of TRPV2, or specifically repressed for TRPV2 expression by shRNAs; and (B) by immunohistochemistry on a stage IV melanoma tumor by comparison with its isotype control. (Scale bar = 200µm for HEK and 1mm for Stage IV melanoma).

(C) Global picture of the ME1004c melanoma tissue microarray used to analyze TRPV2 expression.

(D) Schematic representation of the image analysis workflow used for TRPV2 staining intensity quantification.

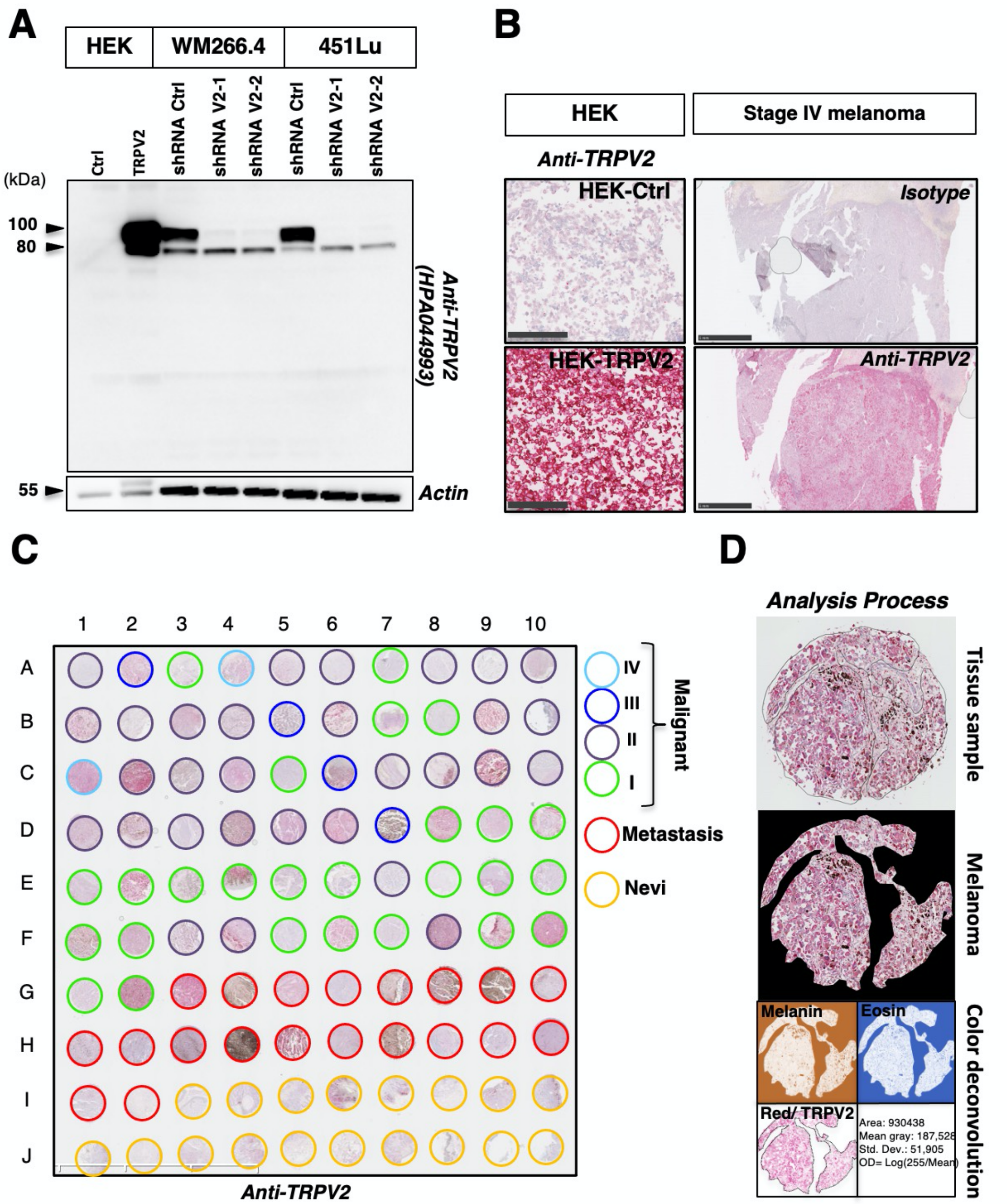
A
HEK
WM266.4
451Lu
Ctrl
TRPV2
shRNA Ctrl
shRNA V2-1
shRNA V2-2
shRNA Ctrl
shRNA V2-1
shRNA V2-2
(kDa)
100
80
55
Anti-TRPV2 (HPA044993)
Actin
B
HEK
Stage IV melanoma
Anti-TRPV2
HEK-Ctrl
Isotype
HEK-TRPV2
Anti-TRPV2
C
1 2 3 4 5 6 7 8 9 10
A B C D E F G H I J
IV
III
II
I
Malignant
Metastasis
Nevi
Anti-TRPV2
D
Analysis Process
Tissue sample
Melanoma
Color deconvolution
Melanin
Eosin
Red/ TRPV2
Area: 930438
Mean gray: 187,528
Std. Dev.: 51,905
OD= Log(255/Mean)

Appendix Figure S11 (related to Figure 7). TRPV2 expression analysis in melanoma patient samples

(A) RNAseq data-based GEPIA generated dot plot profiling TRPV2 differential expression in the TCGA SKCM tumors cohort (n= 461) *versus* [TCGA normal + GTEx normal] datasets (n= 558). Each dot represents a distinct tumor (T) or normal sample (N). Box plot represents the 25th percentile interval and whiskers represent the 95th percentile interval of the distribution. A horizontal line indicates the median value of the expressions. The method for differential analysis is one-way ANOVA, using disease state (Tumor or Normal) as variable for calculating differential expression: The expression data were first log_2(TPM+1) transformed for differential analysis and the log_2FC was defined as median(Tumor) - median(Normal); **P* < 0.01.

(B) Whisker-box plot representing TRPV2 protein expression level according to melanoma stages (TNM grading) in the TMA. (Boxes extend from the 25th to 75th percentiles with Tukey whiskers with outliers plotted as individual points, the horizontal line in each box is plotted at the median).

(C) Immunohistochemistry staining of TRPV2 on grade I (Clark level II Superficial Spreading Melanoma with a Breslow index of 0.4mm) and grade IV (Clark level IV Nodular melanoma with a Breslow index of 6mm) melanoma samples issued from the Rennes University Hospital (CHU) tumor biobank (Centre de ressources biologiques humaines - CRB Santé). (Scale bar = 100µm)

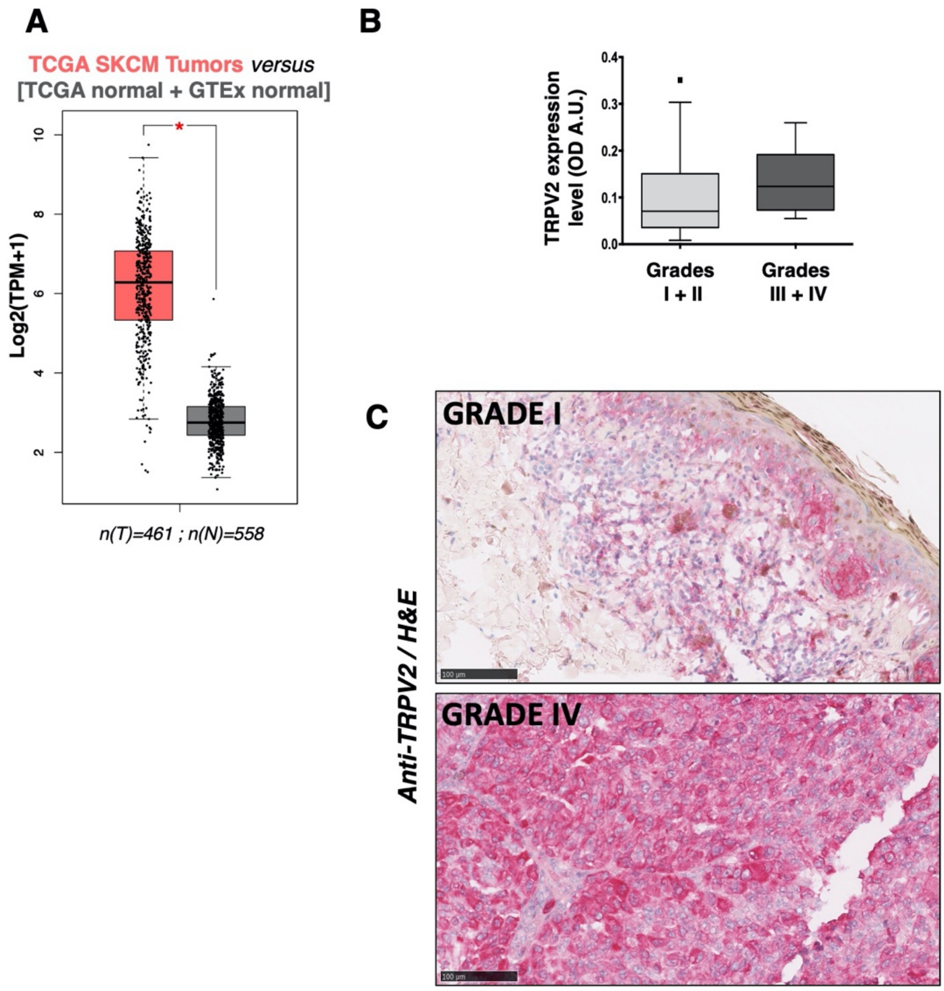

Appendix Table S1. List of PCR primers used in this study.

Gene	Amplicon size	Forward primer sequence (5'-3')	Reverse primer sequence (5'-3')
TBP	161	CGGAGAGTTCTGGGATTGT	GGTTCGTGGCTCTCTTATC
TRPV1	120	GACCTGTGCCGTTTCA	CCTGTGCGACGTGGACTCA
TRPV2	199	GGAATACACAGAGGGCTCCA	CCTCTTCTCAATGGCGATGT
TRPV3	226	ACGAGGCAACAACATCCTTC	CCGCTTCTCCTTGATCTCAC
TRPV4	190/370	CCCGTGAGAACACCAAGTTT	AGTTCATTGATGGGCTCCAC
TRPV6	208	GGATCTGCGGACGGGAGTA	CGAGACACTGAGGGCATAGGA
TRPC1	201	TGGGATGATTTGGTCAGACA	TCTGCCACCAGTGTAGGATG
TRPC6	121	CCTTGCTGTTGCCATTGGAC	GAAGGAGGCTGCGTGTGCTA
TRPM2	303	GGCAGTGGAAGCCTTCAGAT	GATAAAGCGGCTGCGTGAAG
TRPM7	226	AATAATCGGAGGTCTGGCCG	AGCGCTTGGTTTCTGGATCA
TRPA1	140	TGCATGTTGCATTCCACAGAAG	TTGAGGGCTGTAAGCGGTTCATA
STIM1	109	TGTGGAGCTGCCTCAGTATG	CTTCAGCACAGTCCCTGTCA
STIM2	114	GTCTCCATTCCACCCTATCC	GGCTAATGATCCAGGAGGTT
ORAI1	161	ATGAGCCTCAACGAGCACT	GTGGGTAGTCGTGGTCAG
SK3	174	TGGACACTCAGCTCACCAAG	GTTCCATCTTGACGCTCCTC
BRN2	98	GTAACTGTCAAATGCGCGGC	GAGGTGAGCAGGCTGTAGTG

Each primer pair was designed and validated *in silico* using primer3plus and primer-blast software (http://www.bioinformatics.nl/cgi-bin/primer3plus/primer3plus.cgi, http://www.ncbi.nlm.nih.gov./tools/primer-blast/). Primers were designed to localize at intron/exon junctions of sequences commune to all variants of each gene.

Note that the TRPV4 specific primer pair use for RT-PCR amplify TRPV4 variants 1, 4 and 5 with an amplicon size of 370 bp and TRPV4 variants 2 and 3 with an amplicon size of 190 bp (Arniges *et al*, 2006). For qPCR experiments, an alternative primer pair was used amplifying all variants with the same amplicon size of 76 bp (Fwd 5'-CTACGCTTCAGCCCTGGTCTC-3'; Rev 5'-GCAGTTGGTCTGGTCCTCATTG-3' (Spinsanti *et al*, 2008)).

Appendix Table S2. Summary of the exact P value obtained for the multiple comparisons performed on the data presented in Fig 3A and 5B,D.

Fig 3 & 5	WM266.4		451Lu	
	shRNA ctrl vs shRNA V2-1	shRNA ctrl vs shRNA V2-2	shRNA ctrl vs shRNA V2-1	shRNA ctrl vs shRNA V2-2
3A Migration	** P=0,009	** P=0,0034	*** P=0,0003	**** P=<0,0001
3A Invasion	** P=0,0013	** P=0,0017	**** P=<0,0001	**** P=<0,0001
5B (calpain activity)	**P=0,0068	**P=0,0037	**P=0,0033	**P=0,001
5D (Talin cleavage)	*** P=0,0007	*** P=0,0005	*P=0,0388	*P=0,0265

Appendix Table S3. Summary of the exact P value obtained for the multiple comparisons performed on the data presented in EV Fig 2B.

EV Fig 2B	Significant?	Summary	Adjusted P Value
GFP vs. GFP + 5µM Calp	No	ns	>0,9999
GFP vs. GFP + 10µM Calp	No	ns	0,0712
GFP vs. GFP + 25µM PD	No	ns	>0,9999
GFP vs. GFP + 50µM PD	No	ns	0,3324
GFP vs. GFP-TRPV2	Yes	**	0,0025
GFP-TRPV2 vs. GFP-TRPV2 + 5µM Calp	No	ns	>0,9999
GFP-TRPV2 vs. GFP-TRPV2+ 10µM Calp	Yes	***	0,0002
GFP-TRPV2 vs. GFP-TRPV2 + 25µM PD	Yes	*	0,0121
GFP-TRPV2 vs. GFP-TRPV2 + 50µM PD	Yes	***	0,0001

Appendix References

Arniges M, Fernandez-Fernandez JM, Albrecht N, Schaefer M, Valverde MA (2006) Human TRPV4 channel splice variants revealed a key role of ankyrin domains in multimerization and trafficking. *J Biol Chem* 281: 1580-1586

Arozarena I, Bischof H, Gilby D, Belloni B, Dummer R, Wellbrock C (2011) In melanoma, beta-catenin is a suppressor of invasion. *Oncogene* 30: 4531-4543

Spinsanti G, Zannolli R, Panti C, Ceccarelli I, Marsili L, Bachiocco V, Frati F, Aloisi AM (2008) Quantitative Real-Time PCR detection of TRPV1-4 gene expression in human leukocytes from healthy and hyposensitive subjects. *Mol Pain* 4: 51
