# Supplementary material for: The mechanosensitive TRPV2 calcium channel promotes human melanoma invasiveness and metastatic potential
Source: EMBO Rep. 2023 Feb 6;24(4):e55069. doi: 10.15252/embr.202255069 (PMC10074106; doi:10.15252/embr.202255069)
Supplement: Supplementary file 2 — Expanded View Figures PDF [file EMBR-24-e55069-s002.pdf]

Expanded View Figures

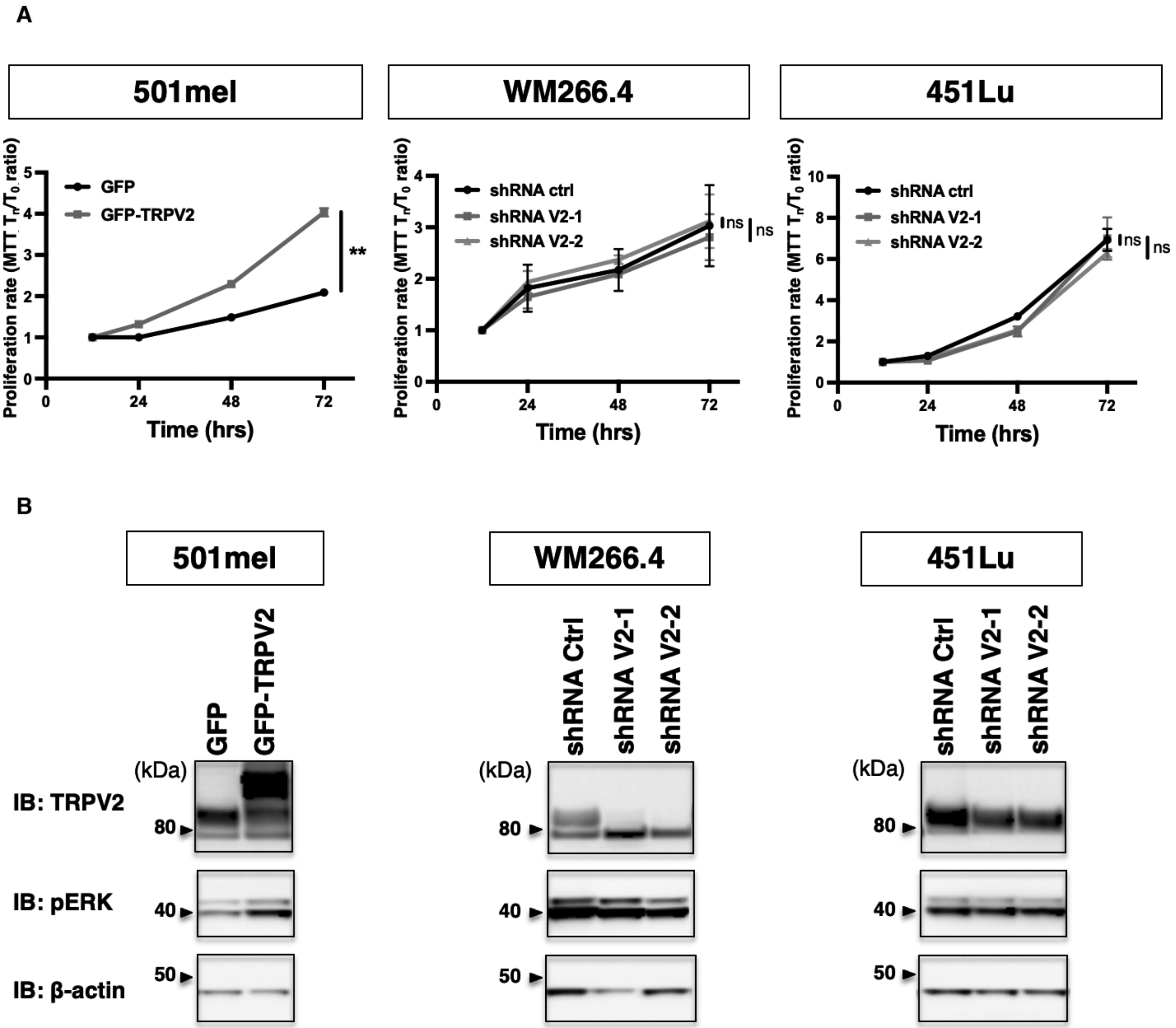

Figure EV1. TRPV2 is dispensable for melanoma tumor cell proliferation (relative to Fig 3).

A Representative proliferation curves comparing the effect of TRPV2 overexpression in 501mel cells, or TRPV2 repression in WM266.4 and 451Lu cells, measured by MTT at 12, 24, 48, or 72 h. Each data point represents the mean ± SEM of $n = 3$ biological replicates with two-way ANOVA multiple comparisons test results for the 72 h time points (**$P = 0.0038$ for 501mel GFP vs. 501mel GFP-TRPV2; ns $P = 0.8422$ for WM266.4 shRNA ctrl vs. ShRNA V2-1, ns $P = 0.9491$ for WM266.4 shRNA ctrl vs. ShRNA V2-2, ns $P = 0.9982$ for 451Lu shRNA ctrl vs. ShRNA V2-1, ns $P = 0.5755$ for 451Lu shRNA ctrl vs. ShRNA V2-2).

B Immunoblotting (IB) of TRPV2, Thr202/Tyr204 Phospho-p44/42 MAPK (pERK), and the β-actin as a loading control in melanoma cell lines modified for TRPV2 expression.

Source data are available online for this figure.

 © 2023 The Authors

Figure EV2. TRPV2 promotes melanoma cell migration through the recruitment of Calpain activity (relative to Fig 5).

A Representative gap closure kinetics from wound-healing assays performed on 501mel cells control (GFP) and 501mel cells overexpressing TRPV2 (GFP-TRPV2) treated either with vehicle, 10 μM calpeptin or 50 μM PD150606. Each data point represents the mean ± SEM of $n = 4$ technical replicates.

B Quantification of the impact of calpain pharmacological inhibition on TRPV2-dependent cell migration. Cell migration speed was assessed as the slope of gap closure kinetics measured for the first 24 h of the wound-healing assays to avoid potential proliferation-induced effects. Histogram illustrates migration speed normalized to 501mel GFP control cells presented as mean ± SEM of $n = 4$ biological replicates with statistical analysis performed using the Kruskal–Wallis test followed by Dunn's multiple comparisons tests (*$P < 0.05$; **$P < 0.01$; ***$P < 0.001$, ns = nonsignificant; See Appendix Table S3 for exact P-values).

C Representative cell migration kinetics from xCELLigence CIM-plate assays performed on GFP-transfected shRNA Ctrl WM266.4 cells, GFP-transfected shRNA V2 WM266.4 cells and shRNA V2 WM266.4 cells transfected with constitutively active Calpain-2 (GFP-CAPN2^{S50E}). Each data point represents the mean ± SD of the Cell Index—a unit automatically computerized by the xCELLigence software—resulting from technical replicates in two independent wells recorded in parallel from the same CIM-plate. Here, we did not use wound-healing assays due to the specific context of transient transfection and because active calpain expression modifies cell adhesion properties making it difficult to obtain nice cell monolayers with delimited wounds.

D Quantification of the ability of active calpain forced expression to rescue the cell migration defect induced by TRPV2 silencing. Cell migration speed was assessed as the slope of the Cell index during the exponential phase. Histogram illustrates cell migration normalized to WM266.4 shRNA Ctrl cells presented as mean ± SEM of $n = 3$ biological replicates with statistical analysis performed using the Kruskal–Wallis test followed by Dunn's multiple comparisons test (***$P = 0.0004$ and ns $P = 0.1484$).

Source data are available online for this figure.

© 2023 The Authors

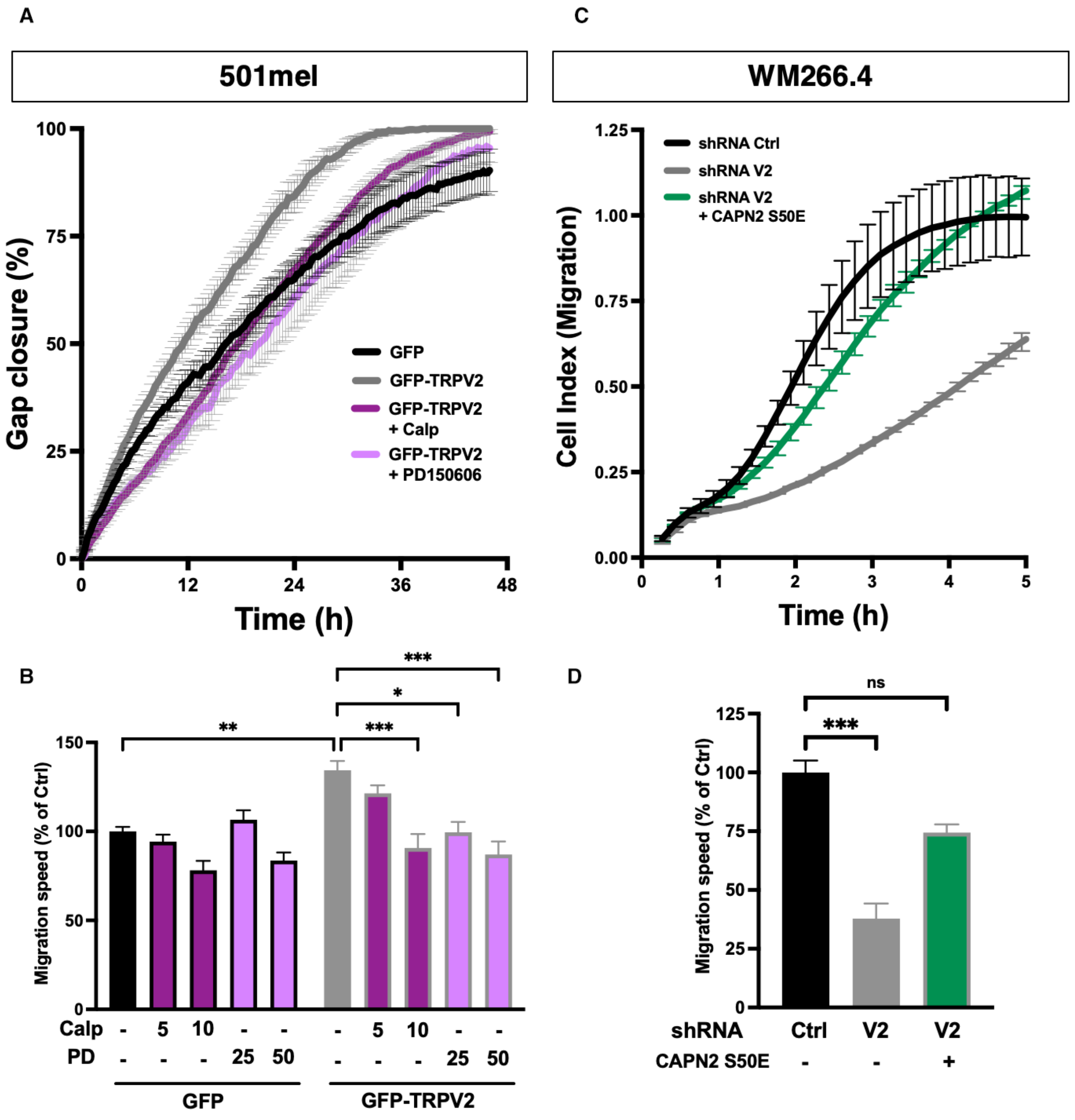

Figure EV2.

 © 2023 The Authors

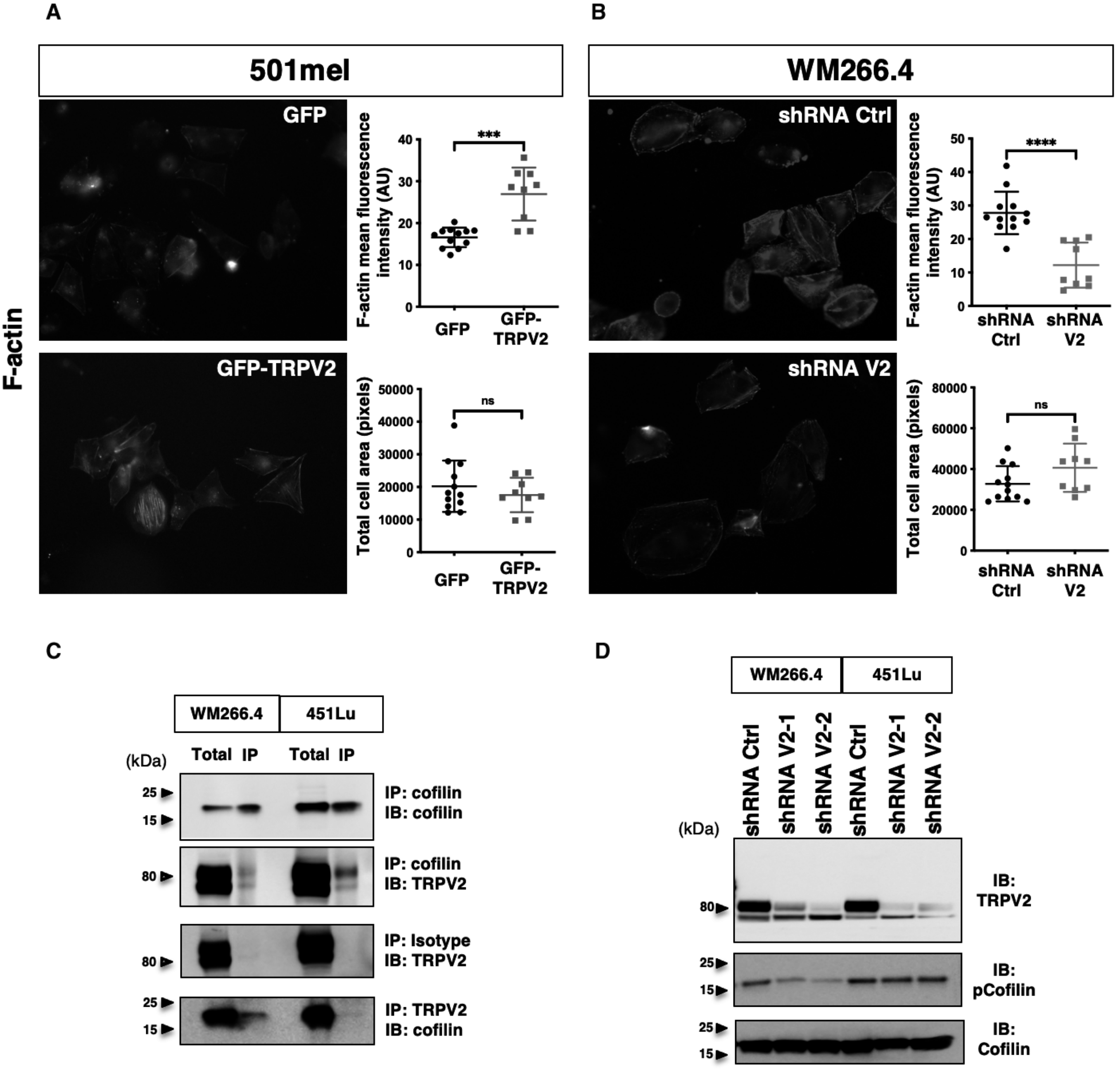

Figure EV3. TRPV2 Regulates Actin cytoskeleton remodeling and cofilin activation.

A, B Fluorescent microscopy images showing live F-actin polymerization in control (GFP) or GFP-TRPV2 overexpressing 501mel cells (A), and in control (Ctrl) or TRPV2-targeting shRNAs transduced WM266.4 cells (B). Quantification of F-actin fluorescent intensity (top plot) and cell area (bottom plot) in both populations. Data are presented as scatter dot plots with mean ± SD, each dot representing mean F-actin fluorescence intensity or mean area per cell in n = 13–16 fields from three independent experiments (***P = 0.0005 and ns P = 0.7021 for 501mel, ****P < 0.0001 and ns P = 0.1308 for WM266.4, the Mann–Whitney test).

C Reverse co-immunoprecipitation (IP) experiments showing a physical interaction between TRPV2 and cofilin in the WM266.4 and 451Lu metastatic melanoma cell lines. Co-IP experiments were performed three times and the immunoblots (IB) show typical results.

D Immunoblot assessment of cofilin activation by measuring total and Ser3-phosphorylated cofilin levels in control (shRNA Ctrl) and TRPV2-silenced (shRNA V2) WM266.4 and 451Lu cells.

Source data are available online for this figure.

Figure EV4. TRPV2 overexpression promotes widespread metastatic colonization of melanoma tumor cells (relative to Fig 6).

A *Ex vivo* BLI measurements for each necropsy collected organ of representative mice xenografted with either GFP control or GFP-TRPV2 overexpressing 501mel-LUC cells.

B Box and whiskers plot shows the number of metastatic foci per organ counted at necropsy of mice xenografted with GFP (black) or GFP-TRPV2 (gray) overexpressing 501mel-LUC cells. Boxes extend from the 25th to 75th percentiles, whiskers from min to max, and each dot correspond to a single mouse (n = 6–7).

Source data are available online for this figure.

© 2023 The Authors

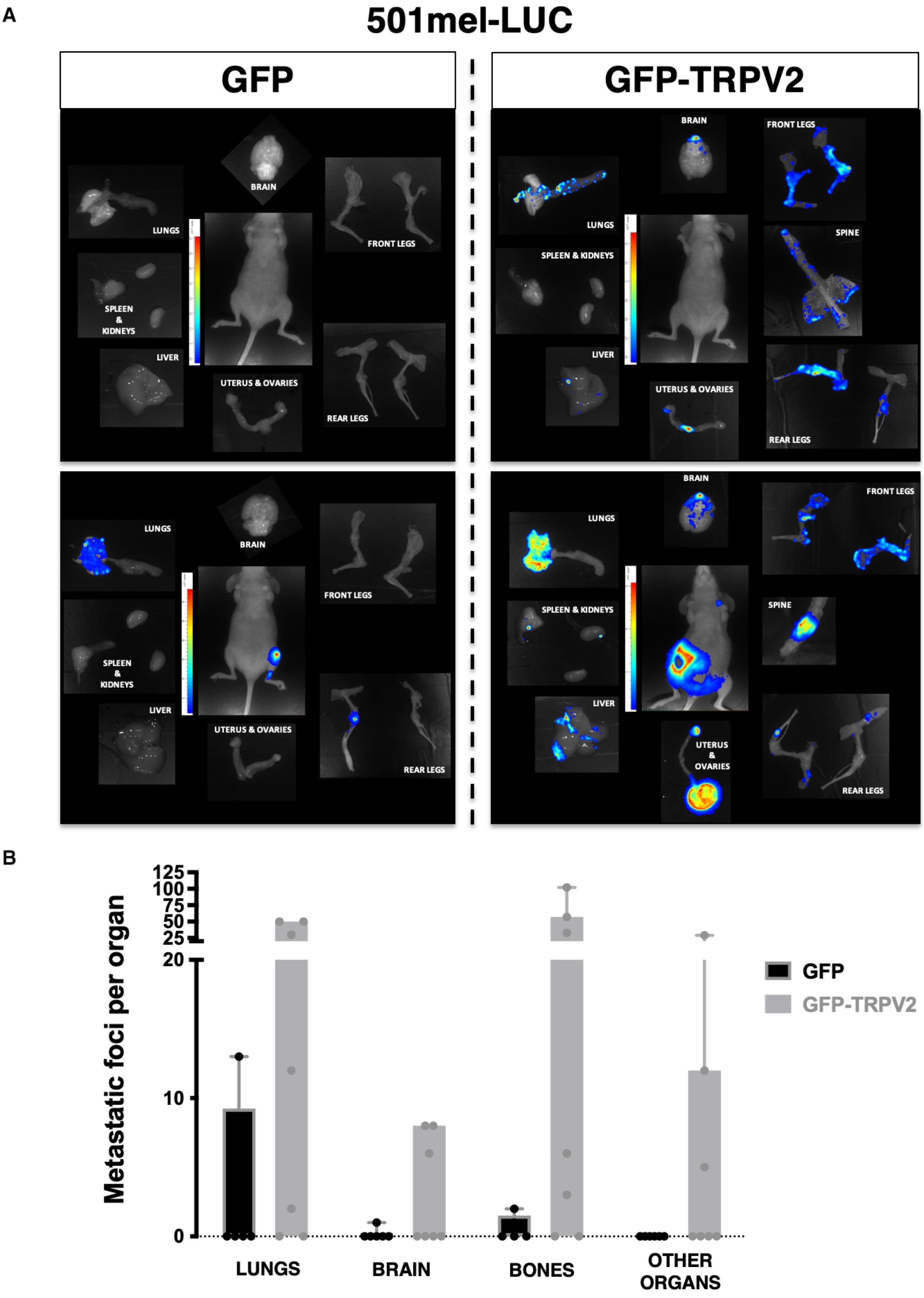
A
501mel-LUC
GFP
GFP-TRPV2
BRAIN
LUNGS
FRONT LEGS
SPLEEN & KIDNEYS
LIVER
UTERUS & OVARIES
REAR LEGS
SPINE
B
Metastatic foci per organ
125
100
75
50
25
20
10
0
LUNGS
BRAIN
BONES
OTHER ORGANS
GFP
GFP-TRPV2

Figure EV4.

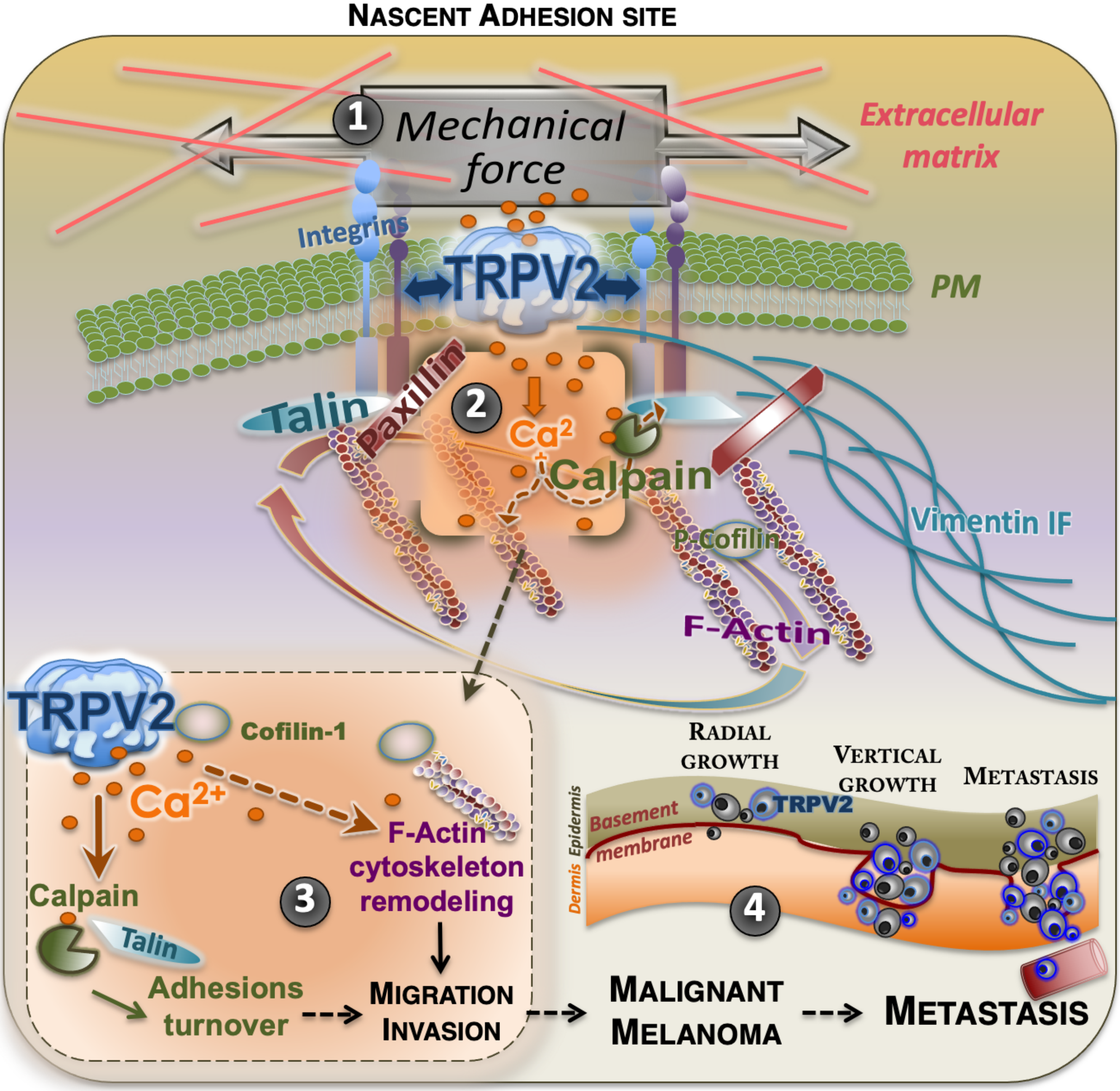

Figure EV5. Mechanistic model of TRPV2 control over metastatic melanoma cells dissemination through the dynamic regulation of nascent adhesion sites.

The essential role of TRPV2 in melanoma migration and invasion could be explained by the newly identified pro-invasive properties of this mechanosensitive channel. Indeed, cancer cells and their associated microenvironment generate considerable mechanical forces applied onto the plasma membrane (PM) (1). These changes in PM tension regulate cell shape and movement. In malignant melanoma cells, the TRPV2 channel is recruited to the PM within paxillin-containing early adhesion structures, and its constitutive activation elicits a subplasmalemmal localized Ca^{2+} ions uptake (2). TRPV2-mediated Ca^{2+} influx triggers the activation of the intracellular Ca^{2+}-dependent cysteine protease, calpain (3). The cleavage of its substrate, the early adhesion protein talin linking membrane integrins and cytoskeleton, *in fine* prompts the disassembly of a subset of adhesion complexes and facilitates cell-extracellular matrix (ECM) contact sites plasticity. Induction, selection, and maturation of nascent adhesion complexes at the cell leading edge serve as sampling the local ECM to select traction points producing forces that will drive the cell body forward. To further regulate the maturation of these adhesion structures along with the remodeling of the cytoskeleton, TRPV2 directly interacts with both the intermediate filament (IF) vimentin network, and the actin severing factor cofilin-1, a central regulator of F-actin dynamics. TRPV2-induced signaling promotes the spatial and temporal accumulation of F-actin bundles to improve advanced melanoma cell motility. Therefore, TRPV2 channel-mediated Ca^{2+} influx tunes the plasticity of the melanoma tumor cell by locally controlling adhesion complexes maturation and cytoskeleton remodeling, potentiating the migratory and invasive behaviors of these malignant cells (4).

 © 2023 The Authors
