# Supplementary figures and images for: The mechanosensitive TRPV2 calcium channel promotes human melanoma invasiveness and metastatic potential
Source: EMBO Rep. 2023 Feb 6;24(4):e55069. doi: 10.15252/embr.202255069 (PMC10074106; doi:10.15252/embr.202255069)

## Slide 1
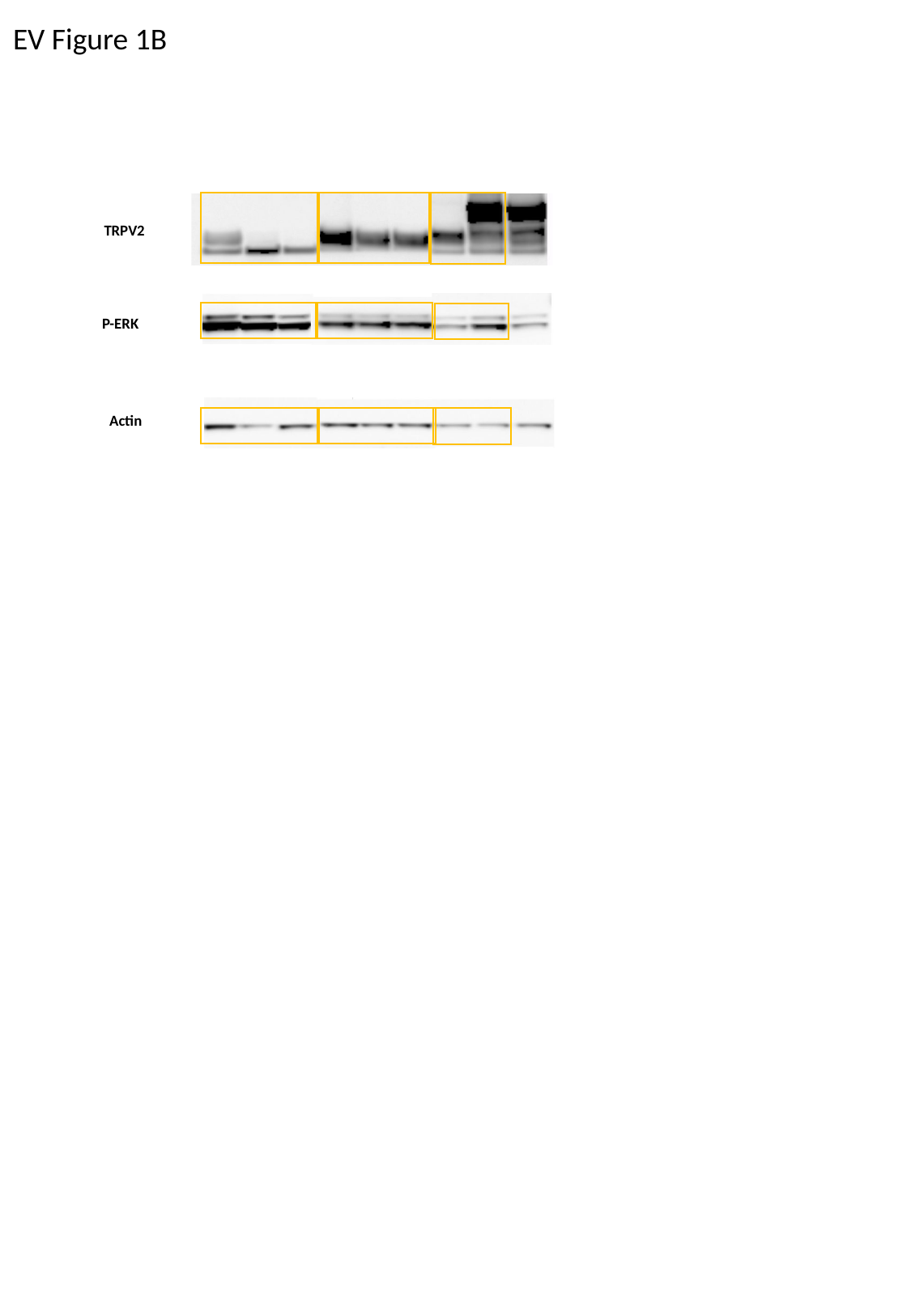

EV Figure 1B
TRPV2
P-ERK
Actin

Supplement: Supplementary file 3 — Source Data for Expanded View [file EMBR-24-e55069-s005.zip › EMBOR-2022-55069V1_SourceDataForEVfigures1/EMBOR-2022-55069V1_SourceDataForEV1B.pptx]

## Slide 1
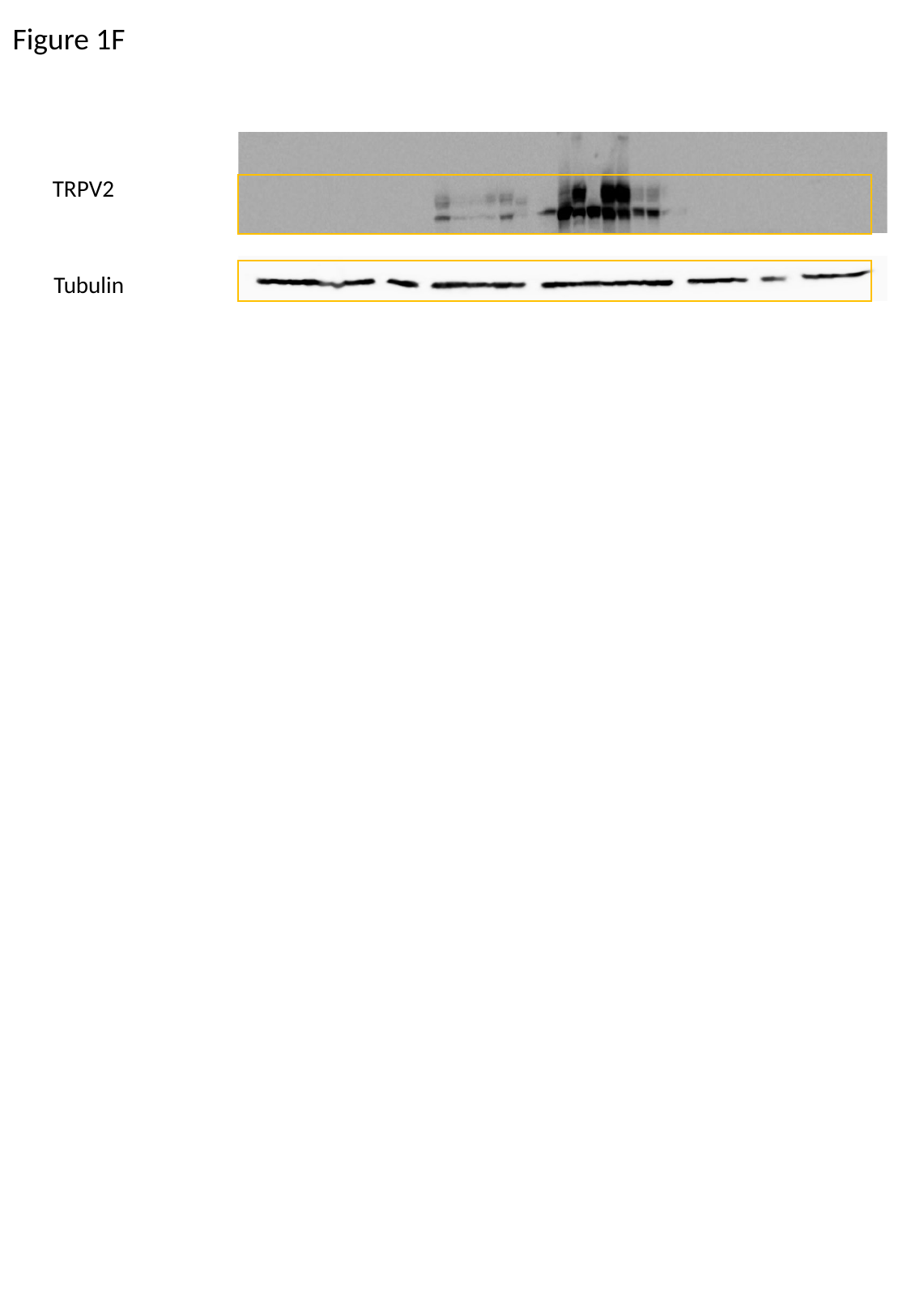

Figure 1F
TRPV2
Tubulin

Supplement: Supplementary file 5 — Source Data for Figure 1 [file EMBR-24-e55069-s012.zip › EMBOR-2022-55069V1_SourceDataForFigure1F.pptx]

## Slide 1
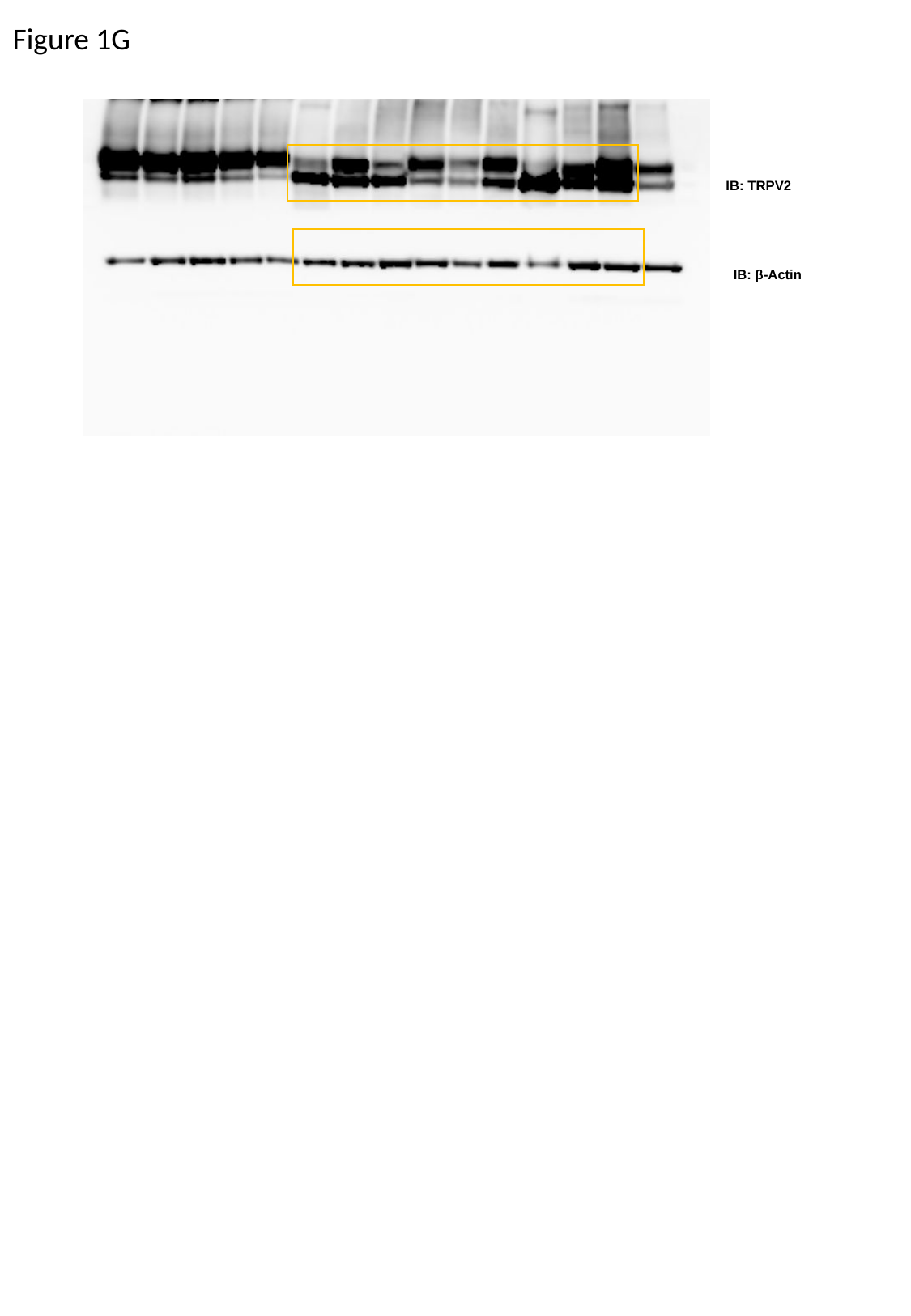

Figure 1G
IB: TRPV2
IB: β-Actin

Supplement: Supplementary file 5 — Source Data for Figure 1 [file EMBR-24-e55069-s012.zip › EMBOR-2022-55069V1_SourceDataForFigure1G.pptx]

## Slide 1
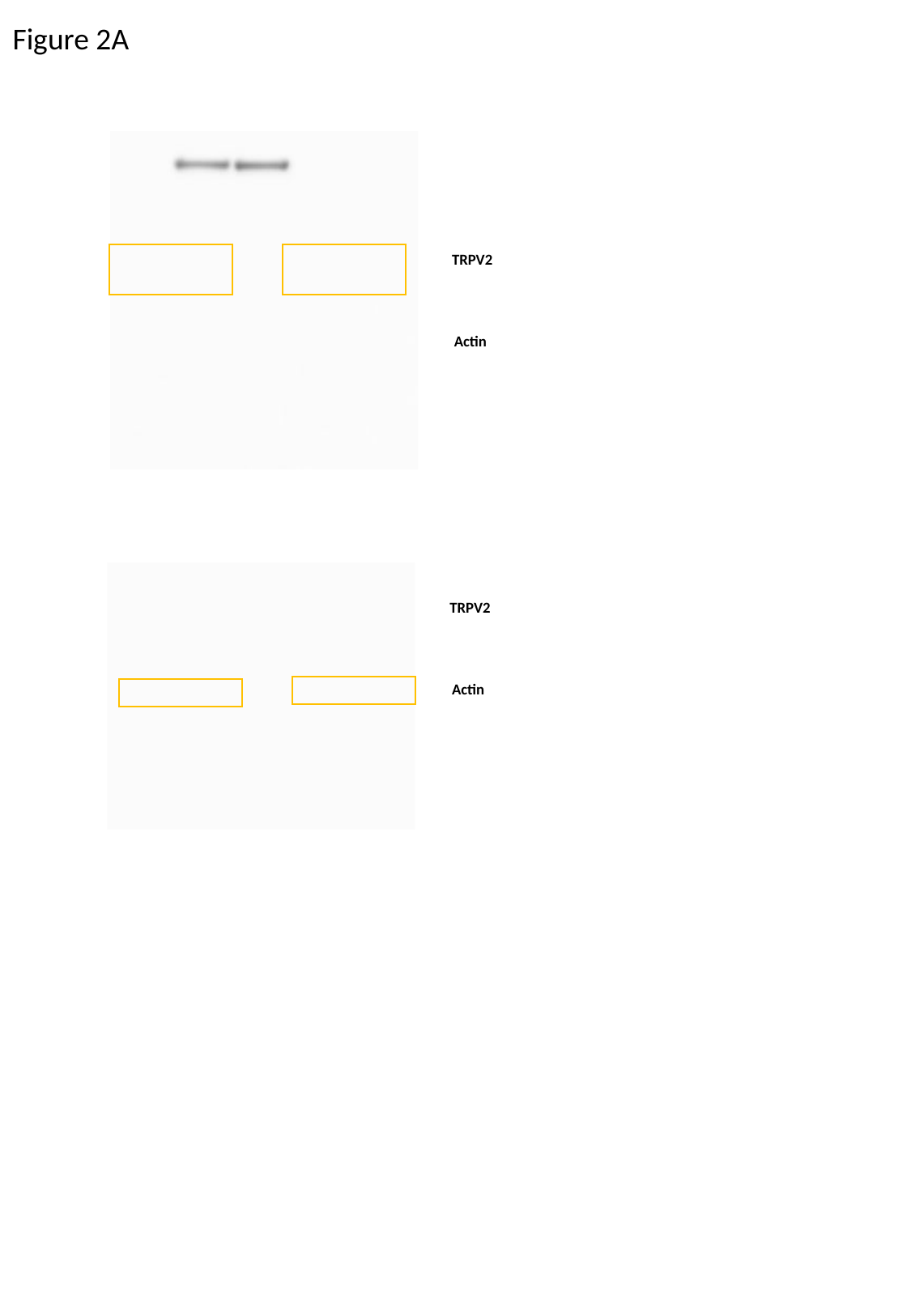

Figure 2A
TRPV2
Actin
TRPV2
Actin

Supplement: Supplementary file 6 — Source Data for Figure 2 [file EMBR-24-e55069-s006.zip › EMBOR-2022-55069V1_SourceDataForFigure2A.pptx]

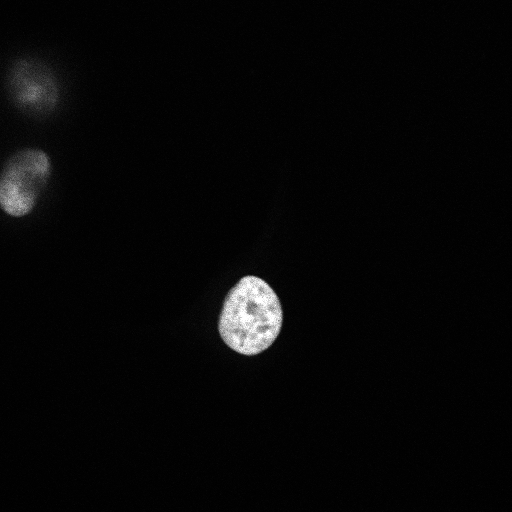

Supplement: Supplementary file 8 — Source Data for Figure 4 [file EMBR-24-e55069-s009.zip › 4A/451LU/451Lu_Act-V2_13/13.tif]

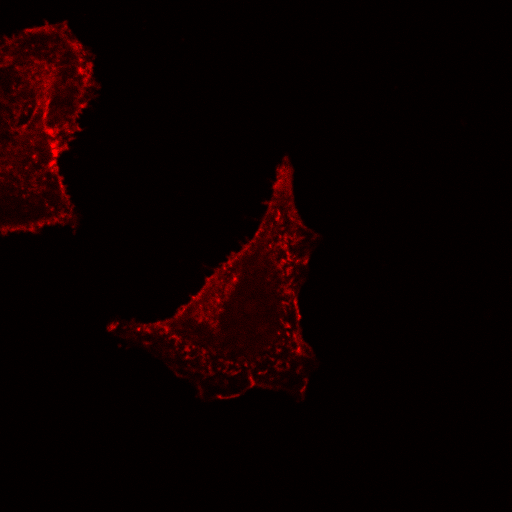

Supplement: Supplementary file 8 — Source Data for Figure 4 [file EMBR-24-e55069-s009.zip › 4A/451LU/451Lu_Act-V2_13/Actin-13.tif]

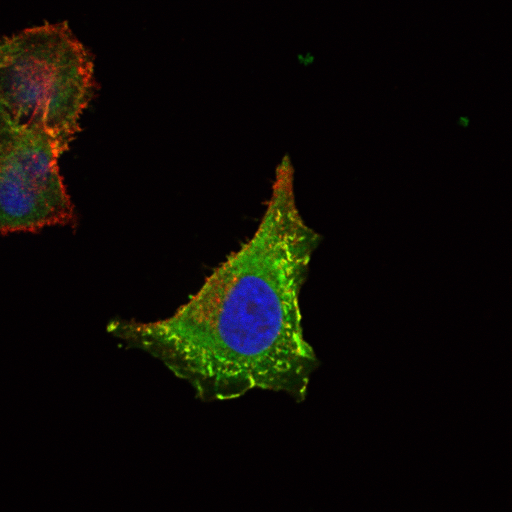

Supplement: Supplementary file 8 — Source Data for Figure 4 [file EMBR-24-e55069-s009.zip › 4A/451LU/451Lu_Act-V2_13/Composite (RGB)-13.tif]

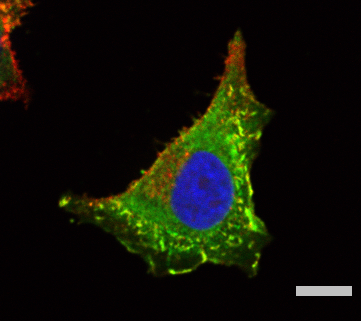

Supplement: Supplementary file 8 — Source Data for Figure 4 [file EMBR-24-e55069-s009.zip › 4A/451LU/451Lu_Act-V2_13/Composite (RGB)-13_scaled.tif]

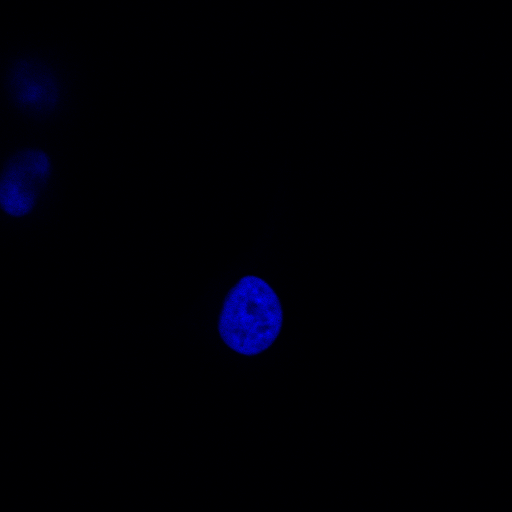

Supplement: Supplementary file 8 — Source Data for Figure 4 [file EMBR-24-e55069-s009.zip › 4A/451LU/451Lu_Act-V2_13/Dapi-13.tif]

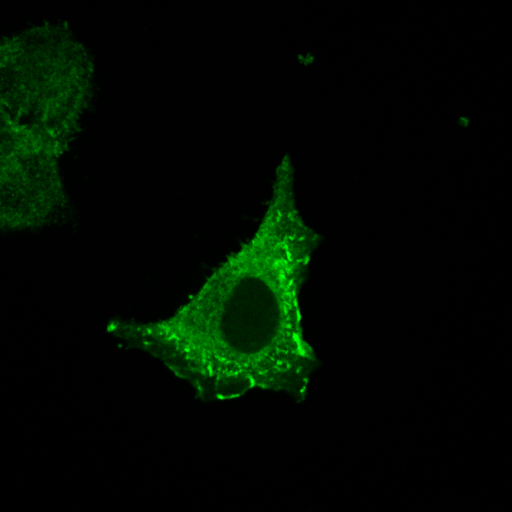

Supplement: Supplementary file 8 — Source Data for Figure 4 [file EMBR-24-e55069-s009.zip › 4A/451LU/451Lu_Act-V2_13/TRPV2-13.tif]

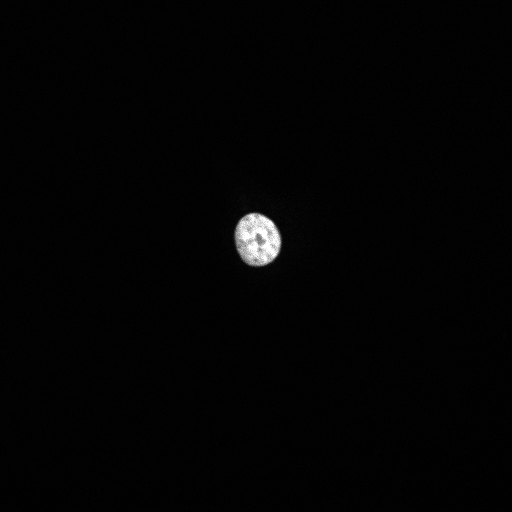

Supplement: Supplementary file 8 — Source Data for Figure 4 [file EMBR-24-e55069-s009.zip › 4A/451LU/451Lu_FAK-V2_11/11.tif]

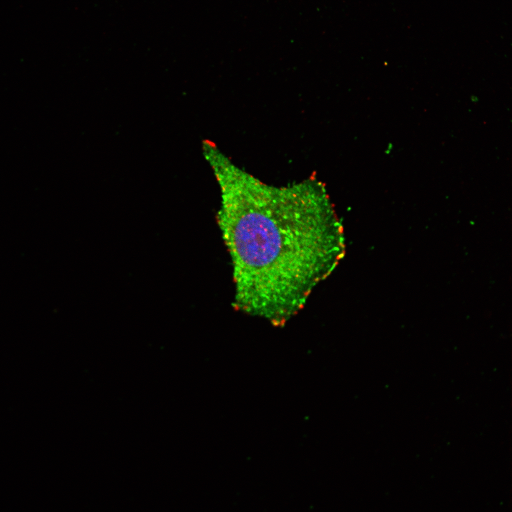

Supplement: Supplementary file 8 — Source Data for Figure 4 [file EMBR-24-e55069-s009.zip › 4A/451LU/451Lu_FAK-V2_11/Composite (RGB)-11.tif]

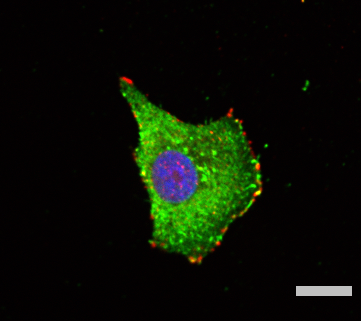

Supplement: Supplementary file 8 — Source Data for Figure 4 [file EMBR-24-e55069-s009.zip › 4A/451LU/451Lu_FAK-V2_11/Composite (RGB)-11_scaled.tif]

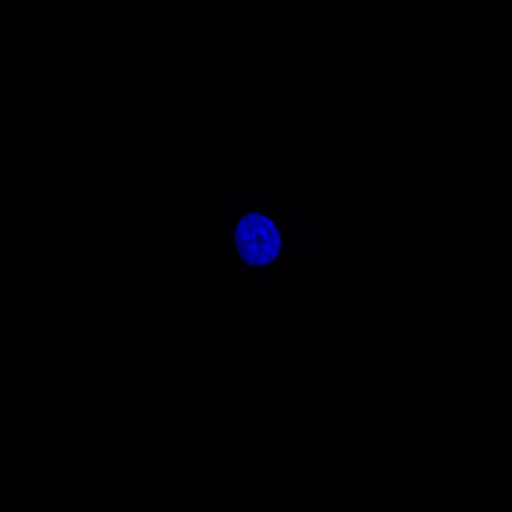

Supplement: Supplementary file 8 — Source Data for Figure 4 [file EMBR-24-e55069-s009.zip › 4A/451LU/451Lu_FAK-V2_11/Dapi-11.tif]

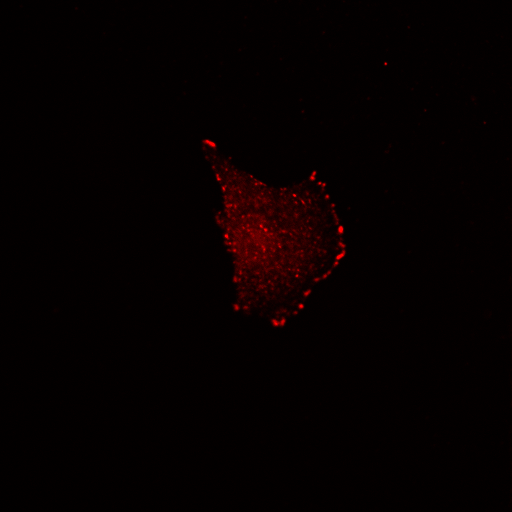

Supplement: Supplementary file 8 — Source Data for Figure 4 [file EMBR-24-e55069-s009.zip › 4A/451LU/451Lu_FAK-V2_11/P-FAKy397.tif]

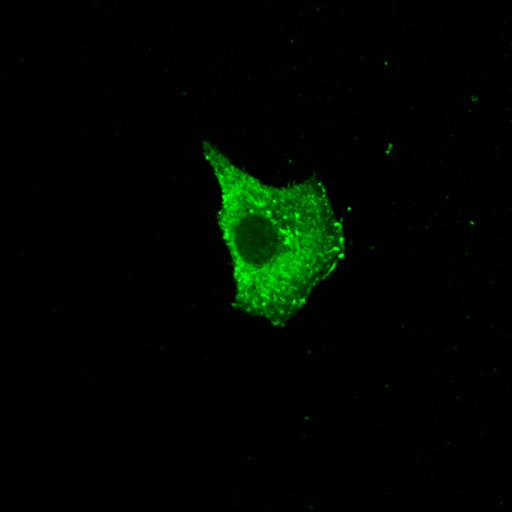

Supplement: Supplementary file 8 — Source Data for Figure 4 [file EMBR-24-e55069-s009.zip › 4A/451LU/451Lu_FAK-V2_11/TRPV2-11.tif]

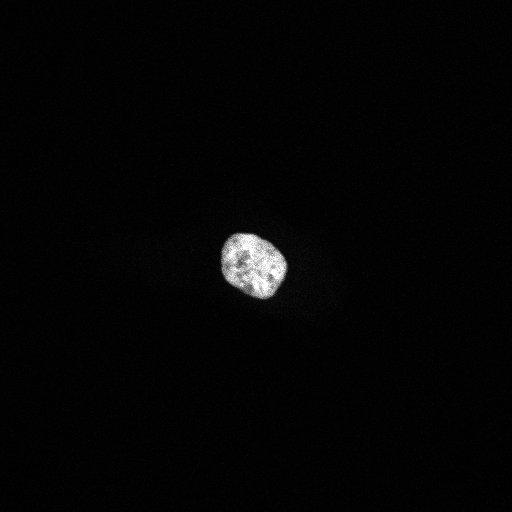

Supplement: Supplementary file 8 — Source Data for Figure 4 [file EMBR-24-e55069-s009.zip › 4A/451LU/451Lu_PAX-V2_17/17.tif]

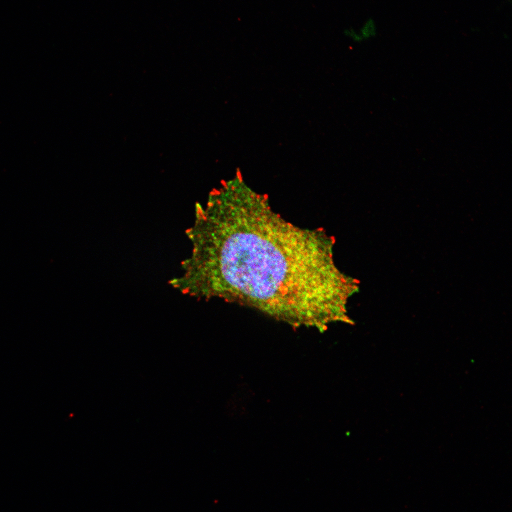

Supplement: Supplementary file 8 — Source Data for Figure 4 [file EMBR-24-e55069-s009.zip › 4A/451LU/451Lu_PAX-V2_17/Composite (RGB)-17.tif]

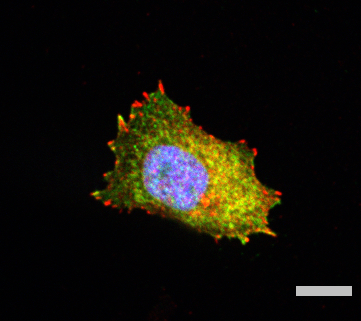

Supplement: Supplementary file 8 — Source Data for Figure 4 [file EMBR-24-e55069-s009.zip › 4A/451LU/451Lu_PAX-V2_17/Composite (RGB)-17_scaled.tif]

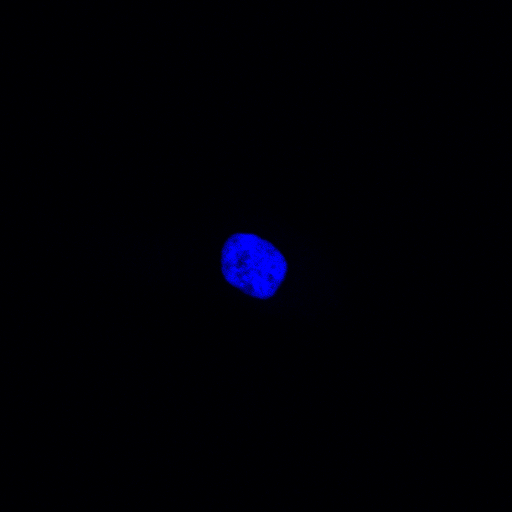

Supplement: Supplementary file 8 — Source Data for Figure 4 [file EMBR-24-e55069-s009.zip › 4A/451LU/451Lu_PAX-V2_17/Dapi-17.tif]

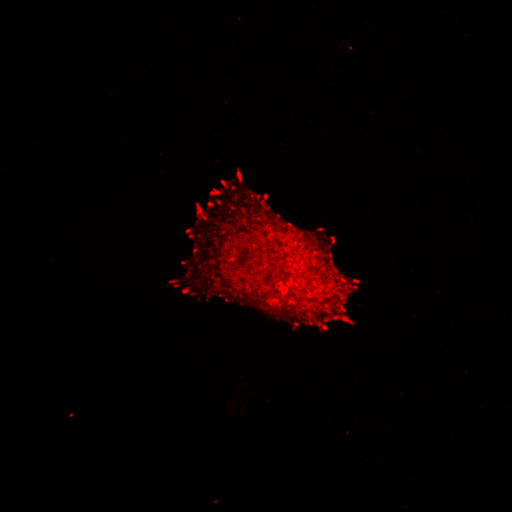

Supplement: Supplementary file 8 — Source Data for Figure 4 [file EMBR-24-e55069-s009.zip › 4A/451LU/451Lu_PAX-V2_17/Paxillin-17.tif]

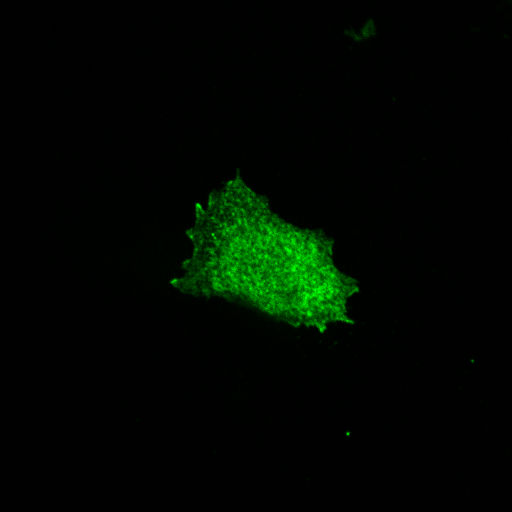

Supplement: Supplementary file 8 — Source Data for Figure 4 [file EMBR-24-e55069-s009.zip › 4A/451LU/451Lu_PAX-V2_17/TRPV2-17.tif]

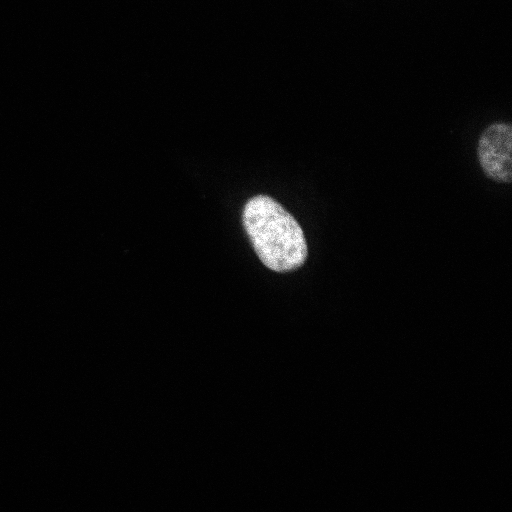

Supplement: Supplementary file 8 — Source Data for Figure 4 [file EMBR-24-e55069-s009.zip › 4A/451LU/451Lu_VCL-V2_4/4.tif]

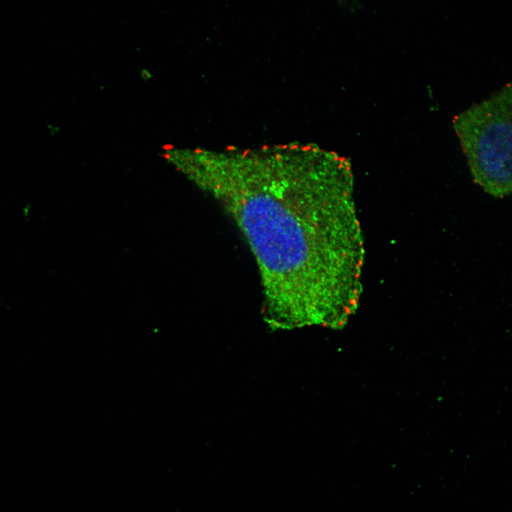

Supplement: Supplementary file 8 — Source Data for Figure 4 [file EMBR-24-e55069-s009.zip › 4A/451LU/451Lu_VCL-V2_4/Composite (RGB)-4.tif]

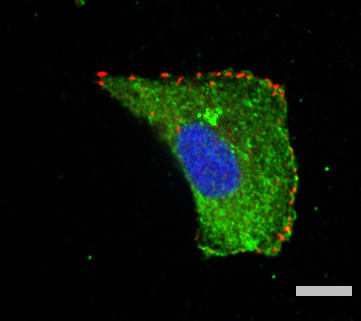

Supplement: Supplementary file 8 — Source Data for Figure 4 [file EMBR-24-e55069-s009.zip › 4A/451LU/451Lu_VCL-V2_4/Composite (RGB)-4_scaled.tif]

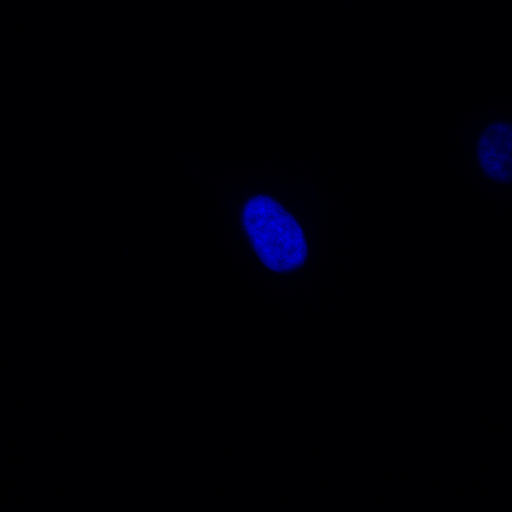

Supplement: Supplementary file 8 — Source Data for Figure 4 [file EMBR-24-e55069-s009.zip › 4A/451LU/451Lu_VCL-V2_4/Dapi-4.tif]

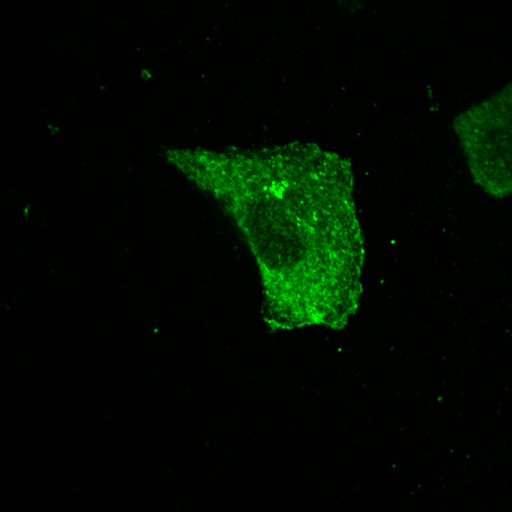

Supplement: Supplementary file 8 — Source Data for Figure 4 [file EMBR-24-e55069-s009.zip › 4A/451LU/451Lu_VCL-V2_4/TRPV2-4.tif]

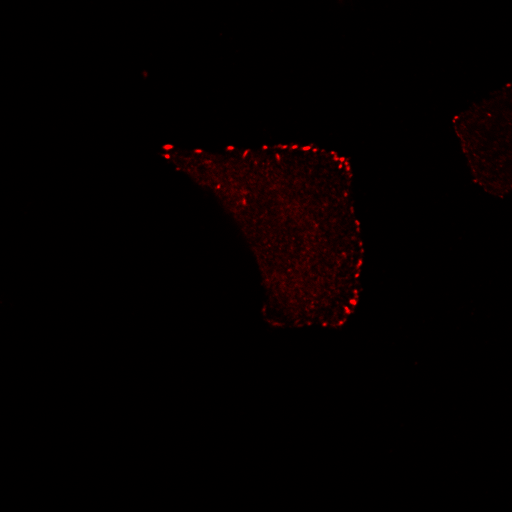

Supplement: Supplementary file 8 — Source Data for Figure 4 [file EMBR-24-e55069-s009.zip › 4A/451LU/451Lu_VCL-V2_4/Vinculin-4.tif]

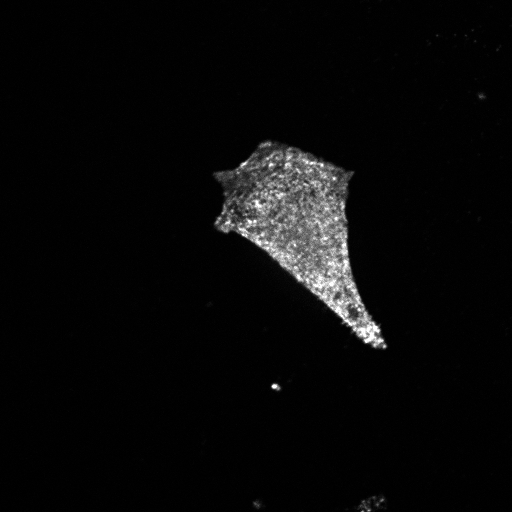

Supplement: Supplementary file 8 — Source Data for Figure 4 [file EMBR-24-e55069-s009.zip › 4A/WM266_4/WM266_Act-V2_13/13.tif]

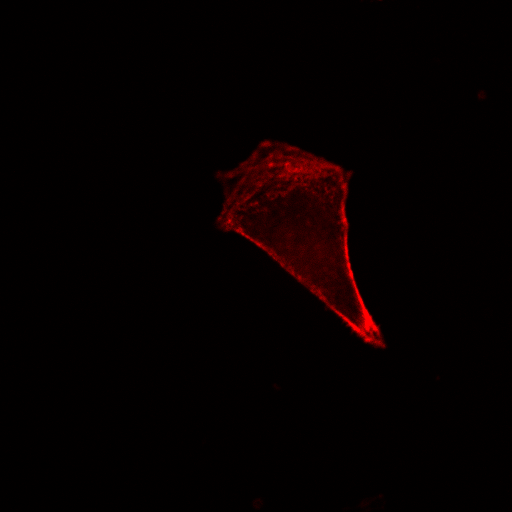

Supplement: Supplementary file 8 — Source Data for Figure 4 [file EMBR-24-e55069-s009.zip › 4A/WM266_4/WM266_Act-V2_13/Actin-13.tif]

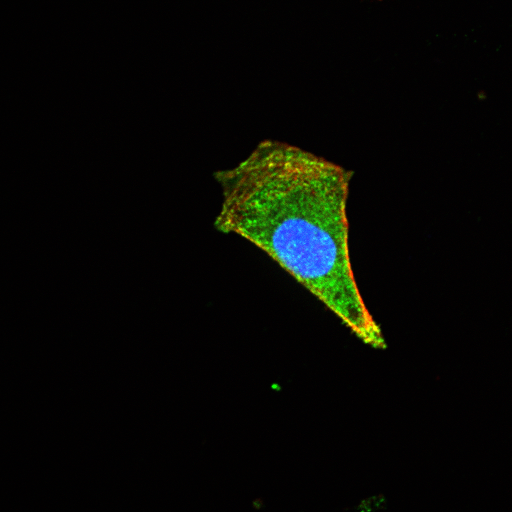

Supplement: Supplementary file 8 — Source Data for Figure 4 [file EMBR-24-e55069-s009.zip › 4A/WM266_4/WM266_Act-V2_13/Composite (RGB)-13.tif]

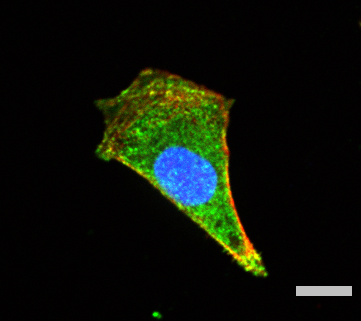

Supplement: Supplementary file 8 — Source Data for Figure 4 [file EMBR-24-e55069-s009.zip › 4A/WM266_4/WM266_Act-V2_13/Composite (RGB)-13_scaled.tif]

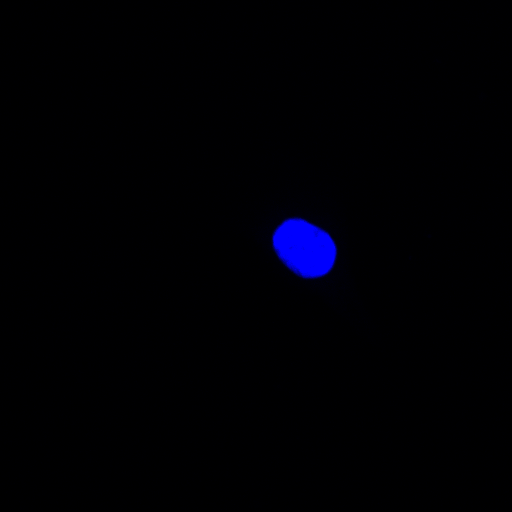

Supplement: Supplementary file 8 — Source Data for Figure 4 [file EMBR-24-e55069-s009.zip › 4A/WM266_4/WM266_Act-V2_13/Dapi-13.tif]

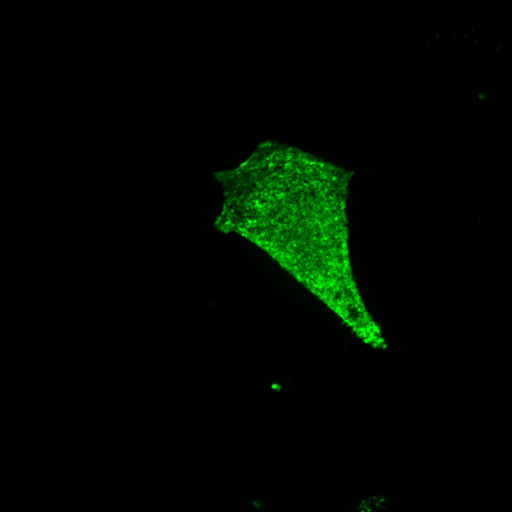

Supplement: Supplementary file 8 — Source Data for Figure 4 [file EMBR-24-e55069-s009.zip › 4A/WM266_4/WM266_Act-V2_13/TRPV2-13.tif]

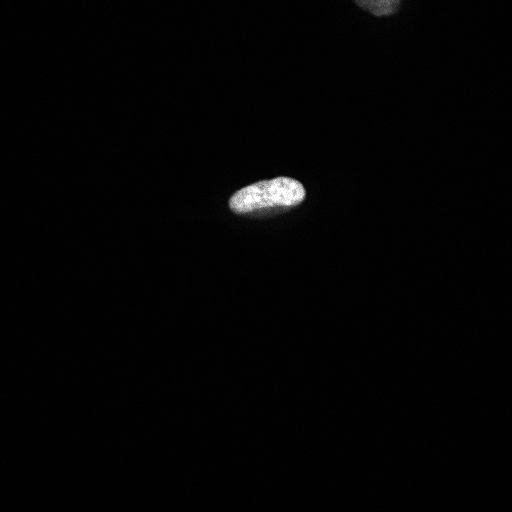

Supplement: Supplementary file 8 — Source Data for Figure 4 [file EMBR-24-e55069-s009.zip › 4A/WM266_4/WM266_FAK-V2_17/17.tif]

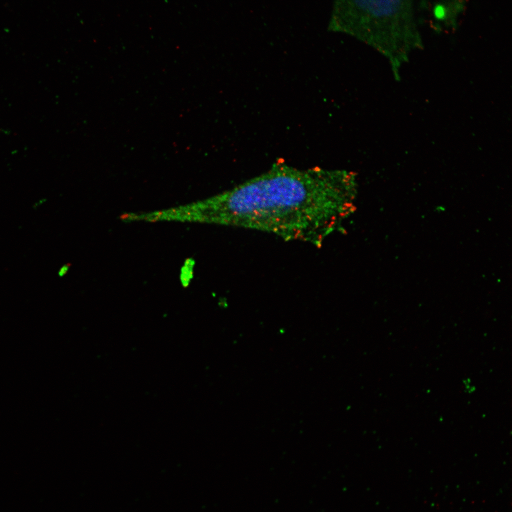

Supplement: Supplementary file 8 — Source Data for Figure 4 [file EMBR-24-e55069-s009.zip › 4A/WM266_4/WM266_FAK-V2_17/Composite (RGB)-17.tif]

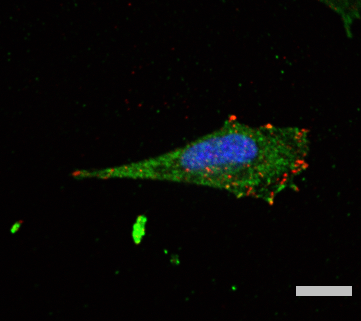

Supplement: Supplementary file 8 — Source Data for Figure 4 [file EMBR-24-e55069-s009.zip › 4A/WM266_4/WM266_FAK-V2_17/Composite (RGB)-17_scaled.tif]

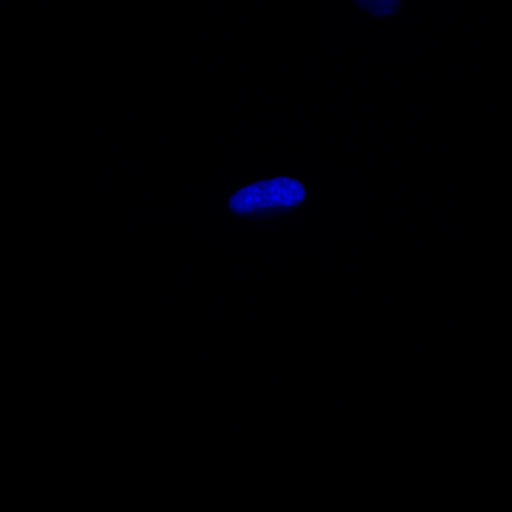

Supplement: Supplementary file 8 — Source Data for Figure 4 [file EMBR-24-e55069-s009.zip › 4A/WM266_4/WM266_FAK-V2_17/Dapi-17.tif]

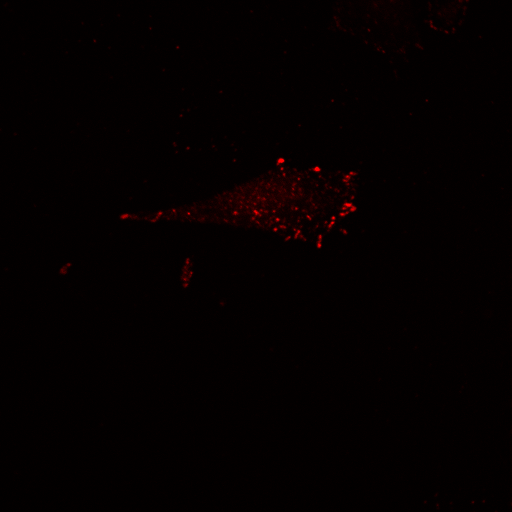

Supplement: Supplementary file 8 — Source Data for Figure 4 [file EMBR-24-e55069-s009.zip › 4A/WM266_4/WM266_FAK-V2_17/P-FAK-17.tif]

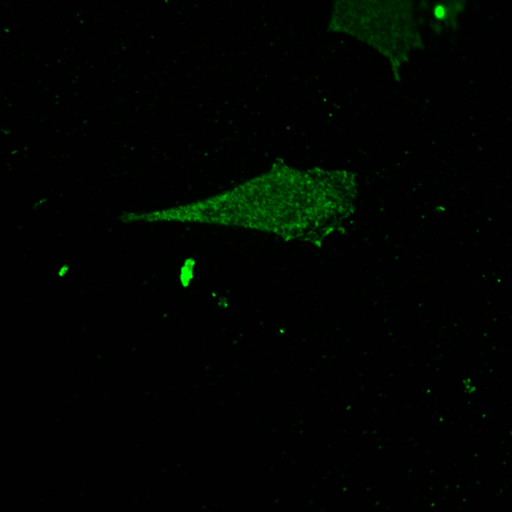

Supplement: Supplementary file 8 — Source Data for Figure 4 [file EMBR-24-e55069-s009.zip › 4A/WM266_4/WM266_FAK-V2_17/TRPV2-17.tif]

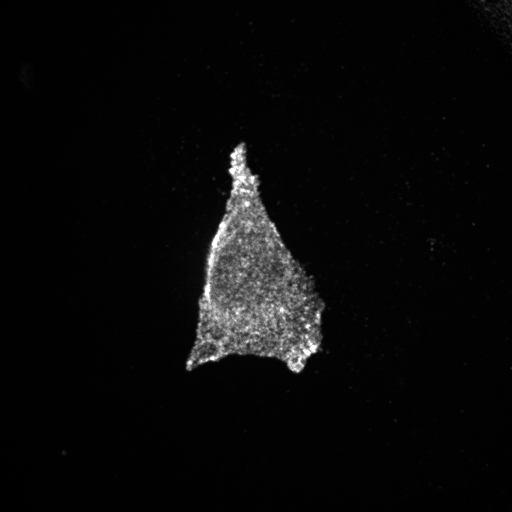

Supplement: Supplementary file 8 — Source Data for Figure 4 [file EMBR-24-e55069-s009.zip › 4A/WM266_4/WM266_PAX-V2_26/26.tif]

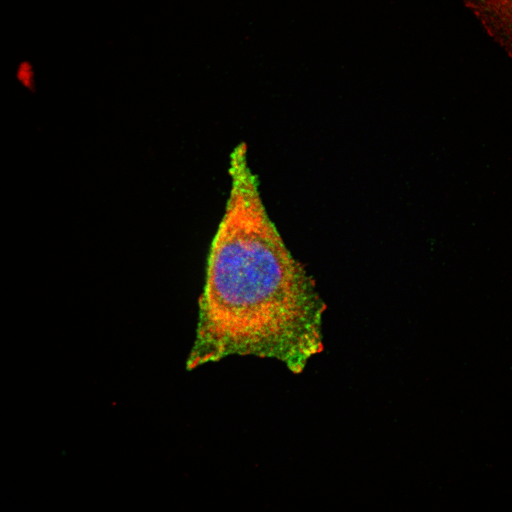

Supplement: Supplementary file 8 — Source Data for Figure 4 [file EMBR-24-e55069-s009.zip › 4A/WM266_4/WM266_PAX-V2_26/Composite (RGB)-26.tif]

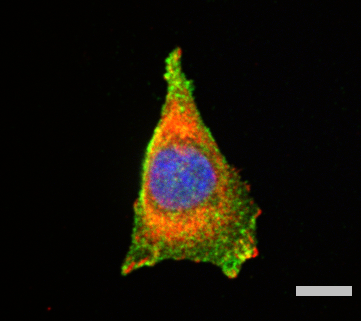

Supplement: Supplementary file 8 — Source Data for Figure 4 [file EMBR-24-e55069-s009.zip › 4A/WM266_4/WM266_PAX-V2_26/Composite (RGB)-26_scaled.tif]

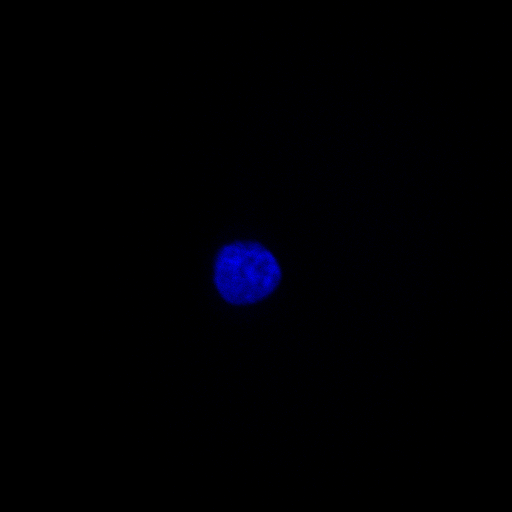

Supplement: Supplementary file 8 — Source Data for Figure 4 [file EMBR-24-e55069-s009.zip › 4A/WM266_4/WM266_PAX-V2_26/Dapi-26.tif]

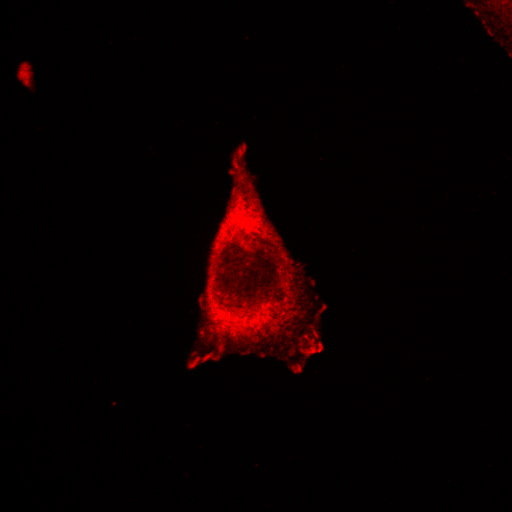

Supplement: Supplementary file 8 — Source Data for Figure 4 [file EMBR-24-e55069-s009.zip › 4A/WM266_4/WM266_PAX-V2_26/Paxillin-26.tif]

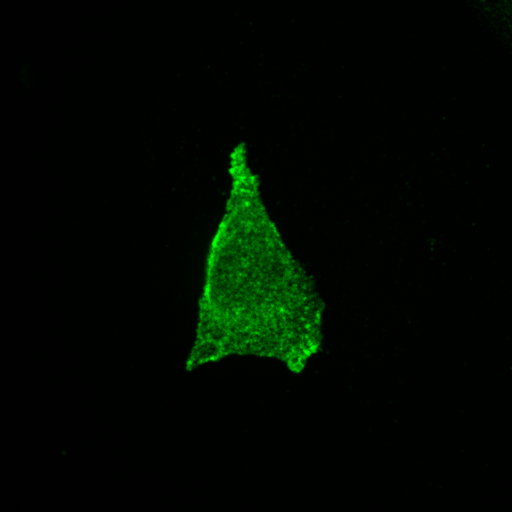

Supplement: Supplementary file 8 — Source Data for Figure 4 [file EMBR-24-e55069-s009.zip › 4A/WM266_4/WM266_PAX-V2_26/TRPV2-26.tif]

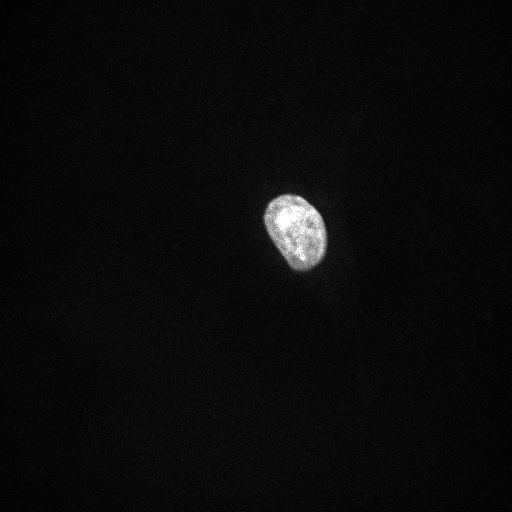

Supplement: Supplementary file 8 — Source Data for Figure 4 [file EMBR-24-e55069-s009.zip › 4A/WM266_4/WM266_VCL-V2_5/5.tif]

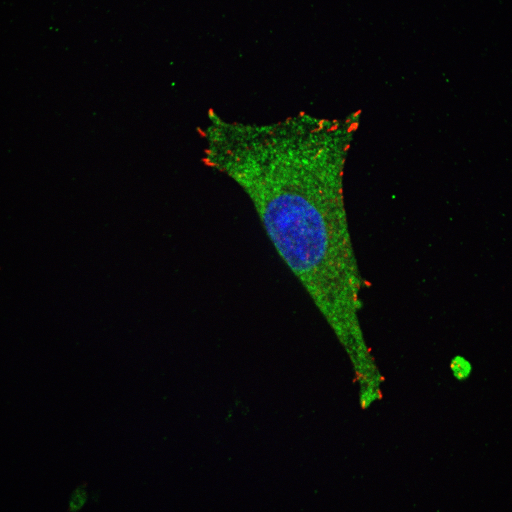

Supplement: Supplementary file 8 — Source Data for Figure 4 [file EMBR-24-e55069-s009.zip › 4A/WM266_4/WM266_VCL-V2_5/Composite (RGB)-5.tif]

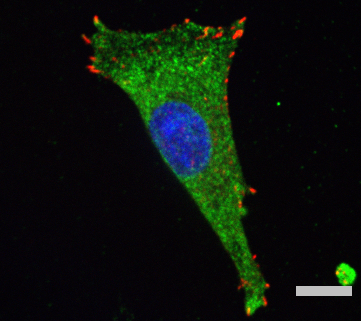

Supplement: Supplementary file 8 — Source Data for Figure 4 [file EMBR-24-e55069-s009.zip › 4A/WM266_4/WM266_VCL-V2_5/Composite (RGB)-5_scaled.tif]

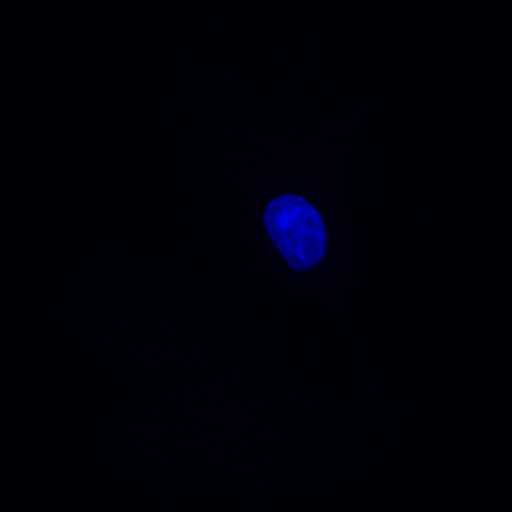

Supplement: Supplementary file 8 — Source Data for Figure 4 [file EMBR-24-e55069-s009.zip › 4A/WM266_4/WM266_VCL-V2_5/Dapi-5.tif]

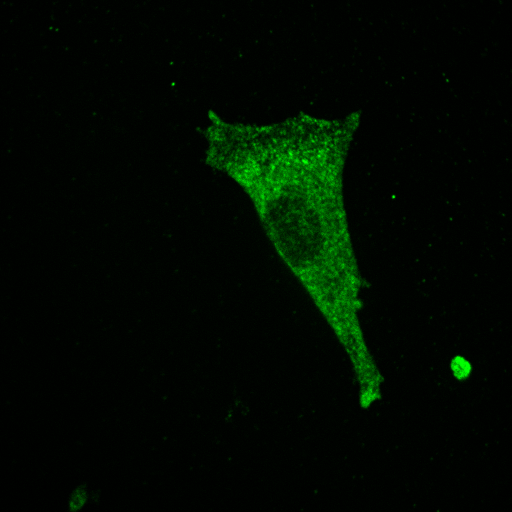

Supplement: Supplementary file 8 — Source Data for Figure 4 [file EMBR-24-e55069-s009.zip › 4A/WM266_4/WM266_VCL-V2_5/TRPV2-5.tif]

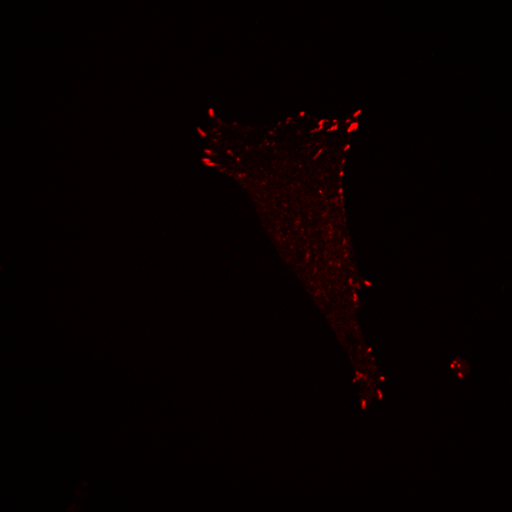

Supplement: Supplementary file 8 — Source Data for Figure 4 [file EMBR-24-e55069-s009.zip › 4A/WM266_4/WM266_VCL-V2_5/Vinculin-5.tif]

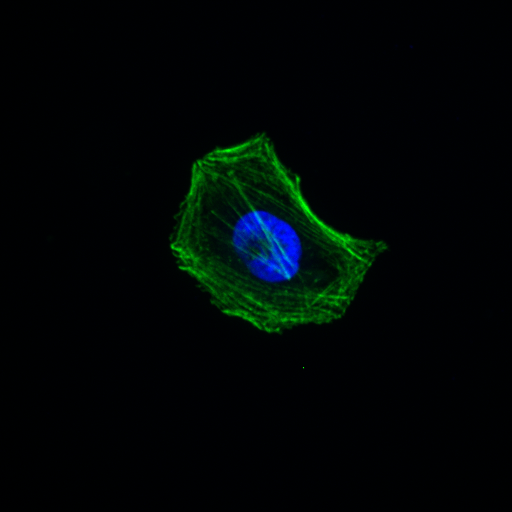

Supplement: Supplementary file 8 — Source Data for Figure 4 [file EMBR-24-e55069-s009.zip › 4B/EMBOR-2022-55069V1_SourceDataForFigure4B_451LuIg_V2.tif]

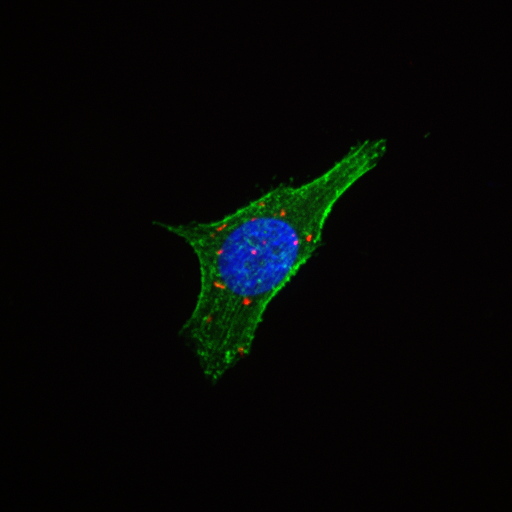

Supplement: Supplementary file 8 — Source Data for Figure 4 [file EMBR-24-e55069-s009.zip › 4B/EMBOR-2022-55069V1_SourceDataForFigure4B_451LuPAX_V2.tif]

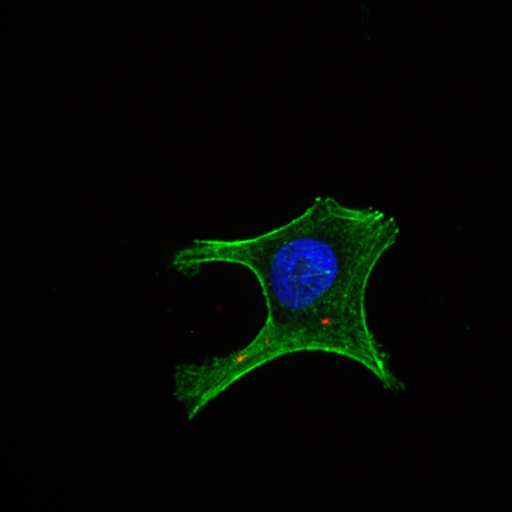

Supplement: Supplementary file 8 — Source Data for Figure 4 [file EMBR-24-e55069-s009.zip › 4B/EMBOR-2022-55069V1_SourceDataForFigure4B_451LuVCL_V2.tif]

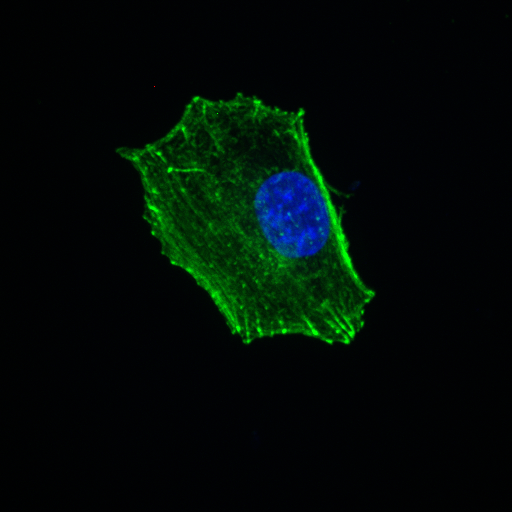

Supplement: Supplementary file 8 — Source Data for Figure 4 [file EMBR-24-e55069-s009.zip › 4B/EMBOR-2022-55069V1_SourceDataForFigure4B_WM266Ig_V2.tif]

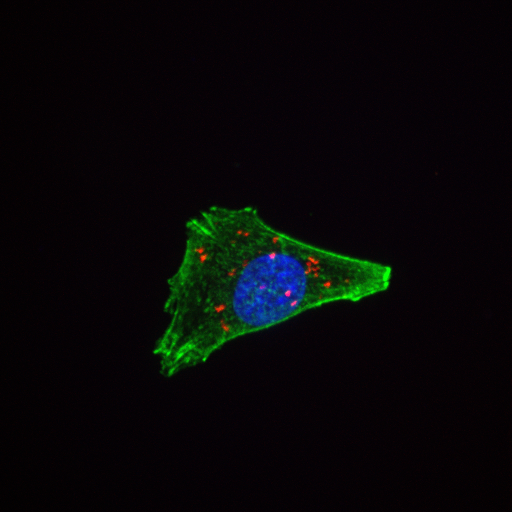

Supplement: Supplementary file 8 — Source Data for Figure 4 [file EMBR-24-e55069-s009.zip › 4B/EMBOR-2022-55069V1_SourceDataForFigure4B_WM266PAX_V2.tif]

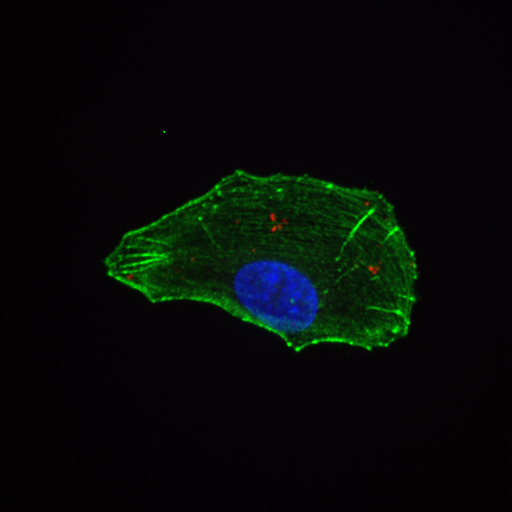

Supplement: Supplementary file 8 — Source Data for Figure 4 [file EMBR-24-e55069-s009.zip › 4B/EMBOR-2022-55069V1_SourceDataForFigure4B_WM266VCL_V2.tif]

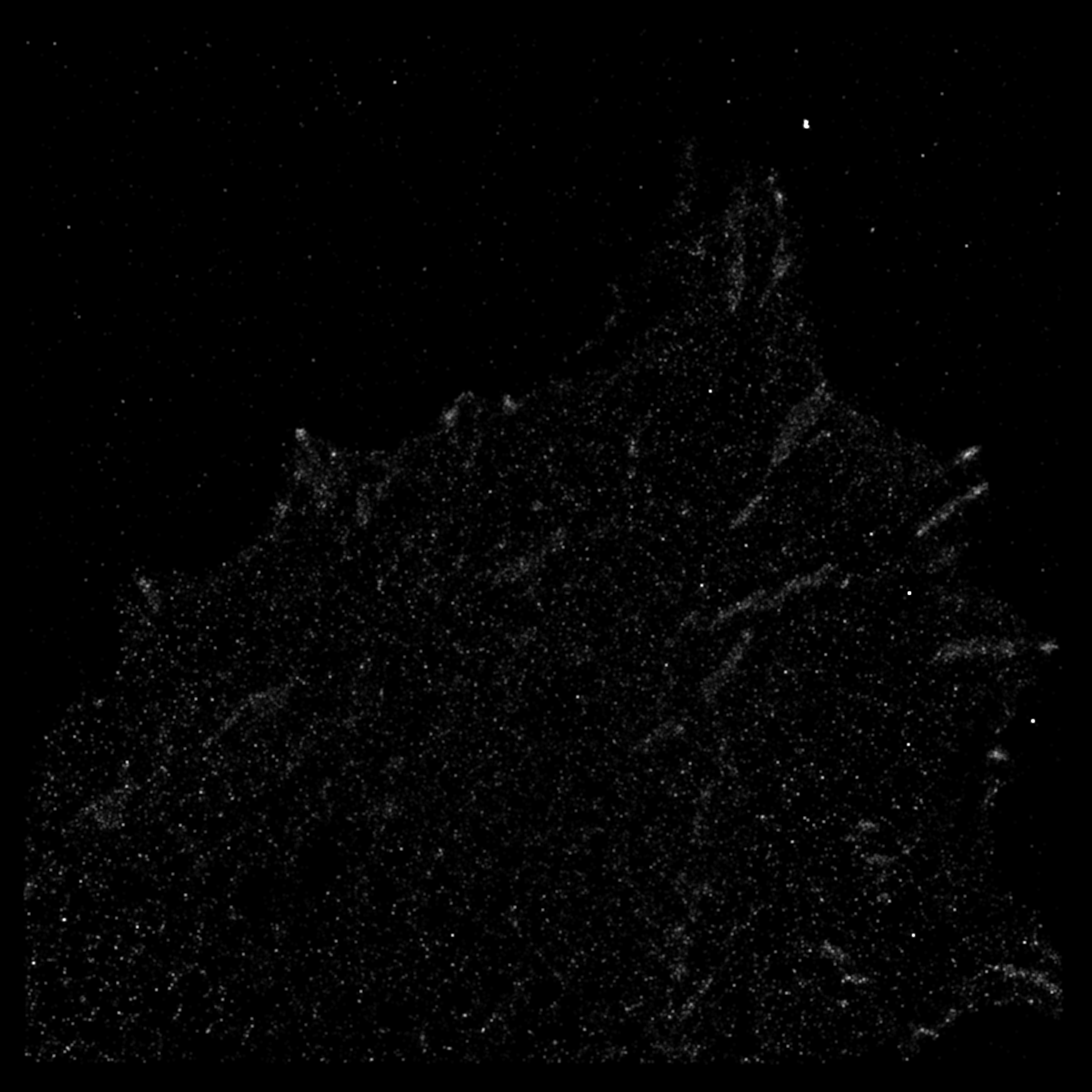

Supplement: Supplementary file 8 — Source Data for Figure 4 [file EMBR-24-e55069-s009.zip › 4C/EMBOR-2022-55069V1_SourceDataForFigure4C_WM266_PAXTRPV2_15nm - PAXAlexa 555.tif]

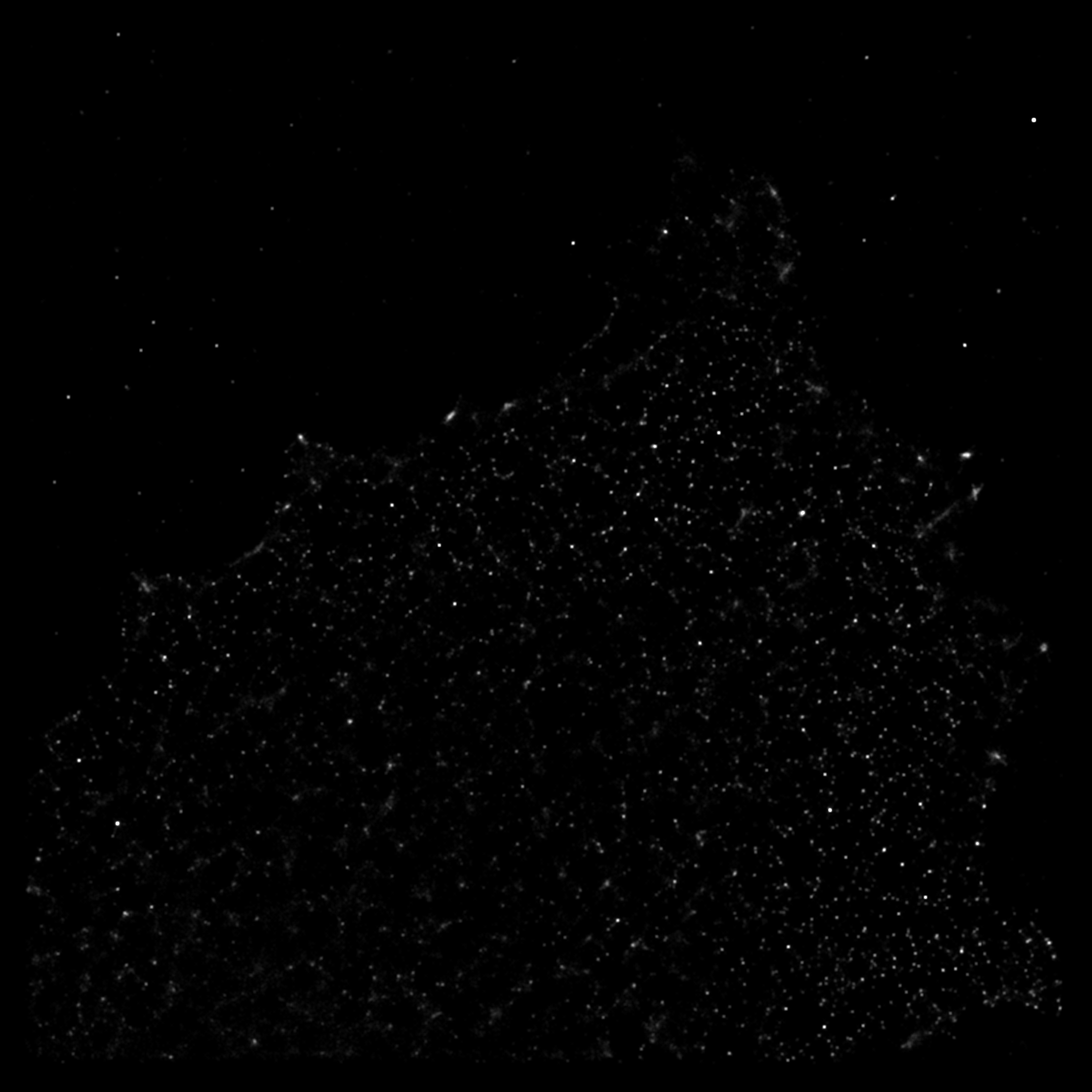

Supplement: Supplementary file 8 — Source Data for Figure 4 [file EMBR-24-e55069-s009.zip › 4C/EMBOR-2022-55069V1_SourceDataForFigure4C_WM266_PAXTRPV2_15nm - TRPV2Alexa 647.tif]

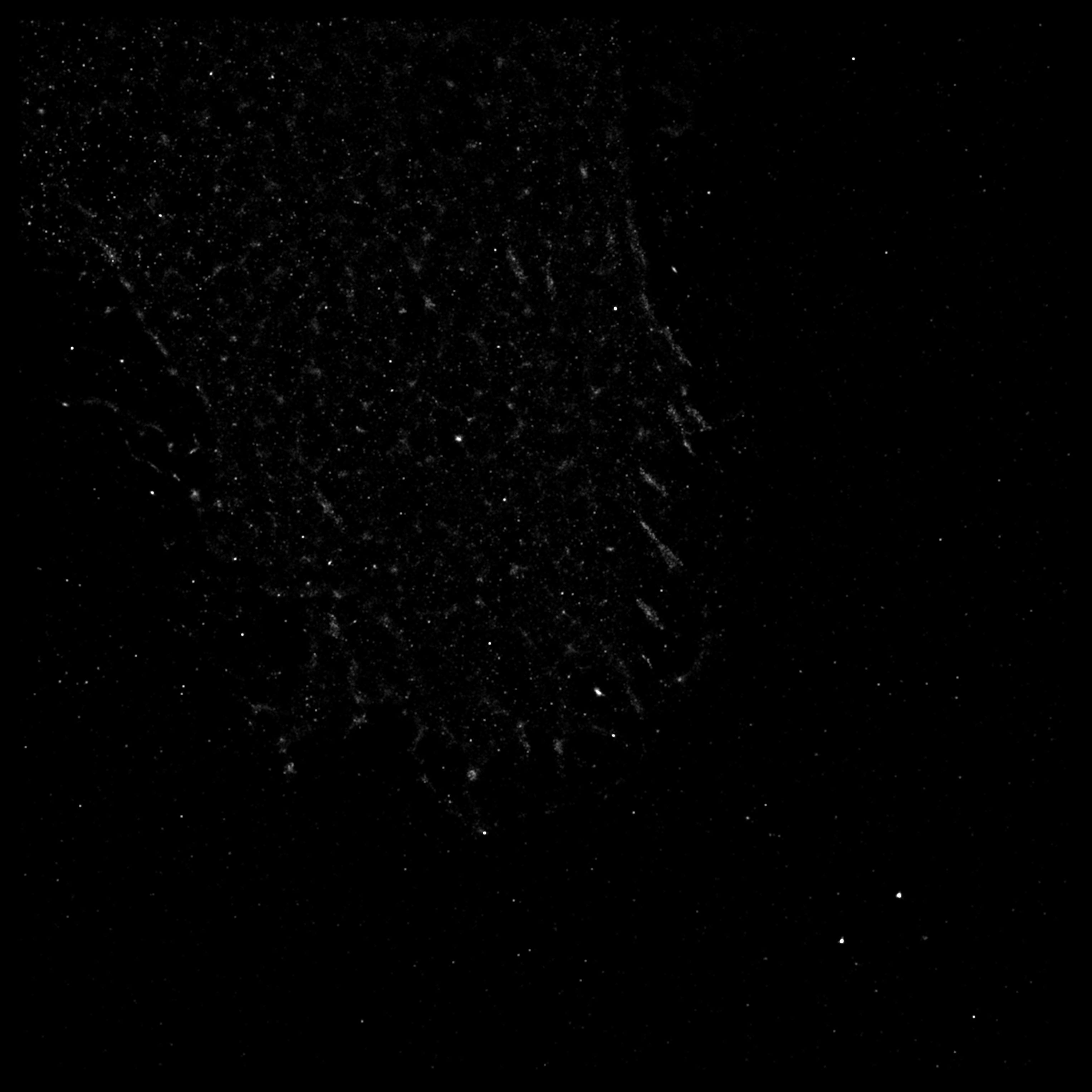

Supplement: Supplementary file 8 — Source Data for Figure 4 [file EMBR-24-e55069-s009.zip › 4C/EMBOR-2022-55069V1_SourceDataForFigure4C_WM266_VCL-TRPV2_15nm - VCLAlexa 555.tif]

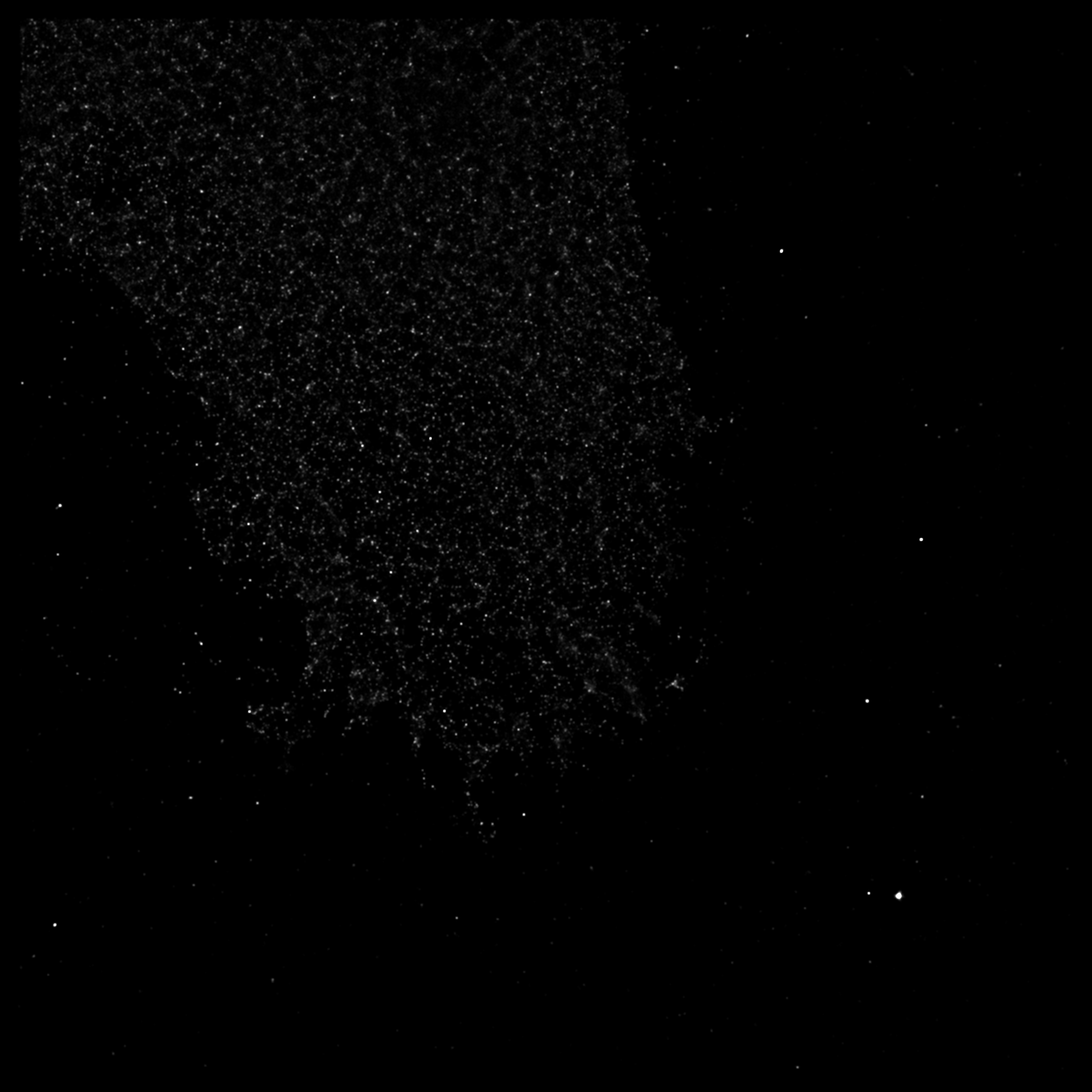

Supplement: Supplementary file 8 — Source Data for Figure 4 [file EMBR-24-e55069-s009.zip › 4C/EMBOR-2022-55069V1_SourceDataForFigure4C_WM266_VCLTRPV2_15nm - TRPV2Alexa 647.tif]

## Slide 1
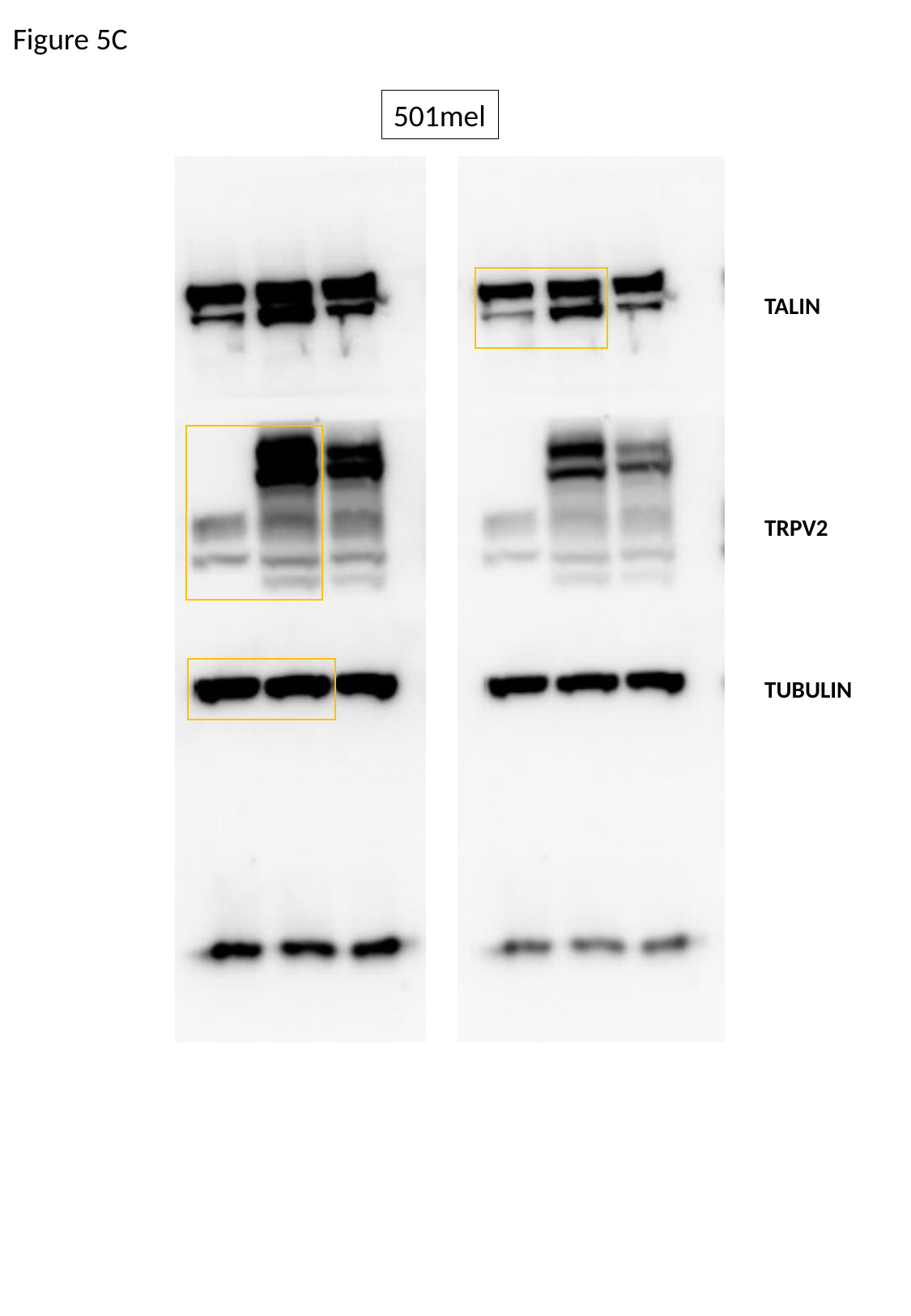

Figure 5C
501mel
TALIN
TRPV2
TUBULIN

## Slide 2
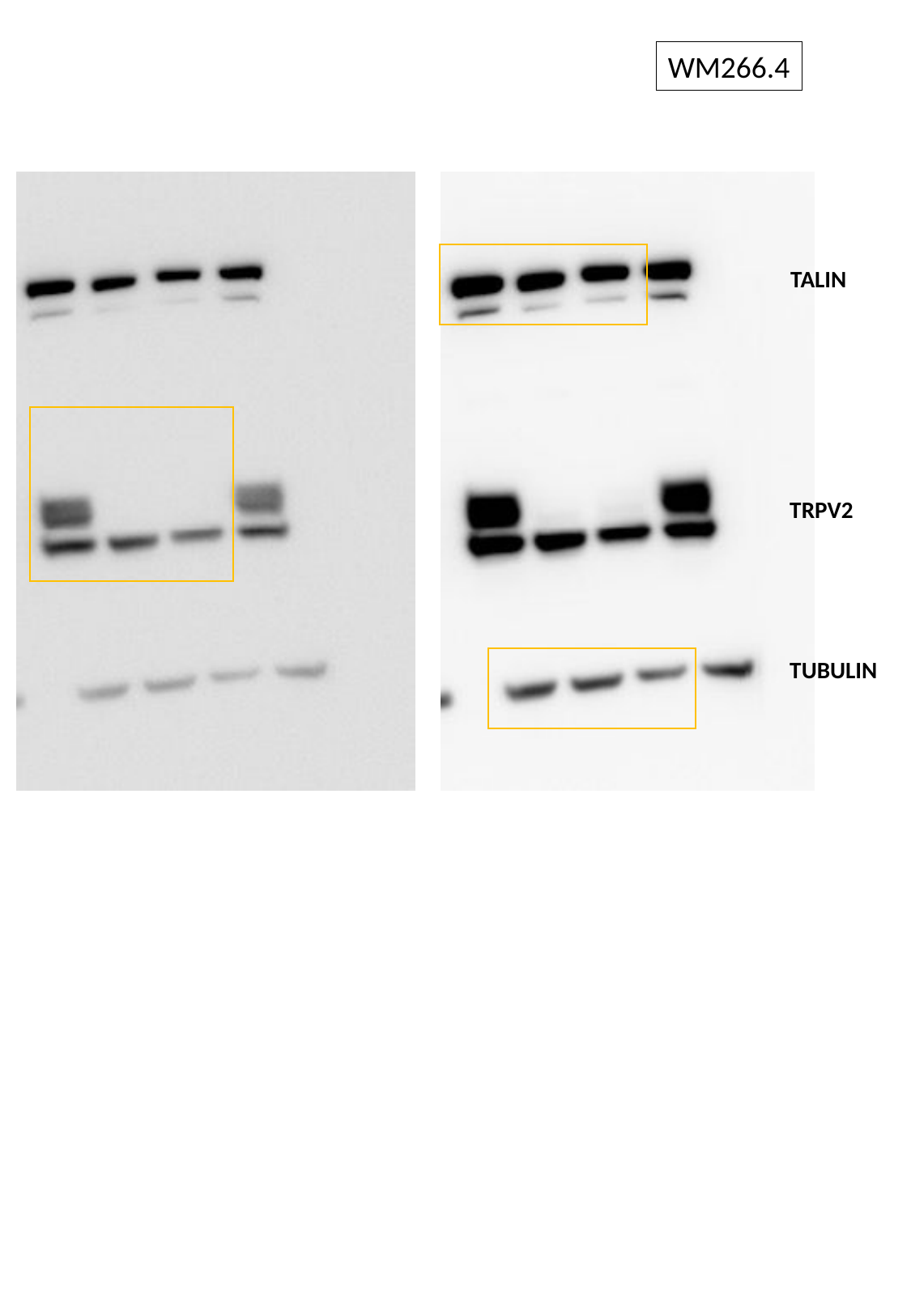

WM266.4
TALIN
TRPV2
TUBULIN

## Slide 3
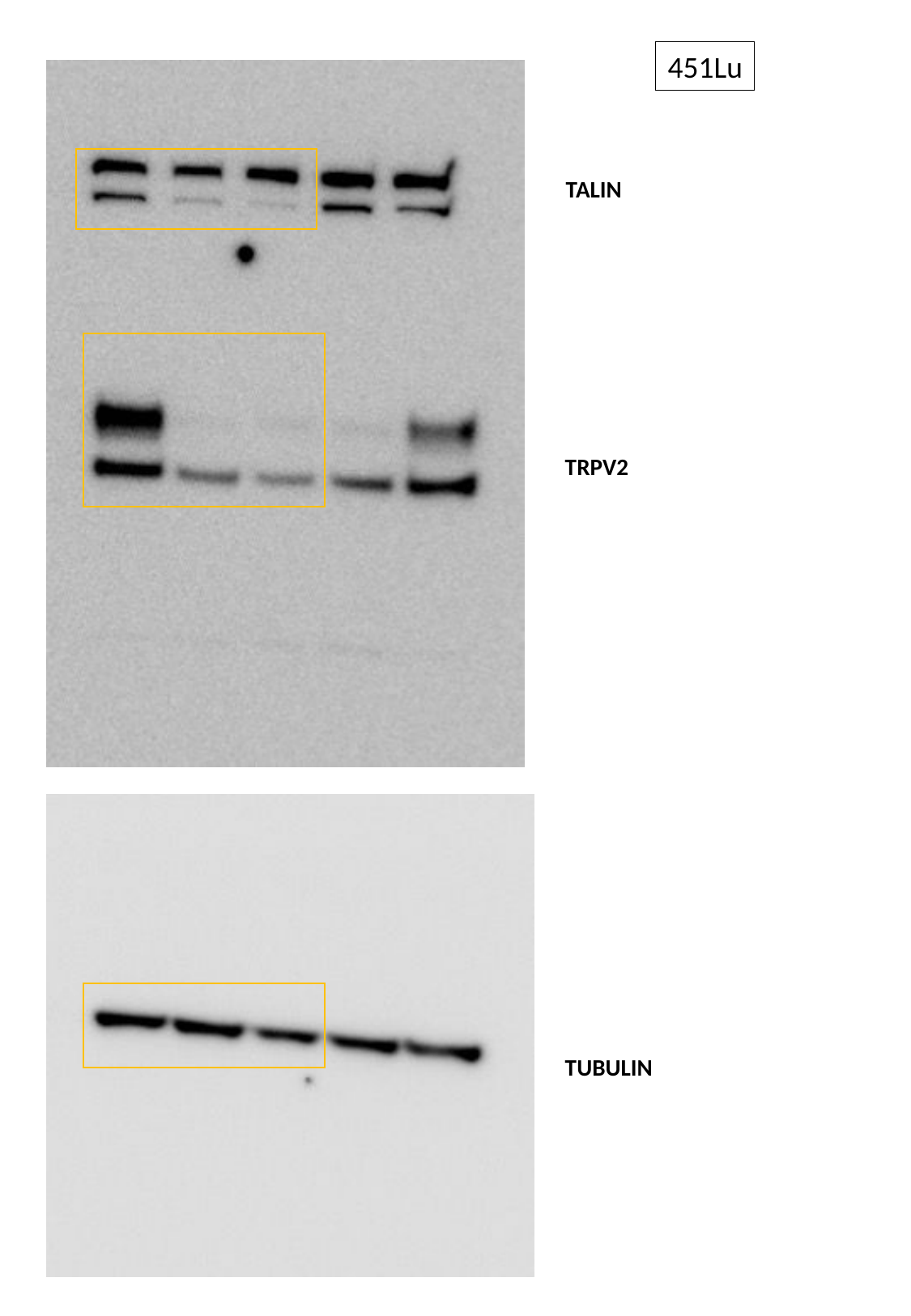

451Lu
TALIN
TRPV2
TUBULIN

Supplement: Supplementary file 9 — Source Data for Figure 5 [file EMBR-24-e55069-s004.zip › EMBOR-2022-55069V1_SourceDataForFigure5C.pptx]
