# Supplementary material for: The mechanosensitive TRPV2 calcium channel promotes human melanoma invasiveness and metastatic potential
Source: EMBO Rep. 2023 Feb 6;24(4):e55069. doi: 10.15252/embr.202255069 (PMC10074106; doi:10.15252/embr.202255069)
Supplement: Supplementary file 4 — PDF+ [file EMBR-24-e55069-s007.pdf]

Article

SOURCE DATA · TRANSPARENT PROCESS · OPEN ACCESS

EMBO reports

The mechanosensitive TRPV2 calcium channel promotes human melanoma invasiveness and metastatic potential

Kenji F Shoji[1], Elsa Bayet[1,2], Sabrina Leverrier-Penna[2], Dahiana Le Devedec[1], Aude Mallavialle[3], Séverine Marionneau-Lambot[4], Florian Rambow[5,6,7], Raul Perret[8], Aurélie Joussaume[1], Roselyne Viel[9], Alain Fautrel[9], Amir Khammari[8], Bruno Constantin[2], Sophie Tartare-Deckert[3] & Aubin Penna[1,2,*]

Abstract

Melanoma is a highly aggressive cancer endowed with a unique capacity of rapidly metastasizing, which is fundamentally driven by aberrant cell motility behaviors. Discovering "migrastatics" targets, specifically controlling invasion and dissemination of melanoma cells during metastasis, is therefore of primary importance. Here, we uncover the prominent expression of the plasma membrane TRPV2 calcium channel as a distinctive feature of melanoma tumors, directly related to melanoma metastatic dissemination. *In vitro* as well as *in vivo*, TRPV2 activity is sufficient to confer both migratory and invasive potentials, while conversely TRPV2 silencing in highly metastatic melanoma cells prevents aggressive behavior. In invasive melanoma cells, TRPV2 channel localizes at the leading edge, in dynamic nascent adhesions, and regulates calcium-mediated activation of calpain and the ensuing cleavage of the adhesive protein talin, along with F-actin organization. In human melanoma tissues, TRPV2 overexpression correlates with advanced malignancy and poor prognosis, evoking a biomarker potential. Hence, by regulating adhesion and motility, the mechanosensitive TRPV2 channel controls melanoma cell invasiveness, highlighting a new therapeutic option for migrastatics in the treatment of metastatic melanoma.

Keywords calpain; melanoma; metastasis; migration; TRPV2 channel
Subject Categories Cancer; Cell Adhesion, Polarity & Cytoskeleton; Membrane & Trafficking
DOI 10.15252/embr.202255069 | Received 17 March 2022 | Revised 21 December 2022 | Accepted 10 January 2023 | Published online 6 February 2023
EMBO Reports (2023) 24: e55069

Introduction

Cutaneous malignant melanoma (CMM) is a cancer arising from skin melanocytes (Kozar *et al*, 2019). *In situ* tumors can be cured by surgical resection, but melanoma has a distinct tendency to very rapidly spread into multiple organs. Metastatic melanoma is the deadliest form of skin cancer, with a rising incidence (Siegel *et al*, 2018). Over the last decade, immuno- and targeted therapies have shown increasing clinical benefit, but remain often insufficient to achieve durable responses (Sharma *et al*, 2017; Kozar *et al*, 2019), due to the heterogeneous and exceedingly plastic properties of melanoma. Hence, understanding the molecular mechanisms enabling the acquisition of the CMM unique metastatic behavior is critical for defining early biomarkers and novel therapeutic targets. The dynamic behavior of CMM dissemination is sustained by an increased cell motility and invasiveness, both requiring precise communication between cells and their environment to breach basement membranes and colonize surrounding tissues. Accumulating evidence has demonstrated that altered calcium (Ca^{2+}) signaling promotes tumor cell-specific phenotypic changes, supporting the metastatic spread (Tajada & Villalobos, 2020).

The transient receptor potential (TRP) Ca^{2+} channel families have been identified as key actors in cancer cell migration and invasion (Leverrier-Penna *et al*, 2020; Karki & Tojkander, 2021). These cationic channels are emerging as very attractive therapeutic targets in oncology due to both their ability to switch on or off specific phenotypic hallmarks of tumor cells, and their accessibility to pharmacological modulators (Bruce & James, 2020). Among the 28

1 Inserm, EHESP, IRSET, UMR_S 1085, Université de Rennes 1, Rennes, France
2 CNRS, 4CS, Université de Poitiers, Poitiers, France
3 INSERM, C3M, team 'labellisée Ligue Contre le Cancer 2022, Université Côte d'Azur, Nice, France
4 Cancéropôle Grand Ouest, In Vivo Platform, Nantes, France
5 Department of Applied Computational Cancer Research, Institute for AI in Medicine (IKIM), University Hospital Essen, Essen, Germany
6 University of Duisburg-Essen, Essen, Germany
7 German Cancer Consortium (DKTK), Partner Site Essen, Essen, Germany
8 Service de Dermatologie, CHU Nantes, CIC 1413, INSERM, Immunology and New Concepts in ImmunoTherapy, INCIT, UMR 1302, Nantes Université, Nantes, France
9 CNRS, Inserm UMS Biosit, H2P2 Core Facility, Université de Rennes 1, Rennes, France
*Corresponding author. Tel: +(33) 549 453 996; E-mail: aubin.penna@cnrs.fr

© 2023 The Authors. Published under the terms of the CC BY 4.0 license.

human members of this channel superfamily, 4 subfamilies out of 6, TRPCanonical, TRPVanilloid, TRPMelastatin, and TRPAnkyrin, have been involved in tumor invasiveness. Unlike the shared structural similarities, the ionic signature of each polymodal member is unique, and corresponds to various heterogeneous stimuli, including chemical and physical stimuli such as mechanical force. Some members, including TRPC1, -4, -5, -6, TRPV2, -4, -6 and TRPM4, -7, -8 are functionally related to cellular events, including phenotypic transition, or structures, such as actin cytoskeleton and focal adhesions, that are essential for mechanotransduction in cell migration, consequently contributing to metastasis (Canales *et al*, 2019). Meanwhile the role for TRP Ca^{2+}-permeable channels signaling in the specific context of CMM progression is poorly documented. Among the TRP channels involved in melanoma, TRPM1 was described as important in melanocyte function and malignant melanoma pathophysiology, knowing that its expression correlates positively with the differentiation status of melanocytes and inversely with the aggressiveness and tumor thickness of malignant melanoma (Oancea *et al*, 2009). As for the stretch- and swelling-activated TRPM7 channel, despite a strong connection between its overexpression, metastasis and a poor prognosis in numerous cancer types (Guilbert *et al*, 2009, 2013; Middelbeek *et al*, 2012; Chen *et al*, 2015; Yee *et al*, 2015; Gao *et al*, 2017; Rybarczyk *et al*, 2017), its expression has been shown to be steady overall in both melanocytes and melanoma cells, and did not correlate to melanoma cell lines' invasive potential (McNeill *et al*, 2007). Still, little is known on the contribution of specific Ca^{2+} channel subunits in the particular context of CMM progression (Arozarena *et al*, 2011b; Macia *et al*, 2015).

The scope of this study was to identify atypical profiles among the numerous Ca^{2+}-conducting channels expressed in melanoma cells, to determine an eventual association with tumor metastatic progression, and to elucidate the associated molecular mechanisms. Using 2D and 3D *in vitro* models, as well as *in vivo* models and human tissues, we have established the essential role of the mechanosensitive Ca^{2+} channel TRPV2 during the metastatic switch of melanoma cells, defining this channel as a promising biomarker and migrastatic target.

Results

TRPV2 is the predominantly expressed calcium channel in metastatic melanoma

To identify Ca^{2+}-conducting channel subunits supporting melanoma metastasis formation, we screened the cancer genome atlas (TCGA) of skin cutaneous melanoma (SKCM) tumors. TRPV2 transcript stood out as the most expressed among most members of the major Ca^{2+}-permeable channel families, where at least one subunit has previously been associated with accrued motility behavior (Fig 1A). More specifically, within the TRP family, TRPV2 displayed the highest differential expression between human nevi and melanoma samples (Fig 1B). Likewise, in a set of metastatic CMM cell lines, TRPV2 expression exceeded the expression of the other nonvoltage-gated Ca^{2+} channel levels (Fig 1C and D). By querying transcriptomic data from the NCI-60 panel and the Broad-Novartis cancer cell line encyclopedia (CCLE), we evidenced that the exacerbated expression of TRPV2 was distinctive from melanoma cell lines, as compared to other cancer-derived cell lines (Fig 1E and Appendix Fig S1A). Consistently, among a large panel of cancer cell lines originating from different tissues, TRPV2 channel proteins were preferentially detected in melanoma cells (Fig 1F). In tumors, TRPV2 transcripts were also highly expressed in SKCM as compared to 36 other cancer types (Appendix Fig S1B).

Molecularly, melanomas harbor somatic 'driver mutations' that are mutually exclusive: 50% present gain-of-function BRAF mutations, while another 25% exhibit NRAS mutations (Cancer Genome

Figure 1. TRPV2's strong and predominant expression is a hallmark of metastatic melanoma.

A Analysis of Ca^{2+}-permeable channels mRNA expression levels (RNAseq data from the TCGA cohort) in skin cancer melanoma (SKCM) tumors (n(T) = 461). Transcript intensity is expressed as log2(TPM + 1) transformed expression data (Tang *et al*, 2017). The Ca^{2+}-permeable channels plotted on the *x*-axis are grouped by family: ATP-gated P2X; PIEZO; VGCC (voltage-gated Ca^{2+} channels); SOCC (store-operated Ca^{2+} channels); TRP (transient receptor potential).

B Heatmap comparing TRP channels RNA expression levels (mean of the normalized probe intensities) in nevi (n = 9) and melanomas (n = 104) biopsies (GEO: GSE46517; Data ref: Kwong *et al*, 2013). Probe set ID: TRPA1 217590_s_at; TRPC1 205802_at;TRPC3 206425_s_at; TRPC4 220817_at; TRPC6 206528_at; TRPC7 208589_at; TRPM1 206479_at; TRPM2 205708_s_at; TRPM3 211422_at; TRPM4 219360_s_at; TRPM6 221102_s_at; TRPM8 220226_at; TRPV1 219632_s_at; TRPV2 219282_s_at; TRPV4 219516_at; TRPV5 208267_at; TRPV6 206827_s_at; no probe for TRPM5/TRPM7/TRPV3 in the used [HG-U133A] Affymetrix Human Genome U133A Array. The red arrow points to TRPV2.

C Expression profile of the indicated ion channel analyzed by RT-PCR in the WM266.4 and 451Lu metastatic melanoma cell lines (middle and bottom panels, respectively). Expected amplicons sizes (positive controls in top panel) are in base pairs (bp): TRPV1 = 120, TRPV2 = 199, TRPV3 = 226, TRPV4 = 190/370, TRPV6 = 208, TRPC1 = 201, TRPC6 = 121, TRPM2 = 303, TRPM7 = 226, TRPA1 = 140, Orai1 = 161, STIM1 = 109, STIM2 = 114.

D Quantitative RT-PCR expression analyses of channel subunits consistently detected in both the WM266.4 and the 451Lu melanoma cell lines. Transcript levels were normalized to TATA box-binding protein (TBP) mRNA levels. Data are represented as mean ± SEM (n = 3 biological replicates). The red arrow points to TRPV2.

E Relative TRPV2 mRNA expression analysis in the NCI-60 cell lines panel. *y*-axis represents TRPV2 transcript intensity expressed in mean-centered *z*-score; bars either show increased or decreased expression relative to the mean expression. The cell lines plotted on the *x*-axis are grouped by tissue of origin. CNS: central nervous system. Data were generated by querying for TRPV2 as input in CellMiner™ (http://discover.nci.nih.gov/cellminer/; Reinhold *et al*, 2012; See also Appendix Fig S1).

F TRPV2 immunoblotting in 36 out of the 60 NCI-60 cell lines. Tubulin was used as a loading control. Note that after longer exposure times, TRPV2 expression was also detected in some breast and prostate cancer cell lines, as it has been previously described (Monet *et al*, 2010; Gambade *et al*, 2016; Elbaz *et al*, 2018).

G TRPV2 protein expression in melanoma cell lines harboring either B-RAF (black) or N-Ras (blue) mutations. β-Actin was used as a loading control.

H Quantitative RT-PCR analysis of both TRPV2 (black bars, left axis) and BRN2 (green bars, right axis) transcripts expression (normalized to TBP), in normal human epidermal melanocytes (NHEM), noninvasive 501mel, superficial spreading melanoma WM793, metastatic melanoma WM266.4 and 451Lu cell lines. BRN2 is a marker of the melanoma invasive phenotype (see also Appendix Fig S4 for active β-catenin levels representing an alternative invasive marker). Data are represented as mean ± SEM (n = 3 biological replicates).

Source data are available online for this figure.

 © 2023 The Authors

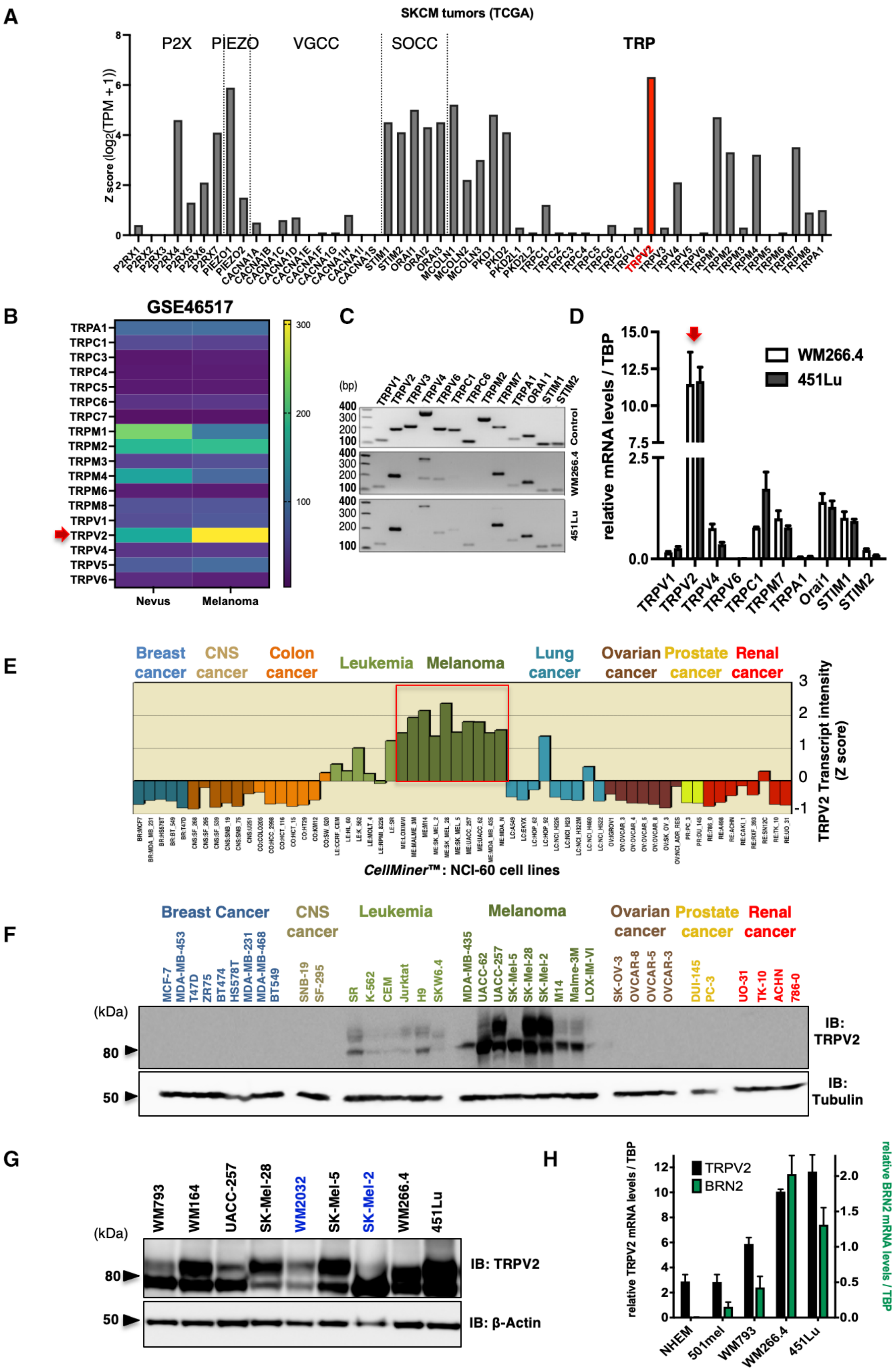
A
SKCM tumors (TCGA)
P2X
PIEZO
VGCC
SOCC
TRP
Z score (log2(TPM + 1))
B
GSE46517
Nevus
Melanoma
C
(bp)
Control
WM266.4
451Lu
D
relative mRNA levels / TBP
WM266.4
451Lu
E
Breast cancer
CNS cancer
Colon cancer
Leukemia
Melanoma
Lung cancer
Ovarian cancer
Prostate cancer
Renal cancer
TRPV2 Transcript intensity (Z score)
CellMiner™: NCI-60 cell lines
F
Breast Cancer
CNS cancer
Leukemia
Melanoma
Ovarian cancer
Prostate cancer
Renal cancer
(kDa)
IB: TRPV2
IB: Tubulin
G
WM793
WM164
UACC-257
SK-Mel-28
WM2032
SK-Mel-5
SK-Mel-2
WM266.4
451Lu
(kDa)
IB: TRPV2
IB: β-Actin
H
relative TRPV2 mRNA levels / TBP
relative BRN2 mRNA levels / TBP
TRPV2
BRN2
NHEM
501mel
WM793
WM266.4
451Lu

Figure 1.

Atlas Network, 2015). By analyzing TRPV2 protein expression in an extended panel of melanoma cell lines harboring either mutation, we consistently detected high amounts of TRPV2 regardless of the mutational status (Fig 1G and Appendix Fig S1C). Considering its predominant expression in melanoma, we investigated the functional relevance of this Ca^{2+} channel subunit in melanoma progression.

TRPV2 expression levels correlate with the invasive phenotype of melanoma tumor cell lines

Melanoma malignancy is mostly driven by its unique ability to rapidly disseminate and form distant metastasis. We, therefore, investigated TRPV2 expression with respect to melanoma invasiveness, by quantifying TRPV2 transcripts along with POU3F2(BRN2) transcription factor mRNAs, a well-established marker of the melanoma invasive phenotype (Fane *et al*, 2019). While very low levels of TRPV2 mRNAs were present in normal human epithelial melanocytes (NHEM), a gradual increase in TRPV2 transcripts correlated with the rise of BRN2 expression in melanoma cells (ranging from the noninvasive 501mel, to the superficial spreading melanoma WM793, then to the metastatic melanoma WM266.4 and 451Lu; Herlyn *et al*, 1990; Juhasz *et al*, 1993; Arozarena *et al*, 2011a; Arozarena *et al*, 2011b; Tichet *et al*, 2015; Arozarena & Wellbrock, 2017) (Fig 1H and Appendix Fig S1D). In the broader CCLE melanoma cell lines dataset, the overall expressions of TRPV2 and POU3F2(BRN2) were also correlated (Appendix Fig S1E). In addition to TRPV2 mRNA expression, both TRPV2 protein levels (Fig 2A) and its functionality, which was assessed upon TRPV2 channel overactivation with the potent agonist cannabidiol (CBD) (Qin *et al*, 2008) (Appendix Fig S2A), mimicked the expression of the BRN2 invasiveness marker. Altogether suggesting a link between the expression of functional TRPV2 channels and the BRN2-associated invasive phenotype of melanoma cell lines.

TRPV2 channels are addressed to the plasma membrane and active in metastatic melanoma cells

Plasma membrane (PM) trafficking has been postulated as an important regulatory mechanism for TRPV2 activity, notably in response to a mechanical stress (Nagasawa & Kojima, 2015; Mignen *et al*, 2017). Surface protein biotinylation experiments evidenced that the subset of TRPV2 channels present at the PM increased gradually with the invasive potential of the tested melanoma cell line (Fig 2A). When assessed by confocal immunofluorescence, TRPV2 was consistently detected at the PM of the highly metastatic melanoma cell lines, WM266.4 and 451Lu (Appendix Fig S2B). The measurement of the nonstimulated Ca^{2+} influx, using the Mn^{2+} quenching assay, next correlated the amplitude of the constitutive Ca^{2+} entry to the extent of TRPV2 distribution at the PM (Fig 2B). Indeed, in the 501mel cells, where the sparse TRPV2 labeling remained mostly intracellular, the quenching rate was weak as opposed to both metastatic cell lines harboring PM-resident TRPV2, with the 451Lu cells displaying the highest constitutive Ca^{2+} entry.

To determine whether resting Ca^{2+} entries directly depend upon TRPV2 function in advanced melanoma, we modulated TRPV2 expression in three selected cell lines with well-defined, but opposite invasive phenotypes (Fig 1H, Appendix Fig S1D; Herlyn *et al*, 1990; Juhasz *et al*, 1993; Arozarena *et al*, 2011a; Arozarena *et al*, 2011b; Tichet *et al*, 2015; Arozarena & Wellbrock, 2017). WM266.4 cells are a classical model of dedifferentiated and invasive melanoma cells, often used as the phenotypic counterpart to the differentiated and poorly metastatic 501mel cell line (Arozarena *et al*, 2011a, 2011b; Chapman *et al*, 2014; Rowling *et al*, 2020). The highly invasive and migratory human 451Lu cell line, was established as a human melanoma metastasis model, based on its ability to spontaneously metastasize in nude mice (Herlyn *et al*, 1990). Additionally, when compared to other melanoma cell lines, WM266.4 and 451Lu cells exhibited the highest TRPV2 expression (see Figs 1G and 2A for direct comparison). Hence, TRPV2 was overexpressed in the melanocytic and noninvasive 501mel cell line. Conversely endogenous TRPV2 expression was silenced with two different shRNA sequences in the highly metastatic WM266.4 and 451Lu cell lines. Successful TRPV2 overexpression or repression was confirmed by immunoblotting (Fig 2C), and Ca^{2+} imaging experiments further validated the channel functionality (Appendix Fig S2C). GFP-TRPV2 overexpression in 501mel cells yielded an elevated response to CBD, whereas TRPV2 silencing (shRNA-V2) in WM266.4 and 451Lu cells, hampered CBD-induced Ca^{2+} influx. Resting Ca^{2+} signals in nonstimulated adherent cells then showed that TRPV2 overexpression in 501mel cells doubled the basal Ca^{2+}-influx, while TRPV2 silencing in WM266.4 and 451Lu cells impaired it (Fig 2D and E). In unstimulated metastatic melanoma cells, a subset of TRPV2 channels is, therefore, addressed to the PM and is active, allowing Ca^{2+} entry.

Figure 2. TRPV2 expression, plasma membrane targeting, and contribution to basal Ca^{2+} influx, increase with melanoma cell aggressiveness. ▶

A Analysis for total and plasma membrane (PM) TRPV2 expression in melanoma cell lines. β-actin was used as loading and cell integrity control. PM fractions were isolated using the surface protein biotinylation technique. The higher molecular weight bands of TRPV2 correspond to the glycosylated and mature channel (Barnhill *et al*, 2004).

B Constitutive Ca^{2+} influx comparison between resting 501mel (Light blue), WM266.4 (Blue) and 451Lu cells (Dark blue) assessed using the Fura-2 Mn^{2+} quenching assay. Representative traces of Fura-2 quenching kinetics are shown on the top panel (each point represents the mean of technical quadruplicates normalized to the baseline). The bar graph summarizes the quantification of the measured quenching rates and is presented as mean ± SEM ($n > 3$ biological replicates). $^{**}P = 0.0032$ and $^{****}P < 0.0001$, ordinary one-way ANOVA followed by Dunnett's multiple comparisons post-test.

C TRPV2 overexpression in the non-invasive 501mel cell line (GFP-TRPV2) or downregulation by lentiviral-delivery of TRPV2 specific shRNAs (shRNA V2-1 and V2-2) in the WM266.4 and 451Lu metastatic melanoma cell lines assessed by western-blot. β-actin was used as a loading control.

D Representative traces of Fura-2 Mn^{2+} quenching rates in control (GFP) or GFP-TRPV2 overexpressing 501mel cells, or in control (shRNA Ctrl) or TRPV2 repressed (shRNA V2) WM266.4 and 451Lu cells (each point represents the mean of technical quadruplicates normalized to the baseline).

E Average normalized quenching rates (as in D) presented as mean ± SEM ($n > 3$ biological replicates). $^{**}P = 0.0095$, the Mann–Whitney test (left panel); $^{*}P = 0.0399$–0.0336 (middle panel) and $^{*}P = 0.0438$, $^{**}P = 0.0033$ (right panel) the Kruskal–Wallis test followed by Dunn's multiple comparisons test.

Source data are available online for this figure.

 © 2023 The Authors

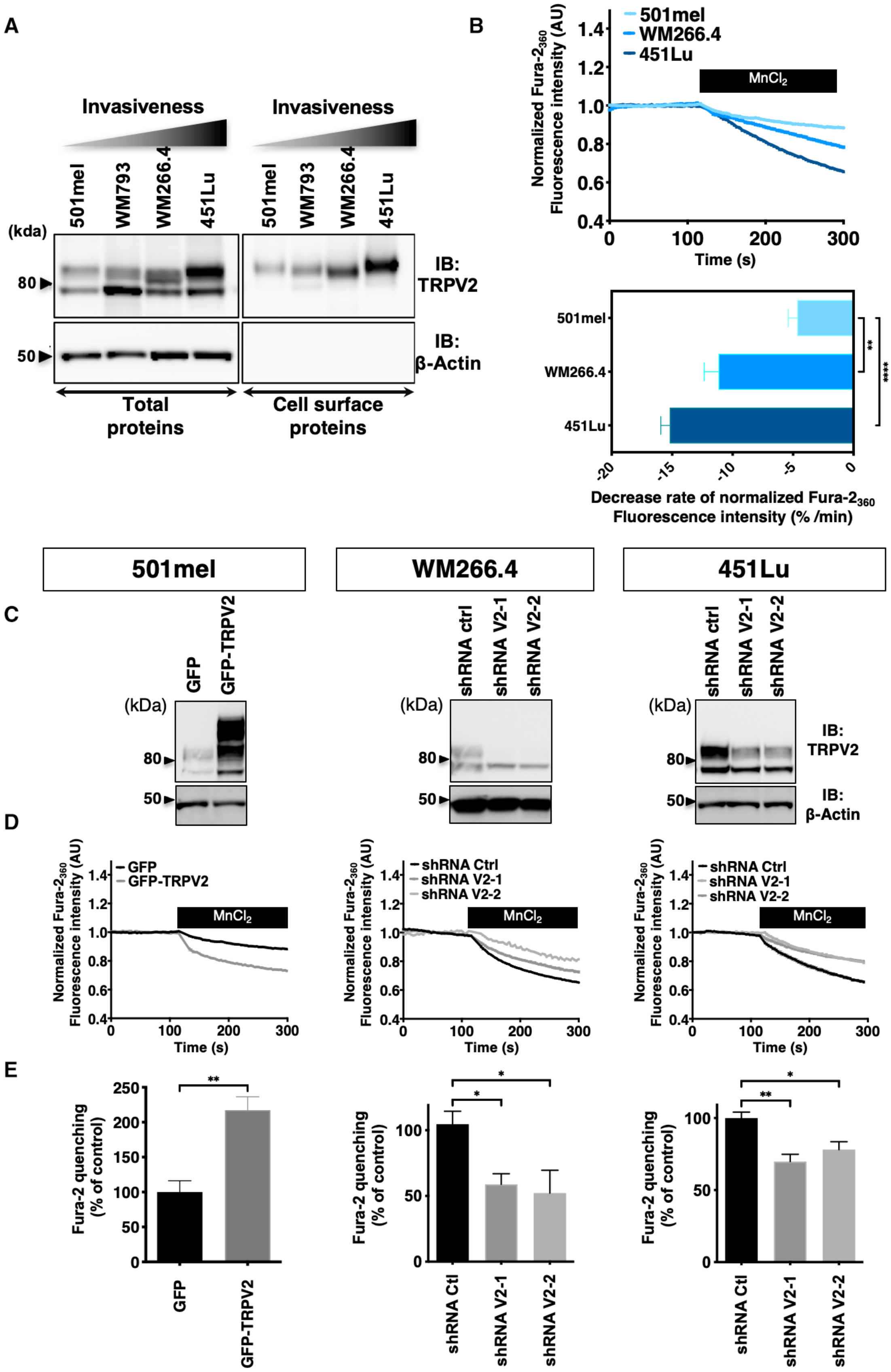
A
Invasiveness
Invasiveness
501mel
WM793
WM266.4
451Lu
501mel
WM793
WM266.4
451Lu
(kda)
80
50
IB: TRPV2
IB: β-Actin
Total proteins
Cell surface proteins
B
501mel
WM266.4
451Lu
MnCl2
Normalized Fura-2360 Fluorescence intensity (AU)
Time (s)
Decrease rate of normalized Fura-2360 Fluorescence intensity (% /min)
501mel
WM266.4
451Lu
**
****
501mel
WM266.4
451Lu
C
GFP
GFP-TRPV2
(kDa)
shRNA ctrl
shRNA V2-1
shRNA V2-2
IB: TRPV2
IB: β-Actin
D
GFP
GFP-TRPV2
shRNA Ctrl
shRNA V2-1
shRNA V2-2
MnCl2
Time (s)
E
Fura-2 quenching (% of control)
GFP
GFP-TRPV2
shRNA Ctl
shRNA V2-1
shRNA V2-2

Figure 2.

TRPV2 is dispensable for cell proliferation but is essential for melanoma tumor cell migration and invasion

Depending on the tumoral context, TRPV2 has been shown to be specifically involved in proliferation, and/or in the progression toward a pro-invasive phenotype (Siveen *et al*, 2020). We, therefore, evaluated whether TRPV2 expression affects either of these hallmarks in melanoma. Although, TRPV2 overexpression increased the growth rate of the noninvasive 501mel cells, TRPV2 silencing had no impact on cell viability or ERK phosphorylation in both metastatic cell lines, suggesting that TRPV2 is dispensable for malignant melanoma proliferative/survival behavior (Fig EV1A and B).

However, TRPV2 overexpression in the 501mel cells was sufficient to increase both their migratory and invasive capacities by 2.5 and 1.3 folds respectively (Fig 3A). Reciprocally, upon TRPV2 silencing in the metastatic melanoma cell lines (WM266.4 and 451Lu), both serum-induced migration and, to a greater extent, invasion (through a matrigel layer) were strongly hampered (by 40–80% and 65–95%, respectively). Additionally, a similar "anti-migratory" effect was obtained on WM266.4 cells treated with Tranilast, a pharmacological inhibitor of TRPV2 (Appendix Fig S3A), reinforcing the crucial role of TRPV2-dependent Ca^{2+} entry in the migration and invasion potentials of melanoma cells.

A detailed motility analysis of the WM266.4 cells, presenting well-adapted morphological characteristics for individual cell 2D-tracking, was carried out in the presence of a serum gradient (Appendix Fig S3B). Differentially labeled shRNA-Ctrl and -V2 expressing cells were mixed, seeded, and recorded simultaneously. Although cell spreading and lamellipodial protrusions appeared as constant features, motility was considerably altered in TRPV2-silenced cells, as attested by a robust reduction of speed and random displacement, compared to control cells (Fig 3B and C). Additionally, to recapitulate the *in situ* tumor-confined environment, WM266.4 cells were grown as melanospheres, embedded in collagen-I matrices, and 3D dynamics was followed for 3 days. Corroborating our 2D-data, TRPV2 repression drastically precluded the 3D-invasion capacity of metastatic melanoma cells (Fig 3D and E and Appendix Fig S3C). Interestingly, we did not observe any significant difference in the size of the melanospheres formed by the WM266.4 cells, whether expressing TRPV2 or not (unpublished observations), confirming that TRPV2 silencing does not disrupt advanced melanoma cells proliferation or survival potentials.

Migratory melanoma cells display high plasticity, and their migration could result from a combination of several interconnected mechanisms, notably a pseudo-epithelial–mesenchymal transition (pseudo-EMT) and interactions with the microenvironment. When we assessed EMT-associated markers, 501Mel and WM266.4 cells exhibited distinctively opposite profiles, epithelial- or mesenchymal-like, respectively, while 451Lu cells presented an intermediate phenotype. Most importantly, neither TRPV2 overexpression nor its downregulation altered the levels of the pseudo-EMT markers tested (Appendix Fig S4).

TRPV2 associates with nascent adhesive structures in metastatic melanoma cells

Cell migration requires highly coordinated interactions between the extracellular matrix and the intracellular cytoskeleton via multiprotein adhesion structures (Ridley *et al*, 2003). Upon mechanical tension, these structures dynamically transition from nascent adhesions to focal complexes and then to focal adhesions (FAs) (Gardel *et al*, 2010). In spite of a critical role established for Ca^{2+} signaling in both adhesion and actin cytoskeleton dynamics (Schwab *et al*, 2012; Wei *et al*, 2012), no study in cancer cells inferred a direct function for the mechanosensitive channel TRPV2 in these processes. However, a large-scale proteomic analysis detected TRPV2 as the only Ca^{2+} channel present in adhesion structures (Robertson *et al*, 2015), prompting us to explore its potential interaction with the acto-adhesive machinery in the dynamic context of migration. In migrating metastatic WM266.4 and 451Lu cells, PM endogenous TRPV2 was frequently distributed at the leading edge of the cells, forming defined clusters near the lamellipodia. In these proximal spots, TRPV2 preferentially colocalized with the marker of nascent adhesion, paxillin, while colocalization events with the markers of mechanically engaged FAs, vinculin or activated pY397-FAK were barely detectable (Fig 4A) (Gardel *et al*, 2010). A combination of proximity-ligation assays and spatial distribution analysis of molecules by dual-color direct stochastic optical reconstruction microscopy (dSTORM) on migrating melanoma cells, confirmed the preferential co-clustering of TRPV2 channels with paxillin- rather than vinculin-containing structures (Fig 4B and C). Additionally, we noted that TRPV2 colocalized with F-actin and physically interacts with vimentin (Fig 4A and Appendix Fig S5A and B), further arguing for the involvement

Figure 3. TRPV2 is essential for melanoma tumor cell migration and invasion.

A Impact of TRPV2 genetic manipulation in 501mel, WM266.4 and 451Lu on serum-induced migration and matrigel invasion. Representative pictures show crystal violet-stained cells that have migrated through 8 μm pore size membranes. Histograms illustrate the average numbers of migrating/invading cells normalized to control (presented as mean ± SEM of $n = 3$ independent experiments). For 501mel the Mann–Whitney test was used for statistical analysis (**$P = 0.0022$); For WM266.4&451Lu one-way ANOVA followed by Dunnett's multiple comparisons tests was used (**$P < 0.01$; ***$P < 0.001$; ****$P < 0.0001$; See Appendix Table S2 for exact P-values).

B Tracks comparison between fibronectin-plated shRNA Ctrl or shRNA V2 WM266.4 cells migrating towards a serum gradient over a 12 h period ($n = 10$ and 12 cells, respectively) (See also Appendix Fig S3B).

C Velocity analysis of 2D migration experiments described in B (dot, single cell, $n = 19$–21). Scatter plots show mean ± SD. ****$P < 0.0001$, the Mann–Whitney test.

D TRPV2-silencing effect on 3D invasion. Representative images were taken every day for 3 days after collagen embedding of spheroids from WM266.4 cells expressing either shRNA ctrl or TRPV2-targeting shRNAs (scale bar = 0.5 mm).

E Quantification of collagen invasion. For each spheroid, the cell-covered area at day 2 was normalized to the starting area of the collagen-embedded spheroids. Histograms represent the invasion relative to control WM266.4 spheroids (shRNA Ctrl) from at least 12 spheroids from three independent experiments (See also Appendix Fig S3C). M. ****$P < 0.0001$ and **$P = 0.0012$ the Kruskal–Wallis test followed by Dunn's multiple comparisons test.

Source data are available online for this figure.

 © 2023 The Authors

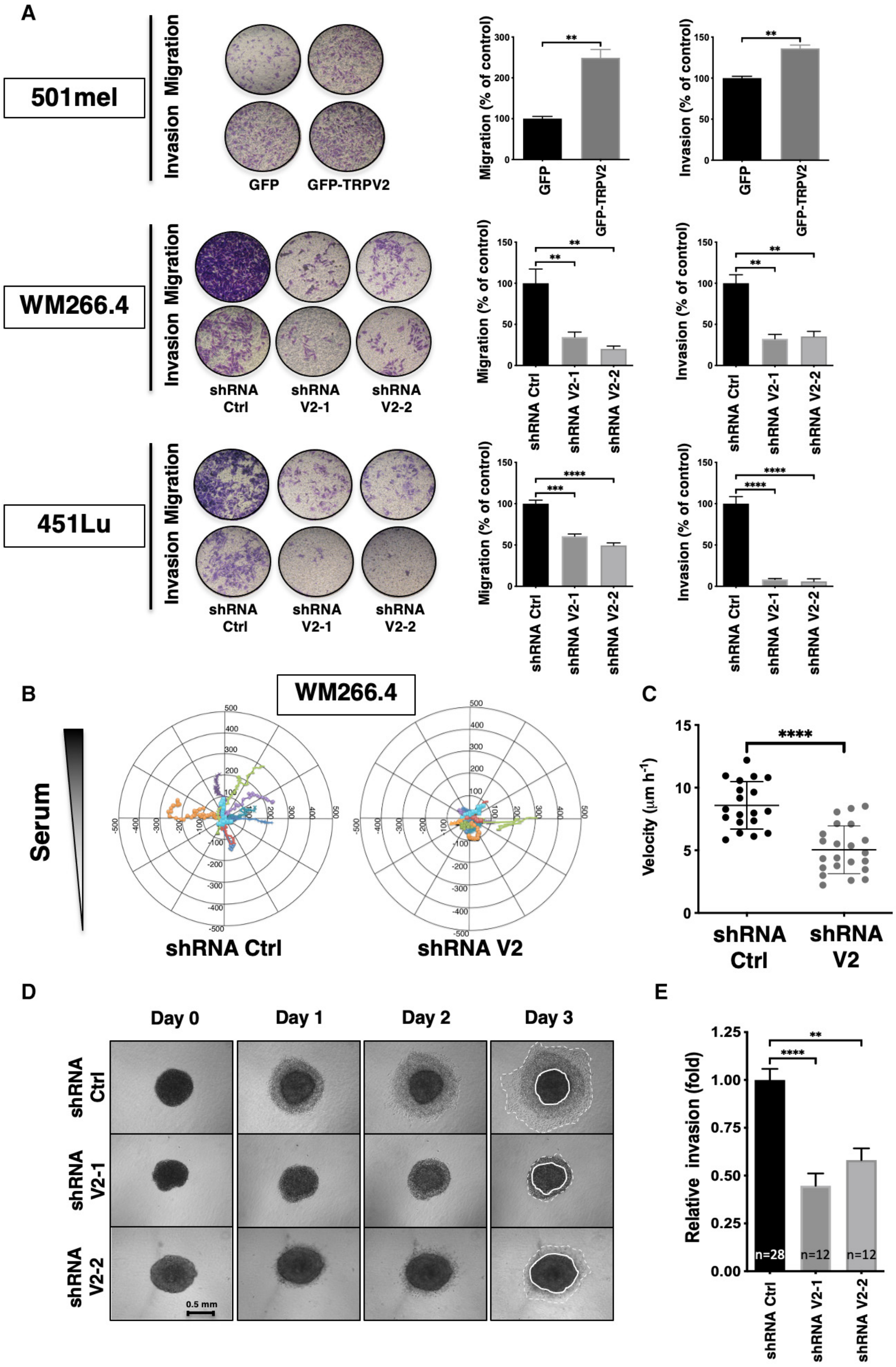
A
501mel
Invasion Migration
GFP GFP-TRPV2
Migration (% of control)
Invasion (% of control)
WM266.4
shRNA Ctrl
shRNA V2-1
shRNA V2-2
451Lu
B
WM266.4
Serum
shRNA Ctrl
shRNA V2
C
Velocity (μm h-1)
D
Day 0 Day 1 Day 2 Day 3
0.5 mm
E
Relative invasion (fold)
n=28 n=12 n=12

Figure 3.

of TRPV2 channel in the mechanical regulation of nascent adhesions (Jiu *et al*, 2015; Liu *et al*, 2015a).

TRPV2 modulates melanoma tumor cell migration through the control of the calpain-dependent maturation of adhesions and actin cytoskeleton remodeling

To investigate how TRPV2 participates in adhesion complex dynamics, we evaluated TRPV2 impact over FAs maturation. Mechanically engaged adhesions were detected and quantified following vinculin staining (Fig 5A and Appendix Fig S6A). Overexpressing TRPV2 in the 501mel cell line reduced by 23% the occurrence of FAs, while TRPV2 silencing in the WM266.4 and 451Lu cells increased the frequency of FAs by 27 and 24%, respectively. Of note, modulating TRPV2 expression either way did not impact total vinculin levels (Appendix Fig S6B). A known Ca^{2+}-dependent mechanism for adhesion disassembly involved the calpain-mediated proteolysis of talin, a mechanosensitive adhesion protein described as a key regulator of the initial step of adhesomes assembly, notably by interfering with vinculin recruitment and adhesions mechanical engagement (Haining *et al*, 2016; Chen *et al*, 2018; Schumacher *et al*, 2021). We, therefore, tested whether TRPV2 could regulate the adhesive-associated activity of calpains. In the noninvasive cell line, TRPV2 overexpression induced a 2-fold increase in calpain basal activity compared to MOCK cells, while reciprocally TRPV2 silencing in both metastatic melanoma cell lines halved the protease activity (Fig 5B). Regarding talin proteolysis, which largely depends upon extracellular Ca^{2+} signaling in our melanoma models (Appendix Fig S6C and D), it exactly mirrored the fluctuations of calpain activity resulting from TRPV2 modulation (Fig 5C and D). Talin cleaved isoform (190 kDa) increased by 75% in 501mel cells overexpressing TRPV2 compared to control cells mostly exhibiting full-length form (230 kDa), reflecting FAs stability. In the malignant WM266.4 and 451Lu cells, talin cleaved isoform was distinctly detected, in turn corroborating the adhesion plasticity of these highly migrating cells. Upon TRPV2 silencing, concomitantly to the enhanced adhesion and loss of motility, the cleavage of talin decreased by 60–80%. Formally connecting calpain activity to TRPV2-dependent migration, the pharmacological inhibition of calpains with selective inhibitors (Calpeptin or PD150606, blocking either the active site or both Ca^{2+}-binding domains of the protease) reversed the enhanced motility induced upon TRPV2 overexpression in 501mel cells (Fig EV2A and B and Appendix Fig S6E). Consistently, overexpressing a constitutively active form of calpain (Glading *et al*, 2004) rescued most of the migrative defects resulting from TRPV2 silencing in metastatic cells (Fig EV2C and D). Hence, we showed that TRPV2 regulates calpain activity, and the subsequent cleavage of the early adhesion assembly protein talin, in order to control adhesion dynamics and further established that the modulation of calpain activity underpins the mechanistic basis of TRPV2-mediated control over melanoma cells migration.

Adhesions growth cooperatively reinforces the link between the extracellular matrix (ECM) and the cytoskeleton, with adhesion components such as talin present at this interface. As TRPV2 activation has been related to mechanically induced actin assembly/disassembly rate (Sugio *et al*, 2017), we analyzed in migrating CMM models filamentous actin (F-actin) cytoskeleton and quantified F-actin bundles normalized to the cell area (Fig EV3A and B). Despite overall steady melanoma cells areas, TRPV2 overexpression in 501mel cells was by itself sufficient to considerably promote F-actin accumulation, whereas TRPV2 silencing in aggressive melanoma cells disrupted F-actin network. In a previous proteomic screen, we have identified cofilin-1, a known modulator of F-actin dynamics, as a potential TRPV2-interacting protein (unpublished data). Here, we showed that TRPV2 physically interacts with cofilin-1 in metastatic melanoma cell lines (Fig EV3C). In WM266.4 cells and, to a lesser extent in 451Lu cells, we observed that TRPV2 silencing reduced the level of inactive cofilin-1 (phosphorylated form), suggesting that TRPV2 represses cofilin-1 activity (Fig EV3D). Taken together, our results highlight a new role for TRPV2 in regulating advanced melanoma cell motility through the control of the calpain-mediated mechanical maturation of nascent adhesions, conjointly to cofilin-1-induced reorganization of the actin cytoskeleton.

TRPV2 expression is critical to the *in vivo* metastatic potential of melanoma tumor cells

To determine whether TRPV2 activity ultimately impacts the formation of metastasis *in vivo*, we injected bioluminescent human melanoma cells displaying modulated expression levels of TRPV2 into the tail vein of immunocompromised mice, and followed metastasis formation by bioluminescence imaging (BLI). At the assay end point, *in vivo* BLI showed a tendency, albeit not statistically significant, toward an increasing metastatic potential for the GFP-TRPV2 overexpressing 501mel cells (Fig 6A). Meanwhile, *ex vivo* BLI at necropsy uncovered a substantial increase of the metastatic burden in mice injected with GFP-TRPV2 overexpressing cells compared to controls (Fig 6B), revealing that TRPV2 expression was sufficient to

Figure 4. TRPV2 specifically associates with paxillin, a marker of nascent adhesive structures.

A Representative confocal images of low confluency WM266.4 and 451Lu metastatic melanoma cells seeded on fibronectin-coated coverslips. Cell nuclei are depicted with DAPI in blue, TRPV2 in green and indicated proteins (actin, paxillin, vinculin or pY397-FAK) in red (scale bar = 20 μm). Insets are magnifications of the indicated area. Arrows indicate sites of colocalization.

B Representative confocal images of the *in situ* detection of endogenous TRPV2 interactions with paxillin and vinculin by proximity-ligation assays (PLA). Low confluency WM266.4 and 451Lu metastatic melanoma cells plated on fibronectin-coated coverslips were stained for F-actin (green), cell nuclei (blue) and PLA reaction using antibodies specific of the indicated proteins (red) (scale bar = 20 μm; identical for all pictures). Red fluorescent spots indicate the association of the two proteins of interest, close to 40 nm. Scatter plots represent the quantification of the number of PLA spots per cell (bars indicate the medians) between TRPV2 and a control antibody (IgG), paxillin (PAX), or vinculin (VCL) (n = 29–51 cells from at least three independent experiments).

C Super-resolution (dSTORM) imaging of the clustering of TRPV2 channels with paxillin, but not with vinculin, in migrating WM266.4 cells (Scale bar = 5 μm). a and b insets show expanded views of a region of the cell and arrows highlight TRPV2 channel and paxillin co-clusters.

Source data are available online for this figure.

© 2023 The Authors

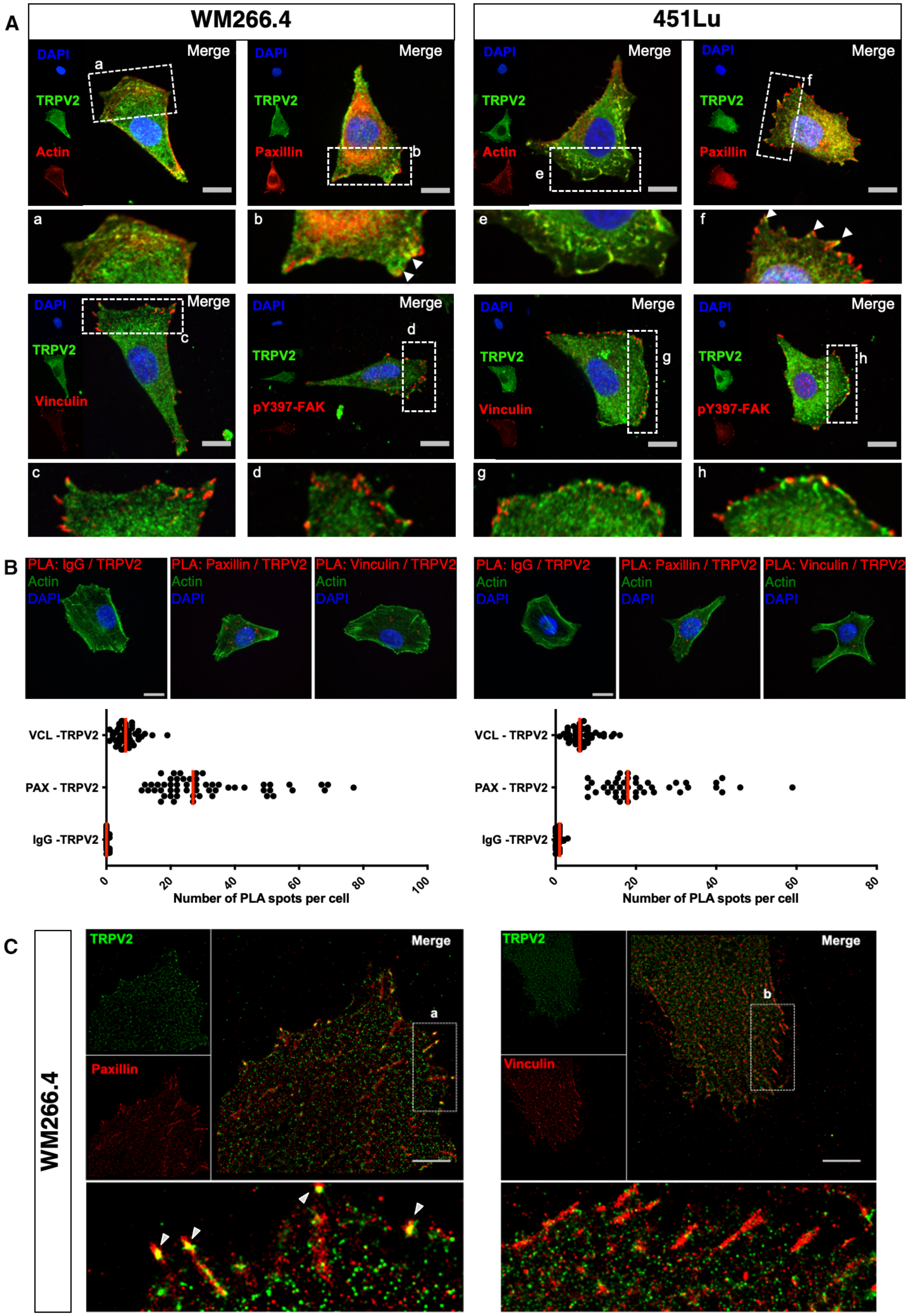
A
WM266.4
451Lu
DAPI
TRPV2
Actin
Paxillin
Vinculin
pY397-FAK
Merge
B
PLA: IgG / TRPV2
PLA: Paxillin / TRPV2
PLA: Vinculin / TRPV2
VCL -TRPV2
PAX - TRPV2
IgG -TRPV2
Number of PLA spots per cell
C
WM266.4

Figure 4.

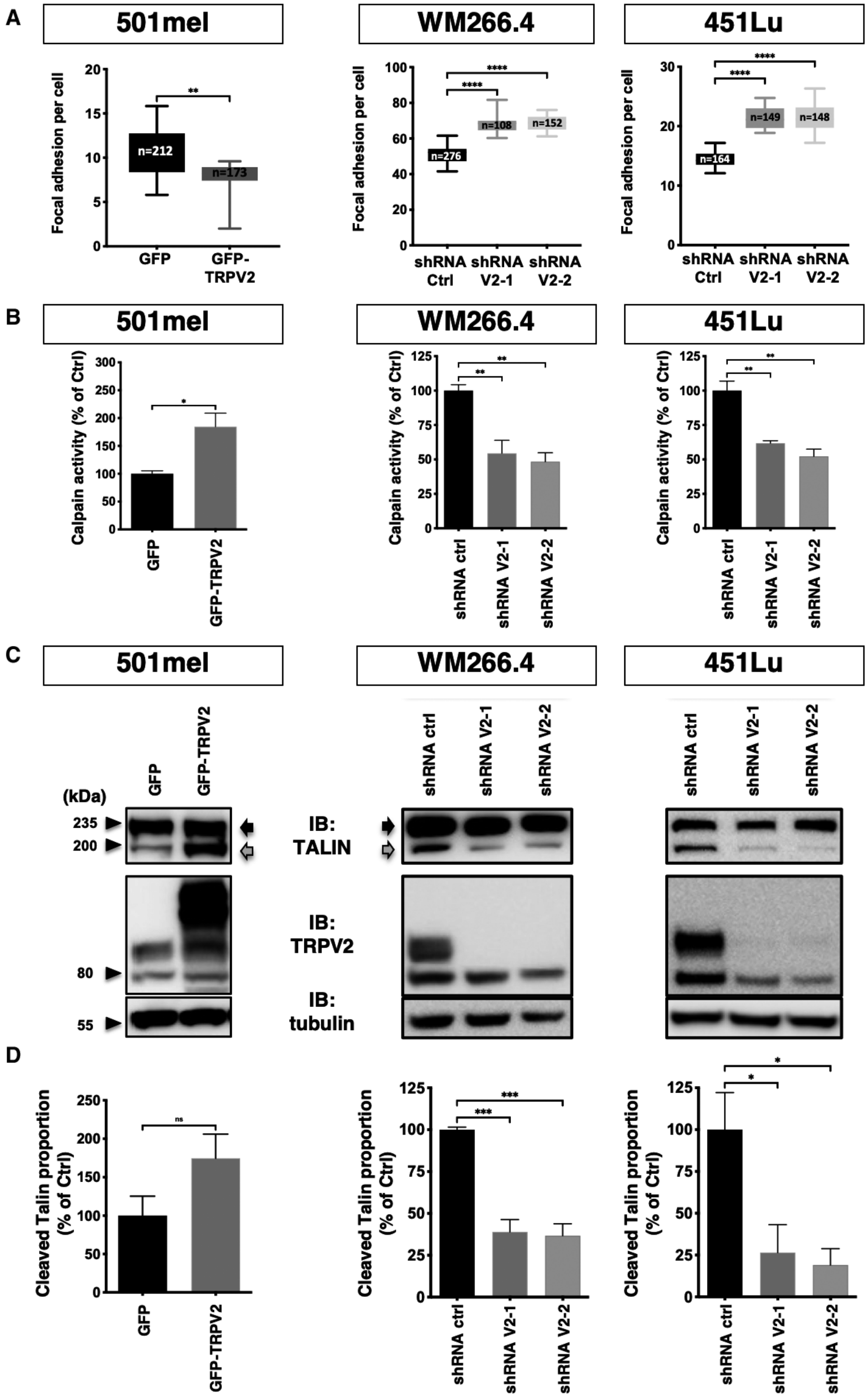

Figure 5.

 © 2023 The Authors

Figure 5. TRPV2 activity controls the calpain-dependent mechanical maturation of focal adhesions.

A Quantification of vinculin-stained focal adhesion sites per cell. Confocal images taken on low confluency melanoma cells seeded on fibronectin-coated coverslips were analyzed using the imageJ software to count the number of vinculin clusters per cell. The total number of cells counted for each cell line is indicated in the boxes. Data are presented as box and whiskers plots (Boxes extend from the 25th to 75th percentiles, whiskers from min to max, the horizontal line in each box is plotted at the median). $^{**}P = 0.0066$, the Mann–Whitney test (501mel); $^{****}P < 0.0001$ the Kruskal–Wallis test followed by Dunn's multiple comparisons tests (WM266.4 and 451Lu).

B Comparison of calpain activity in control (GFP) or overexpressing TRPV2 (GFP-TRPV2) 501mel cells, and in control (shRNA Ctrl) or TRPV2-silenced (shRNA V2-1, -2) WM266.4 cells or 451Lu cells. Bar graphs show mean normalized calpain activity ± SEM ($n = 3$ biological replicates). For 501mel the Mann–Whitney test was used for statistical analysis ($^{*}P = 0.05$); For WM266.4&451Lu one-way ANOVA followed by Dunnett's multiple comparisons tests was used ($^{**}P < 0.01$; See Appendix Table S2 for exact *P*-values).

C Representative immunoblots showing the full-length (230 kDa; Black Arrows) and the calpain-mediated degradation product (190 kDa; Gray Arrows) of talin after overexpression or repression of TRPV2, in the corresponding cell lines. Tubulin was used as a loading control.

D Densitometric analysis of the cleaved-talin ratios from three independent experiments (as described in C) normalized to control. Data are presented as mean ± SEM. For 501mel the Mann–Whitney test was used for statistical analysis (ns $P = 0.2$); For WM266.4&451Lu one-way ANOVA followed by Dunnett's multiple comparisons tests was used ($^{*}P < 0.05$; $^{***}P < 0.001$; See Appendix Table S2 for exact *P*-values).

Source data are available online for this figure.

endow 501mel cells with metastatic competences. Numerous metastatic foci could be observed in lungs, bones, and brain (Fig EV4A and B) yet small and rather dim, which likely accounts for the lack of detection of luminescence in the entire animal. In parallel, as early as 24 h postinjection, TRPV2 repression in 451Lu cells prevented their extravasation into the lungs (Appendix Fig S7). At the assay end point, *in vivo* BLI showed that TRPV2-silenced melanoma cells have lost their metastatic potential in mice, as compared to TRPV2-expressing control cells (Fig 6C). *Ex vivo* BLI confirmed that TRPV2 repression tends to decrease long-term lung colonization (Fig 6D), although the quantification of individual foci by BLI was likely underestimated as shown by the immunofluorescence analysis of lung sections eventually evidencing numerous individual metastatic lesions in 451Lu control-injected mice, retaining endogenous TRPV2 expression that is strongly detected at the cell periphery (Appendix Fig S8A–C). TRPV2 requirement for the formation of melanoma metastasis was likewise validated in the experimental model of xenografted zebrafish allowing a direct comparison of the metastatic potential of two different cell lines in the same organism. To distinguish between control-shRNA and TRPV2-shRNA WM266.4 expressing cells that are both GFP-labeled, control cells were double-stained with the red fluorescent dye Cm-Dil. Then, equal amounts of both shRNA-expressing cells were mixed and co-injected in the duct of Cuvier of 2 day old zebrafish embryos. Thirty-six hours posttransplantation, only double-stained control cells, expressing TRPV2, have disseminated throughout the fish body (Appendix Fig S9A and B). Altogether, these *in vivo* results established that melanoma tumor cells rely at least in part on TRPV2 to succeed in disseminating and form distant metastases.

TRPV2 expression in human melanoma is a marker of advanced malignancy and bad prognosis

The interdependence between TRPV2 and the metastatic phenotype of melanoma tumor cells *in vitro* and *in vivo* prompted us to evaluate the clinical significance of TRPV2 expression in human melanoma. Initially, we performed *in silico* analyses of RNAseq data from either the TCGA SKCM versus the matched TCGA and GTEx normal datasets, or the GSE46517 (Kabbarah *et al*, 2010) dataset (Fig 7A and Appendix Fig S11A). Consistent with previous observations on NHEM versus melanoma cell lines (Fig 1H), TRPV2 mRNA level was found significantly higher in melanoma tumors, including primary and metastatic lesions, as compared to healthy or nevi samples. Using combined immunohistochemical and tissue microarray (TMA) analyses (Fig 7B–D and Appendix Fig S10A–D), we assessed TRPV2 expression *in situ* over 100 patient samples, including 62 malignant melanomas (Grade I-IV), 20 lymph node metastases and 18 benign nevi. Analysis of this tissue cohort demonstrated that overall, malignant melanomas and lymph node metastasis were extensively expressing TRPV2, while benign nevi exhibited faint or no staining (Fig 7B–D and Appendix Fig S10C). A closer look at the results suggested a tendency toward a positive correlation between the progression of the disease and TRPV2 expression. However, the statistical analysis did not support these observations, likely due to interindividual variability as well as a limited number of high-grade lesions (4 grade III and 2 grade IV) (Fig 7C and D and Appendix Fig S11B). Yet, a similar trend was observed on stages I and IV melanomas randomly picked from the tumor biobank of the Rennes university hospital (Appendix Fig S11C).

Figure 6. TRPV2 expression level dictates the *in vivo* metastatic potential of human melanoma tumor cells xenografted in mice.

A–D Representative bioluminescence imaging (BLI) data of mice injected intravenously with the nonmetastatic melanoma cell line 501mel-Luc transfected with either GFP-TRPV2 or GFP control (A, B), or with the invasive 451Lu-Luc melanoma cell line expressing either control shRNA or TRPV2 targeting shRNA (C, D). Tumor growth and metastasis formation were monitored for 64 days (501mel-Luc) or 35 days (451Lu-Luc) after injection. Graphs presented in A and C show *in vivo* normalized photon flux quantification at the end time point (ns $P = 0.0903$ (A) and $^{**}P = 0.0064$ (C), the Mann–Whitney test). Graphs presented in (B) and (D) show the number of metastatic foci per animal (B) or per lungs (D) counted at necropsy ($^{*}P = 0.0397$ (B) and ns $P = 0.0691$ (D), unpaired *t*-tests). For (D) Representative *ex vivo* BLI images of lung metastasis are shown (Scale bar = 5 mm).

Data information: Boxes extend from the 25th to 75th percentiles, whiskers from min to max, the horizontal line in each box is plotted at the median and each dot correspond to a single mouse ($n = 6$–7).
Source data are available online for this figure.

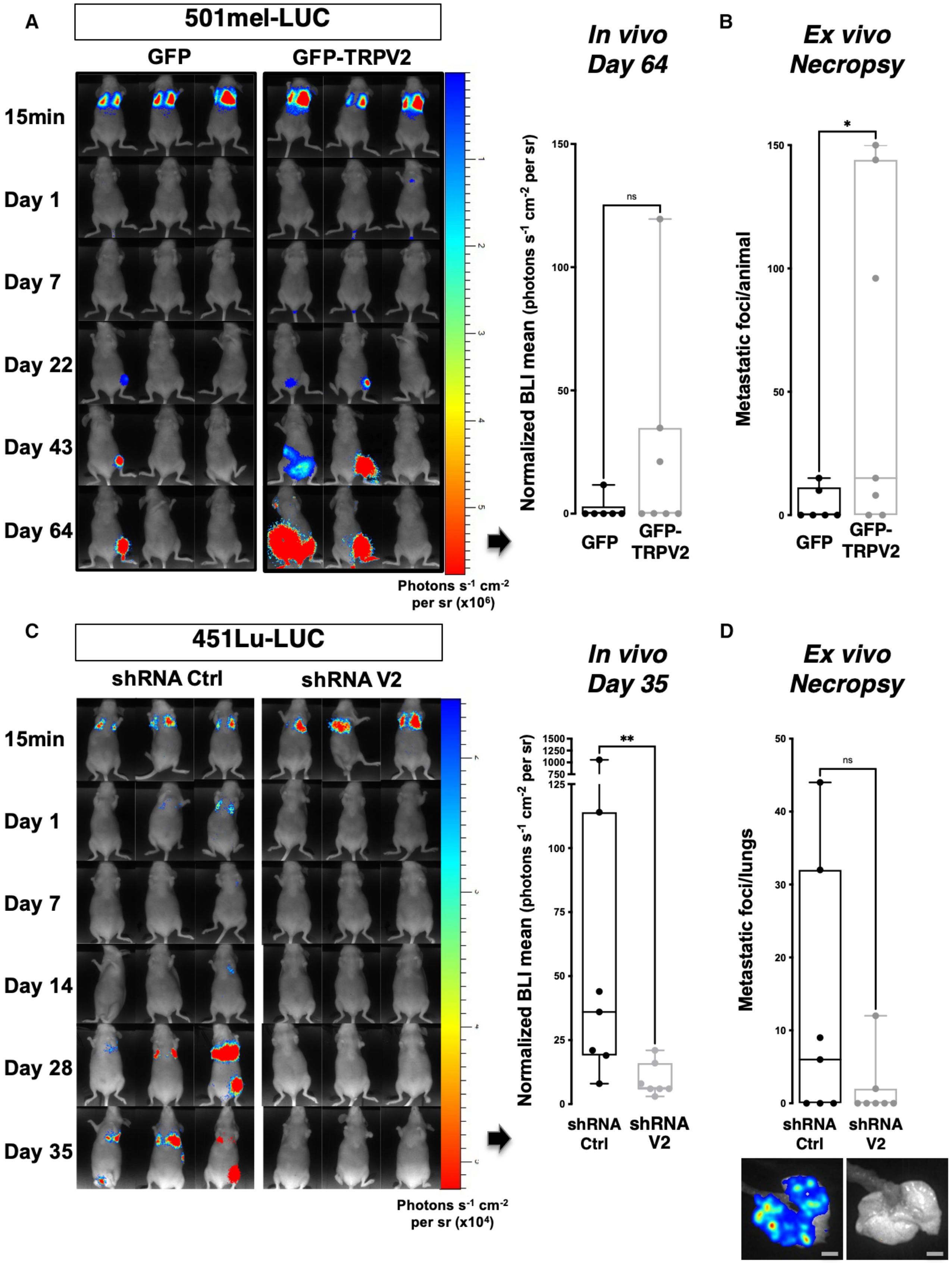
A
501mel-LUC
GFP
GFP-TRPV2
15min
Day 1
Day 7
Day 22
Day 43
Day 64
Photons s-1 cm-2 per sr (x10^6)
In vivo Day 64
Normalized BLI mean (photons s-1 cm-2 per sr)
ns
GFP
GFP-TRPV2
B
Ex vivo Necropsy
Metastatic foci/animal
*
GFP
GFP-TRPV2
C
451Lu-LUC
shRNA Ctrl
shRNA V2
15min
Day 1
Day 7
Day 14
Day 28
Day 35
Photons s-1 cm-2 per sr (x10^4)
In vivo Day 35
Normalized BLI mean (photons s-1 cm-2 per sr)
**
shRNA Ctrl
shRNA V2
D
Ex vivo Necropsy
Metastatic foci/lungs
ns
shRNA Ctrl
shRNA V2

Figure 6.

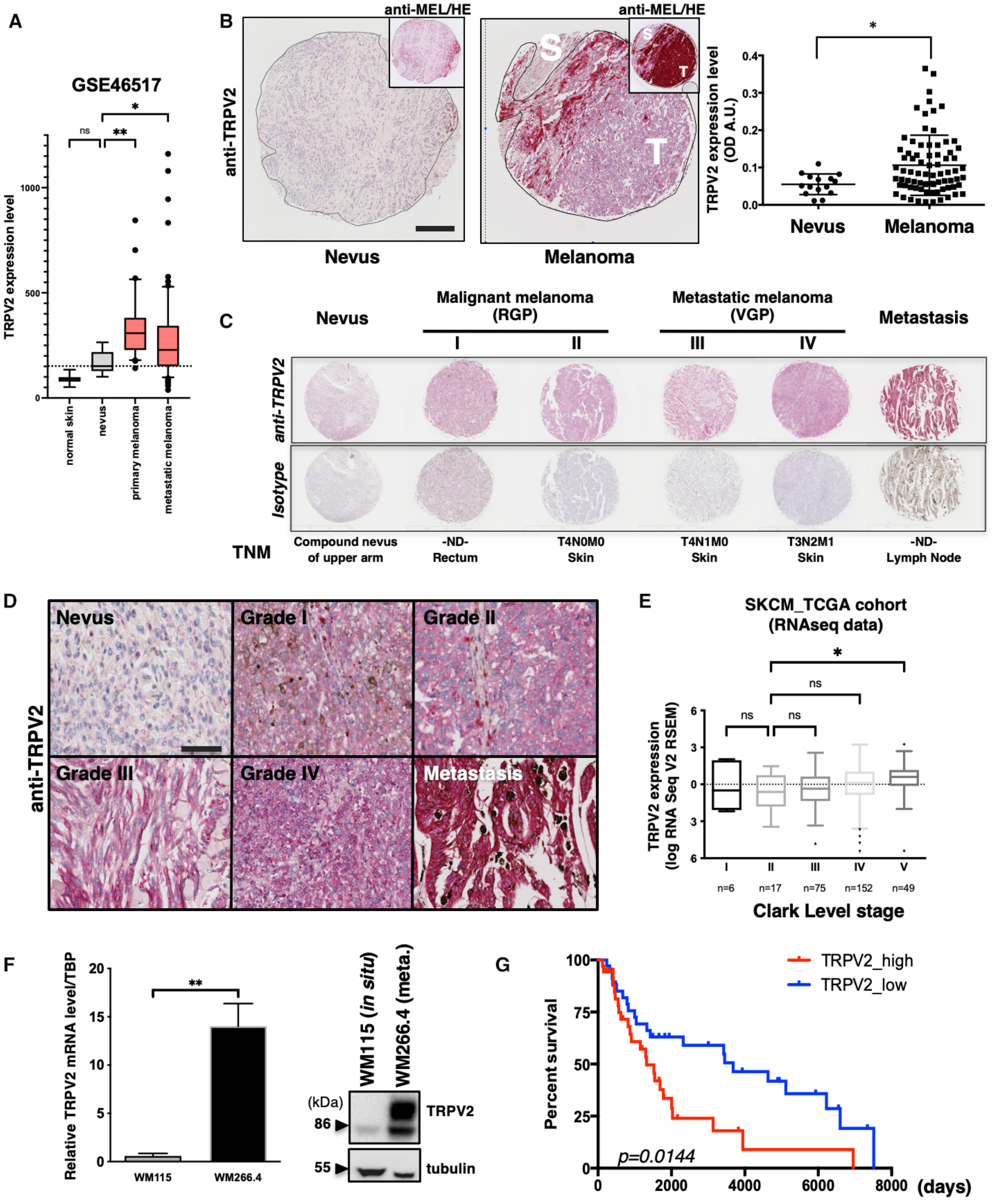
A
GSE46517
TRPV2 expression level
normal skin
nevus
primary melanoma
metastatic melanoma
B
anti-TRPV2
anti-MEL/HE
Nevus
Melanoma
TRPV2 expression level (OD A.U.)
C
Nevus
Malignant melanoma (RGP)
Metastatic melanoma (VGP)
Metastasis
anti-TRPV2
Isotype
TNM
Compound nevus of upper arm
-ND- Rectum
T4N0M0 Skin
T4N1M0 Skin
T3N2M1 Skin
-ND- Lymph Node
D
anti-TRPV2
Nevus
Grade I
Grade II
Grade III
Grade IV
Metastasis
E
SKCM_TCGA cohort (RNAseq data)
TRPV2 expression (log RNA Seq V2 RSEM)
n=6
n=17
n=75
n=152
n=49
Clark Level stage
F
Relative TRPV2 mRNA level/TBP
WM115
WM266.4
WM115 (in situ)
WM266.4 (meta.)
(kDa)
TRPV2
tubulin
G
TRPV2_high
TRPV2_low
Percent survival
p=0.0144
(days)

Figure 7.

Figure 7. TRPV2 expression is associated with malignancy and poor prognosis in human melanoma.

A TRPV2 RNA expression levels (microarray probe: 219282_s_at) measured in normal skin ($n = 7$ biological replicates), nevi ($n = 9$ biological replicates), primary ($n = 31$ biological replicates) and metastatic ($n = 73$ biological replicates) melanomas (GEO: GSE46517; Data ref: Kwong *et al*, 2013). TRPV2 levels are increased in malignant lesions compared to normal skin and nevi. Data are represented as a box and whiskers plot with outliers plotted as individual points (Boxes extend from the 25th to 75th percentiles, whiskers from the 10th to 90th percentiles, the horizontal line in each box is plotted at the median; ns $P = 0.1383$, *$P = 0.0488$ and **$P = 0.0016$, the Kruskal–Wallis test). The horizontal dotted line corresponds to TRPV2 median expression in nevi.

B Representative melanoma tissue lesions from a tissue microarray (TMA) comparing TRPV2 staining between a benign nevus and a malignant melanoma (See also Appendix Fig S10) (Scale bar = 200 µm). Top right insets show the same images stained with an anti-melanoma triple cocktail (HMB45+A103 (Melan-A)+T311(Tyrosinase)). Regions depicted with a black line represent the interface between the diffusely stained tumor (T) and the surrounding normal stroma (S). Right panel shows the scatter plot of the complete quantification of TRPV2 staining in 77 patients with malignant melanoma compared to 16 nevi tissues (dot, single patient sample) with lines for mean ± SEM (*$P = 0.0473$, the Mann–Whitney test).

C Comparison of six representative tissues from patients at different TNM grades of melanoma progression stained with anti-TRPV2 or the isotype control. RGP, radial growth phase; VGP, vertical growth phase.

D Representative images showing detailed TRPV2 staining from *in situ* melanoma lesions with progressive TNM grades and from lymph node (LN) metastasis (Scale bar = 50 µm).

E TRPV2 mRNA expression (from the TCGA cohort RNAseq data) according to Clark level pathological cancer stages. This grading system describes the level of anatomical invasion of the melanoma; I: confined to the epidermis (*in situ*); II: invasion into the papillary dermis; III: invasion to the junction of the papillary and reticular dermis; IV: invasion into the reticular dermis; V: invasion into the subcutaneous fat. TRPV2: mRNA expression z-scores relative to all samples are represented as a box and whiskers plot with outliers plotted as individual points (Boxes extend from the 25th to 75th percentiles with Tukey whiskers, the horizontal line in each box is plotted at the median; n are indicated on the plot; ns $P = 0.5647$ for II vs. IV or > 0.9999 for all other comparisons, *$P = 0.0348$, the Kruskal–Wallis test).

F Analysis of TRPV2 expression by quantitative RT-PCR (left panel) and western blot (right panel) in the WM115/WM266.4 pair of isogenic melanoma cell lines. The WM115 cell line was derived from an *in situ* tumor while the WM266.4 was established from a skin metastasis of the same patient. Relative transcript levels are presented as mean ± SEM from $n = 4$–9 biological replicates (**$P = 0.0028$, the Mann–Whitney test).

G Kaplan–Meier plot showing the association of TRPV2 expression (10% highest vs. 10% lowest expressers in the skin cutaneous melanoma cohort TCGA dataset) with melanoma patient survival (*$P = 0.0144$, the Log-rank (Mantel–Cox) test).

Source data are available online for this figure.

Moreover, analysis of TRPV2 transcript levels according to the Clark staging system, defining anatomical invasion, revealed that higher the expression of TRPV2 was, the deeper the tumor had penetrated into the skin layers (Fig 7E). Comparison of isogenic cell lines pairs, WM164/451Lu and WM115/WM266.4, further established that TRPV2 expression was higher in metastasis-derived cell lines compared to the poorly invasive matched cell lines originated from *in situ* tumors (Figs 1G and 7F). Most importantly, survival analysis evidenced that melanoma tumors expressing high levels of TRPV2 correlated with shorter life expectancies in patients, compared to low TRPV2 expressers (Fig 7G).

Discussion

TRPV2 is a nonselective cation channel showing a high Ca^{2+} permeability, which can be activated upon mechanical stress (Nagasawa & Kojima, 2015; Sugio *et al*, 2017; Katanosaka *et al*, 2018). TRPV2 is highly expressed in the nervous systems (where it enhances axonal outgrowth in developing neurons) and in immune cells (where it notably participates in phagocytosis, cytokine secretion, migration/chemotaxis and inflammation) (Shibasaki *et al*, 2010; Santoni *et al*, 2013; Cohen *et al*, 2015). In cancers, the overexpression of TRPV2 was associated with increased survival of patients with hepatocellular carcinoma, glioma and glioblastoma, while the opposite was seen for esophageal squamous cell carcinoma, urothelial carcinoma, prostate, breast and gastric cancer (Santoni *et al*, 2020). Overall, TRPV2 expression has been found deregulated in patients with advanced metastatic disease compared to primary solid tumors (Siveen *et al*, 2020). Here, we established that in melanoma tumor cells the expression of functional TRPV2 channels was correlated with invasiveness, making TRPV2 mandatory for the dissemination and formation of distant metastases *in vivo*. Similarly, in human melanoma biopsies, TRPV2 expression increased together with tumor progression, invasive phenotype, metastatic potential and ultimately a shorter life expectancy. Consistently with the above reports based on tumors from different origins, our results set TRPV2 as a valuable prognosis marker in melanoma tumor progression.

Among the biological processes regulated by TRPV2 channel during metastatic progression, TRPV2 has been notably associated with proliferation, for instance in esophageal squamous cell carcinoma (Kudou *et al*, 2019), or with the progression toward a pro-invasive phenotype in prostate and bladder cancers (Monet *et al*, 2009; Liu & Wang, 2013; Oulidi *et al*, 2013). Melanoma is an aggressive cancer endowed with unique features of cellular plasticity, coupled with a rare ability to switch back and forth between proliferative and invasive phenotypes. Using gain- and loss-of-function approaches, we established that TRPV2 expression and activity potentiates the acquisition of both the migratory and invasive phenotypes of melanoma cells, while dispensable for their proliferative/survival behaviors. Note that specifically addressing TRPV2 role, by modulating its expression, prevents the issue of indirect/off-target Ca^{2+} effects often raised with pharmacomodulators that may be accountable for some disparities seen in the literature.

The acquisition of an invasive phenotype through an EMT program is, in some cancer cells, regulated by Ca^{2+} signaling (Pedri *et al*, 2021; Van den Eynde *et al*, 2021). In metastatic melanoma cells, we investigated whether the mechanistic basis for TRPV2-mediated aggressive potential could rely on such reprogramming which, in this specific case, is referred as pseudo-EMT since melanocytes are not epithelial cells and their invasive state may not be exclusively mesenchymal. Nevertheless, modulating TRPV2 expression—either way—had no impact on the expression of EMT markers. We, however, noted that the noninvasive 501mel versus the highly invasive WM266.4 cells exhibited antagonistic markers

 © 2023 The Authors

profiles, corresponding to either a pseudo-epithelial or a mesenchymal-associated signature, respectively, and suggesting that the WM266.4 have completed a transition process to acquire their metastatic potential. These observations were consistent with their morphology and migrative features, as well as with the expression of the BRN2 invasiveness marker. Regarding the 451Lu cells, the expression profiles of pseudo-EMT and BRN2 markers were representative of a previously described "intermediate phenotype" (Rambow *et al*, 2019). Cells in a "partial" state defined as capable of both proliferation and migration, are matching with the 451Lu extremely invasive potential associated with a mesenchymal/amoeboid morphology and fewer engaged acto-adhesive structures compared to the WM266.4 cells. Interestingly, these "intermediate" cells are the ones expressing the highest level of TRPV2. Since melanoma progression can occur in the absence of the conventional "phenotype switching" (Tuncer *et al*, 2019), these signatures are reflecting the highly heterogeneous and dynamic properties of metastatic melanoma cells, capable of adopting a unique range of phenotypes, which have been described as driven by the microenvironment (Gabbireddy *et al*, 2021).

The tumor microenvironment (TME) is indeed thought to contribute to the process of metastasis, and is clearly of importance for both cellular migration and invasion (Ju *et al*, 2018). In unstimulated melanoma cells, we evidenced that TRPV2 is localized at the PM and is active, yet we excluded a growth factor-dependent regulation of its trafficking (unpublished observations). We, however, know that the dynamic endosomal-PM translocation of TRPV2, regulating its activity (Kojima & Nagasawa, 2014), can be induced by mechanical cues (Karki & Tojkander, 2021). The cAMP-dependent protein kinase A (PKA) and the PI3K/AKT pathways have been shown to regulate TRPV2 trafficking to the PM (Stokes *et al*, 2004; Nagasawa & Kojima, 2015), and intriguingly both pathways are, respectively, negatively or positively regulated by PIEZO1 (Hung *et al*, 2016; Zhang *et al*, 2022), a stretch-activated channel involved in adhesion maturation and confinement-induced migration (Canales Coutino & Mayor, 2021; Yao *et al*, 2022; Zhang *et al*, 2022).

Hence, adhesion dynamics coupled with the mechanical constraints applied to the PM could be critical factors controlling TRPV2 activity. Consequently, the regulation of TRPV2 permeation would globally impact the resting Ca^{2+} homeostasis, which has been directly correlated to melanoma aggressiveness (Arozarena *et al*, 2011b). The constant changes in cell-matrix contact points and cell shape are guided by mechanical cues triggering adhesion dynamics and actin cytoskeleton remodeling (Burridge & Guilluy, 2016). Soon after the cell-matrix engagement, the adapter protein paxillin gets recruited to form nascent adhesions. To date, TRPV2 is the only Ca^{2+}-permeant channel reported as directly participating in paxillin-rich adhesive structures, revealing a mechanism through which TRPV2 could boost melanoma tumor cell invasiveness. Regulations of these nascent adhesion structures may directly influence sensing, forces generation and maturation into adhesion complexes that drive the cell body forward. Generally, paxillin clusters are found at the proximal end of the adhesion site, where paxillin strongly binds to integrins during the early phases of FA formation, and where F-actin also enters and accumulates, defining paxillin as initiating highly dynamic acto-adhesive complexes (Legerstee *et al*, 2019). In migrating CMM cells, we also determined that TRPV2 prompted F-actin filament accumulation together with cofilin-1 inactivation. We showed that TRPV2 physically interacts with cofilin-1, an actin severing factor known to coordinate the spatiotemporal organization of F-actin during cell migration by integrating transmembrane signals (Lehtimaki *et al*, 2017). Knowing that mechanically induced rearrangements of actin depend on TRPV2 during axonal outgrowth (Sugio *et al*, 2017), and that intracellular Ca^{2+} increments have been shown to promote actin assembly to improve melanoma cell migration (Baljinnyam *et al*, 2010), we postulated that in nascent adhesion, the mechanical stimulation of TRPV2 signaling contributes to F-actin bundles structure stabilization, by promoting cofilin-1 inactivation. Interestingly, in advanced melanoma models, TRPV2 also associates with the intermediate filament vimentin network, conceivably in order to extensively regulate cytoskeletal organization and adhesion structures mechanical maturation (Jiu *et al*, 2015; Liu *et al*, 2015a). Note that as such, vimentin expression often correlates with tumor aggressiveness (Strouhalova *et al*, 2020).

Directional migration of the cell requires the continuous, coordinated formation and turnover of adhesions at the leading edge of the cell body and release of this attachment at the rear. As the cell leading edge advances, a subpopulation of nascent adhesions disassembles, while some grow and mature into focal complexes and then FAs. Nascent adhesion assembling is supported by the recruitment of the mechanosensitive protein talin, which can bind directly to paxillin but also to actin and integrins (Haining *et al*, 2016). In metastatic melanoma models, we observed that TRPV2 activity regulates calpain activation and the ensuing cleavage of talin. With the Ca^{2+}-dependent protease calpain being a major regulator of adhesion components degradation and its substrate, talin, directly impacting the recruitment of cytoskeletal adapters and the mechanical engagement of adhesions (Chen *et al*, 2018; Schumacher *et al*, 2021). To date, the plasma membrane elements controlling the calpains system have been poorly described, raising the question of its mechanosensitive regulation. In migrating metastatic melanoma cells, TRPV2 mediates at least part of the Ca^{2+} signal activating calpain-mediated proteolysis of talin, hindering the mechanical maturation of adhesions while favoring their turnover. TRPV2 preferential localization to nascent adhesions further implies a spatiotemporal regulation of calpain activity, in order to coordinate adhesion and efficient migration. In line with this, the inhibition of FA stabilization substantially increased the instantaneous speeds of amoeboid-migrating melanoma cells (Liu *et al*, 2015b). Interestingly, in metastatic breast cancer and head and neck carcinoma cells, the conversion to amoeboid migration, enabling a low-adhesive and energy-conserving migration strategy, has been recently described as involving the calpain-mediated cleavage of talin (preprint: te Boekhorst *et al*, 2020). Hence, TRPV2-mediated activation of calpain promoting the spatiotemporally regulated proteolysis of talin, and the resulting adhesion dynamic could be an adaptive mechanism specific to cells endowed with fast migrating features such as the metastatic melanoma cells.

In conclusion, CMM, which is traditionally viewed as one of the most metastatic and therapy-resistant malignancy, remains an incurable disease for the great majority of patients and, consequently, is in great need for specifying the molecular mechanisms underpinning metastatic dissemination. Over the last decade, it appeared

© 2023 The Authors

evident that Ca^{2+} channels act as important regulators of specific steps in tumor progression (Bruce & James, 2020; Tajada & Villalobos, 2020). We hereby reported a central role for the prominently expressed Ca^{2+}-conducting TRPV2 channel during the dynamic process of melanoma cells metastatic dissemination and identified calpains as one of TRPV2 key functional targets in that context. Hence, based on our results we propose the following model (Fig EV5): Recruitment of TRPV2 at the PM within paxillin-rich proximal nascent adhesion structures places this mechanosensitive channel at the interface between the metastatic cell intracellular machinery and the TME. In highly invasive metastatic melanoma cells, TRPV2-mediated Ca^{2+} influx, potentially induced by mechanical forces, induces the cleavage of talin (and potentially of other substrates) by the Ca^{2+}-dependent calpains, spatiotemporally regulating cell adhesion dynamics. Concomitantly, TRPV2 enables F-actin stabilization by directly controlling cofilin-1 activity. As a central component of the F-actin/adhesion/ECM interface, TRPV2 coordinates dynamic cytoskeletal rearrangements intertwined with active adhesion turnover. TRPV2, therefore, represents a great molecular candidate for mediating a tunable force-transmitting structural linkage from the cytoskeleton to the TME via adhesion complexes, controlling *in fine* melanoma cell migration.

As TRPV2 directly correlates to the aggressiveness of the tumor and to patient mortality in human melanoma biopsies, it stands out as a valuable biomarker for malignant tumors with bad prognosis. Due to their accessibility to pharmacological modulation and their exposure at the cell surface, Ca^{2+} channels represent a propitious class of drug targets. Therefore, TRPV2 pharmacological blockade hints as a promising therapeutic option for migrastatics in the treatment of advanced-stage melanoma.

Materials and Methods

Reagents and Tools table

Reagent/resource	Reference or source	Identifier or catalog number
**Experimental models**		
501mel (human melanoma)	Dr. R. Halaban (Yale University School of Medicine, New Haven, USA)	
WM266.4 (human melanoma)	ATCC	Cat# CRL-1676
451Lu (human melanoma)	Drs. M. Herlyn and J. Villanueva (The Wistar Institute, Philadelphia, USA)	
NHEM (Normal Human Epithelial Melanocyte from for skin)	BIOalternatives (Gençay, France)	
NMRI nude mice (*M. musculus*)	Charles river	
SHrN® hairless NOD.SCID mice (*M. musculus*)		
Melanoma tissue microarray	US Biomax	Cat# ME1004c
**Recombinant DNA**		
pcDNA3.1(+)-Zeocin_eGFP	This study	
pcDNA3.1(+)-Zeocin_eGFP-TRPV2 (human)	This study	
NON SILENCING shRNA_pGIPZ (shRNA control)	Thermo Scientific/GE Dharmacon	Cat# RHS4346
hTRPV2 shRNA_pGIPZ (shRNA V2-1)	Thermo Scientific/GE Dharmacon	Cat# RHS4531-EG51393 cloneID#RHS4430-200207433-V2LHS_97630
hTRPV2 shRNA_pGIPZ (shRNA V2-2)	Thermo Scientific/GE Dharmacon	Cat# RHS4531-EG51393 cloneID#RHS4430-200281506-V3LHS_387624
pMX_GFP-CAPN2 S50E	Dr. O. DESTAING (IAB, Grenoble, France)	
**Antibodies**		
Mouse monoclonal anti-beta Actin (clone AC-74)	Sigma-Aldrich	Cat# A5316, RRID:AB_476743
Rat mnoclonal anti-CD31 (PECAM-1) (clone SZ31)	Dianova	Cat# DIA-310, RRID:AB_2631039
Rabbit monoclonal anti-Cofilin (clone D3F9) XP®	Cell Signaling Technology	Cat# 5175, RRID:AB_10622000
Rabbit monoclonal anti-Phospho-Cofilin (Ser3) (clone 77G2)	Cell Signaling Technology	Cat# 3313, RRID:AB_2080597
Rabbit monoclonal anti-Phospho-FAK (Tyr397) (clone D20B1)	Cell Signaling Technology	Cat# 8556, RRID:AB_10891442
Rabbit monoclonal anti-Phospho-p44/42 MAPK (Erk1/2) (Thr202/Tyr204) (clone D13.14.4E) XP®	Cell Signaling Technology	Cat# 4370, RRID:AB_2315112
Mouse monoclonal anti-Paxillin (clone 349)	BD Biosciences	Cat# 612405, RRID:AB_647289

 © 2023 The Authors

Reagents and Tools table (continued)

Reagent/resource	Reference or source	Identifier or catalog number
Rabbit polyclonal anti-PMEL	Thermo Fisher Scientific	Cat# PA5-32491, RRID:AB_2549958
Mouse monoclonal anti-Talin (clone 8d4)	Sigma-Aldrich	Cat# T3287, RRID:AB_477572
Rabbit polyclonal anti-TRPV2 (VRL-1 H-105)	Santa-Cruz Biotechnology	Cat # sc-30155, RRID:AB_2209150
Rabbit polyclonal anti-TRPV2 (Prestige Antibodies® Powered by Atlas Antibodies)	Sigma-Aldrich	Cat # HPA044993, RRID:AB_10960889
Mouse monoclonal anti-Vimentin (clone V9)	Agilent	Cat# M0725, RRID:AB_10013485
Mouse monoclonal anti-Vinculin (clone VLN01)	Thermo Fisher Scientific	Cat# MA5-11690, RRID:AB_10976821
**Oligonucleotides and sequence-based reagents**		
PCR primers	This study and Spinsanti *et al* (2008)	Appendix Table S1
**Chemicals, enzymes and other reagents**		
SiR-actin kit	Spirochrome	Cat# SC001
Acti-Stain 555 Phalloidin	Cytoskeleton	Cat# PHDH1
Rhodamine Phalloidin	Thermo Fisher Scientific	Cat# R415
Fura-2, AM, cell permeant	Thermo Fisher Scientific	Cat# F1221
(−)-Cannabidiol	Tocris	Cat# 1570
Tranilast	Sigma-Aldrich	Cat# T0318
Calpeptin	Selleck Chemicals	Cat# S7396
PD150606	Medchem Express	Cat# HY-100529
Duolink® In Situ Red Starter Kit Mouse/Rabbit	Sigma-Aldrich	Cat# DUO92101
Sulfo-NHS-LC-Biotine EZ-Link™	Thermo Fisher Scientific	Cat# 21335
Pierce™ High Capacity Streptavidin Agarose	Thermo Fisher Scientific	Cat# 20361
Calpain Activity Assay Kit	PromoKine	Cat# PK-CA577-K240
Human plasma fibronectin	Sigma-Aldrich	Cat# F0895
**Software**		
FIJI v1.0	https://fiji.sc	Fiji (RRID:SCR_002285)
PRISM v9.3.1	GraphPAD	GraphPad Prism (RRID:SCR_002798)
MetaFLUOR	Universal imaging	MetaFluor Fluorescence Ratio Imaging Software (RRID:SCR_014294)
RTCA Software	Agilent/ACEA	RTCA Software (RRID:SCR_014821)
**Other**		
EnSpire® 2300 Multilabel Plate Reader	PerkinElmer	
xCELLigence RTCA DP System	Agilent/ACEA	Agilent xCELLigence RTCA eSight Real-Time Cell Analyzer (RTCA) (RRID:SCR_019571)
JuLI™ Stage Real-Time Cell History Recorder	NanoEnTek Inc	
AutoScratch Wound Making Tool	Agilent/BioTek	
Gemini twin wave electroporator	BTX	

Methods and Protocols

Cell lines

The WM266.4 human melanoma cell line was purchased from ATCC and cultured in Roswell Park Memorial Institute (RPMI) 1640 Medium plus 8% Fetal Bovine Serum (FBS). The 451Lu cells were provided by Drs. M. Herlyn and J. Villanueva (The Wistar Institute, Philadelphia, USA), the 501mel cells were a gift from Dr. R. Halaban (Yale University School of Medicine, New Haven, USA), and both human melanoma cell lines were cultured in Dulbecco's modified Eagle Medium (DMEM) plus 8% Fetal Bovine Serum (FBS). All cells were routinely tested for the absence of mycoplasma.

Plasmid constructs

The eGFP coding sequence from the pEGFP-C2 vector (Clontech), with or without the coding sequence of wild-type human TRPV2 (Penna *et al*, 2006) added in 3′, were inserted into the pcDNA3.1(+)-Zeocin vector (Life Technologies). All constructs were verified by sequencing. Validated nonsilencing control and TRPV2 targeting shRNAmir-pGIPZ lentiviral vectors were purchased from Dharmacon.

The plasmid encoding a GFP-tagged constitutively active calpain 2 S50E mutant was a gift from Dr. O. Destaing (IAB, Grenoble, France).

Lentiviral production and transfection/transduction procedures

Lentiviral particles production and cell transduction were performed according to manufacturer instructions. Briefly, 293SZ cells were co-transfected with shRNAmir-pGIPZ plasmids and the lentiviral psPAX2 and pMD2.G packaging plasmids in antibiotic-free medium using Ca^{2+}-phosphate–mediated transfection. Target cells were infected with freshly thawed lentiviral particles diluted in growth medium supplemented with polybrene (3 μg/ml). For transfection, cells were electroporated with 10 μg of DNA using an ECM-830 square wave or a Gemini twin wave electroporator (BTX Instrument Division, Harvard Apparatus). Selection of stable clones was achieved with selective doses of either zeocin or puromycin for 2–4 days, at which time mock cells were eradicated. For the transfected 501mel cell lines, cell sorting based on GFP fluorescence was performed on a FACSAria Fusion cytometer (Becton Dickinson).

Real-time quantitative PCR

Total RNA was isolated using the Nucleospin RNA II kit (Macherey-Nagel) following manufacturer's instructions. RNA concentrations were estimated using a NanoDrop analyzer ND1000 (Thermo-Fisher). 0.5 μg purified RNA was reverse transcribed in a volume of 20 μl using the High-capacity cDNA Reverse Transcription kit (Applied Biosystems) and random hexamers according to manufacturer's instructions. qPCR was performed on 0.5 ng cDNA samples, in sealed 96-well microtiter plates using the SYBR Green™ PCR Master Mix (Applied Biosystems) and gene-specific primer pairs (see Appendix Table S1) with the 7300SDS Real-Time PCR System (Applied Biosystems). The ΔΔCt method was used to calculate relative expression values, which were normalized to the housekeeping gene Tbp.

Bioinformatics analyses on publicly available data

Data on TRPV2 transcript expression patterns in the NCI-60 cell line set were generated by querying TRPV2 as input in CellMiner (http://discover.nci.nih.gov/cellminer/) as described in (Reinhold *et al*, 2012). The NCI-60 is a panel of 60 diverse human cancer cell lines used by the Developmental Therapeutics Program of the U.S. National Cancer Institute RRID:SCR_003057. Gene-centric RMA-normalized TRPV2 mRNA expression data in the Broad Institute and Novartis's Cancer Cell Line Encyclopedia (CCLE) larger cell panel was obtained through the CCLE website (https://portals.broadinstitute.org/ccle) (Barretina *et al*, 2012). To establish the differential plot of TRPV2 expression across all cancers plus the specific analysis in SKCM compared to healthy tissue, we analyzed RNA sequencing expression data of tumor and normal samples from The Cancer Genome Atlas (TCGA) and the GTEx projects (through the GEPIA web server (http://gepia.cancer-pku.cn/index.html; Tang *et al*, 2017)) as well as from the GEO: GSE46517 dataset (Kabbarah *et al*, 2010; Data ref: Kwong *et al*, 2013). TCGA was further interrogated for TRPV2 RNA expression across different tumor types and melanoma subgroups defined by the Clark level. Survival analysis (Kaplan–Meier estimate) was performed by comparing overall survival of the 10% highest to the 10% lowest TRPV2 expressers. The statistical significance was assessed with a Mantel–Cox log-rank test.

Biochemical techniques

Immunoblotting was performed as previously described (Penna & Cahalan, 2007). Cells were lysed in RIPA buffer (150 mM NaCl, 50 mM Tris, 0.1% v/v SDS, 0.25% v/v Na-Deoxycholate, 1% v/v NP-40, 1 mM EDTA; pH 7.4) supplemented with a protease and phosphatase inhibitor cocktail for 20 min on ice. The protein concentration in supernatant was estimated using the BCA assay according to Pierce's protocol. A total of 30 μg of protein was loaded into NuPage 4–12% gels and transferred onto a nitrocellulose membrane.

Co-immunoprecipitation experiments were performed using cells cultured in 100 mm fibronectin-coated dishes. After sample preparation, 1.5 mg of cell lysate per sample was used for immunoprecipitation as described elsewhere (Penna *et al*, 2008). Briefly, supernatants were tumbled for 20 min on ice with the appropriate primary antibody, followed by 3 h of incubation at 4°C with protein A-Sepharose beads (Sigma Aldrich). The beads were then washed five times with a lysis buffer, suspended in LDS sample buffer and heated for 5 min. The samples were resolved and blotted according to the above-described protocol.

For cell surface protein biotinylation assays, cells were cultured to 75% confluency in 6-well plate dishes, washed in cold PBS and incubated with the biotinylation reagent (Sulfo-NHS-LC-biotin 0.5 mg/ml in PBS pH8 with 0.1 mM $CaCl_2$, 1 mM $MgCl_2$) for 30 min at 37°C. Free biotinylation reagent was then removed by washing twice in PBS containing 50 mM glycine and 0.1% BSA and once in PBS alone. Cell lysates were prepared as described above in RIPA buffer supplemented with 25 mM NH_4Cl. 100 μg of total protein was incubated with 25 μl of High-capacity Streptavidin agarose resin for 1 h at 4°C. Captured proteins were eluted in NuPAGE-LDS sample buffer, 4% SDS, 160 mM DTT by heating 10 min at 70°C.

Mn^{2+} quenching assay

Basal Ca^{2+} permeability was measured in Fura-2 loaded adherent melanoma cells. Briefly, cells were seeded in black 96-well clear bottom microplates (10^5 cells/well), loaded with 5 μM Fura2/AM for 40 min at 37°C and washed with Ca^{2+}-free HBSS solution (132 mM NaCl, 5.4 mM KCl, 0.8 mM $MgCl_2$, 10 mM HEPES and 5.6 mM D-Glucose, pH 7.4). A baseline was established in Ca^{2+}-free HBSS solution then a final concentration of 215 μM $MnCl_2$ was added. Fluorescence emission at 510 nm was acquired every 3 s following Fura-2 excitation at its isosbestic point, 359 nm, on a multimode plate reader (Enspire, Perkin-Elmer). Mn^{2+} entry was measured as the rate of decline (quenching) of Fura-2 fluorescence intensity.

Single cell Ca^{2+} imaging

Cells plated on glass coverslips were loaded with 5 μM Fura-2/AM in culture medium at 37°C for 45 min and then washed three times in HBSS solution (142.6 mM NaCl, 5.6 mM KCl, 2 mM $CaCl_2$, 1 mM $MgCl_2$, 10 mM HEPES, 5 mM Glucose, pH 7.4) followed by de-esterification at 37°C for 15 min. Changes in $[Ca^{2+}]_i$ were monitored in cells bathed in HBSS using a DMIRB (Leica) inverted microscope-based imaging system equipped with a 40×/1.35 UApo N340 high UV light transmittance oil immersion objective (Olympus), a CoolSnapHQ fast-cooled monochromatic digital camera (Princeton instrument), a DG-4 Ultra High Speed Wavelength Switcher (Sutter Instruments) for fluorophore excitation and METAFLUOR software

© 2023 The Authors

(Universal Imaging) for image acquisition and analysis. Data were acquired every 10 s (emission at 510 nm) at 340 and 380 nm excitation wavelengths and all images were background subtracted. Cells with spontaneous or aberrant Ca^{2+} activity were identified by imaging and eliminated from the analysis. Depicted curves represent a minimum of three independent experiments.

Immunofluorescence staining and confocal microscopy
Glass coverslips were coated with human fibronectin (10 μg/ml) in Ca^{2+}/Mg^{2+}-PBS. WM266.4 and 451Lu cells seeded at low confluency were fixed in PBS-4% PFA-4% sucrose for 10 min, quenched with PBS-glycine 0.1 M for 30 min, then permeabilized and blocked with PBS-10% FCS-0.2% saponin for 15 min. Primary antibodies diluted in PBS-5% FCS-0.2% saponin incubation lasted 2 h. For immunodetection, secondary antibodies (diluted 1/1,000) were incubated for 30 min at 37°C. F-actin was detected using Acti-stain. Nuclei were stained with DAPI at 1 μg/ml for 15 min. Coverslips were mounted in Vectashield. Fluorescence micrographs were taken using laser scanning confocal systems (TCS SP8 model mounted on a DMI 6000 CS inverted microscope, Leica or IX81-based Olympus FV1000). For FAs counting (vinculin), fixation was done in 70% ethanol and no saponin was used.

Proximity ligation assay
Proximity-ligation assays (PLA) experiments were performed using the Red Mouse/Rabbit Duolink Starter Kit (Sigma-Aldrich) according to the manufacturer's instructions. Plating, fixation, permeabilization and blocking were done as described above. F-actin was detected using Acti-stain-488-Phalloidin. Fluorescence was analyzed using the confocal microscope IX81-based Olympus FV1000 with UPLSAPO-NA 1.35 60× oil objective and the IQ3 software (Andor). Maximum projection intensities (MIP) of images were created from z stacks with a step interval of 0.2 μm. Quantification of PLA fluorescent spots was carried out using the particle analyzer application of ImageJ, RRID:SCR_003070 software.

Single molecule imaging
Optical setup
We used an Olympus IX83 inverted microscope with an autofocus system. The excitation path was composed of three laser lines: 637, 532, and 405 nm (Errol lasers) and a TIRF module (Errol lasers) used in combination with a matched 390/482/532/640 multiband filter (LF405/488/532/635-A-000, Semrock). The fluorescence was collected through an Olympus x100 1.49 NA oil immersion objective lens. The detection path was composed of a SAFe module (Abbelight) and a Flash 4 v3 (Hamamatsu). The pixel size in the object was 100 nm.

Image acquisition
The diffraction limited epifluorescence images were acquired at low illumination irradiance (0.15 $kW.cm^{-2}$), while the dSTORM images were obtained using a high illumination irradiance (4 $kW.cm^{-2}$) until a sufficient molecule density was obtained (around 1 molecule per μm^2) and the acquisition could be started. The exposure time was set at 50 ms. Acquisitions were performed using the Nemo software (Abbelight). To achieve a single molecule regime in dSTORM acquisition, a dedicated buffer (Smart kit, Abbelight) was used.

Image processing
Acquired data were processed using the Nemo software (Abbelight). After removing the background signal, molecules were detected and the numbers of EPI and UAF photons were measured to extract the corresponding axial positions. Lateral drifts were corrected from the localized data thanks to a cross-correlation-based algorithm.

Cell proliferation assay
Cell viability was evaluated using the MTT assay. Briefly, 40,000 cells were cultured for 24–72 h in flat-bottom 96-well plates in a final volume of 100 μl. Then, 15 μl of MTT (5 mg/mL in PBS) solution were added and after 4 h of incubation at 37°C the absorbance was measured at 570 nm using the EnSpire® 2300 Multilabel Plate Reader (Perkin Elmer).

***In vitro* migration/invasion assays**
Migration and invasion experiments were performed using transwell migration assays. Briefly, 2 × 10^5 501mel or WM266.4 cells, or 4 × 10^5 451Lu cells suspended in serum-free media were added to the top chamber of transwell permeable supports (8 μm pores, Corning) coated or not with Matrigel (Corning). Chemo-attraction was induced by media supplemented with 10% FCS into the bottom chamber. After 12 h, cells were fixed in 70% cold ethanol and stained with Crystal Violet. Cells that have reached the downside of the porous membrane were counted using a cell counter (ImageJ). Alternatively, migration was monitored in real time using the xCELLigence RTCA DP System (RTCA, Roche Diagnostics, Mannheim, Germany) or in wound-healing assays. xCELLigence experiments were performed as recommended by the manufacturer. Briefly, 5 × 10^4 cells in 150 μl final volume of FCS-free media were added into each well of the upper chamber of a CIM-plate 16 (ACEA Biosciences, Inc., San Diego, CA, USA). Conditioned media (160 μl) supplemented with 16% FCS was added to the lower chamber of each well. In order to establish an extracellular matrix, the underside of the upper chamber was coated with 60 μl of human fibronectin (4 μg/ml) (Sigma-Aldrich, St. Louis, MO, USA) per well prior seeding. Cell migration toward the lower chamber was continuously monitored every 15 min, and data were collected and analyzed by the xCELLigence 1.2.1 software. For wound-healing assays, cells were seeded at high density (4 × 10^4 cells per well of a 96-well plate) in complete medium, enabling the attainment of confluence overnight. A BioTek AutoScratch wound making tool (Agilent, Santa Clara, CA, USA) was used to automatically create reproducible scratch wounds in cell monolayers and nonattached cells were removed by washing. Cells were then placed in complete media containing either pharmacological inhibitors or the DMSO vehicle. Refilling of the scratched area was followed by a time-lapse bright field imaging system (JuLI™ Stage Real-Time Cell History Recorder, NanoEnTek Inc, Seoul, Korea) taking images every 20 min during 24–72 h. For gap closure, the cell density was measured within replicated wells using the integrated JuLI™Stage confluence tool.

2D cell migration and cell tracking
Serum-starved WM266.4 cells expressing shRNA control or TRPV2 were seeded in chambers coated with fibronectin (Ibidi) filled with serum-free medium. Migration was induced with a 5% FCS gradient and carried out for 12 h. Pictures were taken using an inverted Olympus IX71 microscope equipped with a Cool SnapHQ camera

installed on a Delta Vision system. Images were analyzed using the SofWorX and ImageJ softwares.

Three-dimensional spheroid growth and invasion assay

Melanoma spheroids were prepared using the liquid overlay method. Briefly, 24-well culture plates (Corning) were coated with 1.5% agarose (Life Technologies) in sterile water. Cells from a single-cell suspension were added at 10,000 per well. The plates were incubated at 37°C in a 5% CO2 atmosphere until spheroids were formed (72 h). Spheroids were harvested and implanted into a gel of rat collagen I (Becton Dickinson). Complete melanoma medium was overlaid on top of the solidified collagen. Pictures of the invading spheroids were taken each day using an inverted microscope. Images were processed and growth areas measured using ImageJ software.

Calpain activity measurement

Calpain activity was measured using the calpain activity assay kit (Promokine). Briefly 2×10^6 cells were seeded on 6-well plates for 12 h, washed and suspended in 100 µl of extraction buffer. Two hundred microgram of proteins was diluted in 85 µl of the extraction buffer. Calpain activity was revealed by adding 10 µl of reaction buffer and 5 µl of fluorescent calpain substrate. Activity was measured using a fluorometer equipped with a 400 nm excitation filter and a 505 nm emission filter. One microliter of calpain inhibitor was added to subtract the background.

***Mouse models and in vivo* experiments**

All animal experiments were approved by the French animal care ethics committee in concordance with French and European Union laws (license #44565) and conformed to the relevant regulatory standards. Experimental metastasis studies were performed as previously described (Chantome *et al*, 2013; Tichet *et al*, 2015) on 6–8 week old female mice. Mice were housed at UTE-IRS1 (Nantes-University) under the animal care license #C44-278. Anesthetized mice were placed into a restraining device, 1×10^6 melanoma cells engineered to express a luciferase reporter gene (LUC cells) were suspended in 100 µl of PBS and injected through a 30-gauge needle into the tail vein of nude mice (SCID hairless NOD (SHrN™) for 501mel, or NMRI for 451Lu). Metastasis formation and relative amounts of tumor burden were assessed weekly using whole-body bioluminescent imaging (BLI) (ΦimageurTM; Biospace Lab). Mice were given 150 mg/kg body-weight of D-luciferin potassium salt (Interchim). Images were acquired 3–5 min after injection and collected in real time until plate saturation was reached. Photons count per second per steradian per square centimeter was recorded by a photon imager system. For BLI plots, photon flux was calculated by using a rectangular ROI encompassing the thorax using the software Photovision+ (version 1.3; Biospace Lab). This value was normalized to the value obtained immediately after injection, so that all mice had an arbitrary starting signal of 100. At necropsy, *ex vivo* BLI measurement was performed within 15 min after D-luciferin injection.

Zebrafish tumor cell implantation and micrometastasis analysis

Zebrafish embryos were raised, staged, and maintained according to standard procedure. For cell microinjection, 2 days post fertilization (dpf), phenylthiourea (PTU, Sigma-Aldrich)-treated zebrafish were dechorionized and anesthetized using 0.04 mg/ml Tricaine. WM266.4 human melanoma cells harvested in HyQTase™, counted and labeled or not with CM-Dil tracker were then mixed in equal quantity and loaded into borosilicate capillaries at a density of 8×10^7 cells/ml. Injections were performed using a pneumatic picopump (World Precision Instruments) and a micromanipulator. Cell injection was performed above the ventral duct of Cuvier as described in Teng *et al* (2013). After confirmation of a visible cell mass at the injection site, selected zebrafish were transferred to an incubator and maintained at 34°C for 36 h. Micrometastasis formation was analyzed on living zebrafish embryos anesthetized with Tricaine. Images were captured with a DeltaVision imaging system and processed using ImageJ/Fiji software.

Immunohistochemistry

The melanoma tissue microarray ME1004c (US Biomax Inc.) came with the clinical stage, gender, age, organ, TNM classification, and HMB45 profiles. Other melanoma samples were obtained from the Rennes University Hospital (CHU) tumor biobank (Centre de ressources biologiques humaines - CRB Santé). Staining was performed on a Discovery Automated IHC stainer (Roche) using a rabbit polyclonal anti-TRPV2 antibody (1:100, HPA044993, RRID:AB_10960889), or the same concentration of a control isotype, or the melanoma triple cocktail (HMB45+A103+T311, Ventana, Roche). Signal enhancement was performed using the Ventana ChromoMap Kit Slides (biotin free system). Sections were counterstained with hematoxylin and mounted with DPX. A detailed assessment was done by anatomopathologists. For the TMA, individual tumor regions were analyzed by color deconvolution using the Fast Red, Fast Blue, DAB filter (ImageJ, Appendix Fig S10D) based on (Stanisz *et al*, 2014). Specific TRPV2 stained regions were then subjected to densitometry analysis. Optical density was obtained using the formula: OD = Log(255/Mean). For immunostaining of metastasis from xenograft experiments, lungs were perfused with PBS, fixed in 4% paraformaldehyde, paraffin-embedded and sectioned. Sections of lungs (4 µm) were treated on a Discovery Automated IHC stainer (Roche) to remove paraffin, unmask epitopes and sequentially stain with rabbit polyclonal antibodies against TRPV2 (1:50; HPA044993 Sigma) or pmel17/HMB45 (1:50) and rat monoclonal antibody anti-mouse CD31 (1:200; clone SZ31) before detection with Alexa Fluor-conjugated secondary antibodies. Sections were mounted in Antifade Reagent with DAPI (Life Technologies).

Statistical analysis

All data are displayed as means ± SEM for $n \geq 3$ biological replicates. Statistical differences among cell lines or treatments were done by unpaired Student *t* tests or by ANOVA tests for multiple comparisons, as appropriate. Statistical analyses were performed in Prism 6.0 (GraphPad Prism, RRID:SCR_002798). Values with a P-value < 0.05 were considered statistically significant.

Data availability

The data generated in this study are available within the article and its supplementary data files. This study includes no data deposited in external repositories. Further information and requests for resources and reagents may be directed to and will be fulfilled by the corresponding author.

Expanded View for this article is available online.

© 2023 The Authors

Acknowledgements

We are grateful to BIOalternatives (FX Bernard) for providing normal human epidermal melanocytes, to Dr P-Y Rescan (LPGP-INRA, Rennes, France) for providing access to the zebrafish husbandry and technical guidance, to the IRSET Inserm U1085 SMS and DR@TE teams for housing and facility access, as well as to the Abbelight company (N. Bourg) and both the MRic and ImageUP microscopy platforms for critical assistance. We thank the TCGA Research Network. This work would not have been possible without Dr. FA Rassendren, Dr L Larue, Dr MD Galibert and Dr O Destaing help and critical discussions, and without Dr B Jégou's precious support. This work was funded by Roche/Groupe Cancérologie Cutanée de la Société Française de Dermatologie, the University of Rennes-1, Fondation ARC pour la Recherche sur le Cancer (PJA20151203419), La Ligue contre le cancer (Comités 35 & 86), the Région Nouvelle Aquitaine (Chaire Universitaire Canaux Calciques et Mélanome, AP; 16/RALPC-P-R-23), and the French Government (ANR, program #ANR-12-JSV2-0004-001). KFS was an ANR postdoctoral fellowship recipient. EB was supported by the Région Poitou-Charente.

Author contributions

Kenji F Shoji: Formal analysis; investigation; visualization; methodology; writing – original draft; writing – review and editing. **Elsa Bayet:** Formal analysis; investigation; visualization. **Sabrina Leverrier-Penna:** Formal analysis; investigation; visualization; methodology; writing – review and editing. **Dahiana Le Devedec:** Formal analysis; investigation. **Aude Mallavialle:** Formal analysis; investigation. **Severine Marionneau-Lambot:** Supervision; investigation; methodology; project administration. **Florian Rambow:** Formal analysis; investigation; visualization; methodology. **Raul Perret:** Investigation. **Aurelie Joussaume:** Investigation. **Roselyne Viel:** Investigation. **Alain Fautrel:** Resources; supervision; methodology. **Amir Khammari:** Supervision. **Bruno Constantin:** Supervision. **Sophie Tartare-Deckert:** Resources; supervision; writing – review and editing. **Aubin Penna:** Conceptualization; resources; formal analysis; supervision; funding acquisition; investigation; visualization; methodology; writing – original draft; project administration; writing – review and editing.

Disclosure and competing interests statement

AP and KFS are involved in a patent protecting the use of TRPV2 as a biomarker and as a therapeutic target for melanoma (WO2017064159A1). All other authors declare that they have no competing interests or disclosures.

References

Arozarena I, Wellbrock C (2017) Targeting invasive properties of melanoma cells. *FEBS J* 284: 2148 – 2162

Arozarena I, Bischof H, Gilby D, Belloni B, Dummer R, Wellbrock C (2011a) In melanoma, beta-catenin is a suppressor of invasion. *Oncogene* 30: 4531 – 4543

Arozarena I, Sanchez-Laorden B, Packer L, Hidalgo-Carcedo C, Hayward R, Viros A, Sahai E, Marais R (2011b) Oncogenic BRAF induces melanoma cell invasion by downregulating the cGMP-specific phosphodiesterase PDE5A. *Cancer Cell* 19: 45 – 57

Baljinnyam E, De Lorenzo MS, Xie LH, Iwatsubo M, Chen S, Goydos JS, Nowycky MC, Iwatsubo K (2010) Exchange protein directly activated by cyclic AMP increases melanoma cell migration by a Ca^{2+}–dependent mechanism. *Cancer Res* 70: 5607 – 5617

Barnhill JC, Stokes AJ, Koblan-Huberson M, Shimoda LM, Muraguchi A, Adra CN, Turner H (2004) RGA protein associates with a TRPV ion channel during biosynthesis and trafficking. *J Cell Biochem* 91: 808 – 820

Barretina J, Caponigro G, Stransky N, Venkatesan K, Margolin AA, Kim S, Wilson CJ, Lehar J, Kryukov GV, Sonkin D *et al* (2012) The cancer cell line encyclopedia enables predictive modelling of anticancer drug sensitivity. *Nature* 483: 603 – 607

Bruce JIE, James AD (2020) Targeting the calcium signalling machinery in cancer. *Cancers (Basel)* 12: 2351

Burridge K, Guilluy C (2016) Focal adhesions, stress fibers and mechanical tension. *Exp Cell Res* 343: 14 – 20

Canales Coutino B, Mayor R (2021) The mechanosensitive channel Piezo1 cooperates with semaphorins to control neural crest migration. *Development* 148: dev200001

Canales J, Morales D, Blanco C, Rivas J, Diaz N, Angelopoulos I, Cerda O (2019) A TR(i)P to cell migration: new roles of TRP channels in mechanotransduction and cancer. *Front Physiol* 10: 757

Cancer Genome Atlas Network (2015) Genomic classification of cutaneous melanoma. *Cell* 161: 1681 – 1696

Chantome A, Potier-Cartereau M, Clarysse L, Fromont G, Marionneau-Lambot S, Gueguinou M, Pages JC, Collin C, Oullier T, Girault A *et al* (2013) Pivotal role of the lipid raft SK3-Orai1 complex in human cancer cell migration and bone metastases. *Cancer Res* 73: 4852 – 4861

Chapman A, Fernandez del Ama L, Ferguson J, Kamarashev J, Wellbrock C, Hurlstone A (2014) Heterogeneous tumor subpopulations cooperate to drive invasion. *Cell Rep* 8: 688 – 695

Chen JP, Wang J, Luan Y, Wang CX, Li WH, Zhang JB, Sha D, Shen R, Cui YG, Zhang Z *et al* (2015) TRPM7 promotes the metastatic process in human nasopharyngeal carcinoma. *Cancer Lett* 356: 483 – 490

Chen J, Wu Y, Zhang L, Fang X, Hu X (2018) Evidence for calpains in cancer metastasis. *J Cell Physiol* 234: 8233 – 8240

Cohen MR, Johnson WM, Pilat JM, Kiselar J, DeFrancesco-Lisowitz A, Zigmond RE, Moiseenkova-Bell VY (2015) Nerve growth factor regulates transient receptor potential Vanilloid 2 via extracellular signal-regulated kinase signaling to enhance neurite outgrowth in developing neurons. *Mol Cell Biol* 35: 4238 – 4252

Elbaz M, Ahirwar D, Xiaoli Z, Zhou X, Lustberg M, Nasser MW, Shilo K, Ganju RK (2018) TRPV2 is a novel biomarker and therapeutic target in triple negative breast cancer. *Oncotarget* 9: 33459 – 33470

Fane ME, Chhabra Y, Smith AG, Sturm RA (2019) BRN2, a POUerful driver of melanoma phenotype switching and metastasis. *Pigment Cell Melanoma Res* 32: 9 – 24

Gabbireddy SR, Vosatka KW, Chung AJ, Logue JS (2021) Melanoma cells adopt features of both mesenchymal and amoeboid migration within confining channels. *Sci Rep* 11: 17804

Gambade A, Zreika S, Gueguinou M, Chourpa I, Fromont G, Bouchet AM, Burlaud-Gaillard J, Potier-Cartereau M, Roger S, Aucagne V *et al* (2016) Activation of TRPV2 and BKCa channels by the LL-37 enantiomers stimulates calcium entry and migration of cancer cells. *Oncotarget* 7: 23785 – 23800

Gao SL, Kong CZ, Zhang Z, Li ZL, Bi JB, Liu XK (2017) TRPM7 is overexpressed in bladder cancer and promotes proliferation, migration, invasion and tumor growth. *Oncol Rep* 38: 1967 – 1976

Gardel ML, Schneider IC, Aratyn-Schaus Y, Waterman CM (2010) Mechanical integration of actin and adhesion dynamics in cell migration. *Annu Rev Cell Dev Biol* 26: 315 – 333

Glading A, Bodnar RJ, Reynolds IJ, Shiraha H, Satish L, Potter DA, Blair HC, Wells A (2004) Epidermal growth factor activates m-calpain (calpain II), at least in part, by extracellular signal-regulated kinase-mediated phosphorylation. *Mol Cell Biol* 24: 2499 – 2512

Guilbert A, Gautier M, Dhennin-Duthille I, Haren N, Sevestre H, Ouadid-Ahidouch H (2009) Evidence that TRPM7 is required for breast cancer cell proliferation. *Am J Physiol Cell Physiol* 297: C493 – C502

© 2023 The Authors

Guilbert A, Gautier M, Dhennin-Duthille I, Rybarczyk P, Sahni J, Sevestre H, Scharenberg AM, Ouadid-Ahidouch H (2013) Transient receptor potential melastatin 7 is involved in oestrogen receptor-negative metastatic breast cancer cells migration through its kinase domain. *Eur J Cancer* 49: 3694 – 3707

Haining AW, Lieberthal TJ, Del Rio Hernandez A (2016) Talin: a mechanosensitive molecule in health and disease. *FASEB J* 30: 2073 – 2085

Herlyn D, Iliopoulos D, Jensen PJ, Parmiter A, Baird J, Hotta H, Adachi K, Ross AH, Jambrosic J, Koprowski H *et al* (1990) *In vitro* properties of human melanoma cells metastatic in nude mice. *Cancer Res* 50: 2296 – 2302

Hung WC, Yang JR, Yankaskas CL, Wong BS, Wu PH, Pardo-Pastor C, Serra SA, Chiang MJ, Gu Z, Wirtz D *et al* (2016) Confinement sensing and signal optimization via Piezo1/PKA and myosin II pathways. *Cell Rep* 15: 1430 – 1441

Jiu Y, Lehtimaki J, Tojkander S, Cheng F, Jaalinoja H, Liu X, Varjosalo M, Eriksson JE, Lappalainen P (2015) Bidirectional interplay between vimentin intermediate filaments and contractile actin stress fibers. *Cell Rep* 11: 1511 – 1518

Ju RJ, Stehbens SJ, Haass NK (2018) The role of melanoma cell-stroma interaction in cell motility, invasion, and metastasis. *Front Med (Lausanne)* 5: 307

Juhasz I, Albelda SM, Elder DE, Murphy GF, Adachi K, Herlyn D, Valyi-Nagy IT, Herlyn M (1993) Growth and invasion of human melanomas in human skin grafted to immunodeficient mice. *Am J Pathol* 143: 528 – 537

Kabbarah O, Nogueira C, Feng B, Nazarian RM, Bosenberg M, Wu M, Scott KL, Kwong LN, Xiao Y, Cordon-Cardo C *et al* (2010) Integrative genome comparison of primary and metastatic melanomas. *PLoS One* 5: e10770

Karki T, Tojkander S (2021) TRPV protein family-from mechanosensing to cancer invasion. *Biomolecules* 11: 1019

Katanosaka K, Takatsu S, Mizumura K, Naruse K, Katanosaka Y (2018) TRPV2 is required for mechanical nociception and the stretch-evoked response of primary sensory neurons. *Sci Rep* 8: 16782

Kojima I, Nagasawa M (2014) Trpv2. *Handb Exp Pharmacol* 222: 247 – 272

Kozar I, Margue C, Rothengatter S, Haan C, Kreis S (2019) Many ways to resistance: how melanoma cells evade targeted therapies. *Biochim Biophys Acta Rev Cancer* 1871: 313 – 322

Kudou M, Shiozaki A, Yamazato Y, Katsurahara K, Kosuga T, Shoda K, Arita T, Konishi H, Komatsu S, Kubota T *et al* (2019) The expression and role of TRPV2 in esophageal squamous cell carcinoma. *Sci Rep* 9: 16055

Kwong LN, Kabbarah O, Nogueira C, Wagner SN, Chin L (2013) Human melanoma samples comparing nevi and primary and metastatic melanoma. Gene Expression Omnibus GSE46517 (https://www.ncbi.nlm.nih.gov/geo/query/acc.cgi?acc=GSE46517). [DATASET]

Legerstee K, Geverts B, Slotman JA, Houtsmuller AB (2019) Dynamics and distribution of paxillin, vinculin, zyxin and VASP depend on focal adhesion location and orientation. *Sci Rep* 9: 10460

Lehtimaki J, Hakala M, Lappalainen P (2017) Actin filament structures in migrating cells. *Handb Exp Pharmacol* 235: 123 – 152

Leverrier-Penna S, Destaing O, Penna A (2020) Insights and perspectives on calcium channel functions in the cockpit of cancerous space invaders. *Cell Calcium* 90: 102251

Liu Q, Wang X (2013) Effect of TRPV2 cation channels on the proliferation, migration and invasion of 5637 bladder cancer cells. *Exp Ther Med* 6: 1277 – 1282

Liu CY, Lin HH, Tang MJ, Wang YK (2015a) Vimentin contributes to epithelial-mesenchymal transition cancer cell mechanics by mediating cytoskeletal organization and focal adhesion maturation. *Oncotarget* 6: 15966 – 15983

Liu YJ, Le Berre M, Lautenschlaeger F, Maiuri P, Callan-Jones A, Heuze M, Takaki T, Voituriez R, Piel M (2015b) Confinement and low adhesion induce fast amoeboid migration of slow mesenchymal cells. *Cell* 160: 659 – 672

Macia A, Herreros J, Marti RM, Canti C (2015) Calcium channel expression and applicability as targeted therapies in melanoma. *Biomed Res Int* 2015: 587135

McNeill MS, Paulsen J, Bonde G, Burnight E, Hsu MY, Cornell RA (2007) Cell death of melanophores in zebrafish trpm7 mutant embryos depends on melanin synthesis. *J Invest Dermatol* 127: 2020 – 2030

Middelbeek J, Kuipers AJ, Henneman L, Visser D, Eidhof I, van Horssen R, Wieringa B, Canisius SV, Zwart W, Wessels LF *et al* (2012) TRPM7 is required for breast tumor cell metastasis. *Cancer Res* 72: 4250 – 4261

Mignen O, Constantin B, Potier-Cartereau M, Penna A, Gautier M, Gueguinou M, Renaudineau Y, Shoji KF, Felix R, Bayet E *et al* (2017) Constitutive calcium entry and cancer: updated views and insights. *Eur Biophys J* 46: 395 – 413

Monet M, Gkika D, Lehen'kyi V, Pourtier A, Vanden Abeele F, Bidaux G, Juvin V, Rassendren F, Humez S, Prevarsakaya N (2009) Lysophospholipids stimulate prostate cancer cell migration via TRPV2 channel activation. *Biochim Biophys Acta* 1793: 528 – 539

Monet M, Lehen'kyi V, Gackiere F, Firlej V, Vandenberghe M, Roudbaraki M, Gkika D, Pourtier A, Bidaux G, Slomianny C *et al* (2010) Role of cationic channel TRPV2 in promoting prostate cancer migration and progression to androgen resistance. *Cancer Res* 70: 1225 – 1235

Nagasawa M, Kojima I (2015) Translocation of TRPV2 channel induced by focal administration of mechanical stress. *Physiol Rep* 3: e12296

Oancea E, Vriens J, Brauchi S, Jun J, Splawski I, Clapham DE (2009) TRPM1 forms ion channels associated with melanin content in melanocytes. *Sci Signal* 2: ra21

Oulidi A, Bokhobza A, Gkika D, Vanden Abeele F, Lehen'kyi V, Ouafik L, Mauroy B, Prevarskaya N (2013) TRPV2 mediates adrenomedullin stimulation of prostate and urothelial cancer cell adhesion, migration and invasion. *PLoS One* 8: e64885

Pedri D, Karras P, Landeloos E, Marine JC, Rambow F (2021) Epithelial-to-mesenchymal-like transition events in melanoma. *FEBS J* 289: 1352 – 1368

Penna A, Cahalan M (2007) Western blotting using the Invitrogen NuPage Novex Bis Tris minigels. *J Vis Exp* 264

Penna A, Juvin V, Chemin J, Compan V, Monet M, Rassendren FA (2006) PI3-kinase promotes TRPV2 activity independently of channel translocation to the plasma membrane. *Cell Calcium* 39: 495 – 507

Penna A, Demuro A, Yeromin AV, Zhang SL, Safrina O, Parker I, Cahalan MD (2008) The CRAC channel consists of a tetramer formed by Stim-induced dimerization of Orai dimers. *Nature* 456: 116 – 120

Qin N, Neeper MP, Liu Y, Hutchinson TL, Lubin ML, Flores CM (2008) TRPV2 is activated by cannabidiol and mediates CGRP release in cultured rat dorsal root ganglion neurons. *J Neurosci* 28: 6231 – 6238

Rambow F, Marine JC, Goding CR (2019) Melanoma plasticity and phenotypic diversity: therapeutic barriers and opportunities. *Genes Dev* 33: 1295 – 1318

Reinhold WC, Sunshine M, Liu H, Varma S, Kohn KW, Morris J, Doroshow J, Pommier Y (2012) CellMiner: a web-based suite of genomic and pharmacologic tools to explore transcript and drug patterns in the NCI-60 cell line set. *Cancer Res* 72: 3499 – 3511

Ridley AJ, Schwartz MA, Burridge K, Firtel RA, Ginsberg MH, Borisy G, Parsons JT, Horwitz AR (2003) Cell migration: integrating signals from front to back. *Science* 302: 1704 – 1709

Robertson J, Jacquemet G, Byron A, Jones MC, Warwood S, Selley JN, Knight D, Humphries JD, Humphries MJ (2015) Defining the phospho-adhesome

 © 2023 The Authors

through the phosphoproteomic analysis of integrin signalling. *Nat Commun* 6: 6265

Rowling EJ, Miskolczi Z, Nagaraju R, Wilcock DJ, Wang P, Telfer B, Li Y, Lasheras-Otero I, Redondo-Munoz M, Sharrocks AD *et al* (2020) Cooperative behaviour and phenotype plasticity evolve during melanoma progression. *Pigment Cell Melanoma Res* 33: 695 – 708

Rybarczyk P, Vanlaeys A, Brassart B, Dhennin-Duthille I, Chatelain D, Sevestre H, Ouadid-Ahidouch H, Gautier M (2017) The transient receptor potential melastatin 7 channel regulates pancreatic cancer cell invasion through the Hsp90alpha/uPA/MMP2 pathway. *Neoplasia* 19: 288 – 300

Santoni G, Farfariello V, Liberati S, Morelli MB, Nabissi M, Santoni M, Amantini C (2013) The role of transient receptor potential vanilloid type-2 ion channels in innate and adaptive immune responses. *Front Immunol* 4: 34

Santoni G, Amantini C, Maggi F, Marinelli O, Santoni M, Nabissi M, Morelli MB (2020) The TRPV2 cation channels: from urothelial cancer invasiveness to glioblastoma multiforme interactome signature. *Lab Invest* 100: 186 – 198

Schumacher S, Vazquez Nunez R, Biertumpfel C, Mizuno N (2021) Bottom-up reconstitution of focal adhesion complexes. *FEBS J* 289: 3360 – 3373

Schwab A, Fabian A, Hanley PJ, Stock C (2012) Role of ion channels and transporters in cell migration. *Physiol Rev* 92: 1865 – 1913

Sharma P, Hu-Lieskovan S, Wargo JA, Ribas A (2017) Primary, adaptive, and acquired resistance to cancer immunotherapy. *Cell* 168: 707 – 723

Shibasaki K, Murayama N, Ono K, Ishizaki Y, Tominaga M (2010) TRPV2 enhances axon outgrowth through its activation by membrane stretch in developing sensory and motor neurons. *J Neurosci* 30: 4601 – 4612

Siegel RL, Miller KD, Jemal A (2018) Cancer statistics, 2018. *CA: Cancer J Cli* 68: 7 – 30

Siveen KS, Nizamuddin PB, Uddin S, Al-Thani M, Frenneaux MP, Janahi IA, Steinhoff M, Azizi F (2020) TRPV2: a cancer biomarker and potential therapeutic target. *Dis Markers* 2020: 8892312

Spinsanti G, Zannolli R, Panti C, Ceccarelli I, Marsili L, Bachiocco V, Frati F, Aloisi AM (2008) Quantitative Real-Time PCR detection of TRPV1-4 gene expression in human leukocytes from healthy and hyposensitive subjects. *Mol Pain* 4: 51

Stanisz H, Saul S, Muller CS, Kappl R, Niemeyer BA, Vogt T, Hoth M, Roesch A, Bogeski I (2014) Inverse regulation of melanoma growth and migration by Orai1/STIM2-dependent calcium entry. *Pigment Cell Melanoma Res* 27: 442 – 453

Stokes AJ, Shimoda LM, Koblan-Huberson M, Adra CN, Turner H (2004) A TRPV2-PKA signaling module for transduction of physical stimuli in mast cells. *J Exp Med* 200: 137 – 147

Strouhalova K, Prechova M, Gandalovicova A, Brabek J, Gregor M, Rosel D (2020) Vimentin intermediate filaments as potential target for cancer treatment. *Cancers (Basel)* 12: 184

Sugio S, Nagasawa M, Kojima I, Ishizaki Y, Shibasaki K (2017) Transient receptor potential vanilloid 2 activation by focal mechanical stimulation requires interaction with the actin cytoskeleton and enhances growth cone motility. *FASEB J* 31: 1368 – 1381

Tajada S, Villalobos C (2020) Calcium permeable channels in cancer hallmarks. *Front Pharmacol* 11: 968

Tang Z, Li C, Kang B, Gao G, Li C, Zhang Z (2017) GEPIA: a web server for cancer and normal gene expression profiling and interactive analyses. *Nucleic Acids Res* 45: W98 – W102

te Boekhorst V, Jiang L, Mählen M, Meerlo M, Dunkel G, Durst FC, Yang Y, Levine H, Burgering BMT, Friedl P (2020) Calpain-2 regulates hypoxia/HIF-induced amoeboid reprogramming and metastasis. *bioRxiv* https://doi.org/10.1101/2020.01.06.892497 [PREPRINT]

Teng Y, Xie X, Walker S, White DT, Mumm JS, Cowell JK (2013) Evaluating human cancer cell metastasis in zebrafish. *BMC Cancer* 13: 453

Tichet M, Prod'Homme V, Fenouille N, Ambrosetti D, Mallavialle A, Cerezo M, Ohanna M, Audebert S, Rocchi S, Giacchero D *et al* (2015) Tumour-derived SPARC drives vascular permeability and extravasation through endothelial VCAM1 signalling to promote metastasis. *Nat Commun* 6: 6993

Tuncer E, Calcada RR, Zingg D, Varum S, Cheng P, Freiberger SN, Deng CX, Kleiter I, Levesque MP, Dummer R *et al* (2019) SMAD signaling promotes melanoma metastasis independently of phenotype switching. *J Clin Invest* 129: 2702 – 2716

Van den Eynde C, De Clercq K, Vriens J (2021) Transient receptor potential channels in the epithelial-to-mesenchymal transition. *Int J Mol Sci* 22: 8188

Wei C, Wang X, Zheng M, Cheng H (2012) Calcium gradients underlying cell migration. *Curr Opin Cell Biol* 24: 254 – 261

Yao M, Tijore A, Cheng D, Li JV, Hariharan A, Martinac B, Tran Van Nhieu G, Cox CD, Sheetz M (2022) Force- and cell state-dependent recruitment of Piezo1 drives focal adhesion dynamics and calcium entry. *Sci Adv* 8: eabo1461

Yee NS, Kazi AA, Li Q, Yang Z, Berg A, Yee RK (2015) Aberrant over-expression of TRPM7 ion channels in pancreatic cancer: required for cancer cell invasion and implicated in tumor growth and metastasis. *Biol Open* 4: 507 – 514

Zhang S, Cao S, Gong M, Zhang W, Zhang W, Zhu Z, Wu S, Yue Y, Qian W, Ma Q *et al* (2022) Mechanically activated ion channel Piezo1 contributes to melanoma malignant progression through AKT/mTOR signaling. *Cancer Biol Ther* 23: 336 – 347

License: This is an open access article under the terms of the Creative Commons Attribution License, which permits use, distribution and reproduction in any medium, provided the original work is properly cited.

Expanded View Figures

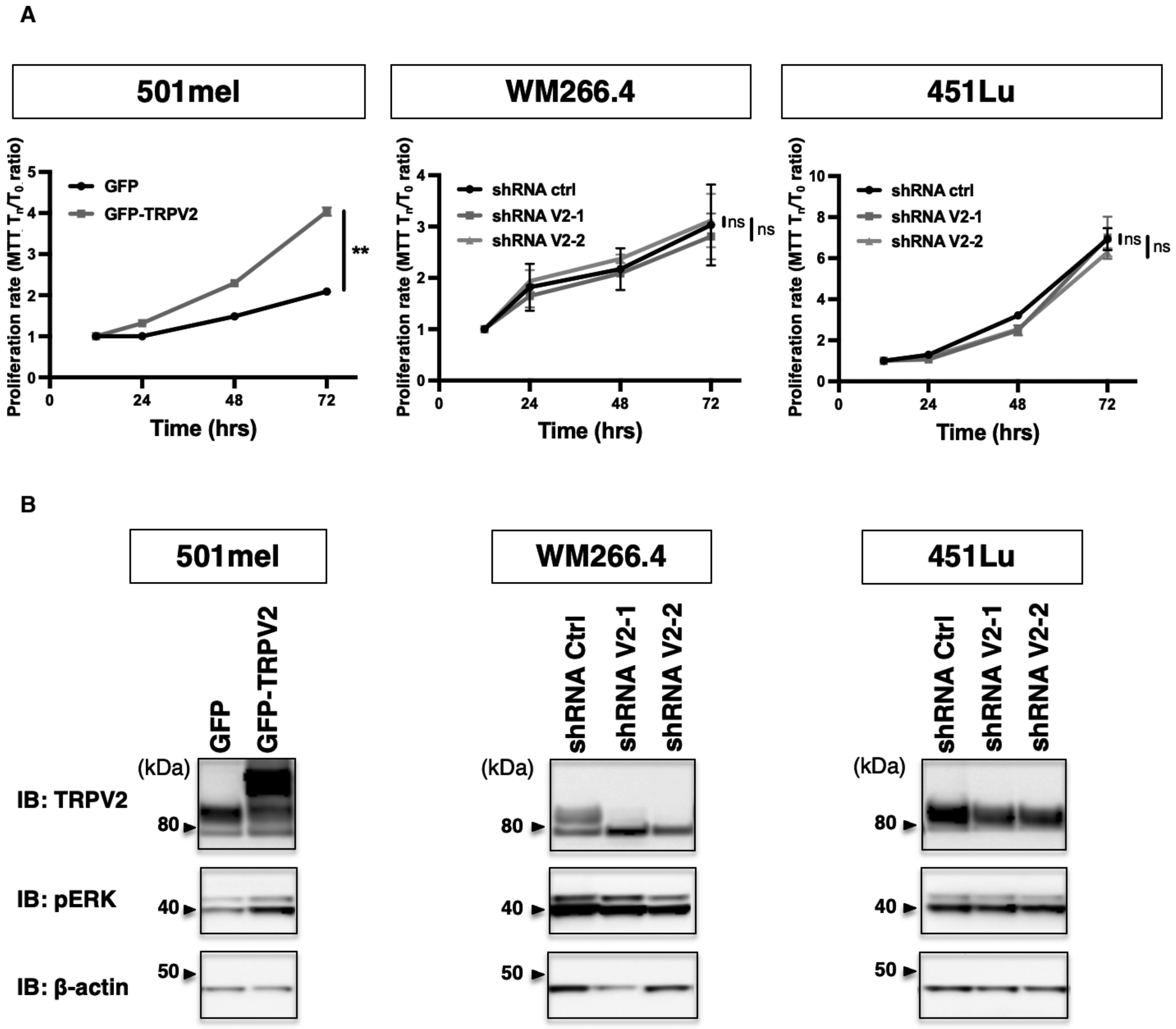

Figure EV1. TRPV2 is dispensable for melanoma tumor cell proliferation (relative to Fig 3).

A Representative proliferation curves comparing the effect of TRPV2 overexpression in 501mel cells, or TRPV2 repression in WM266.4 and 451Lu cells, measured by MTT at 12, 24, 48, or 72 h. Each data point represents the mean ± SEM of $n = 3$ biological replicates with two-way ANOVA multiple comparisons test results for the 72 h time points (**$P = 0.0038$ for 501mel GFP vs. 501mel GFP-TRPV2; ns $P = 0.8422$ for WM266.4 shRNA ctrl vs. ShRNA V2-1, ns $P = 0.9491$ for WM266.4 shRNA ctrl vs. ShRNA V2-2, ns $P = 0.9982$ for 451Lu shRNA ctrl vs. ShRNA V2-1, ns $P = 0.5755$ for 451Lu shRNA ctrl vs. ShRNA V2-2).

B Immunoblotting (IB) of TRPV2, Thr202/Tyr204 Phospho-p44/42 MAPK (pERK), and the β-actin as a loading control in melanoma cell lines modified for TRPV2 expression.

Source data are available online for this figure.

 © 2023 The Authors

Figure EV2. TRPV2 promotes melanoma cell migration through the recruitment of Calpain activity (relative to Fig 5).

A Representative gap closure kinetics from wound-healing assays performed on 501mel cells control (GFP) and 501mel cells overexpressing TRPV2 (GFP-TRPV2) treated either with vehicle, 10 μM calpeptin or 50 μM PD150606. Each data point represents the mean ± SEM of $n = 4$ technical replicates.

B Quantification of the impact of calpain pharmacological inhibition on TRPV2-dependent cell migration. Cell migration speed was assessed as the slope of gap closure kinetics measured for the first 24 h of the wound-healing assays to avoid potential proliferation-induced effects. Histogram illustrates migration speed normalized to 501mel GFP control cells presented as mean ± SEM of $n = 4$ biological replicates with statistical analysis performed using the Kruskal–Wallis test followed by Dunn's multiple comparisons tests (*$P < 0.05$; **$P < 0.01$; ***$P < 0.001$, ns = nonsignificant; See Appendix Table S3 for exact *P*-values).

C Representative cell migration kinetics from xCELLigence CIM-plate assays performed on GFP-transfected shRNA Ctrl WM266.4 cells, GFP-transfected shRNA V2 WM266.4 cells and shRNA V2 WM266.4 cells transfected with constitutively active Calpain-2 (GFP-CAPN2^{S50E}). Each data point represents the mean ± SD of the Cell Index—a unit automatically computerized by the xCELLigence software—resulting from technical replicates in two independent wells recorded in parallel from the same CIM-plate. Here, we did not use wound-healing assays due to the specific context of transient transfection and because active calpain expression modifies cell adhesion properties making it difficult to obtain nice cell monolayers with delimited wounds.

D Quantification of the ability of active calpain forced expression to rescue the cell migration defect induced by TRPV2 silencing. Cell migration speed was assessed as the slope of the Cell index during the exponential phase. Histogram illustrates cell migration normalized to WM266.4 shRNA Ctrl cells presented as mean ± SEM of $n = 3$ biological replicates with statistical analysis performed using the Kruskal–Wallis test followed by Dunn's multiple comparisons test (***$P = 0.0004$ and ns $P = 0.1484$).

Source data are available online for this figure.

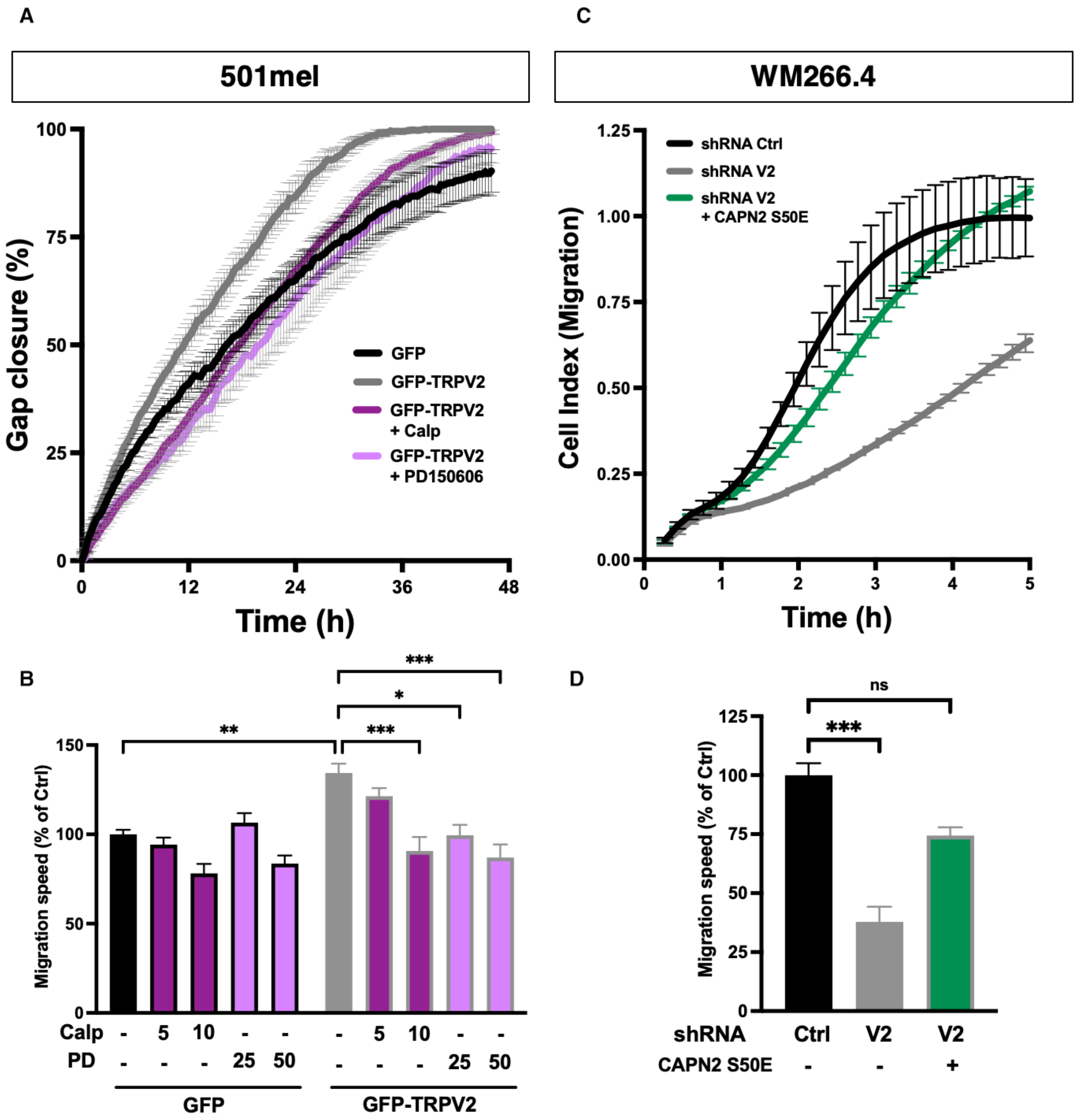

Figure EV2.

 © 2023 The Authors

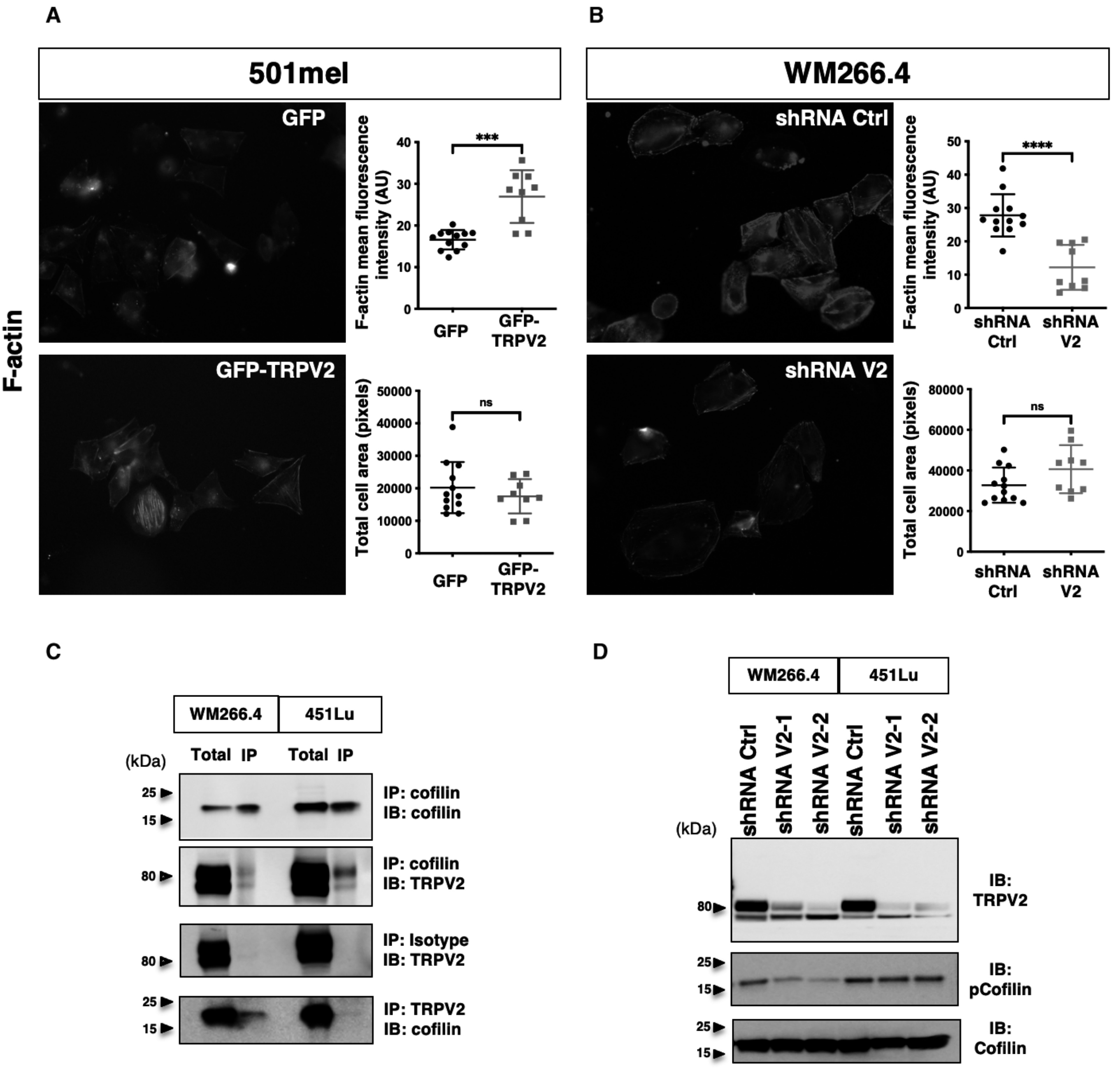

Figure EV3. TRPV2 Regulates Actin cytoskeleton remodeling and cofilin activation.

A, B Fluorescent microscopy images showing live F-actin polymerization in control (GFP) or GFP-TRPV2 overexpressing 501mel cells (A), and in control (Ctrl) or TRPV2-targeting shRNAs transduced WM266.4 cells (B). Quantification of F-actin fluorescent intensity (top plot) and cell area (bottom plot) in both populations. Data are presented as scatter dot plots with mean ± SD, each dot representing mean F-actin fluorescence intensity or mean area per cell in n = 13–16 fields from three independent experiments (***P = 0.0005 and ns P = 0.7021 for 501mel, ****P < 0.0001 and ns P = 0.1308 for WM266.4, the Mann–Whitney test).

C Reverse co-immunoprecipitation (IP) experiments showing a physical interaction between TRPV2 and cofilin in the WM266.4 and 451Lu metastatic melanoma cell lines. Co-IP experiments were performed three times and the immunoblots (IB) show typical results.

D Immunoblot assessment of cofilin activation by measuring total and Ser3-phosphorylated cofilin levels in control (shRNA Ctrl) and TRPV2-silenced (shRNA V2) WM266.4 and 451Lu cells.

Source data are available online for this figure.

Figure EV4. TRPV2 overexpression promotes widespread metastatic colonization of melanoma tumor cells (relative to Fig 6).

A *Ex vivo* BLI measurements for each necropsy collected organ of representative mice xenografted with either GFP control or GFP-TRPV2 overexpressing 501mel-LUC cells.

B Box and whiskers plot shows the number of metastatic foci per organ counted at necropsy of mice xenografted with GFP (black) or GFP-TRPV2 (gray) overexpressing 501mel-LUC cells. Boxes extend from the 25th to 75th percentiles, whiskers from min to max, and each dot correspond to a single mouse (n = 6–7).

Source data are available online for this figure.

 © 2023 The Authors

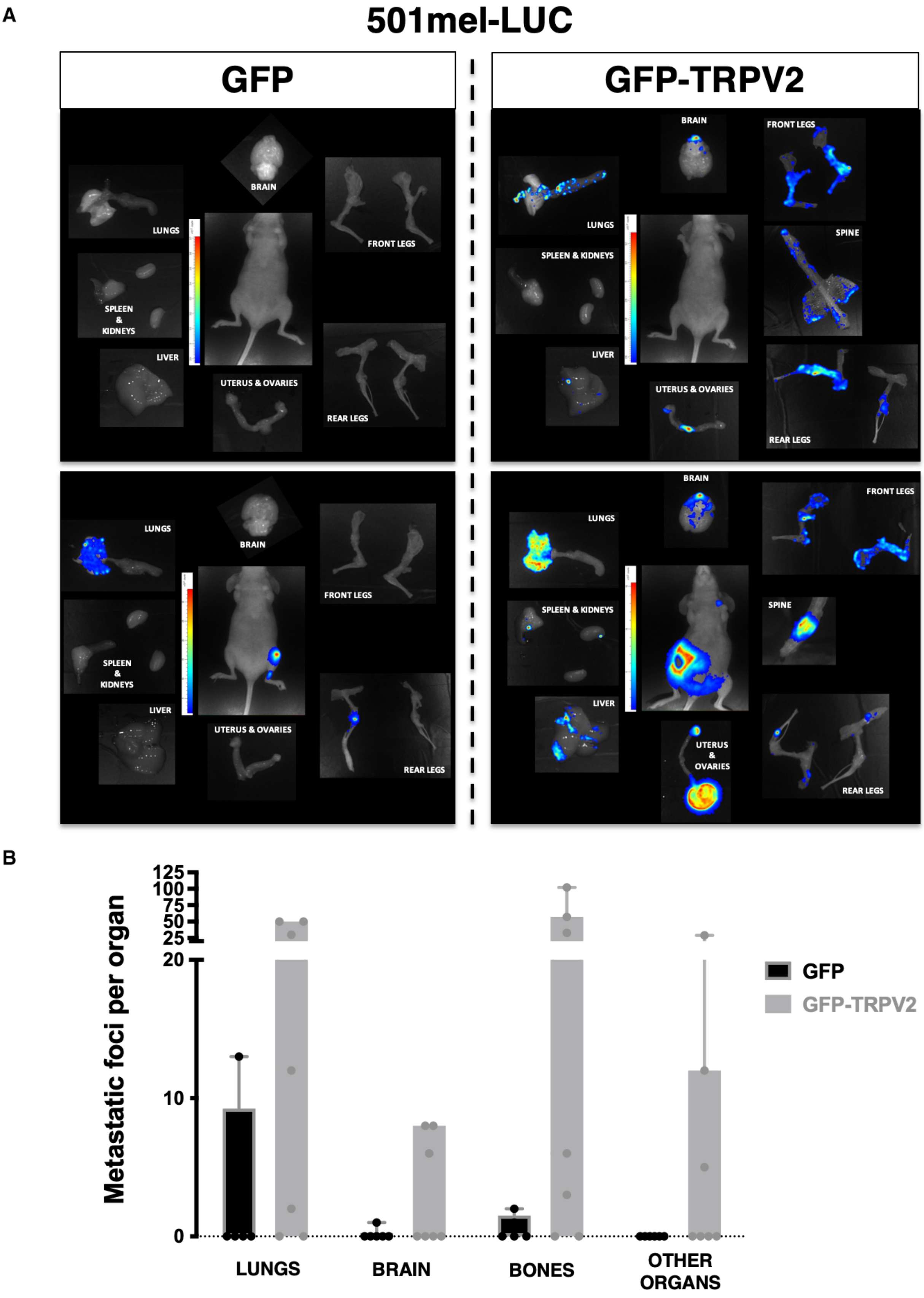
A
501mel-LUC
GFP
GFP-TRPV2
BRAIN
LUNGS
FRONT LEGS
SPLEEN & KIDNEYS
LIVER
UTERUS & OVARIES
REAR LEGS
SPINE
B
Metastatic foci per organ
125
100
75
50
25
20
10
0
LUNGS
BRAIN
BONES
OTHER ORGANS
GFP
GFP-TRPV2

Figure EV4.

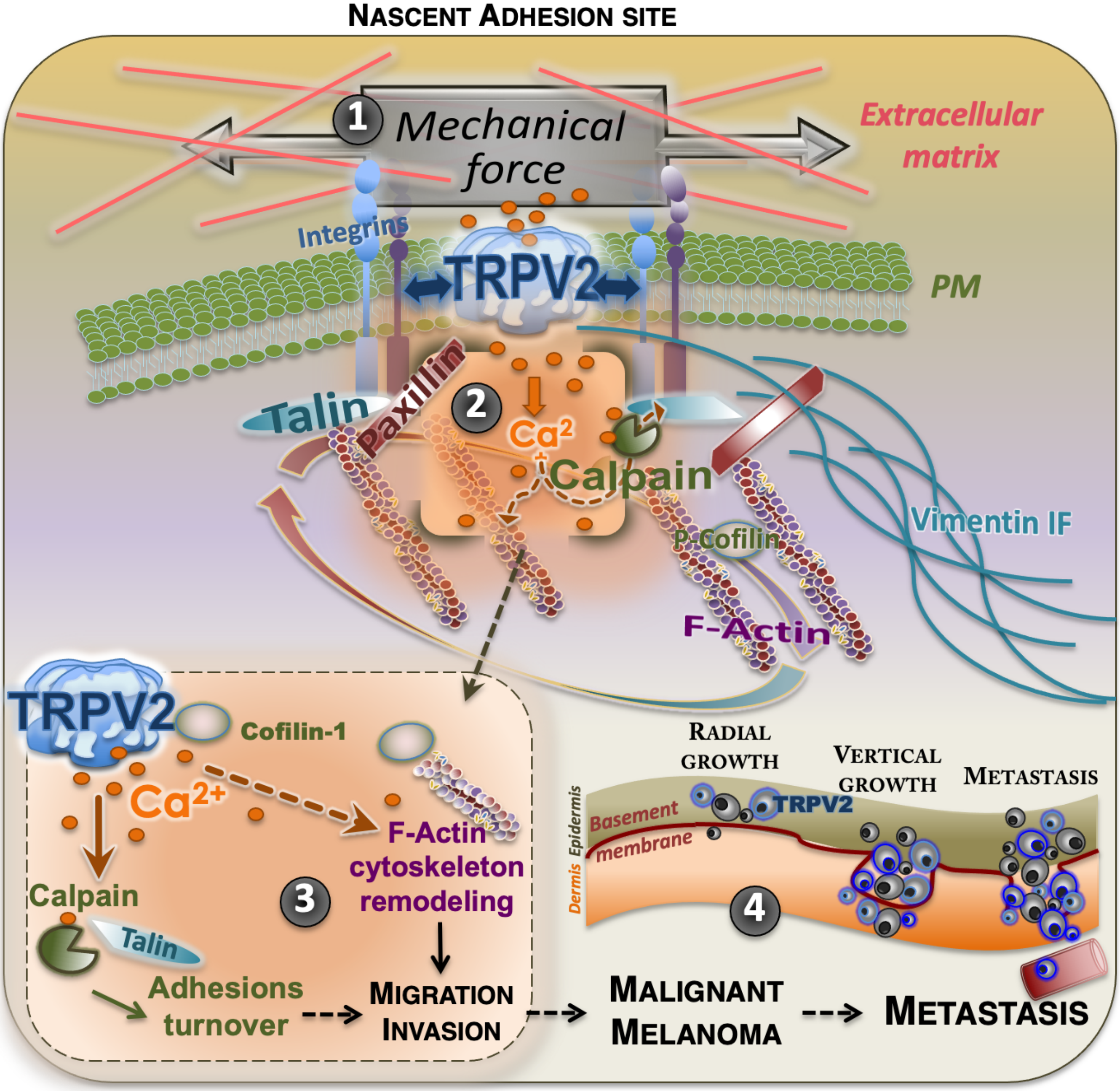

Figure EV5. Mechanistic model of TRPV2 control over metastatic melanoma cells dissemination through the dynamic regulation of nascent adhesion sites.

The essential role of TRPV2 in melanoma migration and invasion could be explained by the newly identified pro-invasive properties of this mechanosensitive channel. Indeed, cancer cells and their associated microenvironment generate considerable mechanical forces applied onto the plasma membrane (PM) (1). These changes in PM tension regulate cell shape and movement. In malignant melanoma cells, the TRPV2 channel is recruited to the PM within paxillin-containing early adhesion structures, and its constitutive activation elicits a subplasmalemmal localized Ca^{2+} ions uptake (2). TRPV2-mediated Ca^{2+} influx triggers the activation of the intracellular Ca^{2+}-dependent cysteine protease, calpain (3). The cleavage of its substrate, the early adhesion protein talin linking membrane integrins and cytoskeleton, *in fine* prompts the disassembly of a subset of adhesion complexes and facilitates cell-extracellular matrix (ECM) contact sites plasticity. Induction, selection, and maturation of nascent adhesion complexes at the cell leading edge serve as sampling the local ECM to select traction points producing forces that will drive the cell body forward. To further regulate the maturation of these adhesion structures along with the remodeling of the cytoskeleton, TRPV2 directly interacts with both the intermediate filament (IF) vimentin network, and the actin severing factor cofilin-1, a central regulator of F-actin dynamics. TRPV2-induced signaling promotes the spatial and temporal accumulation of F-actin bundles to improve advanced melanoma cell motility. Therefore, TRPV2 channel-mediated Ca^{2+} influx tunes the plasticity of the melanoma tumor cell by locally controlling adhesion complexes maturation and cytoskeleton remodeling, potentiating the migratory and invasive behaviors of these malignant cells (4).

 © 2023 The Authors

Appendix for "The mechanosensitive TRPV2 calcium channel promotes human melanoma invasiveness and metastatic potential"by Kenji F. Shoji *et al.*

Table of content

**Appendix Figure S1 (related to Figure 1).**	**p.1**
**Appendix Figure S2 (related to Figure 2).**	**p.3**
**Appendix Figure S3 (related to Figure 3).**	**p.4**
**Appendix Figure S4.**	**p.5**
**Appendix Figure S5 (related to Figure 4).**	**p.6**
**Appendix Figure S6 (related to Figure 5).**	**p.7**
**Appendix Figure S7 (related to Figure 6).**	**p.9**
**Appendix Figure S8 (related to Figure 6).**	**p.10**
**Appendix Figure S9 (related to Figure 6).**	**p.12**
**Appendix Figure S10 (related to Figure 7).**	**p.13**
**Appendix Figure S11 (related to Figure 7).**	**p.14**
**Appendix Table S1.**	**p.15**
**Appendix Table S2.**	**p.16**
**Appendix Table S3.**	**p.16**

Appendix Figure S1 (related to Figure 1). TRPV2 is predominantly expressed in melanoma tumors as compared to nevi tissues or to cancers from other origin

(A) TRPV2 mRNA expression analysis within the Broad-Novartis Cancer Cell Line Encyclopedia (CCLE) project RNAseq data. The box plot is sorted and colored by the average distribution of TRPV2 expression in a lineage (The highest average distribution is in red on the left and the red arrow points to the melanoma lineage). Lineages are composed of cell lines from the same area/organ or system of the body with the number of corresponding cell lines indicated in the figure. Data is represented as whisker-box plots with outliers plotted as individual points (Boxes extend from the 25th to 75th percentiles, whiskers from the 10th to 90th percentiles, the solid horizontal line in each box is plotted at the median and the dashed one at the mean).

(B) GEPIA (Gene expression profiling interactive analysis) generated dot plot profiling of TRPV2 gene expression across multiple cancer types and paired normal samples (TCGA tumors *vs* TCGA normal) based on TCGA RNAseq data directly extracted from tissue samples. Cancer types are as follow (T, tumor sample; N, matched normal sample): ACC, Adrenocortical carcinoma (T n=77; N n=-); BLCA, Bladder carcinoma (T n=404; N n=19); BRCA, Breast invasive carcinoma (T n=1085; N n=112); CESC, Cervical squamous cell carcinoma and endocervical adenocarcinoma (T n=306; N n=3); CHOL, Cholangiocarcinoma (T n=36; N n=9); COAD, Colon adenocarcinoma (T n=275; N n=41); DLBC, Diffuse Large B-cell Lymphoma (T n=47; N n=-); ESCA, Esophageal carcinoma (T n=182; N n=13); GBM, Glioblastoma multiform (T n=163; N n=-); HNSC, Head and neck squamous cell carcinoma (T n=519; N n=44); KICH, Kidney chromophobe (T n=66; N n=25); KIRC, Kidney renal clear cell carcinoma (T n=523; N n=72); KIRP, Kidney renal papillary cell carcinoma (T n=286; N n=32); LAML, Acute Myeloid Leukemia (T n=173; N n=-); LGG, Lower grade glioma (T n=518; N n=207); LIHC, Liver hepatocellular carcinoma (T n=369; N n=50); LUAD, Lung adenocarcinoma (T n=483; N n=59); LUSC, Lung squamous cell carcinoma (T n=486; N n=50); MESO, Mesothelioma (T n=87; N n=-); OV, Ovarian serous cystadenocarcinoma (T n=426; N n=-); PAAD, Pancreatic adenocarcinoma (T n=179; N n=4); PCPG, Pheochromocytoma and Paraganglioma (T n=182; N n=3); PRAD, Prostate adenocarcinoma (T n=492; N n=52); READ, Rectum adenocarcinoma (T n=92; N n=10); SARC, Sarcoma (T n=262; N n=2); SKCM, Skin cutaneous melanoma (T n=461; N n=1); STAD, Stomach adenocarcinoma (T n=408; N n=36); TGCT, Testicular germ cell tumor (T n=137; N n=-); THCA, Thyroid carcinoma (T n=512; N n=59); THYM, Thymoma (T n=118; N n=2); UCEC, Uterine corpus endometrial carcinoma (T n=174; N n=13); UCS, Uterine carcinosarcoma (T n=57; N n=-); UVM; Uveal melanoma (T n=79; N n=-).

(C) TCGA_SKCM based GEPIA2 generated dot plot profiling of TRPV2 gene expression across the main melanoma mutational status: BRAF_Hotspot_Mutants (n=149), NF1_Any_Mutants (n=27), RAS_Hotspot_Mutants (n=92), Triple_WT (n=45).

For B and C the box plot shows TRPV2 expression levels in different cancers and normal tissues or melanoma subtypes as whisker-box plots with outliers plotted as individual points (Boxes extend from the 25th to 75th percentiles, whiskers from the 5th to 95th percentiles, the solid horizontal line in each box is plotted at the median).

(D-E) TRPV2 expression was correlated with POU3F2(BRN2) levels, based on mRNA data presented in figure 1G for the cell lines used in this study (E) or based on publicly available expression data for an

extended panel of 83 melanoma cell lines from the CCLE cohort (F).

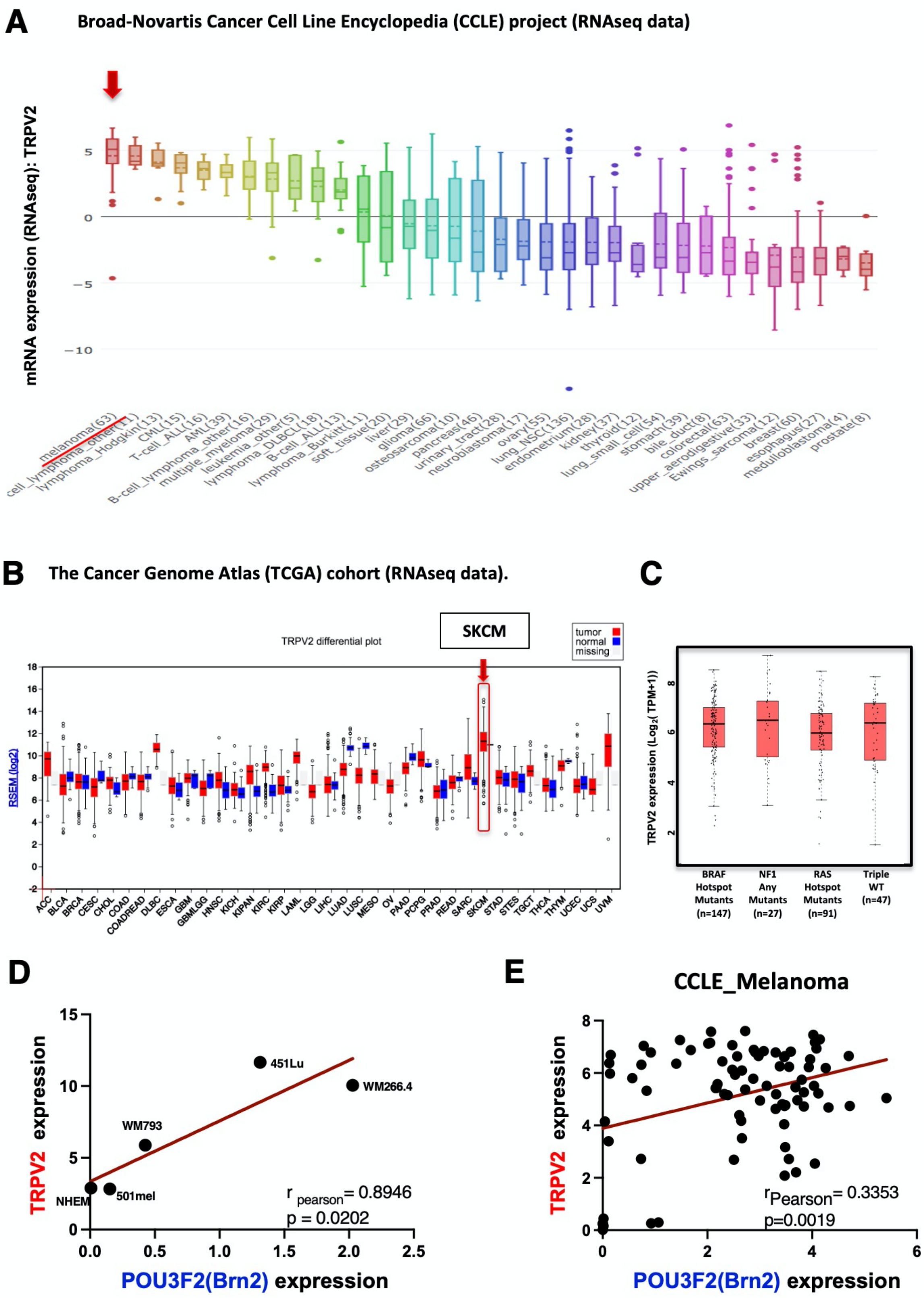

Appendix Figure S2 (related to Figure 2). TRPV2 functional expression and plasma membrane localization correlate with the invasive phenotype of melanoma cells

(A) Representatives Ca^{2+} influx responses to 40 µM Cannabidiol (CBD) stimulation. Data represents means ± SD from at least 15 cells per cell line.

(B) Confocal microphotographs showing the distribution of TRPV2 (red) and stained nuclei (blue) in the indicated melanoma cell lines. The dotted line highlights the membrane edge in the WM793 cell line. Note that TRPV2 distribution at the plasma membrane correlates with the invasive potential of the melanoma cell lines. (Scale bar = 10µm).

(C) Representative kinetics of CBD induced Ca^{2+} influx in melanoma cell lines modified for TRPV2 expression. Non-invasive 501mel cells (left) overexpressing GFP or GFP-TRPV2. WM266.4 (middle) and 451Lu (right) metastatic cells transduced with either control (Ctrl) or TRPV2-targeting sequences V2-1 and V2-2 shRNAs. Data represents means ± SEM of the number of cells indicated on the figures and are representative of at least 3 independent experiments.

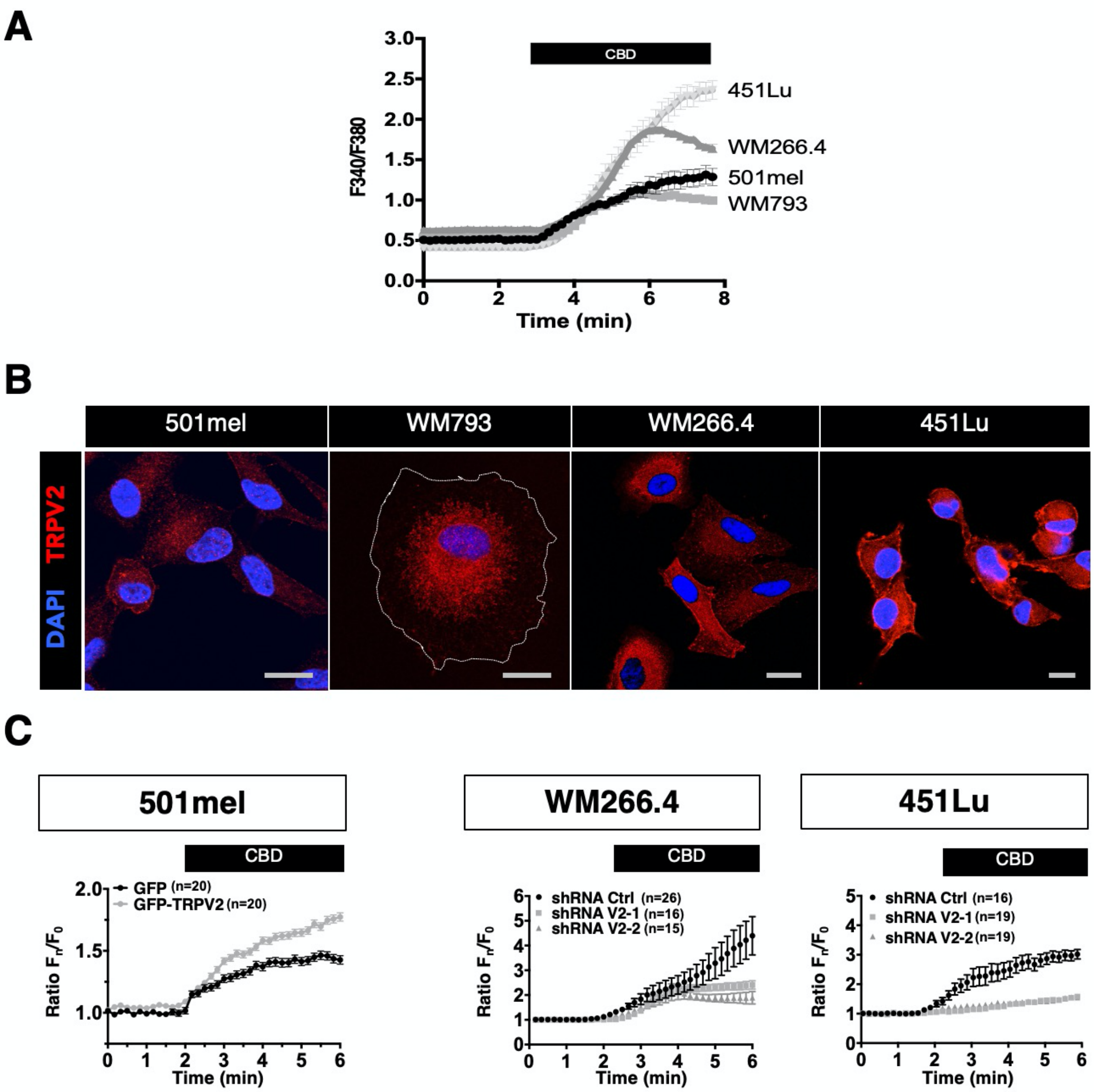

Appendix Figure S3 (related to Figure 3). TRPV2 Ca^{2+}-conducting activity is essential for melanoma tumor cell migration

(A) WM266.4 metastatic melanoma cells migration behavior upon TRPV2 pharmacological inhibition with Tranilast (1mM) assessed in scratch wound healing assays. Left panel shows representative images. Right panel shows gap closure quantification at 24h presented as mean ± SEM (n=5 biological replicates; ****P <0.0001, Unpaired t-test).

(B) Schematic representation of 2D cell migration assays using WM266.4 cells transduced with control (red) or TRPV2 (green) shRNAs, and placed in a 0-5% FCS gradient using an Ibidi migration chamber. Representative fluorescent image showing a typical field of seeded cells being evaluated for 12h, under temperature and CO_2 controlled conditions. (Scale bar = 30μm).

(C) Invasion kinetics of collagen-embedded spheroids based on Figure 3 D-E experiments. Data are presented as mean ± SEM of n>4 independent spheroids for each condition.

A

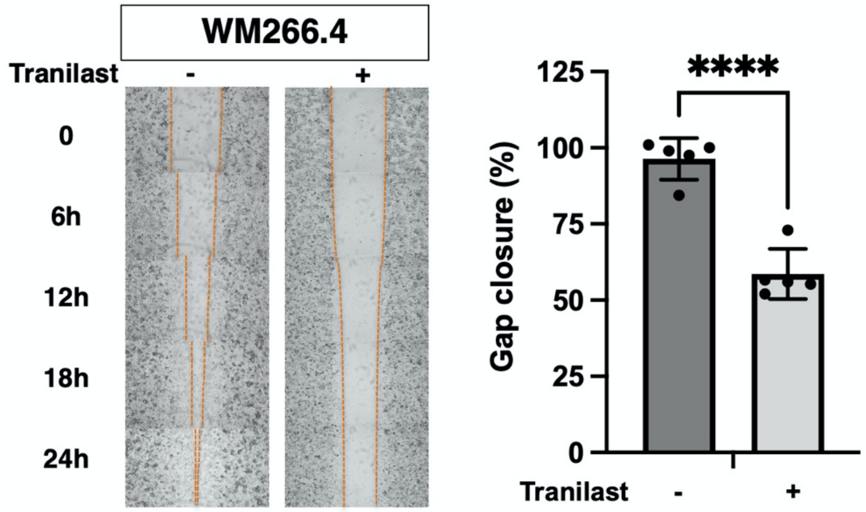

B

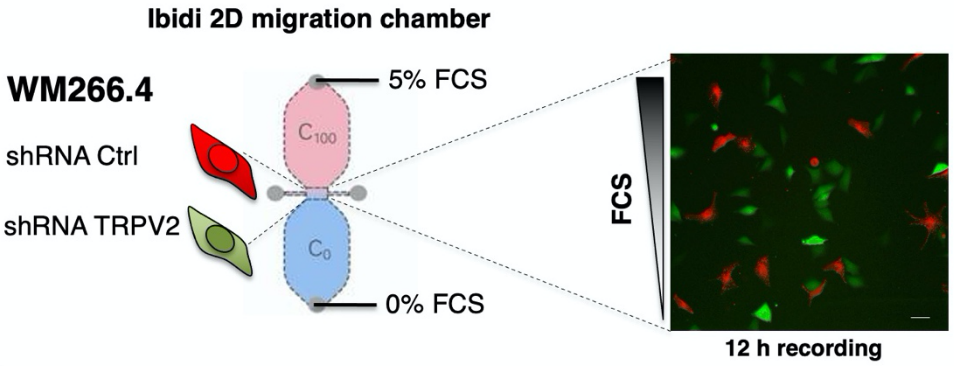

C

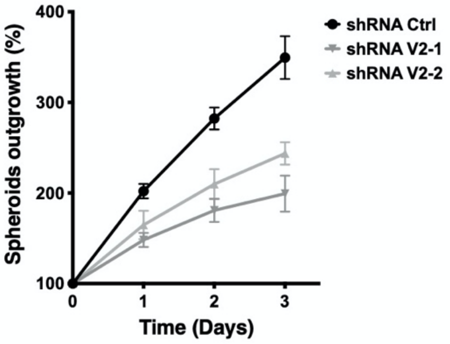

Appendix Figure S4. Modulating TRPV2 expression does not affect pseudo-EMT markers

Representative immunoblots analysis of the indicated “epithelial” and “mesenchymal” markers in control (GFP) or GFP-TRPV2 overexpressing 501mel cells, and in WM266.4 and 451Lu cells expressing either control (ctrl) or TRPV2-targeting (V2-1 or V2-2) shRNAs. HSP60 was used as a loading control. MITF= Microphthalmia-associated transcription factor; E-CAD= E-Cadherin; FN1= Fibronectin; N-CAD= N-Cadherin; SPARC= secreted protein acidic and rich in cysteine; a-SMA= alpha-smooth muscle actin; VIM= Vimentin.

The star indicates the detection of non-phospho, active β-catenin (β-CAT) (non-phosphorylated on Ser33, Ser37 and Thr41 residues, D13A1 Rabbit mAb, Cell Signalling). Note that 501mel cells display high levels of active β-catenin, acting as a suppressor of cell invasion in melanoma (Arozarena *et al*, 2011). WM266.4 cells strongly express the mesenchymal markers tested, suggesting that these cells have completed a mesenchymal transition, while 451lu cells present an intermediate phenotype.

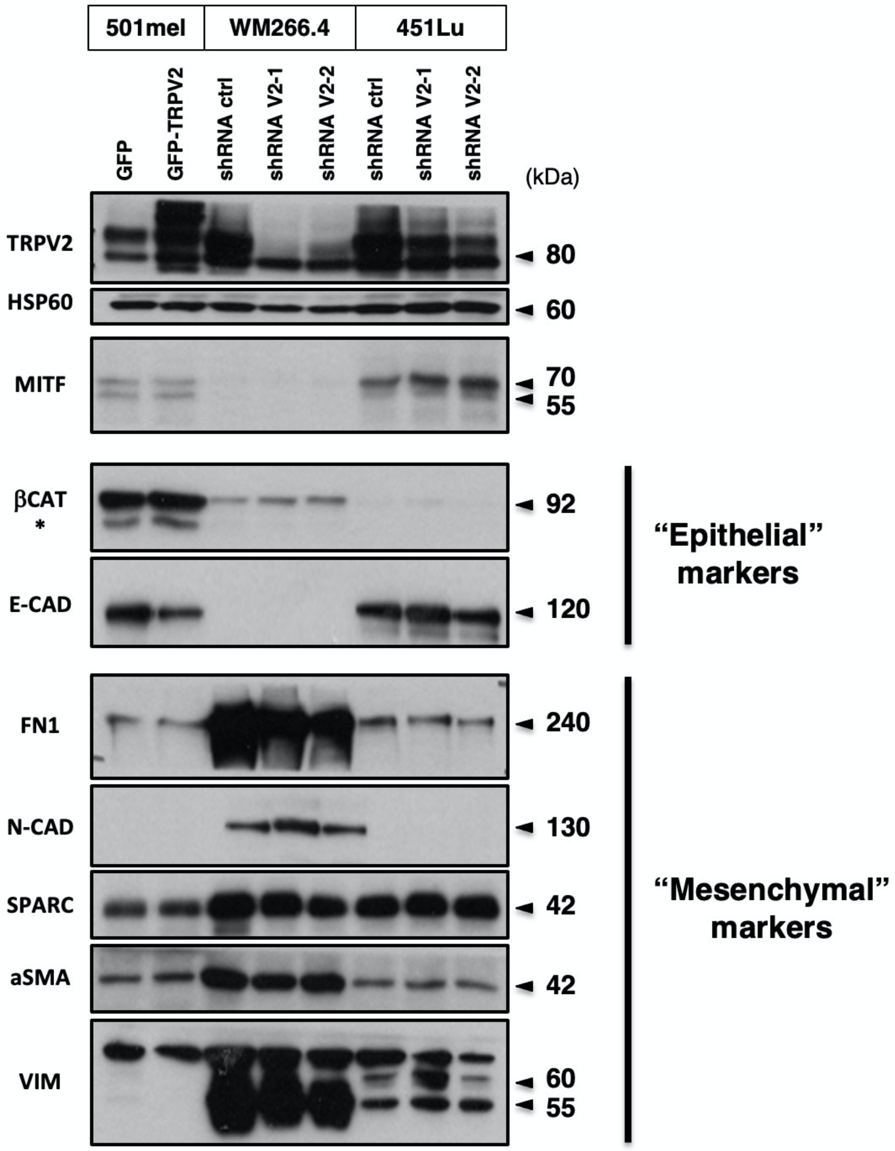

Appendix Figure S5 (related to Figure 4). TRPV2 associates with Vimentin in metastatic melanoma cells

(A) Representative confocal images of low confluence WM266.4 and 451Lu melanoma cells seeded on fibronectin-coated coverslips. Cell nuclei are depicted in blue (DAPI), TRPV2 in red and vimentin (VIM) in green. Bottom panels show magnification of the indicated area and arrows indicate sites of co-localization. (Scale bar = 20μm for top panels and 3μm for bottom panels).

(B) Representative immunoblotting (IB) of reciprocal co-immunoprecipitation (IP) of TRPV2 with vimentin in WM266.4 and 451Lu cells. Experiments were performed three times.

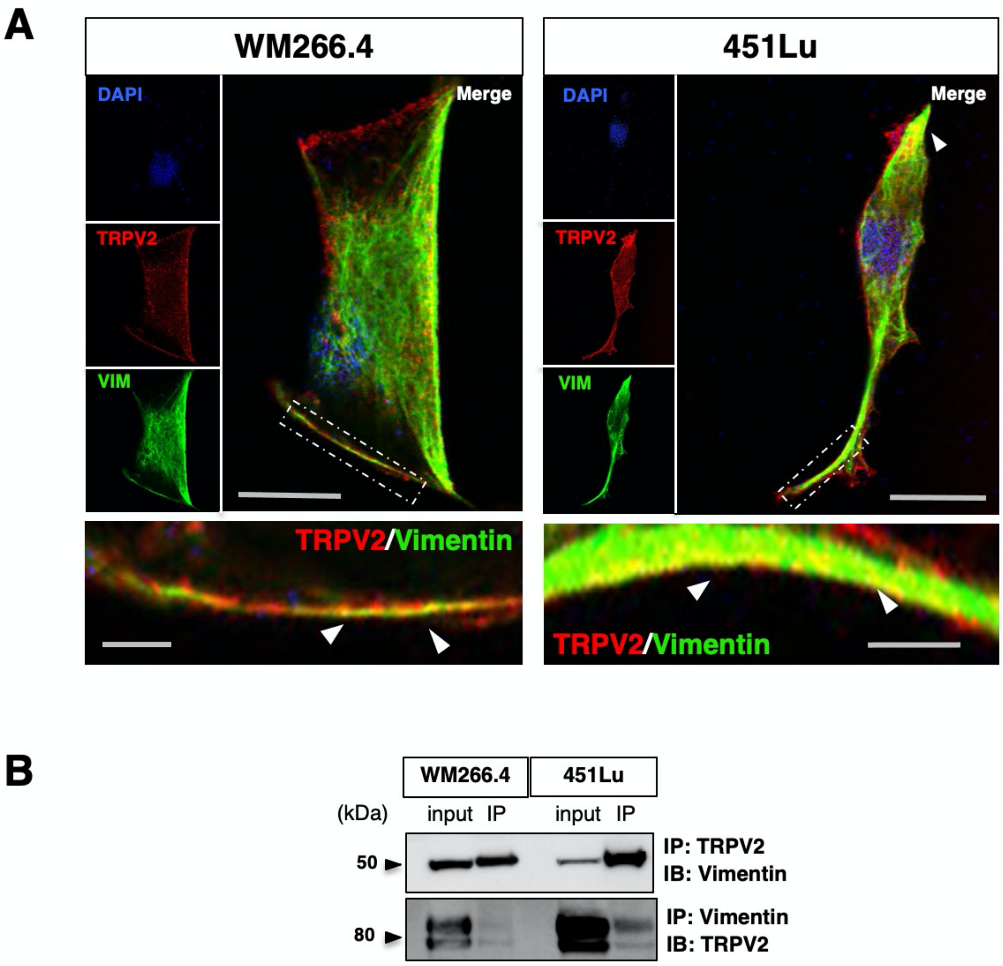

Appendix Figure S6 (related to Figure 5). TRPV2 controls adhesions maturation and turnover through the Ca^{2+}-dependent activation of calpain

(A) Representative vinculin (red) and nuclei (DAPI, blue) staining, used to quantify mature FAs per cell, in cell lines where TRPV2 expression was modulated. (Scale bar = 10µm).

(B) Immunoblotting (IB) assessing the total amount of vinculin following TRPV2 genetic manipulation. β-actin was used as a loading control.

(C & D) IB assessment of the Ca^{2+}-dependency of talin cleavage, an early adhesion protein proteolyzed by calpain. Transfected 501mel cells (C), or wild-type WM266.4 and 451Lu cells (D) were bathed in nominally Ca^{2+}-free (0) or 2 mM Ca^{2+} (2) HBSS external solution. Five minutes prior lysis, cells were treated with either 5 µM Ionomycin, a selective potent Ca^{2+} ionophore allowing normalization of the intracellular [Ca^{2+}] with the extracellular [Ca^{2+}], or with cannabidiol (CBD, 40 µM for 10 min) eliciting a TRPV2-mediated Ca^{2+} influx. Note that following either of these treatments (both resulting in an increased cytosolic [Ca^{2+}]), talin was majoritarly present under its full-length form (Black Arrows) in 0 mM Ca^{2+} cultured cells, whereas a strong occurrence of the 190 kDa calpain-cleaved isoform (Grey Arrows) was observed in the 2 mM Ca^{2+} external solution bathed cells. In the context of TRPV2-dependent Ca^{2+} influx mediated by CBD stimulation, 501mel cells overexpressing GFP-TRPV2 were used instead of GFP cells lacking TRPV2. In all TRPV2-expressing melanoma cells, CBD-induced Ca^{2+} influx leads to talin proteolysis reflecting calpain activation.

(E) Representative immunoblots showing the effect of calpain pharmacological inhibition on the relative levels of full-length (230 kDa; Black Arrow) and the calpain-mediated degradation product (190 kDa, Grey Arrow) of talin in control or TRPV2 overexpressing 501mel cells. Tubulin was used as loading control.

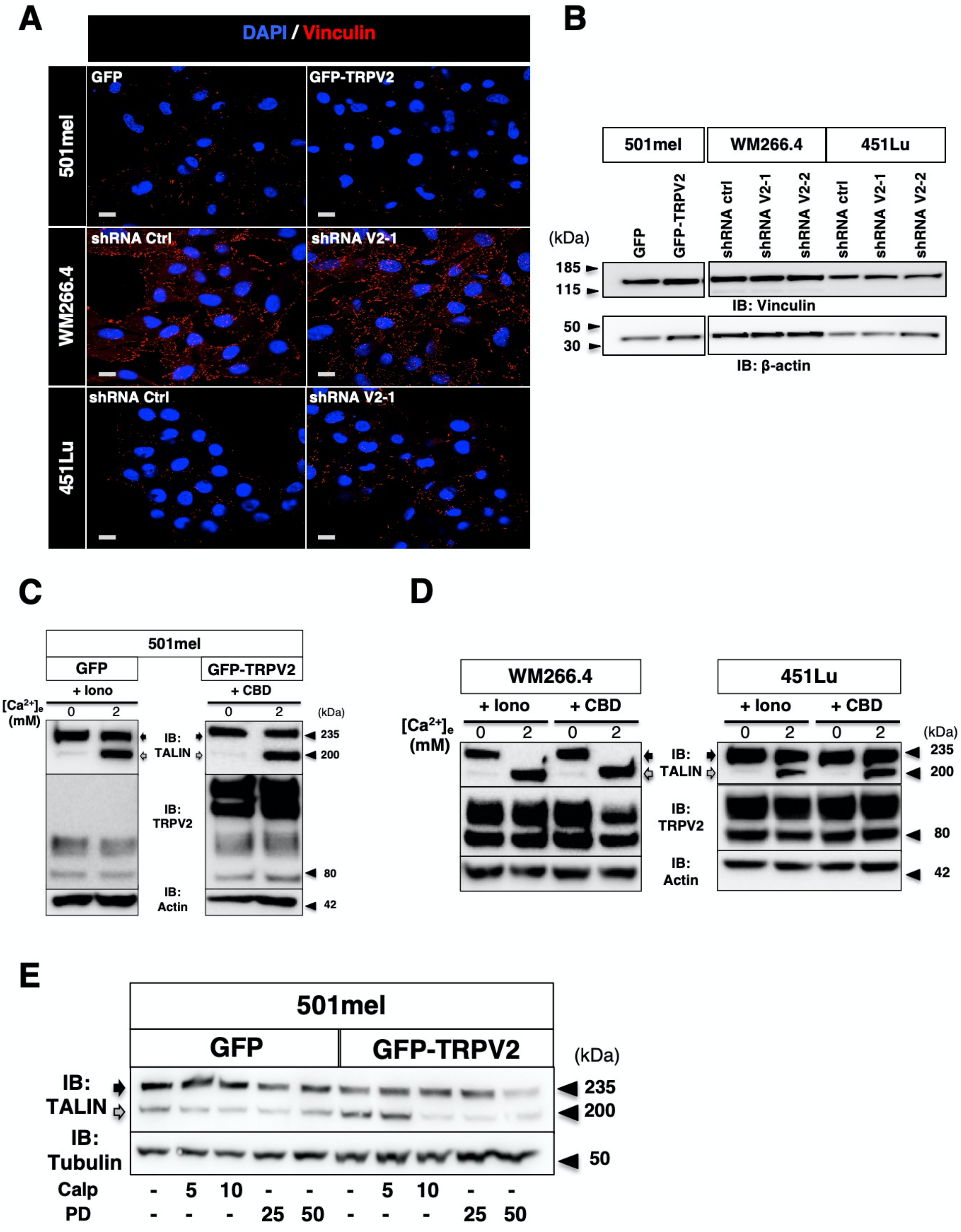
A
DAPI / Vinculin
501mel
GFP
GFP-TRPV2
WM266.4
shRNA Ctrl
shRNA V2-1
451Lu
shRNA Ctrl
shRNA V2-1
B
501mel
WM266.4
451Lu
(kDa)
GFP
GFP-TRPV2
shRNA ctrl
shRNA V2-1
shRNA V2-2
shRNA ctrl
shRNA V2-1
shRNA V2-2
185
115
IB: Vinculin
50
30
IB: β-actin
C
501mel
GFP
GFP-TRPV2
+ Iono
+ CBD
[Ca2+]e (mM)
0
2
0
2
(kDa)
IB: TALIN
235
200
IB: TRPV2
80
IB: Actin
42
D
WM266.4
451Lu
+ Iono
+ CBD
+ Iono
+ CBD
[Ca2+]e (mM)
0
2
0
2
0
2
0
2
(kDa)
IB: TALIN
235
200
IB: TRPV2
80
IB: Actin
42
E
501mel
GFP
GFP-TRPV2
(kDa)
IB: TALIN
235
200
IB: Tubulin
50
Calp - 5 10 - - - 5 10 - -
PD - - - 25 50 - - - 25 50

Appendix Figure S7 (related to Figure 6). TRPV2 knockdown impairs the lung extravasation of melanoma tumor cells.

Lung extravasation/colonization analysis 24 h after inoculation of 451Lu-LUC cells expressing either ctrl shRNA or TRPV2 targeting shRNA. Scatter plots (Box and whiskers) of normalized BLI photon flux in lungs (Boxes extend from the 25th to 75th percentiles, whiskers from min to max, the horizontal line in each box is plotted at the median and each dot correspond to a single mouse (n=7); $^{**}P$ = 0.0082, unpaired *t*-test)

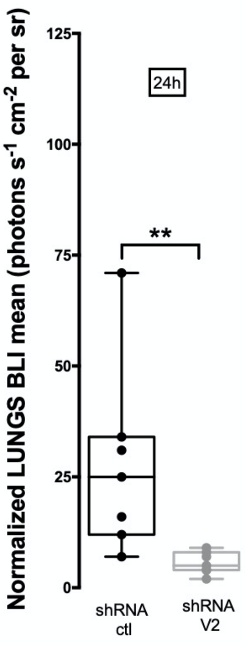

Appendix Figure S8 (related to Figure 6). *Ex vivo* characterization of lung metastasis colonized by human melanoma cells

(A & B) Immunofluorescence analyses of lung sections from mice injected with 451Lu-LUC cells expressing either control or TRPV2 targeting shRNAs. Representative microscopy images of lung sections labeled with DAPI (blue), antibodies against the mouse endothelial cells marker CD31/PECAM-1 (red) and the human melanoma marker MEL (A) or TRPV2 (B) (yellow). (Scale bar = 5 mm).

(C) High magnification pictures of lung metastatic foci showing that human melanoma cells (a, b insets) have colonized the lung parenchyma, as well as the strong expression and plasma membrane localization of endogenous TRPV2 (c, d insets) in these tumor xenografts.

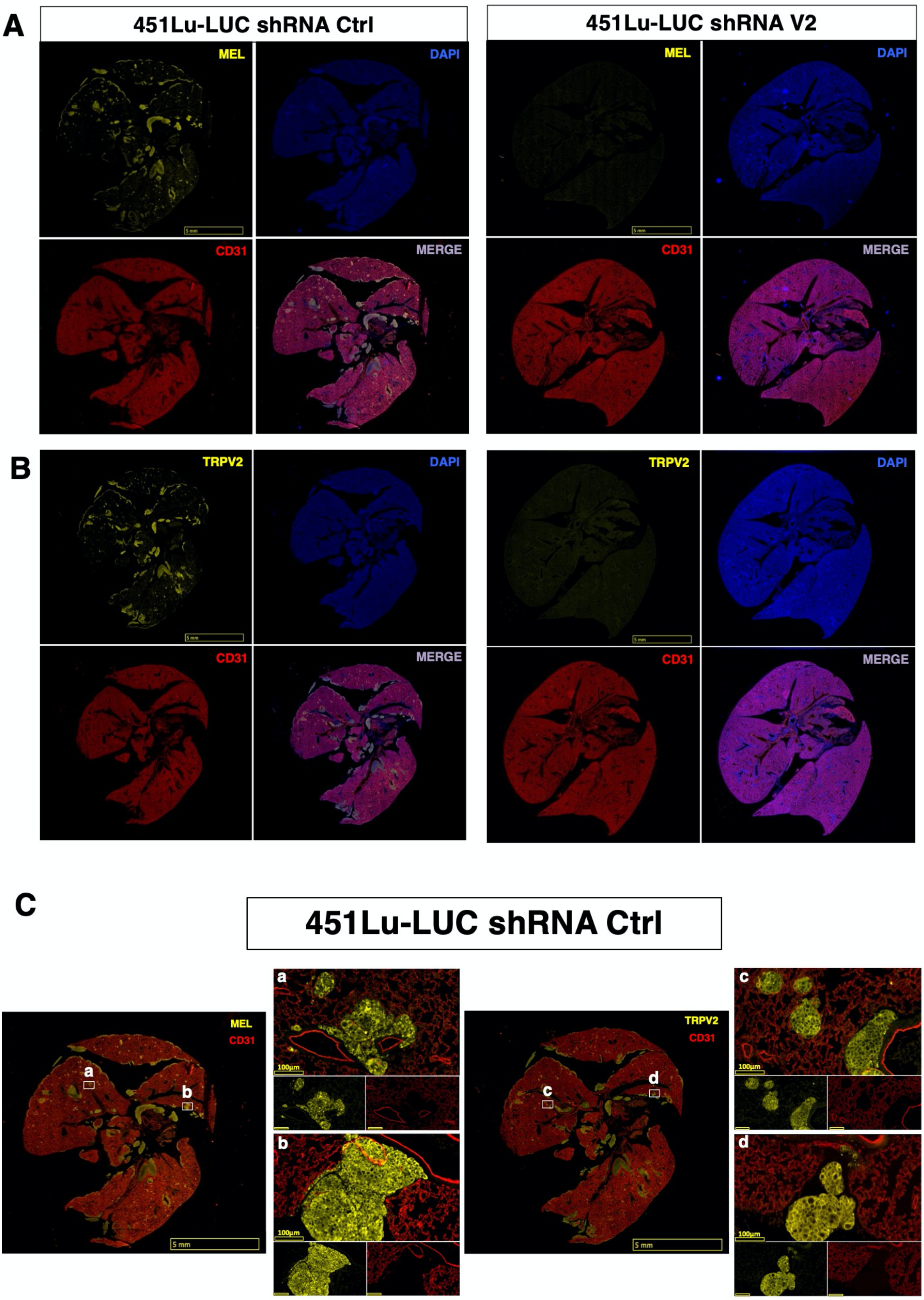
A
451Lu-LUC shRNA Ctrl
451Lu-LUC shRNA V2
MEL
DAPI
CD31
MERGE
B
TRPV2
DAPI
CD31
MERGE
C
451Lu-LUC shRNA Ctrl
MEL
CD31
TRPV2
a
b
c
d
5 mm
100µm

Appendix Figure S9 (related to Figure 6). Direct comparison of the *in vivo* metastatic potential of TRPV2-modulated melanoma cells in xenografted zebrafish

(A) Schematic representation of the experimental strategy used to evaluate the impact of TRPV2 repression in human melanoma cells on micrometastasis formation in xenografted zebrafish. As control- and TRPV2-shRNA expressing WM266.4 cells were both GFP-labeled, control cells were double-stained with the CM-Dil red fluorescent dye, and equal amounts of both populations were co-injected in the duct of Cuvier of 2 days-old zebrafish embryos. 36h post-transplantation, selected xenografted zebrafish were imaged.

(B) Representative fluorescent microscopy images of zebrafish larvae co-injected with shRNA control (orange) and shRNA TRPV2 (green) melanoma cells. Quantification of tumor burden per embryo shows a significant reduction of the metastatic potential of TRPV2-silenced cells compared to control cells. Eyes autofluorescence was not considered in the quantification. Comparison was done in 9 individual zebrafish larvae. Scatter plots show mean ± SD. ****P* <0.001, Unpaired *t*-test.

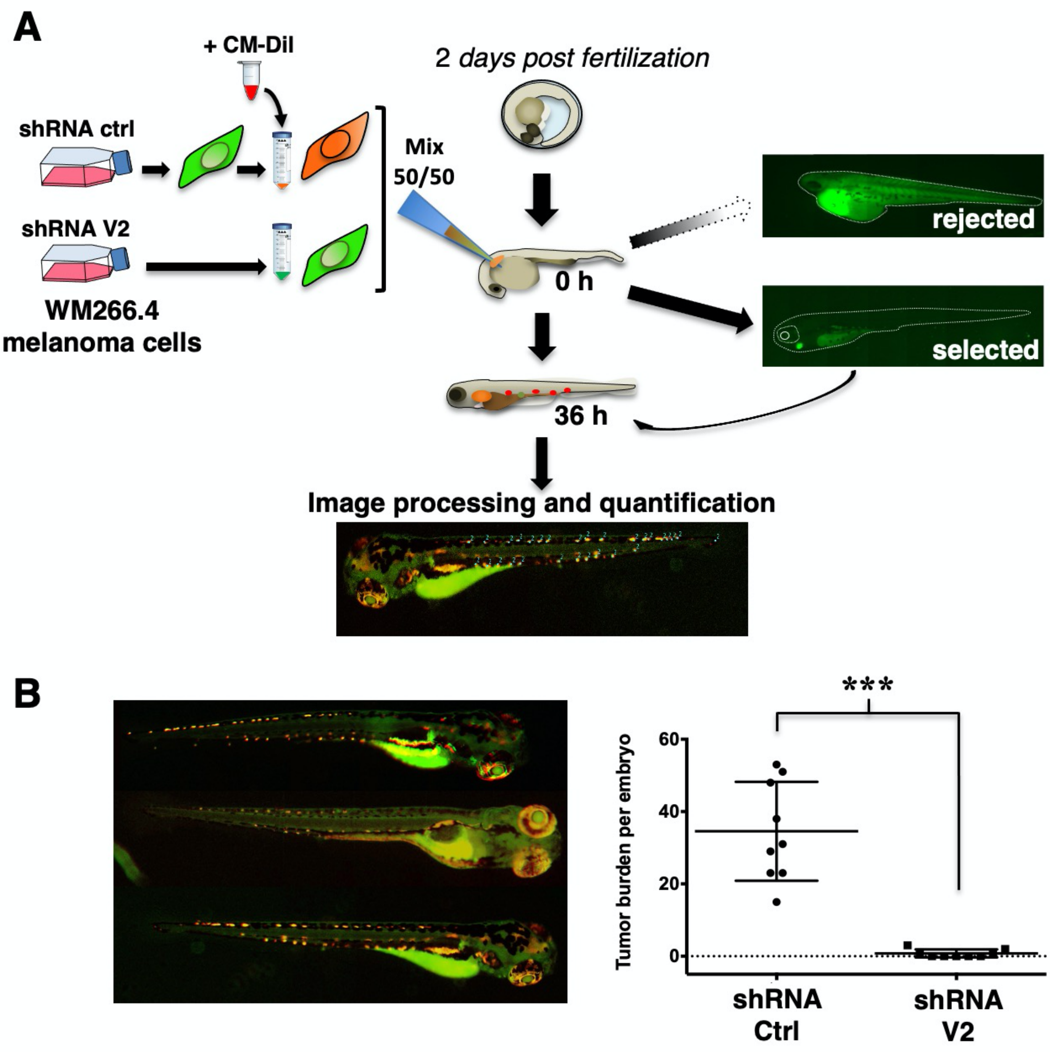

Appendix Figure S10 (related to Figure 7). Antibody validation and quantification method of TRPV2 expression in melanoma patient samples

(A & B) Specificity of the HPA044993 anti-TRPV2 polyclonal antibody (*Sigma*). Non-transfected HEK cells were used as negative control and human TRPV2 transfected HEK cells as positive control. TRPV2 antibody was tested (A) by immunoblotting on WM266.4 and 451Lu melanoma cells either endogenously expressing high levels of TRPV2, or specifically repressed for TRPV2 expression by shRNAs; and (B) by immunohistochemistry on a stage IV melanoma tumor by comparison with its isotype control. (Scale bar = 200µm for HEK and 1mm for Stage IV melanoma).

(C) Global picture of the ME1004c melanoma tissue microarray used to analyze TRPV2 expression.

(D) Schematic representation of the image analysis workflow used for TRPV2 staining intensity quantification.

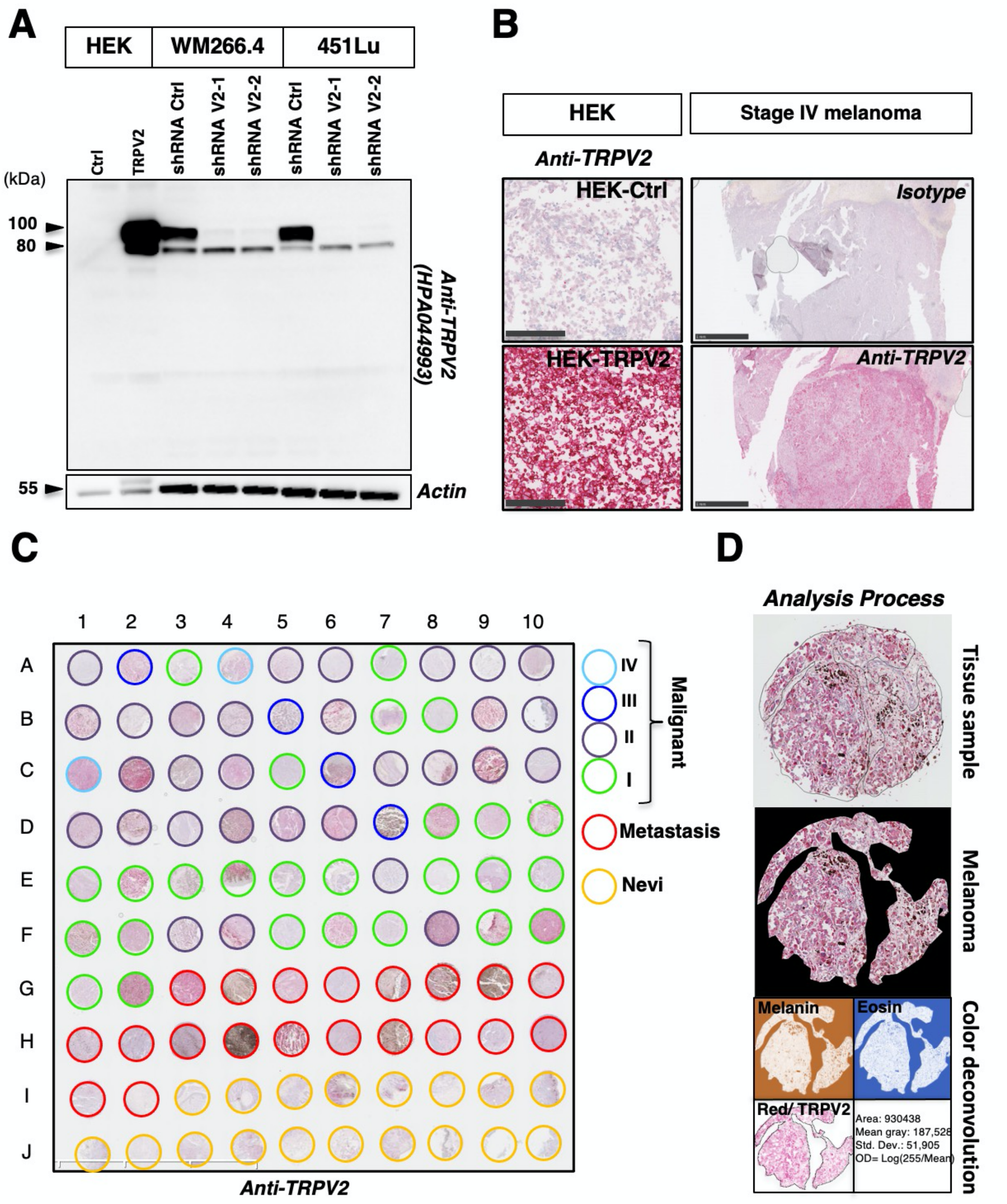
A
HEK
WM266.4
451Lu
Ctrl
TRPV2
shRNA Ctrl
shRNA V2-1
shRNA V2-2
shRNA Ctrl
shRNA V2-1
shRNA V2-2
(kDa)
100
80
55
Anti-TRPV2 (HPA044993)
Actin
B
HEK
Stage IV melanoma
Anti-TRPV2
HEK-Ctrl
Isotype
HEK-TRPV2
Anti-TRPV2
C
1 2 3 4 5 6 7 8 9 10
A B C D E F G H I J
IV
III
II
I
Malignant
Metastasis
Nevi
Anti-TRPV2
D
Analysis Process
Tissue sample
Melanoma
Color deconvolution
Melanin
Eosin
Red/ TRPV2
Area: 930438
Mean gray: 187,528
Std. Dev.: 51,905
OD= Log(255/Mean)

Appendix Figure S11 (related to Figure 7). TRPV2 expression analysis in melanoma patient samples

(A) RNAseq data-based GEPIA generated dot plot profiling TRPV2 differential expression in the TCGA SKCM tumors cohort (n= 461) *versus* [TCGA normal + GTEx normal] datasets (n= 558). Each dot represents a distinct tumor (T) or normal sample (N). Box plot represents the 25th percentile interval and whiskers represent the 95th percentile interval of the distribution. A horizontal line indicates the median value of the expressions. The method for differential analysis is one-way ANOVA, using disease state (Tumor or Normal) as variable for calculating differential expression: The expression data were first log_2(TPM+1) transformed for differential analysis and the log_2FC was defined as median(Tumor) - median(Normal); **P* < 0.01.

(B) Whisker-box plot representing TRPV2 protein expression level according to melanoma stages (TNM grading) in the TMA. (Boxes extend from the 25th to 75th percentiles with Tukey whiskers with outliers plotted as individual points, the horizontal line in each box is plotted at the median).

(C) Immunohistochemistry staining of TRPV2 on grade I (Clark level II Superficial Spreading Melanoma with a Breslow index of 0.4mm) and grade IV (Clark level IV Nodular melanoma with a Breslow index of 6mm) melanoma samples issued from the Rennes University Hospital (CHU) tumor biobank (Centre de ressources biologiques humaines - CRB Santé). (Scale bar = 100µm)

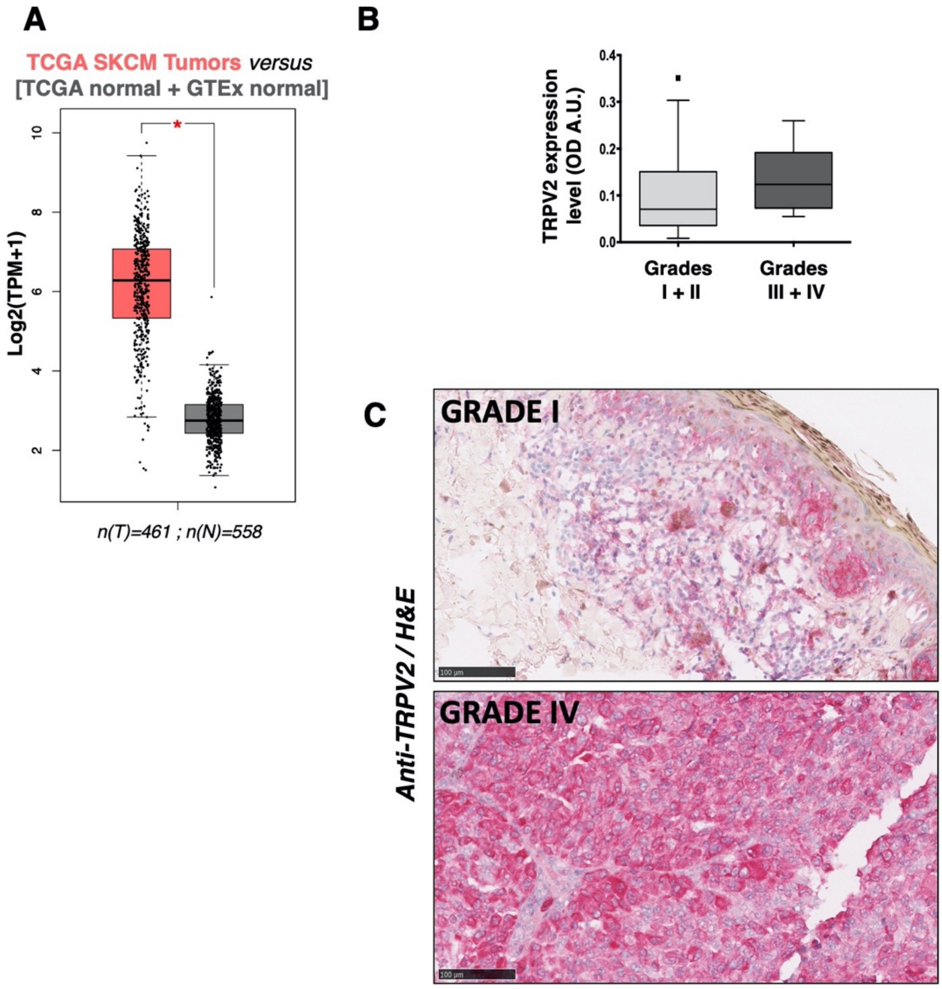

Appendix Table S1. List of PCR primers used in this study.

Gene	Amplicon size	Forward primer sequence (5'-3')	Reverse primer sequence (5'-3')
TBP	161	CGGAGAGTTCTGGGATTGT	GGTTCGTGGCTCTCTTATC
TRPV1	120	GACCTGTGCCGTTTCA	CCTGTGCGACGTGGACTCA
TRPV2	199	GGAATACACAGAGGGCTCCA	CCTCTTCTCAATGGCGATGT
TRPV3	226	ACGAGGCAACAACATCCTTC	CCGCTTCTCCTTGATCTCAC
TRPV4	190/370	CCCGTGAGAACACCAAGTTT	AGTTCATTGATGGGCTCCAC
TRPV6	208	GGATCTGCGGACGGGAGTA	CGAGACACTGAGGGCATAGGA
TRPC1	201	TGGGATGATTTGGTCAGACA	TCTGCCACCAGTGTAGGATG
TRPC6	121	CCTTGCTGTTGCCATTGGAC	GAAGGAGGCTGCGTGTGCTA
TRPM2	303	GGCAGTGGAAGCCTTCAGAT	GATAAAGCGGCTGCGTGAAG
TRPM7	226	AATAATCGGAGGTCTGGCCG	AGCGCTTGGTTTCTGGATCA
TRPA1	140	TGCATGTTGCATTCCACAGAAG	TTGAGGGCTGTAAGCGGTTCATA
STIM1	109	TGTGGAGCTGCCTCAGTATG	CTTCAGCACAGTCCCTGTCA
STIM2	114	GTCTCCATTCCACCCTATCC	GGCTAATGATCCAGGAGGTT
ORAI1	161	ATGAGCCTCAACGAGCACT	GTGGGTAGTCGTGGTCAG
SK3	174	TGGACACTCAGCTCACCAAG	GTTCCATCTTGACGCTCCTC
BRN2	98	GTAACTGTCAAATGCGCGGC	GAGGTGAGCAGGCTGTAGTG

Each primer pair was designed and validated *in silico* using primer3plus and primer-blast software (http://www.bioinformatics.nl/cgi-bin/primer3plus/primer3plus.cgi, http://www.ncbi.nlm.nih.gov./tools/primer-blast/). Primers were designed to localize at intron/exon junctions of sequences commune to all variants of each gene.

Note that the TRPV4 specific primer pair use for RT-PCR amplify TRPV4 variants 1, 4 and 5 with an amplicon size of 370 bp and TRPV4 variants 2 and 3 with an amplicon size of 190 bp (Arniges *et al*, 2006). For qPCR experiments, an alternative primer pair was used amplifying all variants with the same amplicon size of 76 bp (Fwd 5'-CTACGCTTCAGCCCTGGTCTC-3'; Rev 5'-GCAGTTGGTCTGGTCCTCATTG-3' (Spinsanti *et al*, 2008)).

Appendix Table S2. Summary of the exact P value obtained for the multiple comparisons performed on the data presented in Fig 3A and 5B,D.

Fig 3 & 5	WM266.4		451Lu	
	shRNA ctrl vs shRNA V2-1	shRNA ctrl vs shRNA V2-2	shRNA ctrl vs shRNA V2-1	shRNA ctrl vs shRNA V2-2
3A Migration	*** P=0,009*	*** P=0,0034*	**** P=0,0003*	***** P=<0,0001*
3A Invasion	*** P=0,0013*	*** P=0,0017*	***** P=<0,0001*	***** P=<0,0001*
5B (calpain activity)	***P=0,0068*	***P=0,0037*	***P=0,0033*	***P=0,001*
5D (Talin cleavage)	**** P=0,0007*	**** P=0,0005*	**P=0,0388*	**P=0,0265*

Appendix Table S3. Summary of the exact P value obtained for the multiple comparisons performed on the data presented in EV Fig 2B.

EV Fig 2B	Significant?	Summary	Adjusted P Value
GFP vs. GFP + 5µM Calp	*No*	*ns*	*>0,9999*
GFP vs. GFP + 10µM Calp	*No*	*ns*	*0,0712*
GFP vs. GFP + 25µM PD	*No*	*ns*	*>0,9999*
GFP vs. GFP + 50µM PD	*No*	*ns*	*0,3324*
GFP vs. GFP-TRPV2	*Yes*	**	*0,0025*
GFP-TRPV2 vs. GFP-TRPV2 + 5µM Calp	*No*	*ns*	*>0,9999*
GFP-TRPV2 vs. GFP-TRPV2+ 10µM Calp	*Yes*	***	*0,0002*
GFP-TRPV2 vs. GFP-TRPV2 + 25µM PD	*Yes*	*	*0,0121*
GFP-TRPV2 vs. GFP-TRPV2 + 50µM PD	*Yes*	***	*0,0001*

Appendix References

Arniges M, Fernandez-Fernandez JM, Albrecht N, Schaefer M, Valverde MA (2006) Human TRPV4 channel splice variants revealed a key role of ankyrin domains in multimerization and trafficking. *J Biol Chem* 281: 1580-1586

Arozarena I, Bischof H, Gilby D, Belloni B, Dummer R, Wellbrock C (2011) In melanoma, beta-catenin is a suppressor of invasion. *Oncogene* 30: 4531-4543

Spinsanti G, Zannolli R, Panti C, Ceccarelli I, Marsili L, Bachiocco V, Frati F, Aloisi AM (2008) Quantitative Real-Time PCR detection of TRPV1-4 gene expression in human leukocytes from healthy and hyposensitive subjects. *Mol Pain* 4: 51
