# Supplementary material for: The mechanosensitive TRPV2 calcium channel promotes human melanoma invasiveness and metastatic potential
Source: EMBO Rep. 2023 Feb 6;24(4):e55069. doi: 10.15252/embr.202255069 (PMC10074106; doi:10.15252/embr.202255069)
Supplement: Supplementary file 12 [file EMBR-24-e55069-s003.pdf]

EMBO reports

The mechanosensitive TRPV2 calcium channel promotes melanoma invasiveness and metastatic potential.

Kenji Shoji, Elsa Bayet, Sabrina Leverrier-Penna, Dahiana Le Devedec, Aude Mallavialle, Severine Marionneau-Lambot, Florian Rambow, Raul Perret, Aurelie Joussaume, Roselyne Viel, Alain Fautrel, Amir Khammari, Bruno Constantin, Sophie TARTARE-DECKERT, and Aubin PENNA
DOI: 10.15252/embr.20 2255069

Corresponding author(s): Aubin PENNA (aubin.penna@univ-poitiers.fr)

Review Timeline:

Transfer from Review Commons:	17th Mar 22
Editorial Decision:	1st Apr 22
Revision Received:	12th Oct 22
Editorial Decision:	2nd Dec 22
Revision Received:	21st Dec 22
Accepted:	10th Jan 23

Editor: Martina Rembold

Transaction Report: This manuscript was transferred to EMBO Reports following peer review at Review Commons.

(Note: With the exception of the correction of typographical or spelling errors that could be a source of ambiguity, letters and reports are not edited. Depending on transfer agreements, referee reports obtained elsewhere may or may not be included in this compilation. Referee reports are anonymous unless the Referee chooses to sign their reports.)

Revision 0

Review #1

1. Evidence, reproducibility and clarity:

Evidence, reproducibility and clarity (Required)

From the cancer database analysis (for Ca2+ permeable channels that associated with cancer cell mobility behavior), the authors found that TRPV2 transcript expression is exceeding the other Ca2+ permeable channel levels, and further RT-PCR confirmed that the highest expression of TRPV2 transcript is in melanoma and correlated with the more aggressive metastatic phenotypes of melanoma cells. By overexpression of TRPV2 in less metastatic melanoma cells (501mel), or knock-down of TRPV2 in more metastatic melanoma cells (WM266.4 and 451Lu), they showed that TRPV2 levels is functionally correlated with melanoma cells metastasis capability (assayed by both transwell invasive and migration analyses). In addition, they provide some in vitro evidence to support the notion that activation of TRPV2, localized in highly dynamic nascent adhesion clusters, directly regulates calpain-mediated cleavage of the adhesive protein talin together with F-actin network, which is likely the mechanism of TRPV2 activation mediated increased melanoma cell mobility. Overall, the experiments are well designed and of reasonable rational. It's of great interest and have clinical implications for showing that TRPV2 act as a cancer biomarker and potential therapeutic target for metastatic melanoma. I just have several concerns here, 1) how specific the calcium influx via TRPV2 contributes to the downstream signaling, such as calpain activation (as we know many routes of calcium influx can activate calpain); 2) except knock-down of TRPV2, is there any pharmacological inhibitors or pore-region mutation of V2 that can block the calcium influx via TRPV2? If such inhibitor or mutant are able to display the similar effects as knock-down of TRPV2 in cell migration assay, that would definitely add more definitive evidence for the role of TRPV2 in melanoma metastases.

Minor issue:

For Fig. 6 B/D, is there any significant difference between ctrl and shTRPV2 group? It seems the error bar is huge, I doubt this will give you a solid answer for the claim "TRPV2 level determines the in vivo metastases potentials of melanoma cells";

It seems piezo1 levels are equivalent high to TRPV2 from the data screening (Fig. 1A), at least the author should discuss about the possible link between these two channels, as they both mechanical sensitive and calcium permeable.

2. Significance:

Significance (Required)

It's of great interest and have clinical implications for showing that TRPV2 act as a metastatic melanoma biomarker and potential therapeutic target for metastatic melanoma.

3. How much time do you estimate the authors will need to complete the suggested revisions:

Estimated time to Complete Revisions (Required)

(Decision Recommendation)

Between 1 and 3 months

Review #2

1. Evidence, reproducibility and clarity:

Evidence, reproducibility and clarity (Required)

In their manuscript, Shoji et al uncovered a role for the calcium channel TRPV2 in melanoma cell migration and invasion. They identified TRPV2 as one of the most highly expressed Ca2+ channels in human melanoma and provided evidence suggesting that TRPV2 activity at the plasma membrane correlates with melanoma aggressiveness. Strikingly, genetic modulation of TRPV2 affected melanoma cell migration and invasion capacities of 3 human melanoma cell lines in vitro and in xenograft models. TRPV2 colocalized with paxillin at the leading edge, activated calpain and elicited the cleavage of talin, pointing to nascent adhesion turnover as a mechanism by which TRPV2 promotes melanoma cell motility. The manuscript is well organized and the data supporting TRPV2 function in melanoma cell migration is compelling and clearly presented. To strengthen their conclusions, the authors should consider the following points:

Major points:

1)The gene expression data and TMA staining suggest that high TRPV2 is associated with advanced melanoma. Is the TRPV2 locus altered at the genomic level (by mutation or copy-number alteration)? Could the authors investigate the mechanism of TRPV2 overexpression in melanoma compared to normal melanocytes or benign nevi? Knowing more about the regulatory elements in the TRPV2 promoter or enhancer regions and/or about the regulators of TRPV2 stability/cell surface localization would add considerable value to this study.

2)To explain the effects of TRPV2 manipulation on melanoma cell migration and invasion, the authors propose a mechanism whereby TRPV2-dependent Ca2+ influx activates calpain, resulting in talin cleavage and dissolution of nascent focal adhesion. However, these changes observed upon TRPV2 overexpression or downregulation are not formally implicated in the regulation of melanoma cell motility. To prove a causal link between these correlative observations, the authors should i) use calpain inhibitors or express calpain-uncleavable talin and measure cell migration in the context of TRPV2 overexpression, and ii) express a constitutively active form of calpain or downregulate talin and measure cell migration in

the context of TRPV2 downregulation. These experiments would provide a more complete demonstration of the proposed mechanism and would improve the manuscript.

Minor points:
The authors should mention the published literature on the role of TRPM1 in melanoma (especially progression to metastatic disease) in their introduction.

Could the authors speculate on the apparent melanoma specificity of TRPV2 expression (figure 1)?

Page 10, how was the invasiveness of melanoma cell lines measured? The authors should refrain from claiming that there is a "positive correlation between invasiveness and TRPV2 expression" (page 10) based on results from just 4 human melanoma cell lines. To support their claim, they would need to expand their panel of cell lines and/or use appropriate statistics (such as Pearson's correlation test).

Could the authors examine the potential correlation between TRPV2 expression and MITF and AXL levels, which are well-accepted markers of the balance between growth and migration in melanoma?

How does TRPV2 overexpression in Figure 2C compare with normal expression levels in invasive cell lines?
Were the FA analyses presented in Figure 5 performed on fibronectin-coated plates? Please consider adding these details in the figure legends.

Could the authors provide more explicit statistical analyses on Figures 6 and 7D?

How do the authors explain that TRPV2 colocalizes with paxillin and not phospho-FAK (in contrast to what is depicted on Figure 7G)? Could the authors also show talin and TRPV2 staining in Figure 4?

The mechanosensitive nature of TRPV2 is not addressed experimentally in this manuscript. Please state published results more explicitly when referring to this aspect of TRPV2 activity regulation (for example on pages 13 and 19).

2. Significance:

Significance (Required)

The manuscript by Shoji and colleagues provides new insights into the role of calcium channels in melanoma progression, a field that remains largely unexplored. The identification of TRPV2 as a new regulator of melanoma cell migration and invasion is all the more significant that TRPV2 expression levels inversely correlate with patient survival. Future studies will establish whether TRPV2 represents a viable drug target in melanoma.

Field of expertise of the reviewer: melanoma, cancer genetics, metastasis

Referees cross-commenting

I think that the points raised by the other 2 reviewers are well taken. My major point 1 could be disregarded if the other reviewers feel that it is beyond the scope of the paper. However, I still feel that the paper could be stronger if the authors more formally proved the proposed mechanism of action of TRPV2 (major point 2).

3. How much time do you estimate the authors will need to complete the suggested revisions:

Estimated time to Complete Revisions (Required)

(Decision Recommendation)

Between 1 and 3 months

Review #3

1. Evidence, reproducibility and clarity:

Evidence, reproducibility and clarity (Required)

In this manuscript, Shoji et al. describe the involment of mechanosensitive TRPV2 calcium channel in melanoma invasiveness and metastatic. This paper is clearly written and provides convicing data. The association of TRPV2 in melanoma metastasis was elucidated clearly in both in vitro and in vivo based models using TRPV2 over-expression and silencing. We have the following minor remarks:

1.A description of the TRP family in the introduction would be helpful: How is the TRPV2 channel different from other family members? Which normal tissues is TRPV2 highly expressed? Most importantly, why did the authors decide to focus on TRPV2 since several other TRP proteins are highly expressed in melanomas including TRPM1 and TRPM7.

2.Figure 2A: It would be better to include a cell surface protein as loading control on this Western blot.

3.Figure 2A: Why is the upper band of different sizes? Has the TRPV2 channel been glycosylated differently in different cell lines? Or do these represent different splice forms?

4.Statistical significance is not shown changes in figure S3. Thus, the claim on page 11 "Although, TRPV2 overexpression increased the growth rate of the non-invasive 501mel cells, TRPV2 silencing had no impact on the viability nor on ERK phosphorylation in either metastatic cell lines, suggesting that TRPV2 is dispensable for malignant melanoma proliferative/survival (Figures S3A-B)" is not validated.

5.Did OE and KD TRPV2 show changes in cell morphology?

6.The authors should disscuss why the mice injected with GFP-TRPV2 overexpressing cells showed numerous metastatic foci in lungs, brain and bones, whereas, "TRPV2 repression prevented the

extravasation of melanoma cells into the lungs" only?

7.Page 15 : "The TCGA skin melanoma RNAseq dataset was first compared to the matched TCGA and GTEx normal datasets (Figure S13A)." The authors should clarify which subset of the GTEx normal dataset was used.

8.Page 25: Figure 1 legends: add T to the at the beginning of the sentence "The Ca2+- permeable channels plotted on the x-axis are grouped by family...."

9.English needs to be improved throughout the manuscript. A few examples are listed below:

a.Abstract: „...is of primary importance (since being) AS IT IS the main cause..."

b.Abstract: „ Here, we (evidenced a) SHOW THE prominent expression...."

c.Introduction: „..The scope of this study was to identify AN ATYPICAL PROFILE among the numerous...."

d.Materials and Methods: Mice model should be Mouse models.

e.Results: „TRPV2 TRANSCRIPTS STOOD OUT AS THE MOST EXPRESSED among (most members of) the major Ca2+ permeable channels...."

f.„Importantly, the (utmost expression of) TRPV2 transcripts (was) WERE (also revealed) SHOWN TO BE HIGHLY EXPRESSED in SKCM tumours AS compared to...."

g.Page 12: The sentence: "In these define proximal clusters..." Needs to be rearranged or clarified.

h.Discussion: „Nevertheless, modulating TRPV2 expression - either way - had no impact on THE EXPRESSION OF EMT markers. „

i.Discussion: „..the highly invasive WM266.4 cells (were) exhibitED antagonistic markers profiles...."

j.Discussion: „These observations were (in adequation) CONSISTENT with .....as well as with (their levels of) THE EXPRESSION OF THE BRN2 invasiveness marker."

k.The sentence „Therefore, globally impacting resting...." lacks an ending.

l.„(By being recruited) RECRUITMENT OF TRPV2 at the PM within paxillin-rich.....structures PLACES THIS mechanosensitive channel at...."

2. Significance:

Significance (Required)

This manuscript is significant and important as it places the mechanosensory TRPV2 channel in a key role in melanoma metastasis. The authors convincingly show the role of this channel in this process.

Referees cross-commenting

I agree with point 2 raised by reviewer 2. It should not be a major burden to perform the suggested experiments (effects of calpain inhibitor or talin knockdown on migration) but will have a major impact on the strength of the paper. The rest of the suggestions made are cosmetic changes and clarifications.

3. How much time do you estimate the authors will need to complete the suggested revisions:

Estimated time to Complete Revisions (Required)

(Decision Recommendation)

Less than 1 month

Revision Plan

Manuscript number: RC-2021-01119
Corresponding author(s): Penna, Aubin

[The "revision plan" should delineate the revisions that authors intend to carry out in response to the points raised by the referees. It also provides the authors with the opportunity to explain their view of the paper and of the referee reports.
The document is important for the editors of affiliate journals when they make a first decision on the transferred manuscript. It will also be useful to readers of the reprint and help them to obtain a balanced view of the paper.

1. General Statements

Insert here any general statements you wish to make about the goal of the study or about the reviews.

We are sincerely grateful for the tremendous impact this constructive review has on our study. Every comment has been carefully taken into account, and will definitely strengthen our conclusions and improve the manuscript. We appreciate the clear interest that all three Reviewers have shown regarding the significance of the study rationale and its clinical implications in the field of metastatic melanoma. We are also thankful for their appreciation of the novelty of the findings, the pertinence of the data, as well as the clarity of the presentation.

As clearly articulated in this report, our main goal was to provide insights into the urgent need for a new biomarker and potential therapeutic target for metastatic melanoma. Our *in vitro* and *in vivo* data identified the mechanosensitive TRPV2 calcium (Ca^{2+}) channel as a new regulator of melanoma cells metastatic potential. Additionally, we could correlate the expression of TRPV2 to the aggressiveness of the disease and patients' survival, opening new perspectives for future treatment. Beside sustained efforts to validate the new role of this Ca^{2+} channel in the metastatic dissemination, we investigated the mechanistic basis enabling such function. By modulating TRPV2 expression, and therefore directly affecting its "constitutive" activity, we found that the activity of the Ca^{2+}-activated calpain, together with the cleavage of its substrate, the adhesive protein Talin, were dependent upon TRPV2. Such new findings, establishing a mechanism connecting a mechanosensitive channel to the intracellular machinery regulating the adhesion/migration processes in advanced melanoma, are highly significant and promising, albeit raising numerous questions. Based on our initial data, we however did not establish a clear causal link between TRPV2-dependent activation of calpain and melanoma cells migration, even if the almost exact correlation in terms of amplitude between TRPV2 expression/activity, calpain activity and the resulting migration behaviors was striking. As suggested, we are now addressing this point by assessing the migration potential of melanoma cells upon calpain activity modulation in the context of TRPV2 expression or repression. Meanwhile, and according to the present review, this manuscript convincingly achieves our initial goal, namely to identify a new biomarker and potential therapeutic target for advanced melanoma, by placing TRPV2 mechanosensitive channel as a key regulator of metastatic melanoma cells migration.

PS: To facilitate the reading, the points raised by the referees are in black italic and our reply is in blue.

2. Description of the planned revisions

Insert here a point-by-point reply that explains what revisions, additional experimentations and analyses are planned to address the points raised by the referees.

Reviewer #2: ***Major points 2.***
To explain the effects of TRPV2 manipulation on melanoma cell migration and invasion, the authors propose a mechanism whereby TRPV2-dependent Ca2+ influx activates calpain, resulting in talin cleavage and dissolution of nascent focal adhesion. However, these changes observed upon TRPV2 overexpression or downregulation are not formally implicated in the regulation of melanoma cell motility. To prove a causal link between these correlative observations, the authors should i) use calpain inhibitors or express calpain-uncleavable talin and measure cell migration in the context of TRPV2 overexpression, and ii) express a constitutively active form of calpain or downregulate talin and measure cell migration in the context of TRPV2 downregulation. These experiments would provide a more complete demonstration of the proposed mechanism and would improve the manuscript.

Reviewer #2: **Referees cross-commenting**
I think that the points raised by the other 2 reviewers are well taken. My major point 1 could be disregarded if the other reviewers feel that it is beyond the scope of the paper. However, I still feel that the paper could be stronger if the authors more formally proved the proposed mechanism of action of TRPV2 (major point 2).

Reviewer #3: **Referees cross-commenting**
I agree with point 2 raised by reviewer 2. It should not be a major burden to perform the suggested experiments (effects of calpain inhibitor or talin knockdown on migration) but will have a major impact on the strength of the paper. The rest of the suggestions made are cosmetic changes and clarifications.

We fully agree with this comment, and firmly believe that proving a causal link between the TRPV2-dependent activation of calpain and melanoma cells migration would strengthen our conclusions. To that end, and as suggested by the Reviewers, we have undertaken the following additional experiments:

1- In the context of TRPV2 overexpression, we will measure 501mel melanoma cells migration potential in presence of a calpain inhibitor.

2- Reciprocally, in the context of TRPV2 repression, we will assess the migration potential of WM266.4 cells upon expression of either a constitutively active form of calpain, or a siTalin.

Here, invalidating Talin would additionally address the hypothesis that Talin is a major substrate of calpain involved in TRPV2-driven metastatic melanoma cells motility. Although in our study Talin proteolysis was initially used as a second-line readout of calpain activity, given its major role in early adhesion turnover, it is definitely a highly propitious target worthy of interest. However, Talin might not be the only substrate of calpain involved in melanoma cells migration, especially given that calpains have a very large panel of substrates involved in the regulation of adhesions dynamics, including other cell motility modulators, such as Paxillin, Vinculin, or Filamin A (Lamsoul *et al*, 2020). Regarding the overexpression of a constitutively active form of calpain, several points have to be taken into account. First, calpains activity is involved in both early and engaged adhesions, at the leading and the rear edges of the cell, which means that boosting calpains activity may fully disrupt the adhesive properties of the cells (and possibly lead to culture issues for instance). Calpains activity has also been involved in regulating proliferation. Finally, uncontrolled activity of calpain can also induce cell death, notably through apoptosis by activating caspases.
Additionally, within the calpain family, composed of 15 members, the ubiquitous calpain-1 and -2 are the most studied members and are both notably involved in cellular motility. Given that talin is

described as a calpain-2 substrate, and that we observed TRPV2-dependent cleavage of talin in metastatic cells, we could presume that TRPV2-mediated Ca^{2+} influx activates at least calpain-2, which could legitimate the expression of constitutively active form of calpain-2.

Experimentally, both options, the expression of either a constitutively active calpain or a siTalin, involved transfection/overexpression. Transient expression may fail in providing a balance between the appropriate level of expression within the cell, and the ratio of expressing *versus* non-expressing cells, resulting in a highly heterogeneous population in which reliable measurement of migration can be complex. Although establishing stable cell lines would be of great interest for future studies, it will require some extra-time.

In any event, we are convinced that these additional experiments will add value to this study and will reinforce the mechanistic model presented in the manuscript. They will also set the basis for future investigations on the subcellular localization of calpains and their substrates in migrating melanoma cells. A very interesting study has indeed shown an asymmetrical distribution of calpain-2 in neutrophils, which is recruited at the leading edge of the cell during early pseudopod formation to promote directionality (Nuzzi *et al*, 2007).

3. Description of the revisions that have already been incorporated in the transferred manuscript

Please insert a point-by-point reply describing the revisions that were already carried out and included in the transferred manuscript.

Reviewer #1:

***1)** how specific the calcium influx via TRPV2 contributes to the downstream signaling, such as calpain activation (as we know many routes of calcium influx can activate calpain);*

Other routes of Ca^{2+} influx, such as Store Operated Calcium Entry (SOCE), TRPM7 or PIEZOs, have indeed been described as potential activators of calpain, regulating cell adhesion (Chen *et al*, 2011; D'Souza *et al*, 2020; Liu *et al*, 2021; Pardo-Pastor *et al*, 2018; Su *et al*, 2006; Yang *et al*, 2009; Yao *et al*, 2020). It is now well-accepted that in the rear of migrating cells, Ca^{2+} activates calpain which catalyzes the cleavage of FA proteins, such as integrins, talin, vinculin and FAK (Glading *et al*, 2002), promoting the disassembly of the mature structures. As the SOCE signal is significantly impeded in invasive (compared to non-invasive) melanoma cells (Hooper *et al*, 2015), and since this Ca^{2+} entry is not defined as mechanosensitive and its local recruitment at adhesion sites is not clear, SOCE does not appear as a prime candidate in regulating nascent adhesion in advanced melanoma cells. Regarding TRPM7, its ion permeability is distinctive and allows the passage of Ca^{2+}, Mg^{2+} and other cations. This chanzyme has been described as a regulator of calpain-2 (m-calpain) activation in vinculin-containing FAs, but evidence suggest that this TRPM7 function is achieved neither by raising the global cytosolic Ca^{2+} concentration, nor through its kinase activity, but rather *via* increasing the intracellular Mg^{2+} concentration (Su *et al.*, 2006; Su *et al*, 2010; Su *et al*, 2011), leaving the regulation of the Ca^{2+} signal controlling calpain activity as an opened question. Finally, concerning PIEZO1, despite a clear role in regulating FA dynamics (assembly of mature adhesions and not disassembly) its precise function in melanoma migration remains undefined (Chen *et al*, 2018), specially since this channel has been reported as stabilizing adhesion in normal but not in transformed cells (Yao *et al.*, 2020). Importantly, the same study showed that PIEZO1 did not localize to paxillin-containing adhesions in A2058 melanoma cells. However, we do not exclude a cooperation between PIEZO1 and TRPV2 in migrating metastatic cells (which is further discussed below in answer to Reviewer#1's minor issue), that might be involved in the regulation of TRPV2 addressing to the plasma membrane. In any event, we can not rule out the participation of other Ca^{2+}-regulating actors into the modulation of calpain activity.

Importantly, to understand the role of each of these channels in cellular migration, it is necessary to consider the cellular model and context in which these studies are conceived. For instance, most of the above reports relate to mature focal adhesions, leaving the mechanistic basis for nascent adhesion dynamics extremely unsettled. In fibroblast, one report evokes the role of Ca^{2+} by showing that adhesion maturation and proper mechanosensing are regulated by the force-induced cleavage of Talin in early adhesions, which depends upon calpain and the TRPM4 channel (Saxena *et al*, 2017). Although TRPM4 is a Ca^{2+}- and voltage-activated channel, it is permeable only to monovalent cations and not to Ca^{2+}, leaving once again the question of the upstream Ca^{2+} signaling regulator unanswered. In parallel, it is noteworthy that numerous reports involving Ca^{2+} channels activity in cellular migration were based on pharmacological modulations, which can be misleading due to nonspecific/off-target effects. In addition, the activation of a Ca^{2+} channel by an agonist often triggers a massive signal, likely different from the fine-tuned constitutive or endogenous activation, potentially leading to artifacts. Hence, we believe that pharmacological studies, although contributing to our understanding, should be balanced by several approaches to enable solid conclusions.

In our study, using TRPV2 gain or loss-of-function strategies, we showed that the modulation of TRPV2 expression and subsequently of its associated constitutive activity (Figures 2C-E and S2C)

were inducing analogous changes in both calpain activity and the cleavage of its known substrate, Talin (Figure 5 B-D). More precisely, the levels of modulations induced on TRPV2 activity, whether it was up- or down-regulated, were strictly comparable and proportional to the rate of alterations observed on calpain activity and Talin proteolysis. As a matter of fact, TRPV2 silencing prevented at least 50% and up to 80% of Talin cleavage in metastatic cells. The pharmacological activation of TRPV2 by its potent agonist (cannabidiol), in presence or absence of Ca^{2+}, further established the Ca^{2+} dependency of calpain-mediated cleavage of Talin (Figure S7C and D). Altogether these data strongly support the conclusion that, at least part of the Ca^{2+}-activated proteolytic activity of calpain depends upon TRPV2 channel-mediated Ca^{2+} signal. As TRPV2 preferentially localized within nascent adhesion structures, we speculate that in advanced melanoma cells, TRPV2 is regulating calpain-mediated cleavage of adhesion proteins in a spatiotemporally-regulated manner. However, we do not exclude the participation of other Ca^{2+} regulators in the cellular adhesion process. This point has been clarified in the discussion section of the manuscript **(page 22)**.

***2)** except knock-down of TRPV2, is there any pharmacological inhibitors or pore-region mutation of V2 that can block the calcium influx via TRPV2? If such inhibitor or mutant are able to display the similar effects as knock-down of TRPV2 in cell migration assay, that would definitely add more definitive evidence for the role of TRPV2 in melanoma metastases.*

As previously discussed in response to the first comment of Reviewer#1, we believe that pharmacological studies strengthen our understanding, but should be balanced with other approaches. Regarding TRPV2, the pharmacological tools available are highly controversial mainly because of specificity issues. Several compounds have been shown to affect TRPV2 function, however most are contentious compounds still under scrutiny (Iwata *et al*, 2020; Vriens *et al*, 2009). Among them, SKF96365 and Ruthenium Red have been identified as non-specific TRPV2 channel blockers. As an inhibitor, Tranilast remains the most used, although it has not been fully validated as a specific blocker of TRPV2. To consolidate the role of TRPV2-mediated Ca^{2+} influx during melanoma metastatic progression, we tested the effect of Tranilast on invasive melanoma cells, and could measure a significant reduction of their migration behavior assessed with wound healing assays. This data has been included in the present manuscript as a supplemental figure **(Fig S4A)**. Meanwhile, the inhibitory mechanism and the specificity of Tranilast towards human TRPV2 still need further clarifications.

Minor issue:

- For Fig. 6 B/D, is there any significant difference between ctrl and shTRPV2 group? It seems the error bar is huge, I doubt this will give you a solid answer for the claim "TRPV2 level determines the in vivo metastases potentials of melanoma cells";

We sincerely apologize for forgetting to include the statistical analysis of the *in vivo* and *ex vivo* data **Figure 6**, error which we have rectified.

When comparing the number of metastatic foci *ex vivo*, unpaired t-tests were applied, and the resulting P values were 0.0397 (*) in the 501mel-GFP *versus* -GFP-TRPV2 (Figure 6B), and 0.0691 in the 451Lu-shCtrl *versus* -shTRPV2 (Figure 6D). Hence, TRPV2 overexpression in the 501mel model showed a significant increase compared to the control in terms of the number of metastasis, while despite showing a clear trend, invalidation of TRPV2 in the 451Lu cells did not achieve statistical significance *ex vivo* (P=0.0691). As for ethical reasons, each group was limited to 6 to 7 mice, adding a couple of animals may have increased the statistical power. Importantly, in the same group of 451-Lu injected mice, *in vivo* imaging displayed a highly significant loss of photon flux (P=0.0064 **) upon TRPV2 silencing (Figure 6C). Additionally, to strengthen our conclusions, we took advantage of the xenografted zebrafish model, allowing a direct comparison of two cell lines with distinct invasive

potentials in the same organism. In this *in vivo* model, the disseminating potential of human melanoma cells was substantially dependent on TRPV2. Hence, based on the results obtained in 3 different models, we concluded that TRPV2 was critical to promote the *in vivo* metastasis potential of human melanoma cells, and have adjusted accordingly the manuscript (**page 17: *TRPV2 expression is critical to the in vivo metastatic potential of melanoma tumor cells***).

- It seems piezo1 levels are equivalent high to TRPV2 from the data screening (Fig. 1A), at least the author should discuss about the possible link between these two channels, as they both mechanical sensitive and calcium permeable.

PIEZO1 is a stretch-activated cation channel, whose activation can be exclusively achieved *via* mechanical forces applied to the cell plasma membrane (PM) (Coste *et al*, 2010), leading to a short activation phase that allows Ca^{2+} entry, which is followed by an inactivation phase that makes it insensitive to further stimulation (Poole *et al*, 2014). The role of PIEZO1 in cell migration is controversial, as some reports show that inhibition of PIEZO1 increases cell migration, such as in breast cancer, non-small cell lung carcinoma and neural crest cells (Canales Coutino & Mayor, 2021; Huang *et al*, 2019; Yu *et al*, 2021a), whereas others show inhibition in cell migration, such as in gastric cancer and glioma cells (Chen *et al*., 2018; Zhang *et al*, 2018). Canales Coutino & Mayor suggested that PIEZO1 has a conserved role in regulating FA dynamics, but whether this is translated into more or less migration depends on the cellular context.

In melanoma, we observed that Piezo1 is highly expressed in the SKCM tumors dataset (Figure 1A), while Piezo2 expression remains low. Consistently, Piezo1 has been shown to be upregulated in primary and metastatic melanoma compared to normal skin in TCGA, and to correlate with a shorter overall survival of melanoma patients (zhang *et al*, 2022). In this study, PIEZO1 promoted proliferation, invasion and metastasis. However, another study showed that PIEZO1 was important for adhesion maturation in normal cells but did not affect adhesion formation in transformed cells. In normal cells, PIEZO1 was enriched within mature and retracting adhesions, where it was bound to a complex with specific integrins but not adhesion proteins, and this localization was dependent on continued contraction forces. Meanwhile, in the A2058 melanoma cells, PIEZO1 did not localize to paxillin adhesions, suggesting that PIEZO1 functions in mechanosensing processes are distinctively regulated in normal *versus* cancer cells (Yao *et al*., 2020). In neural crest cells, PIEZO1 was further associated with FAs stabilization (Canales Coutino & Mayor, 2021).

Metastatic cells optimize their migratory potential by adapting their migration modes as they encounter different physical microenvironments. Efficient migration in confined spaces has been suggested to involve two independent confinement-sensing pathways: one mediated by PIEZO1/PDE1/PKA and the other by myosinII/Rac1, integrated through a complex feedback circuit (Hung *et al*, 2016). Upon physical confinement, the A375-SM invasive melanoma cancer cells optimize their motility notably *via* activating PIEZO1, which suppresses PKA and actomyosin contractility. In these melanoma cells, the depletion of PIEZO1 reduced only the confined migration. The cAMP-dependent protein kinase A (PKA), is known to play an important role in the migration of carcinoma cells and in the regulation of RhoA and Rac1 functions in several cooperative pathways (Newell-Litwa & Horwitz, 2011). Meanwhile PKA has also been shown to regulate TRPV2 trafficking to the PM (Stokes *et al*, 2004), and consequently its activation, suggesting that in a confined environment PIEZO1 could prevent TRPV2 translocation at the PM *via* the inhibition of PKA. Conversely, in migrating A375 melanoma cells, activation of Piezo1 increased the activation of the PI3K/AKT pathway (zhang *et al*., 2022), which regulates TRPV2 translocation to the PM (Nagasawa & Kojima, 2015). Hence, PIEZO1 could regulate TRPV2 activity, by either addressing it to the PM through the activation of the PI3K/AKT pathway, or impeding its trafficking to the PM *via* PKA inhibition. We speculate that both mechanosensitive channels, TRPV2 and PIEZO1, play an important role in the migration and invasion potentials of metastatic melanoma cells. The activation of these channels could be interconnected, either in an alternative or coordinate fashion, depending on the microenvironment

encountered by the cells. In this context, they could also engage cooperative functions, as TRPV2 is involved in nascent adhesion disassembly while PIEZO1 seems to preferentially stabilize mature FAs. Further investigations will determine whether, and how, these two mechanosensitive channels coordinate adhesion processes for metastatic melanoma cells to adopt the most appropriate migration mode, in order to achieve the fastest mobility. The connection between these two channels has been mentioned in the discussion of the manuscript (**page 21**).

Reviewer #2:

****Minor points:****

The authors should mention the published literature on the role of TRPM1 in melanoma (especially progression to metastatic disease) in their introduction.

In order to give a better overview of the literature on the role of TRPM1, as well as other TRP members, in the specific context of melanoma progression, we mentioned their roles in the Introduction **(pages 4 and 5).** More information can also be found in answer to the minor remark 1 of Reviewer#3**.**

Could the authors speculate on the apparent melanoma specificity of TRPV2 expression (figure 1)?

Several hypotheses can be raised regarding the predominant expression of TRPV2 in melanoma cells. For instance, it could result from **transcription factors** specifically expressed in the melanocytic lineage (such as MITF, SOX10, PAX3…) coupled to melanoma deregulated transcriptional pathways that would drive TRPV2 expression. This hypothesis will be further discussed in response to the major point 1.

Another explanation for this apparent specificity of melanoma cells could be related to the **neural crest origin of the melanocytes**. Indeed, parallels exist between the highly invasive nature of metastatic melanoma and their neural crest/melanoblast precursors. Multiple studies have suggested that melanoma reactivates neural crest migration programs to drive plasticity and invasiveness (Diener & Sommer, 2021; Sommer, 2011). Intriguingly, TRPV2 transcripts are present in pre-migratory neural crest cells from embryos (Hutson *et al*, 2017), and has been shown to regulate axon outgrowth through its activation by membrane stretch during development (Shibasaki *et al*, 2010). This hypothesis is not only linking TRPV2 expression to the neural origins of melanoma cells, but also to common functional features of these cells related to motility.

Finally, TRPV2-specificity in melanoma cells could also be associated with the specific type of migration these cells engage for **rapid plasticity**. As particularly invasive and disseminating cells, melanoma cells can adopt features of both mesenchymal and amoeboid migration (Gabbireddy *et al*, 2021). The mesenchymal-amoeboid transition (MAT) has been proposed as a mechanism for cancer cells to adapt rapidly their migration mode to their environment. This conversion to amoeboid, enabling a low-adhesive and energy-conserving migration strategy, has been described as involving calpain-mediated cleavage of talin in metastatic breast cancer and head and neck carcinoma cells, and therefore according to our mechanistic model might require TRPV2 activation (te Boekhorst *et al*, 2020). Note that this unique phenotypic switch from mesenchymal to fast amoeboid migration also occurs in immune cells, where TRPV2 is actually highly expressed (Liu *et al*, 2015). More specifically, TRPV2 has been involved in migration and chemotaxis in neutrophils and monocytes/macrophages (Santoni *et al*, 2013).

These hypotheses are not mutually exclusive and could actually be intertwined. Our *in vitro* and *in silico* data **(Figures S1E-F, S6** and **Review additional Figure 1)** are supporting an association of TRPV2 expression with a specific signature for cells endowed with unique plasticity potential involving amoeboid migration (de Winde *et al*, 2021; Rodriguez-Hernandez *et al*, 2020). This point is

further developed in response to the comment below regarding MITF/AXL phenotypic signatures. To further elaborate the manuscript, this hypothesis was briefly mentioned in the discussion (**page 20-21**). However, in-depth investigations would be required to bring a thorough answer to this compelling question.

Page 10, how was the invasiveness of melanoma cell lines measured? The authors should refrain from claiming that there is a "positive correlation between invasiveness and TRPV2 expression" (page 10) based on results from just 4 human melanoma cell lines. To support their claim, they would need to expand their panel of cell lines and/or use appropriate statistics (such as Pearson's correlation test).

The invasiveness of the cell lines used in the present study were initially relying on reports from the literature, where their invasive potentials was assessed *in vitro,* or *in vivo* based on their ability to metastasize (Arozarena & Wellbrock, 2017; Herlyn *et al*, 1990; Juhasz *et al*, 1993; Tichet *et al*, 2015). Our own *in vitro* (Figure 3A) and *in vivo* (Figure 6A,C) analysis confirmed the strong migrative/invasive potentials of the WM266.4 and 451Lu cells, as compared to the poorly invasive 501mel. Invasiveness was further assessed by measuring the levels of POU3F2/BRN2 (Figure 1G) considered as a marker of invasiveness (Arozarena *et al*, 2011b; Thomson *et al*, 1995), and the expression of active (non-phospho) b-catenin (Figure S6) described as a suppressor of invasion in melanoma (Arozarena *et al*, 2011a).
Moreover, and as suggested, we applied a Pearson's correlation test to the NHEM plus the 4 main cell lines used in this study (**Figure S1E**, $r_{pearson}=0.8946$, $p=0.0202$), as well as to the larger dataset of melanoma cell lines from the CCLE (**Figure S1F**, $r_{pearson}=0.3353$, $p=0.0019$), and showed a correlation between TRPV2 and POU3F2/BRN2 expressions.
For clarity purpose, we have modified the results section (**page 12**) as follow:
[While very low levels of TRPV2 mRNAs were present in normal human epithelial melanocytes (NHEM), a gradual increase of TRPV2 transcripts correlates with the rise of BRN2 expression in melanoma cells (ranging from the non-invasive 501mel, to the superficial spreading melanoma WM793, then to the metastatic melanoma WM266.4 and 451Lu *(Arozarena et al., 2011a; Arozarena et al., 2011b; Arozarena & Wellbrock, 2017; Herlyn et al., 1990; Juhasz et al., 1993; Tichet et al., 2015) (Fig 1G and S1E). In the broader CCLE melanoma cell lines dataset, the overall expressions of TRPV2 and POU3F2(BRN2) were also correlated (Fig S1F). In addition to TRPV2 mRNA expression, both TRPV2 protein levels (Figure 2A) and its functionality, assessed upon TRPV2 channel over-activation with the potent agonist cannabidiol (CBD) (Qin et al, 2008) (Figure S2A), mimicked the expression of the BRN2 invasiveness marker. Altogether suggesting a correlation between the expression of functional TRPV2 channels and the BRN2-associated invasive phenotype of melanoma cell lines.]*

Could the authors examine the potential correlation between TRPV2 expression and MITF and AXL levels, which are well-accepted markers of the balance between growth and migration in melanoma?

To rapidly adapt to the tumor microenvironment, melanoma cells can switch their molecular and cellular phenotype, including in an epithelial-to-mesenchymal transition (EMT)-like manner. Conventionally, phenotypic changes were linked to the expression levels of MITF (microphthalmia-associated transcription factor) and AXL, in order to regulate differentiation, proliferation and metabolic rewiring. MITF is a melanocyte lineage-specific transcription factor, considered to be the "master regulator of melanocytes" because it is essential for melanoblast survival and melanocyte lineage commitment, and it regulates the expression of melanogenic enzymes. MITF has been proposed as a read-out of the phenotype switching model, with MITF^{high} cells characterized as proliferative, and MITF^{low} as invasive (Hoek *et al*, 2008a), acting as a rheostat where the levels of MITF would determine the phenotypic state (Rambow *et al*, 2019). During the invasion process, melanoma cells exhibit a de-differentiated phenotype, characterized by low pigmentation and reduced proliferation, whereas upon metastatic growth, cells switch back to a differentiated, pigmented and proliferative phenotype. These

phenotypic switches, as well as MITF expression level changes are guided by the distinct tumor microenvironments.

Despite all the evidence suggesting that MITF must be down-regulated for melanoma progression, *MITF* amplification is frequent in melanoma and particularly common in metastatic forms of the disease where it is associated with poor survival (Garraway *et al*, 2005). The MITF gene is actually amplified in 10–15% of melanomas in which BRAF is mutated, as it is the case for the 451Lu invasive cell line (Garraway *et al.*, 2005; Wellbrock *et al*, 2008). Moreover, these reports showed that oncogenic BRAF stimulates MITF transcription in a BRN2-dependent manner, and further correlated BRN2 and MITF expression in human melanoma samples. The relationship between MITF and BRN2 seems complex, since BRN2 has also been described as expressed in a predominantly mutually exclusive pattern with MITF. Of note, BRN2 is expressed in melanoma but not in melanocytes. In line with the previous reports, while MITF was required to maintain proliferation *in vitro*, both MITF and BRN2 expression have been shown necessary for the development of melanoma metastasis *in vivo* (Simmons *et al*, 2017). Finally, a considerable fraction of circulating tumor cells isolated from invasive melanoma patients was MITF positive (Khoja *et al*, 2014), supporting the view that continued expression of MITF is essential in metastatic melanoma cells. Hence, the connection between MITF and melanoma invasiveness might not be as straightforward as anticipated.

We analyzed MITF/AXL expression levels in 4 subpopulations of melanoma cells issued from human biopsies, classified according to their differentiation states (Tsoi signatures). The 4 phenotypes, namely, undifferentiated, neural crest like, transitory and melanocytic, can be defined by the expression of a defined set of genes, notably the so-called “MITF/AXL rheostat” **(Review additional Figure 1A)**. As expected, undifferentiated and neural crest melanomas were $MITF^{low}/AXL^{high}$, whereas the opposite was seen in the 2 further differentiated subpopulations. Regarding TRPV2, although present in all 4 melanoma subtypes, its expression was slightly higher in the population with the melanocytic signature ($MITF^{high}/AXL^{low}$). Correlation analysis in the TCGA SKCM dataset further showed that TRPV2 expression was positively correlated to MITF expression, and rather negatively to AXL expression **(Review additional Figure 1B)**. As we mentioned in the discussion (**page 20**), while 501mel cells exhibit pseudo-epithelial markers (including MITF), and inversely WM266.4 display mesenchymal markers and a morphology evoking a complete mesenchymal transition, 451Lu cells were associated with an intermediate/partial phenotype, such as less viculin-containing adhesions, less N-cadherin and less vimentin, and a high MITF expression (**Figures 5A and S6**). In line with this, it has been described that in cancer cells the amoeboid state is supported by both proliferative and pro-survival signaling (Graziani *et al*, 2022), with some amoeboid cells expressing concomitantly MITF and BRN2 (de Winde *et al.*, 2021). Interestingly, the highly invasive 451Lu cells, exhibiting a unique migratory phenotype with fewer engaged adhesions, are expressing extremely high levels of TRPV2. Sommer and colleagues further showed that in melanoma cells, NGFR overexpression promotes metastasis formation, but is associated with a gene expression program that is not overlapping with the previously established MITF/AXL expression signatures defining proliferative *versus* invasive cells (Restivo *et al*, 2017). They concluded that these processes were either independent of previously reported invasive transcriptional programs or were representative of transient states of cells undergoing dynamic switching. Later on, they reported that the down-regulation of SMAD7, a major negative regulator of the TGF-β signaling pathway, can overcome the need for the “phenotype switching” during melanoma tumor progression, since conditional *Smad7* deletion led to sustained melanoma growth coupled to metastasis formation (Tuncer *et al*, 2019). Importantly, low SMAD7 levels in patient tumors are associated with poor survival. Interestingly, our correlation analysis in the TCGA SKCM dataset revealed that SMAD7 expression is negatively correlated to both TRPV2 and MITF expressions **(Review additional Figure 1C)[Figures for referees not shown.]** .

Hence, contrary to the conventional “phenotype switching” model, melanoma progression can involve malignant melanoma cells simultaneously displaying proliferative and invasive properties, presenting unique migratory signatures, most likely accounting for the intrinsic cellular plasticity and

heterogeneity of melanomas (Kemper *et al*, 2014; Tirosh *et al*, 2016). Conceivably, TRPV2 expression together with MITF expression may be associated with melanomas unique cellular plasticity.

How does TRPV2 overexpression in Figure 2C compare with normal expression levels in invasive cell lines?

When we compared TRPV2 expression at the protein level within the different cell lines, we observed a very similar level of expression between 501mel-TRPV2-GFP and the invasive 451Lu cells while 501mel-GFP were barely expressing TRPV2 (**Figure S6**).

Were the FA analyses presented in Figure 5 performed on fibronectin-coated plates? Please consider adding these details in the figure legends.

Analysis of mature FA numbers was indeed performed on low confluence melanoma cells seeded onto fibronectin-coated coverslips. We apologize for the oversight and amended the legend of **Figure 5** accordingly.

Could the authors provide more explicit statistical analyses on Figures 6 and 7D?

We apologize for the oversight and we have included statistical analyses on Figure 6B,D (unpaired t-test) and 7D (Anova multiple comparison). Please see also the above response to Reviewer#1's comments.

How do the authors explain that TRPV2 colocalizes with paxillin and not phospho-FAK (in contrast to what is depicted on Figure 7G)?

In the invasive melanoma models, we indeed observed clusters of TRPV2 and Paxillin (Pax), but could not detect any colocalization of the channel with pFAK.

Pax is one of the most important proteins for structure and function of integrin-mediated adhesions. As a scaffolding protein, Pax is recruited within early adhesomes at the cell front for the assembly of nascent adhesion complexes by integrin-activators such as Talin, to ultimately lead the adhesion dynamics and cytoskeleton reorganization (Lopez-Colome *et al*, 2017). Pax is also required for the disassembly of FAs at the rear end of the cell. Interactions of Pax with its binding partners are mostly regulated by its phosphorylation status at multiple Tyr and Ser residues. In adhesion sites, p-FAK preferentially complexes with phosphorylated Y31 and Y118-Pax in a periodic pattern (Choi *et al*, 2011; Digman *et al*, 2008), forming very dynamic, force-sensitive, confined and discrete clusters. The remaining adhesion area containing Pax organizes pFAK-independent signaling and adhesion (Bachmann *et al*, 2020). Hence, although FAK is recruited from the cytoplasm to adhesion sites by Pax, Pax interaction with and phosphorylation by FAK is not essential for Pax localization to adhesion sites (Brown *et al*, 1996). In fact, the binding site for FAK is located at the N-term domain, whereas the sequence for localization at adhesions resides in the C-term region of Pax. Studies on the hierarchical assembly of adhesions have shown that the structure of the adhesive platforms may vary according to the distinct combinations of integrin receptors activating specific signaling pathways (Zaidel-Bar *et al*, 2003). Nevertheless, Pax recruitment to the leading edge of membrane protrusions is an early event in the adhesive process (Laukaitis *et al*, 2001; Petropoulos *et al*, 2016).

However we can not exclude that few engaged adhesion structures encompassing TRPV2-Pax-pFAK complexes, if present, were too discrete and dynamic to be detected without super-resolution microscopy techniques. This might explain the very sparse colocalization spots observed in the highly invasive 451Lu cells (Figure 4B h). Although FAK/p-FAK and Src/p-Src are highly expressed in both metastatic cell lines (WM266.4 and 451-Lu) as compared to the nonmetastatic 501mel cells, the modulation of TRPV2 expression (either overexpressed or silenced) did not affect both kinases expression (own unpublished data), further suggesting that, at least at this stage, Pax does not require a p-FAK-dependent signaling. Hence, based on the literature, we concluded that the co-clustering of TRPV2 and Pax observed in migrating melanoma cells occurs within nascent adhesion sites, which at this early stage does not involve a stable interaction with pFAK. The simplified schematic model in **Figure 7G** has been modified accordingly.

The mechanosensitive nature of TRPV2 is not addressed experimentally in this manuscript. Please state published results more explicitly when referring to this aspect of TRPV2 activity regulation (for example on pages 13 and 19).

We apologize for this lack of clarity. The mechanosensitive properties of TRPV2, although well described in the literature as one of its specificity (among the main references: (Katanosaka *et al*, 2018; Nagasawa & Kojima, 2015; Sugio *et al*, 2017; Yu *et al*, 2021b), has indeed not been addressed here in the context of melanoma progression. This aspect is undoubtedly of great interest, but requires highly specific approaches. Hence, the text throughout the manuscript has been clarified accordingly.

Reviewer #3:

****Minor remarks:****

1. A description of the TRP family in the introduction would be helpful: How is the TRPV2 channel different from other family members? Which normal tissues is TRPV2 highly expressed? Most importantly, why did the authors decide to focus on TRPV2 since several other TRP proteins are highly expressed in melanomas including TRPM1 and TRPM7.

A brief presentation of the TRP channels superfamilies, as well as the known roles of TRP members, such as TRPM1 and TRPM7, in melanoma progression has been added to the Introduction **(pages 4 and 5)**. With regards to TRPV2 specificity towards the other members of the TRP family and its tissue-specific expression, we have added this information in the discussion of the manuscript **(page 19)**. However, to clarify the specific interest shown towards TRPV2, we believe it was necessary to further develop our rationale here. Indeed, in our analysis of Ca^{2+}-permeable channels mRNA levels in SKCM tumors (Figure 1A), the expression of other TRP members, such as TRPM1 and TRPM7 were substantial, although lower than TRPV2 expression.

TRPM1 (where M stands for Melastatin), the founding member of the TRPM channels, is indeed described as important in melanocyte function and malignant melanoma pathophysiology. However TRPM1-expression correlates positively with the differentiation status of melanocytes and, inversely, with the aggressiveness and tumor thickness of malignant melanoma (even used as a good prognostic marker). TRPM1 expression was found to correlate with melanin content (Oancea *et al*, 2009) but downregulated in melanoma (Figure S1A). In conjunction with TRPM1, a microRNA (miR-211) located within the TRPM1 gene is also described as playing an important role in tumor suppression (Boyle *et al*, 2011; Mazar *et al*, 2010). Taken together these data were not in favor of TRPM1 as a Ca^{2+}-regulating element of advanced melanoma progression.

Regarding TRPM7, it is the best studied TRP channel in the context of metastasis, and its overexpression has been observed in primary breast cancer tumors (Middelbeek *et al*, 2012), in ductal adenocarcinoma (Guilbert *et al*, 2009) and ER negative invasive ductal cancer (Guilbert *et al*, 2013), in malignant pancreatic tumors (Rybarczyk *et al*, 2017; Yee *et al*, 2015), in metastatic nasopharyngeal tumors (Chen *et al*, 2015) and in bladder cancer (Gao *et al*, 2017). Elevated TRPM7 expression was associated with poor prognosis and metastasis in most of these cancer types. Concerning melanoma tumors, in our study, a high expression of TRPM7 was indeed observed when querying tumors from the SKCM data set (Figure 1A), yet the qPCR analysis on metastatic melanoma cell lines showed little expression of TRPM7 especially in comparison to TRPV2 expression (over 10 times less, Figure 1C). Consistently, TRPM7 expression has been shown overall steady in both melanocytes and melanoma cells, but most importantly did not increase together with the invasive phenotypes of melanoma cell lines (McNeill *et al*, 2007). Additionally, *in silico*, TRPM7 expression did not come out as a poor prognosis factor in melanoma tumors (data not shown). As tumor biopsies consist of cancer cells as well as immune cells, CAFibroblast and cells from the tumoral microenvironment, we hypothesized that TRPM7 was mostly overexpressed in other cell types than in melanoma cells, presumably in fibroblast. Moreover, as our initial goal was to identify a therapeutic target specific for the treatment

of melanoma, we did not select TRPM7 as a potential candidate because of its ubiquitous expression. In any event, we firmly believe that studying the role played by TRPM7, as well as several other TRP family members, in advanced melanoma progression would be of great interest.

As for the reasons why we have focused on TRPV2, not only it was the most expressed channel in human SKCM tumors (Figure 1A), and its overexpression was specific to melanoma lesions compared to nevi samples **(Fig S1A)**, but also in metastatic melanoma cell lines TRPV2 expression was up to 12 times more elevated compared to other TRP members (Figure 1B-C), and finally its overexpression largely prevailed in melanoma in comparison to several other cancer types (Figure 1D-E and S1B-C). Although the mechanistic basis for TRPV2 mechanotransduction potential was up to now unexplained, the mechanosensitive properties of this channel are recognized as one of its specificity (Katanosaka *et al.*, 2018; Nagasawa & Kojima, 2015; Sugio *et al.*, 2017; Yu *et al.*, 2021b). The few studies reporting a role for TRPV2 in cancer progression were correlating its expression and translocation to the cell membrane (a regulatory mechanism that is specific to TRPV2 (Kojima & Nagasawa, 2014)) with tumor cells migration (Monet *et al*, 2009; Monet *et al*, 2010; Oulidi *et al*, 2013), making this mechanosensitive channel a good target for the acquisition of the metastatic potential in melanoma. Additionally, its closest phylogenetic family member, TRPV1, is described as a valuable therapeutic target in pain treatment, with specific pharmacological tools developed that have entered clinical trials, supporting a promising potential for the development of therapeutic treatment targeting TRPV2. Understanding the role of TRPV2 in regulating cell migration in the tumoral cell type exhibiting its highest level of expression, could eventually turn out beneficial for the treatment of the other cancer types involving TRPV2, including prostate and breast cancers.

3. Figure 2A: Why is the upper band of different sizes? Has the TRPV2 channel been glycosylated differently in different cell lines? Or do these represent different splice forms?

According to the size of the multiple bands observed on TRPV2 immunoblots, the upper bands correspond to glycosylated forms of TRPV2. It has been reported that both high mannose-type glycosylation and complex glycosylation of TRPV2 occur (Jahnel *et al*, 2003). Overexpression recombinant TRPV2 in HEK cells further confirmed that anti-TRPV2 immunoreactivity appears as doublet, with similar size as those observed in metastatic melanoma cell lines (Figure S12A). In melanoma cells as well as in leukemia cells, TRPV2 upper band can be actually composed of several bands, likely corresponding to variable glycosylation patterns of TRPV2, differing according to the cell lines (Figure 1E). Interestingly, studies have suggested that TRPV2 glycosylation plays a role in its trafficking towards the plasma membrane and would play an anchoring role (Jahnel *et al.*, 2003), which perfectly correlate with our cell-surface biotinylation assays where the upper glycosylated bands were majoritarily detected in the plasma membrane fraction (Figure 2A).
Up to now, only short-splice variants of TRPV2, s-TRPV2, have been reported. These short variants have been found in normal human urothelial cells and bladder tissue specimens (where it was lost as an early event in bladder carcinogenesis) (Caprodossi *et al*, 2008), as well as in human macrophages (Nagasawa *et al*, 2007). They both lack the pore-forming region and the sixth transmembrane domains, where the glycosylation site is. In our own unpublished data, we have observed that s-TRPV2 variants mRNAs are barely expressed in melanoma cells.

4. Statistical significance is not shown changes in figure S3. Thus, the claim on page 11 "Although, TRPV2 overexpression increased the growth rate of the non-invasive 501mel cells, TRPV2 silencing had no impact on the viability nor on ERK phosphorylation in either metastatic cell lines, suggesting that TRPV2 is dispensable for malignant melanoma proliferative/survival (Figures S3A-B)" is not validated.

We apologize for the oversight and we have now included statistical analyses (2way ANOVA) in **Figure S3**. A statistically significant difference was only seen when comparing the 501mel-GFP cells to their counterpart overexpressing GFP-TRPV2 (P=0.0038). As for the invasive 451Lu cells, the tenuous variations between the sh-Ctrl and both sh-TRPV2 expressing cells did not reach significance (P=0.9982 for sh-V2-1, and P=0.5755 for sh-V2-2). Finally, regarding the WM266.4 cells, despite no significant variations, we wondered if the seeding density used in these experiments was optimal considering the rather slow slope curves. We therefore undertook additional experiments to improve the quality of these data, and will update the final version of the manuscript accordingly. Moreover, we would like to highlight the fact that (as stated in the manuscript) no significant difference was observable in the size of the melanospheres formed by the WM266.4 cells, whether they were expressing TRPV2 or not (Figure 3D-E and S4C).

5. Did OE and KD TRPV2 show changes in cell morphology?

Although we did not perform a thorough morphological analysis of the melanoma cell lines used in this study, we could observe very subtle alterations in their apparent shape upon either TRPV2 expression or silencing. For instance, when overexpressing TRPV2, the 501mel cells seem to display more protrusions (dendritic-like) compared to the control cells which have a more classical "flat polygonal" shape. But this has to be properly analyzed and quantified. Regarding the WM266.4 and 451Lu cells, despite both having highly invasive potential, they display distinct morphological features. WM266.4 are well spread polarized cells with protrusions and forming monolayers in culture, while 451Lu are very heterogeneous, some rounder, and with a propensity to grow in a clonal-type manner forming multilayers clusters, which is once again fitting with their intermediate mesenchymal-amoeboid phenotype (described in the discussion **page 20** and above in response to a couple of minor remarks from Reviewer#2). While beyond the scope of this manuscript, an in-depth morphological analysis of melanoma cells, coupled to investigations of their morphologic and phenotypic migration features depending on the substrate, would definitely widen our understanding of the role played by TRPV2 in regulating melanoma metastatic migration.

6. The authors should discuss why the mice injected with GFP-TRPV2 overexpressing cells showed numerous metastatic foci in lungs, brain and bones, whereas, "TRPV2 repression prevented the extravasation of melanoma cells into the lungs" only?

When comparing the *in vivo* metastatic potentials of the 501mel cells overexpressing TRPV2-GFP with the 451Lu (endogenously highly expressing TRPV2), we indeed noticed that metastasis formation preferentially occurred in the lungs of 451Lu-injected mice, while mainly occurring in both the lungs and the bones of 501mel-TRPV2-GFP-injected mice (Figures 6 and S9B).

Following the injection in the tail vein, cells are rapidly reaching the heart and then the lungs. At this stage, depending on their capacity to cross barriers and therefore invasive potentials, they may extravasate to form distant metastasis. Due to the experimental model, the lungs will therefore be a preferential site for extravasation and metastatic development. Moreover, we speculate that the 451Lu cells display a strong tropism for the lungs due to tumor-derived intrinsic properties of these cells. Indeed, 451Lu (where Lu stands for Lung) melanoma cells were selected from lung metastases of xenografted nude mice after several *in vivo* passages of human melanoma WM164 cells, previously isolated from a stage IV melanoma metastasis of a patient (Herlyn *et al.*, 1990). During the selection process while accumulating enhanced invasiveness, 451Lu cells might have also acquired a high tropism for the lung. Note that, although to a lower extent, 451Lu also metastasized in other organs of the mice.

By contrast, 501mel are melanoma cells of unknown primary histologic origins endowed with very low invasive potentials. Their intrinsic properties in terms of crossing endothelial barriers, intravasation, extravasation and so forth are therefore likely limited. Among the 501mel-TRPV2-injected mice that developed metastasis, foci were localized in the lungs, but also in the bones and

other organs. For instance, bones could provide a propitious microenvironnement for TRPV2-overexpressing cells to migrate to, due to a high extracellular Ca^{2+} concentration.

7. Page 15 : "The TCGA skin melanoma RNAseq dataset was first compared to the matched TCGA and GTEx normal datasets (Figure S13A)." The authors should clarify which subset of the GTEx normal dataset was used.

We apologize for the lack of clarity. The analyses presented in **Figure S13A** were done using the GEPIA online server (http://gepia2.cancer-pku.cn/), providing tools for differential expression analysis using TCGA tumor samples with paired adjacent TCGA normal samples and GTEx normal samples. To integrate the TCGA and GTEx data for comprehensive expression analysis, this server uses UCSC Xena project recomputed TCGA and GTEx raw RNA-Seq data, which makes the two datasets compatible. The normal skin GTEx dataset used by GEPIA includes RNA sequencing data of 557 samples from a non-specified subset, knowing that the GTEx Analysis Release V8 (dbGaP Accession phs000424.v8.p2) encompasses two distinct normal skin subsets: Not sun-exposed (suprapubic), n=604, and sun-exposed (lower leg) n=701. In any event, the median log2(TPM+1) values for TRPV2 expression in these two subsets were 2.86 and 3.11, respectively. Hence, despite a slight increase in sun-exposed *versus* not sun-exposed skin, TRPV2 expression level remains significantly higher in SKCM tumors (median log2(TPM+1)=6.3, Figure S13A).
Since melanocytes represent only a small cellular fraction of normal skin, the choice of the "normal skin" dataset is of high importance and could definitely impact the conclusions. In order to strengthen our data, we have included analyses of TRPV2 expression based on the GSE46517 microarray gene expression dataset (**Figures S13B**) including human samples from normal skin, nevi, primary and metastatic melanoma tumors (Kabbarah *et al*, 2010). The results confirmed TRPV2 overexpression in melanomas compared to normal skin, but most importantly showed that in melanocytes-composed benign nevi, TRPV2 expression remained low, significantly below the level of expression observed in melanomas.

8. Page 25: Figure 1 legends: add T to the at the beginning of the sentence "The Ca2+- permeable channels plotted on the x-axis are grouped by family...."

We have meticulously revised the manuscript and all the following suggested modifications in point 8 and 9 have been included.

9. English needs to be improved throughout the manuscript. A few examples are listed below:

a.Abstract: „...is of primary importance (since being) AS IT IS the main cause..."

b.Abstract: „ Here, we (evidenced a) SHOW THE prominent expression...."

c.Introduction: „..The scope of this study was to identify AN ATYPICAL PROFILE among the numerous...."

d.Materials and Methods: Mice model should be Mouse models.

e.Results: „TRPV2 TRANSCRIPTS STOOD OUT AS THE MOST EXPRESSED among (most members of) the major Ca2+ permeable channels...."

f.„Importantly, the (utmost expression of) TRPV2 transcripts (was) WERE (also revealed) SHOWN TO BE HIGHLY EXPRESSED in SKCM tumours AS compared to...."

g.Page 12: The sentence: "In these define proximal clusters..." Needs to be rearranged or clarified.

h.Discussion: „Nevertheless, modulating TRPV2 expression - either way - had no impact on THE EXPRESSION OF EMT markers.„

i.Discussion: „..the highly invasive WM266.4 cells (were) exhibitED antagonistic markers profiles...."

j.Discussion: „These observations were (in adequation) CONSISTENT with .....as well as with (their levels of) THE EXPRESSION OF THE BRN2 invasiveness marker."

k.The sentence „Therefore, globally impacting resting...." lacks an ending.

l.„(By being recruited) RECRUITMENT OF TRPV2 at the PM within paxillin-rich.....structures PLACES THIS mechanosensitive channel at...."

4. Description of analyses that authors prefer not to carry out

Please include a point-by-point response explaining why some of the requested data or additional analyses might not be necessary or cannot be provided within the scope of a revision. This can be due to time or resource limitations or in case of disagreement about the necessity of such additional data given the scope of the study.

Reviewer #2:
Referees cross-commenting
I think that the points raised by the other 2 reviewers are well taken. **My major point 1 could be disregarded if the other reviewers feel that it is beyond the scope of the paper.** However, I still feel that the paper could be stronger if the authors more formally proved the proposed mechanism of action of TRPV2 (major point 2).

*****Major points:*****
1)*The gene expression data and TMA staining suggest that high TRPV2 is associated with advanced melanoma. Is the TRPV2 locus altered at the genomic level (by mutation or copy-number alteration)? Could the authors investigate the mechanism of TRPV2 overexpression in melanoma compared to normal melanocytes or benign nevi? Knowing more about the regulatory elements in the TRPV2 promoter or enhancer regions and/or about the regulators of TRPV2 stability/cell surface localization would add considerable value to this study.*

We fully agree with Reviewer#1, understanding the regulation of TRPV2 at the genomic level, but also the regulation of its stability and subcellular localization would be of considerable value.

To tackle this compelling point, we performed data set mining using cBioportal (https://www.cbioportal.org/) and determined whether the TRPV2 locus is altered at the genomic level (**Review additional figure 2A-C**). It appeared that mutations or copy-number (CN) alterations of the TRPV2 gene are rare events in melanoma, suggesting that TRPV2 overexpression in melanoma do not stem from CN-amplification. Hence, the exacerbated expression of TRPV2 in melanoma compared to benign nevi and to other cancer types, rather suggests that some melanocytic lineage-specific transcription factors may be at play (*e.g.* SOX10, MITF, PAX3…), in combination with transcriptional regulators and alterations that drive melanoma progression, especially those supporting a pro-invasive/pro-metastatic behavior such as BRN2 (see also above responses to reviewer#2's minor points). Favoring this hypothesis, TRPV2 expression in metastatic melanoma is positively correlated with both MITF and BRN2 (**Figures 1G and S1E-F** and **Review additional Figure 1**). Consistent with a potential regulation of TRPV2 expression by MITF is the fact that TRPV2 expression is induced following MITF overexpression in both the SK-MEL-28 and A375 melanoma cell lines (see Supplemental Data 1 in (Hoek *et al*, 2008b) and Supplementary file 1 in (Dilshat *et al*, 2021)). Yet, the opposite trend was not observed upon MITF invalidation or knock-down, suggesting an indirect regulation of TRPV2 *via* MITF. Mechanistically, it appears that neither MITF nor BRN2 may directly activate TRPV2 expression, as our analyses of the promoter region of TRPV2 using publically available ChIP-seq data did not show any potential binding sites for either transcription factor in melanoma cell lines.

However, we searched for melanoma cell lines with strong TRPV2 expression based on single cell RNAseq data (Wouters *et al*, 2020) and analyzed the corresponding H3k27ac tracks (Verfaillie *et al*, 2015) for predicted transcription factor binding sites (JASPAR database). This approach revealed multiple TFAP2A sites into the putative proximal TRPV2 enhancer region. The TFAP2A gene codes for the AP-2α transcription factor which controls neural crest differentiation and development. Interestingly, TFAP2A is strongly expressed in neural crest cells migrating from the cranial folds during neural tube closure and has been shown to cooperate with MITF to drive melanocyte differentiation

(Seberg *et al*, 2017). In this context, Seberg et al. performed an integrated analysis of both TFAP2A and MITF ChIP-seq with H3K27ac ChIP-seq data from human melanocytes that predicted an active TFAP2A/MITF shared peak associated with the TRPV2 gene (see table S7 in (Seberg *et al.*, 2017)). TFAP2A was also identified as part of a core melanoma-specific molecular signature (Rambow *et al*, 2015) and, later on, showed to be a transcription factor enriched in melanocytic and "intermediate" states melanoma cells (Wouters *et al.*, 2020) consistent with the higher expression of TRPV2 observed in these two MITF^{high} subpopulations (**Review additional Figure 1A)**.

Interestingly it has been recently shown that TFAP2A/AP-2α could facilitate melanoma metastasis formation (White *et al*, 2021) and that TFAP2A is one of the gene of the so-called "MITF program" overexpressed in the SMAD7^{low} signature that overcome the need for phenotype switching to promote metastatic progression (Tuncer *et al.*, 2019). Altogether this suggests that TRPV2 could be under the transcriptional control of TFAP2A and link TFAP2A to invasiveness. Yet, while gene correlation analyses show a positive correlation between TRPV2 and TFAP2A expression in both melanoma cell lines and tumors (**Review additional figure 2D**)[Figures for referees not shown.] , the values of the Pearson's correlation coefficient are rather low and suggest that the regulation of TRPV2 is more complex and possibly dependent on additional factors, such as cofactor availability or chromatin accessibility.

Hence, determining the exact transcription factors combination, epigenetic regulation, non-genomic regulation or tumor microenvironmental factors leading to high expression/activity of TRPV2 and promoting migration/invasion independently of the phenotype switching, while of great interest, is beyond the scope of this manuscript and to be honest beyond our fields of expertise.

Minor points:

Could the authors also show talin and TRPV2 staining in Figure 4?

As stated above, although Talin is a central mechanosensitive adaptor protein in early integrin-mediated contacts, Talin cleavage was initially used as a second-line readout of calpain activity, and we have unfortunately not performed any Talin immunostaining.

Talin participates very early in adhesomes formation and is an important calpain-2 target to induce adhesion turnover (Critchley & Gingras, 2008; Klapholz & Brown, 2017). However, other calpain substrates than Talin, such as Paxillin and Filamin A, another cell motility modulator (Lamsoul *et al.*, 2020) could be concomitantly involved in melanoma cells migration. In order to deepen our understanding of the mechanistic events downstream of calpain activation, the analysis of several proteolytic targets of calpains should be considered. Analysis of their cleavage status, along with high-resolution microscopy approaches to define their precise subcellular localization, and whether these substrates belong to nascent adhesion sites, would be highly informative.

Reviewer #3:

Minor remarks:

2. Figure 2A: It would be better to include a cell surface protein as loading control on this Western blot.

A cell surface protein as a loading control would have indeed been ideal. Among the most common normalizing membrane proteins are beta-catenin and N-cadherin. However, as both proteins are involved in adhesion, their levels of expression are very different in the various melanoma cell lines we have tested (Figure S6). The Na^+/K^+-ATPase pump is another classically used plasma membrane loading control. While present in melanoma, its expression level is highly variable and depends on disease progression (Boukerche *et al*, 2004; Mathieu *et al*, 2009). Conceivably due to the heterogeneity of the melanoma cells, we have been unable to find an ubiquitous cell surface protein, whose expression level is consistent regardless of the cell types tested. This is why we have opted for Actin as loading and cell integrity control in these experiments, where as expected actin is absent from the cell surface proteins fractions. Please note that the cell surface biotinylation assay is a robust technique to isolate and compare the amount of cell surface proteins from samples with an equivalent quantity of total protein and that the observations made with this method were confirmed using two alternative approaches namely, immunostaining and channel activity assays.

References :

Arozarena I, Bischof H, Gilby D, Belloni B, Dummer R, Wellbrock C (2011a) In melanoma, beta-catenin is a suppressor of invasion. *Oncogene* 30: 4531-4543

Arozarena I, Sanchez-Laorden B, Packer L, Hidalgo-Carcedo C, Hayward R, Viros A, Sahai E, Marais R (2011b) Oncogenic BRAF induces melanoma cell invasion by downregulating the cGMP-specific phosphodiesterase PDE5A. *Cancer Cell* 19: 45-57

Arozarena I, Wellbrock C (2017) Targeting invasive properties of melanoma cells. *FEBS J* 284: 2148-2162

Bachmann M, Skripka A, Wehrle-Haller B, Bastmeyer M (2020) Phosphorylated paxillin and FAK constitute subregions within focal adhesions. *bioRxiv*: 2020.2009.2024.311126

Boukerche H, Su ZZ, Kang DC, Fisher PB (2004) Identification and cloning of genes displaying elevated expression as a consequence of metastatic progression in human melanoma cells by rapid subtraction hybridization. *Gene* 343: 191-201

Boyle GM, Woods SL, Bonazzi VF, Stark MS, Hacker E, Aoude LG, Dutton-Regester K, Cook AL, Sturm RA, Hayward NK (2011) Melanoma cell invasiveness is regulated by miR-211 suppression of the BRN2 transcription factor. *Pigment Cell Melanoma Res* 24: 525-537

Brown MC, Perrotta JA, Turner CE (1996) Identification of LIM3 as the principal determinant of paxillin focal adhesion localization and characterization of a novel motif on paxillin directing vinculin and focal adhesion kinase binding. *J Cell Biol* 135: 1109-1123
Canales Coutino B, Mayor R (2021) The mechanosensitive channel Piezo1 cooperates with semaphorins to control neural crest migration. *Development* 148
Caprodossi S, Lucciarini R, Amantini C, Nabissi M, Canesin G, Ballarini P, Di Spilimbergo A, Cardarelli MA, Servi L, Mammana G *et al* (2008) Transient receptor potential vanilloid type 2 (TRPV2) expression in normal urothelium and in urothelial carcinoma of human bladder: correlation with the pathologic stage. *Eur Urol* 54: 612-620
Chen JP, Wang J, Luan Y, Wang CX, Li WH, Zhang JB, Sha D, Shen R, Cui YG, Zhang Z *et al* (2015) TRPM7 promotes the metastatic process in human nasopharyngeal carcinoma. *Cancer Lett* 356: 483-490
Chen X, Wanggou S, Bodalia A, Zhu M, Dong W, Fan JJ, Yin WC, Min HK, Hu M, Draghici D *et al* (2018) A Feedforward Mechanism Mediated by Mechanosensitive Ion Channel PIEZO1 and Tissue Mechanics Promotes Glioma Aggression. *Neuron* 100: 799-815 e797
Chen YF, Chiu WT, Chen YT, Lin PY, Huang HJ, Chou CY, Chang HC, Tang MJ, Shen MR (2011) Calcium store sensor stromal-interaction molecule 1-dependent signaling plays an important role in cervical cancer growth, migration, and angiogenesis. *Proc Natl Acad Sci U S A* 108: 15225-15230
Choi CK, Zareno J, Digman MA, Gratton E, Horwitz AR (2011) Cross-correlated fluctuation analysis reveals phosphorylation-regulated paxillin-FAK complexes in nascent adhesions. *Biophys J* 100: 583-592
Coste B, Mathur J, Schmidt M, Earley TJ, Ranade S, Petrus MJ, Dubin AE, Patapoutian A (2010) Piezo1 and Piezo2 are essential components of distinct mechanically activated cation channels. *Science* 330: 55-60
Critchley DR, Gingras AR (2008) Talin at a glance. *J Cell Sci* 121: 1345-1347
D'Souza RS, Lim JY, Turgut A, Servage K, Zhang J, Orth K, Sosale NG, Lazzara MJ, Allegood J, Casanova JE (2020) Calcium-stimulated disassembly of focal adhesions mediated by an ORP3/IQSec1 complex. *Elife* 9
de Winde CM, George SL, Crosas-Molist E, Hari-Gupta Y, Arp AB, Benjamin AC, Millward LJ, Makris S, Carver A, Imperatore V *et al* (2021) Podoplanin drives dedifferentiation and amoeboid invasion of melanoma. *iScience* 24: 102976
Diener J, Sommer L (2021) Reemergence of neural crest stem cell-like states in melanoma during disease progression and treatment. *Stem Cells Transl Med* 10: 522-533
Digman MA, Brown CM, Horwitz AR, Mantulin WW, Gratton E (2008) Paxillin dynamics measured during adhesion assembly and disassembly by correlation spectroscopy. *Biophys J* 94: 2819-2831
Dilshat R, Fock V, Kenny C, Gerritsen I, Lasseur RMJ, Travnickova J, Eichhoff OM, Cerny P, Moller K, Sigurbjornsdottir S *et al* (2021) MITF reprograms the extracellular matrix and focal adhesion in melanoma. *Elife* 10
Gabbireddy SR, Vosatka KW, Chung AJ, Logue JS (2021) Melanoma cells adopt features of both mesenchymal and amoeboid migration within confining channels. *Sci Rep* 11: 17804
Gao SL, Kong CZ, Zhang Z, Li ZL, Bi JB, Liu XK (2017) TRPM7 is overexpressed in bladder cancer and promotes proliferation, migration, invasion and tumor growth. *Oncol Rep* 38: 1967-1976
Garraway LA, Widlund HR, Rubin MA, Getz G, Berger AJ, Ramaswamy S, Beroukhim R, Milner DA, Granter SR, Du J *et al* (2005) Integrative genomic analyses identify MITF as a lineage survival oncogene amplified in malignant melanoma. *Nature* 436: 117-122
Glading A, Lauffenburger DA, Wells A (2002) Cutting to the chase: calpain proteases in cell motility. *Trends Cell Biol* 12: 46-54
Graziani V, Rodriguez-Hernandez I, Maiques O, Sanz-Moreno V (2022) The amoeboid state as part of the epithelial-to-mesenchymal transition programme. *Trends Cell Biol* 32: 228-242

Guilbert A, Gautier M, Dhennin-Duthille I, Haren N, Sevestre H, Ouadid-Ahidouch H (2009) Evidence that TRPM7 is required for breast cancer cell proliferation. *Am J Physiol Cell Physiol* 297: C493-502

Guilbert A, Gautier M, Dhennin-Duthille I, Rybarczyk P, Sahni J, Sevestre H, Scharenberg AM, Ouadid-Ahidouch H (2013) Transient receptor potential melastatin 7 is involved in oestrogen receptor-negative metastatic breast cancer cells migration through its kinase domain. *Eur J Cancer* 49: 3694-3707

Herlyn D, Iliopoulos D, Jensen PJ, Parmiter A, Baird J, Hotta H, Adachi K, Ross AH, Jambrosic J, Koprowski H *et al* (1990) In vitro properties of human melanoma cells metastatic in nude mice. *Cancer Res* 50: 2296-2302

Hoek KS, Eichhoff OM, Schlegel NC, Dobbeling U, Kobert N, Schaerer L, Hemmi S, Dummer R (2008a) In vivo switching of human melanoma cells between proliferative and invasive states. *Cancer Res* 68: 650-656

Hoek KS, Schlegel NC, Eichhoff OM, Widmer DS, Praetorius C, Einarsson SO, Valgeirsdottir S, Bergsteinsdottir K, Schepsky A, Dummer R *et al* (2008b) Novel MITF targets identified using a two-step DNA microarray strategy. *Pigment Cell Melanoma Res* 21: 665-676

Hooper R, Zhang X, Webster M, Go C, Kedra J, Marchbank K, Gill DL, Weeraratna AT, Trebak M, Soboloff J (2015) Novel Protein Kinase C-Mediated Control of Orai1 Function in Invasive Melanoma. *Mol Cell Biol* 35: 2790-2798

Huang Z, Sun Z, Zhang X, Niu K, Wang Y, Zheng J, Li H, Liu Y (2019) Loss of stretch-activated channels, PIEZOs, accelerates non-small cell lung cancer progression and cell migration. *Biosci Rep* 39

Hung WC, Yang JR, Yankaskas CL, Wong BS, Wu PH, Pardo-Pastor C, Serra SA, Chiang MJ, Gu Z, Wirtz D *et al* (2016) Confinement Sensing and Signal Optimization via Piezo1/PKA and Myosin II Pathways. *Cell Rep* 15: 1430-1441

Hutson MR, Keyte AL, Hernandez-Morales M, Gibbs E, Kupchinsky ZA, Argyridis I, Erwin KN, Pegram K, Kneifel M, Rosenberg PB *et al* (2017) Temperature-activated ion channels in neural crest cells confer maternal fever-associated birth defects. *Sci Signal* 10

Iwata Y, Ito S, Wakabayashi S, Kitakaze M (2020) TRPV2 channel as a possible drug target for the treatment of heart failure. *Lab Invest* 100: 207-217

Jahnel R, Bender O, Munter LM, Dreger M, Gillen C, Hucho F (2003) Dual expression of mouse and rat VRL-1 in the dorsal root ganglion derived cell line F-11 and biochemical analysis of VRL-1 after heterologous expression. *Eur J Biochem* 270: 4264-4271

Juhasz I, Albelda SM, Elder DE, Murphy GF, Adachi K, Herlyn D, Valyi-Nagy IT, Herlyn M (1993) Growth and invasion of human melanomas in human skin grafted to immunodeficient mice. *Am J Pathol* 143: 528-537

Kabbarah O, Nogueira C, Feng B, Nazarian RM, Bosenberg M, Wu M, Scott KL, Kwong LN, Xiao Y, Cordon-Cardo C *et al* (2010) Integrative genome comparison of primary and metastatic melanomas. *PLoS One* 5: e10770

Katanosaka K, Takatsu S, Mizumura K, Naruse K, Katanosaka Y (2018) TRPV2 is required for mechanical nociception and the stretch-evoked response of primary sensory neurons. *Sci Rep* 8: 16782

Kemper K, de Goeje PL, Peeper DS, van Amerongen R (2014) Phenotype switching: tumor cell plasticity as a resistance mechanism and target for therapy. *Cancer Res* 74: 5937-5941

Khoja L, Shenjere P, Hodgson C, Hodgetts J, Clack G, Hughes A, Lorigan P, Dive C (2014) Prevalence and heterogeneity of circulating tumour cells in metastatic cutaneous melanoma. *Melanoma Res* 24: 40-46

Klapholz B, Brown NH (2017) Talin - the master of integrin adhesions. *J Cell Sci* 130: 2435-2446

Kojima I, Nagasawa M (2014) Trpv2. *Handb Exp Pharmacol* 222: 247-272

Lamsoul I, Dupre L, Lutz PG (2020) Molecular Tuning of Filamin A Activities in the Context of Adhesion and Migration. *Front Cell Dev Biol* 8: 591323

Laukaitis CM, Webb DJ, Donais K, Horwitz AF (2001) Differential dynamics of alpha 5 integrin, paxillin, and alpha-actinin during formation and disassembly of adhesions in migrating cells. *J Cell Biol* 153: 1427-1440

Liu S, Xu X, Fang Z, Ning Y, Deng B, Pan X, He Y, Yang Z, Huang K, Li J (2021) Piezo1 impairs hepatocellular tumor growth via deregulation of the MAPK-mediated YAP signaling pathway. *Cell Calcium* 95: 102367

Liu YJ, Le Berre M, Lautenschlaeger F, Maiuri P, Callan-Jones A, Heuze M, Takaki T, Voituriez R, Piel M (2015) Confinement and low adhesion induce fast amoeboid migration of slow mesenchymal cells. *Cell* 160: 659-672

Lopez-Colome AM, Lee-Rivera I, Benavides-Hidalgo R, Lopez E (2017) Paxillin: a crossroad in pathological cell migration. *J Hematol Oncol* 10: 50

Mathieu V, Pirker C, Martin de Lassalle E, Vernier M, Mijatovic T, DeNeve N, Gaussin JF, Dehoux M, Lefranc F, Berger W *et al* (2009) The sodium pump alpha1 sub-unit: a disease progression-related target for metastatic melanoma treatment. *J Cell Mol Med* 13: 3960-3972

Mazar J, DeYoung K, Khaitan D, Meister E, Almodovar A, Goydos J, Ray A, Perera RJ (2010) The regulation of miRNA-211 expression and its role in melanoma cell invasiveness. *PLoS One* 5: e13779

McNeill MS, Paulsen J, Bonde G, Burnight E, Hsu MY, Cornell RA (2007) Cell death of melanophores in zebrafish trpm7 mutant embryos depends on melanin synthesis. *J Invest Dermatol* 127: 2020-2030

Middelbeek J, Kuipers AJ, Henneman L, Visser D, Eidhof I, van Horssen R, Wieringa B, Canisius SV, Zwart W, Wessels LF *et al* (2012) TRPM7 is required for breast tumor cell metastasis. *Cancer Res* 72: 4250-4261

Monet M, Gkika D, Lehen'kyi V, Pourtier A, Vanden Abeele F, Bidaux G, Juvin V, Rassendren F, Humez S, Prevarsakaya N (2009) Lysophospholipids stimulate prostate cancer cell migration via TRPV2 channel activation. *Biochim Biophys Acta* 1793: 528-539

Monet M, Lehen'kyi V, Gackiere F, Firlej V, Vandenberghe M, Roudbaraki M, Gkika D, Pourtier A, Bidaux G, Slomianny C *et al* (2010) Role of cationic channel TRPV2 in promoting prostate cancer migration and progression to androgen resistance. *Cancer Res* 70: 1225-1235

Nagasawa M, Kojima I (2015) Translocation of TRPV2 channel induced by focal administration of mechanical stress. *Physiol Rep* 3

Nagasawa M, Nakagawa Y, Tanaka S, Kojima I (2007) Chemotactic peptide fMetLeuPhe induces translocation of the TRPV2 channel in macrophages. *J Cell Physiol* 210: 692-702

Newell-Litwa KA, Horwitz AR (2011) Cell migration: PKA and RhoA set the pace. *Curr Biol* 21: R596-598

Nuzzi PA, Senetar MA, Huttenlocher A (2007) Asymmetric localization of calpain 2 during neutrophil chemotaxis. *Mol Biol Cell* 18: 795-805

Oancea E, Vriens J, Brauchi S, Jun J, Splawski I, Clapham DE (2009) TRPM1 forms ion channels associated with melanin content in melanocytes. *Sci Signal* 2: ra21

Oulidi A, Bokhobza A, Gkika D, Vanden Abeele F, Lehen'kyi V, Ouafik L, Mauroy B, Prevarskaya N (2013) TRPV2 mediates adrenomedullin stimulation of prostate and urothelial cancer cell adhesion, migration and invasion. *PLoS One* 8: e64885

Pardo-Pastor C, Rubio-Moscardo F, Vogel-Gonzalez M, Serra SA, Afthinos A, Mrkonjic S, Destaing O, Abenza JF, Fernandez-Fernandez JM, Trepat X *et al* (2018) Piezo2 channel regulates RhoA and actin cytoskeleton to promote cell mechanobiological responses. *Proc Natl Acad Sci U S A* 115: 1925-1930

Petropoulos C, Oddou C, Emadali A, Hiriart-Bryant E, Boyault C, Faurobert E, Vande Pol S, Kim-Kaneyama JR, Kraut A, Coute Y *et al* (2016) Roles of paxillin family members in adhesion and ECM degradation coupling at invadosomes. *J Cell Biol* 213: 585-599

Poole K, Herget R, Lapatsina L, Ngo HD, Lewin GR (2014) Tuning Piezo ion channels to detect molecular-scale movements relevant for fine touch. *Nat Commun* 5: 3520

Qin N, Neeper MP, Liu Y, Hutchinson TL, Lubin ML, Flores CM (2008) TRPV2 is activated by cannabidiol and mediates CGRP release in cultured rat dorsal root ganglion neurons. *J Neurosci* 28: 6231-6238

Rambow F, Job B, Petit V, Gesbert F, Delmas V, Seberg H, Meurice G, Van Otterloo E, Dessen P, Robert C *et al* (2015) New Functional Signatures for Understanding Melanoma Biology from Tumor Cell Lineage-Specific Analysis. *Cell Rep* 13: 840-853

Rambow F, Marine JC, Goding CR (2019) Melanoma plasticity and phenotypic diversity: therapeutic barriers and opportunities. *Genes Dev* 33: 1295-1318

Restivo G, Diener J, Cheng PF, Kiowski G, Bonalli M, Biedermann T, Reichmann E, Levesque MP, Dummer R, Sommer L (2017) low neurotrophin receptor CD271 regulates phenotype switching in melanoma. *Nat Commun* 8: 1988

Rodriguez-Hernandez I, Maiques O, Kohlhammer L, Cantelli G, Perdrix-Rosell A, Monger J, Fanshawe B, Bridgeman VL, Karagiannis SN, Penin RM *et al* (2020) WNT11-FZD7-DAAM1 signalling supports tumour initiating abilities and melanoma amoeboid invasion. *Nat Commun* 11: 5315

Rybarczyk P, Vanlaeys A, Brassart B, Dhennin-Duthille I, Chatelain D, Sevestre H, Ouadid-Ahidouch H, Gautier M (2017) The Transient Receptor Potential Melastatin 7 Channel Regulates Pancreatic Cancer Cell Invasion through the Hsp90alpha/uPA/MMP2 pathway. *Neoplasia* 19: 288-300

Santoni G, Farfariello V, Liberati S, Morelli MB, Nabissi M, Santoni M, Amantini C (2013) The role of transient receptor potential vanilloid type-2 ion channels in innate and adaptive immune responses. *Front Immunol* 4: 34

Saxena M, Changede R, Hone J, Wolfenson H, Sheetz MP (2017) Force-Induced Calpain Cleavage of Talin Is Critical for Growth, Adhesion Development, and Rigidity Sensing. *Nano Lett* 17: 7242-7251

Seberg HE, Van Otterloo E, Loftus SK, Liu H, Bonde G, Sompallae R, Gildea DE, Santana JF, Manak JR, Pavan WJ *et al* (2017) TFAP2 paralogs regulate melanocyte differentiation in parallel with MITF. *PLoS Genet* 13: e1006636

Shibasaki K, Murayama N, Ono K, Ishizaki Y, Tominaga M (2010) TRPV2 enhances axon outgrowth through its activation by membrane stretch in developing sensory and motor neurons. *J Neurosci* 30: 4601-4612

Simmons JL, Pierce CJ, Al-Ejeh F, Boyle GM (2017) MITF and BRN2 contribute to metastatic growth after dissemination of melanoma. *Sci Rep* 7: 10909

Sommer L (2011) Generation of melanocytes from neural crest cells. *Pigment Cell Melanoma Res* 24: 411-421

Stokes AJ, Shimoda LM, Koblan-Huberson M, Adra CN, Turner H (2004) A TRPV2-PKA signaling module for transduction of physical stimuli in mast cells. *J Exp Med* 200: 137-147

Su LT, Agapito MA, Li M, Simonson WT, Huttenlocher A, Habas R, Yue L, Runnels LW (2006) TRPM7 regulates cell adhesion by controlling the calcium-dependent protease calpain. *J Biol Chem* 281: 11260-11270

Su LT, Chen HC, Gonzalez-Pagan O, Overton JD, Xie J, Yue L, Runnels LW (2010) TRPM7 activates m-calpain by stress-dependent stimulation of p38 MAPK and c-Jun N-terminal kinase. *J Mol Biol* 396: 858-869

Su LT, Liu W, Chen HC, Gonzalez-Pagan O, Habas R, Runnels LW (2011) TRPM7 regulates polarized cell movements. *Biochem J* 434: 513-521

Sugio S, Nagasawa M, Kojima I, Ishizaki Y, Shibasaki K (2017) Transient receptor potential vanilloid 2 activation by focal mechanical stimulation requires interaction with the actin cytoskeleton and enhances growth cone motility. *FASEB J* 31: 1368-1381

te Boekhorst V, Jiang L, Mählen M, Meerlo M, Dunkel G, Durst FC, Yang Y, Levine H, Burgering BMT, Friedl P (2020) Calpain-2 regulates hypoxia/HIF-induced amoeboid reprogramming and metastasis. *bioRxiv*: 2020.2001.2006.892497

Thomson JA, Murphy K, Baker E, Sutherland GR, Parsons PG, Sturm RA, Thomson F (1995) The brn-2 gene regulates the melanocytic phenotype and tumorigenic potential of human melanoma cells. *Oncogene* 11: 691-700
Tichet M, Prod'Homme V, Fenouille N, Ambrosetti D, Mallavialle A, Cerezo M, Ohanna M, Audebert S, Rocchi S, Giacchero D *et al* (2015) Tumour-derived SPARC drives vascular permeability and extravasation through endothelial VCAM1 signalling to promote metastasis. *Nat Commun* 6: 6993
Tirosh I, Izar B, Prakadan SM, Wadsworth MH, 2nd, Treacy D, Trombetta JJ, Rotem A, Rodman C, Lian C, Murphy G *et al* (2016) Dissecting the multicellular ecosystem of metastatic melanoma by single-cell RNA-seq. *Science* 352: 189-196
Tuncer E, Calcada RR, Zingg D, Varum S, Cheng P, Freiberger SN, Deng CX, Kleiter I, Levesque MP, Dummer R *et al* (2019) SMAD signaling promotes melanoma metastasis independently of phenotype switching. *J Clin Invest* 129: 2702-2716
Verfaillie A, Imrichova H, Atak ZK, Dewaele M, Rambow F, Hulselmans G, Christiaens V, Svetlichnyy D, Luciani F, Van den Mooter L *et al* (2015) Decoding the regulatory landscape of melanoma reveals TEADS as regulators of the invasive cell state. *Nat Commun* 6: 6683
Vriens J, Appendino G, Nilius B (2009) Pharmacology of vanilloid transient receptor potential cation channels. *Mol Pharmacol* 75: 1262-1279
Wellbrock C, Rana S, Paterson H, Pickersgill H, Brummelkamp T, Marais R (2008) Oncogenic BRAF regulates melanoma proliferation through the lineage specific factor MITF. *PLoS One* 3: e2734
White JR, Thompson DT, Koch KE, Kiriazov BS, Beck AC, van der Heide DM, Grimm BG, Kulak MV, Weigel RJ (2021) AP-2alpha-Mediated Activation of E2F and EZH2 Drives Melanoma Metastasis. *Cancer Res* 81: 4455-4470
Wouters J, Kalender-Atak Z, Minnoye L, Spanier KI, De Waegeneer M, Bravo Gonzalez-Blas C, Mauduit D, Davie K, Hulselmans G, Najem A *et al* (2020) Robust gene expression programs underlie recurrent cell states and phenotype switching in melanoma. *Nat Cell Biol* 22: 986-998
Yang S, Zhang JJ, Huang XY (2009) Orai1 and STIM1 are critical for breast tumor cell migration and metastasis. *Cancer Cell* 15: 124-134
Yao M, Tijore A, Cox CD, Hariharan A, Van Nhieu GT, Martinac B, Sheetz M (2020) Force-dependent Piezo1 recruitment to focal adhesions regulates adhesion maturation and turnover specifically in non-transformed cells. *bioRxiv*: 2020.2003.2009.972307
Yee NS, Kazi AA, Li Q, Yang Z, Berg A, Yee RK (2015) Aberrant over-expression of TRPM7 ion channels in pancreatic cancer: required for cancer cell invasion and implicated in tumor growth and metastasis. *Biol Open* 4: 507-514
Yu Y, Wu X, Liu S, Zhao H, Li B, Zhao H, Feng X (2021a) Piezo1 regulates migration and invasion of breast cancer cells via modulating cell mechanobiological properties. *Acta Biochim Biophys Sin (Shanghai)* 53: 10-18
Yu ZH, Ji YC, Li K, Liang T, Liu B, Chen HL, Ni L, Luo ZP, Yang HL (2021b) Stiffness of the extracellular matrix affects apoptosis of nucleus pulposus cells by regulating the cytoskeleton and activating the TRPV2 channel protein. *Cell Signal* 84: 110005
Zaidel-Bar R, Ballestrem C, Kam Z, Geiger B (2003) Early molecular events in the assembly of matrix adhesions at the leading edge of migrating cells. *J Cell Sci* 116: 4605-4613
Zhang J, Zhou Y, Huang T, Wu F, Liu L, Kwan JSH, Cheng ASL, Yu J, To KF, Kang W (2018) PIEZO1 functions as a potential oncogene by promoting cell proliferation and migration in gastric carcinogenesis. *Mol Carcinog* 57: 1144-1155
zhang s, cao s, mengyuan g, wunai z, weifan z, zeen z, shuai w, yue s, lin w, yangyang y *et al* (2022) The Piezo1 Mechanosensitive Ion Channel Promotes Melanoma Malignant Progression by Activating PI3K-AKT Signaling. *PREPRINT (Version 1) available at Research Square*

1st Editorial Decision 1st Apr 2022

Dear Dr. Penna

Thank you once more for the submission of your research manuscript to our journal. I apologize for my delayed response, but I have been sick with COVID and quite some work has accumulated in the meantime. As discussed, we would like to invite you to revise your study for potential publication in EMBO Reports. Please finalize the revisions along the lines outlined in your point-by-point response. Further data on the causal link between TRPV2, calpain/talin and cell migration should be provided, as outlined. In this context, it might also be interesting to test a calpain-resistant form or talin in the context of TRPV2 overexpression.

Please address all referee concerns in a complete point-by-point response. Acceptance of the manuscript will depend on a positive outcome of a second round of review. It is EMBO reports policy to allow a single round of revision only and acceptance or rejection of the manuscript will therefore depend on the completeness of your responses included in the next, final version of the manuscript.

We realize that it is difficult to revise to a specific deadline. In the interest of protecting the conceptual advance provided by the work, we recommend a revision within 3 months (July 1, 2022). Please discuss the revision progress ahead of this time with the editor if you require more time to complete the revisions.

Below, I detail the format requirements for EMBO Reports. Please note that all Material and Methods must be part of the main manuscript.

*****IMPORTANT NOTE:
We perform an initial quality control of all revised manuscripts before re-review. Your manuscript will FAIL this control and the handling will be DELAYED, IF the following APPLIES:

1) A data availability section providing access to data deposited in public databases is missing. If you have not deposited any data, please add a sentence to the data availability section that explains that.

2) Your manuscript contains statistics and error bars based on n=2. Please use scatter blots in these cases. No statistics should be calculated if n=2.

When submitting your revised manuscript, please carefully review the instructions that follow below. Failure to include requested items will delay the evaluation of your revision.*****

When submitting your revised manuscript, we will require:

1) a .docx formatted version of the manuscript text (including legends for main figures, EV figures and tables). Please make sure that the changes are highlighted to be clearly visible.

2) individual production quality figure files as .eps, .tif, .jpg (one file per figure).
Please download our Figure Preparation Guidelines (figure preparation pdf) from our Author Guidelines pages https://www.embopress.org/page/journal/14693178/authorguide for more info on how to prepare your figures.

3) a .docx formatted letter INCLUDING the reviewers' reports and your detailed point-by-point responses to their comments. As part of the EMBO Press transparent editorial process, the point-by-point response is part of the Review Process File (RPF), which will be published alongside your paper.

4) a complete author checklist, which you can download from our author guidelines (). Please insert information in the checklist that is also reflected in the manuscript. The completed author checklist will also be part of the RPF.

5) Please note that all corresponding authors are required to supply an ORCID ID for their name upon submission of a revised manuscript (). Please find instructions on how to link your ORCID ID to your account in our manuscript tracking system in our Author guidelines
()

6) We replaced Supplementary Information with Expanded View (EV) Figures and Tables that are collapsible/expandable online. A maximum of 5 EV Figures can be typeset. EV Figures should be cited as 'Figure EV1, Figure EV2" etc... in the text and their respective legends should be included in the main text after the legends of regular figures.

- For the figures that you do NOT wish to display as Expanded View figures, they should be bundled together with their legends

in a single PDF file called *Appendix*, which should start with a short Table of Content. Appendix figures should be referred to in the main text as: "Appendix Figure S1, Appendix Figure S2" etc. See detailed instructions regarding expanded view here:

- Additional Tables/Datasets should be labeled and referred to as Table EV1, Dataset EV1, etc. Legends have to be provided in a separate tab in case of .xls files. Alternatively, the legend can be supplied as a separate text file (README) and zipped together with the Table/Dataset file.

7) Please note that a Data Availability section at the end of Materials and Methods is now mandatory. In case you have no data that requires deposition in a public database, please state so instead of refereeing to the database.
See also < https://www.embopress.org/page/journal/14693178/authorguide#dataavailability>). Please note that the Data Availability Section is restricted to new primary data that are part of this study.

8) Figure legends and data quantification:
The following points must be specified in each figure legend:
- the name of the statistical test used to generate error bars and P values,
- the number (n) of independent experiments (please specify technical or biological replicates) underlying each data point,
- the nature of the bars and error bars (s.d., s.e.m.)
- If the data are obtained from n {less than or equal to} 2, use scatter blots showing the individual data points.
Discussion of statistical methodology can be reported in the materials and methods section, but figure legends should contain a basic description of n, P and the test applied.
See also the guidelines for figure legend preparation:
https://www.embopress.org/page/journal/14693178/authorguide#figureformat
- Please also include scale bars in all microscopy images.

9) We would also encourage you to include the source data for figure panels that show essential data. Numerical data should be provided as individual .xls or .csv files (including a tab describing the data). For blots or microscopy, uncropped images should be submitted (using a zip archive if multiple images need to be supplied for one panel). Additional information on source data and instruction on how to label the files are available .

10) Our journal encourages inclusion of *data citations in the reference list* to directly cite datasets that were re-used and obtained from public databases. Data citations in the article text are distinct from normal bibliographical citations and should directly link to the database records from which the data can be accessed. In the main text, data citations are formatted as follows: "Data ref: Smith et al, 2001" or "Data ref: NCBI Sequence Read Archive PRJNA342805, 2017". In the Reference list, data citations must be labeled with "[DATASET]". A data reference must provide the database name, accession number/identifiers and a resolvable link to the landing page from which the data can be accessed at the end of the reference. Further instructions are available at .

11) As part of the EMBO publication's Transparent Editorial Process, EMBO reports publishes online a Review Process File to accompany accepted manuscripts. This File will be published in conjunction with your paper and will include the referee reports, your point-by-point response and all pertinent correspondence relating to the manuscript.

You are able to opt out of this by letting the editorial office know (emboreports@embo.org). If you do opt out, the Review Process File link will point to the following statement: "No Review Process File is available with this article, as the authors have chosen not to make the review process public in this case."

We would also welcome the submission of cover suggestions, or motifs to be used by our Graphics Illustrator in designing a cover.

I look forward to seeing a revised form of your manuscript when it is ready and please let me know if you have questions or comments regarding the revision.

Yours sincerely,

Martina Rembold, PhD
Senior Editor
EMBO reports

Manuscript number: RC-2021-01119 / EMBOR-2022-55069V1
Corresponding author(s): Penna, Aubin

Reviewers' reports and point-by-point responses

We are sincerely grateful for the tremendous impact this constructive review had on our study. Every comment was carefully taken into account, and definitely strengthened our conclusions and improved the manuscript. We appreciate the clear interest that all three Reviewers have shown regarding the significance of the study rationale and its clinical implications in the field of metastatic melanoma. We are also thankful for their appreciation of the novelty of the findings, the pertinence of the data, as well as the clarity of the presentation.

As clearly articulated in this report, our main goal was to provide insights into the urgent need for a new biomarker and potential therapeutic target for metastatic melanoma. Our *in vitro* and *in vivo* data identified the mechanosensitive TRPV2 calcium (Ca^{2+}) channel as a new regulator of melanoma cells metastatic potential. Additionally, we could correlate the expression of TRPV2 to the aggressiveness of the disease and patients' survival, opening new perspectives for future treatment. Beside sustained efforts to validate the new role of this Ca^{2+} channel in the metastatic dissemination, we investigated the mechanistic basis enabling such function. By modulating TRPV2 expression, and therefore directly affecting its "constitutive" activity, we found that the activity of the Ca^{2+}-activated calpain, together with the cleavage of its substrate, the adhesive protein Talin, were dependent upon TRPV2. Such new findings, establishing a mechanism connecting a mechanosensitive channel to the intracellular machinery regulating the adhesion/migration processes in advanced melanoma, are highly significant and promising, albeit raising numerous questions. Although our initial data revealed converging evidence associating TRPV2 expression/activity, calpain activity and the resulting migration behaviors, the regulation of calpain activity upon the changes in TRPV2 expression was not formally related to melanoma cells motility. As suggested by the referees, we have now assessed the migration potential of melanoma cells upon calpain activity modulation in the context of TRPV2 overexpression or repression, and established a clear causal link between the TRPV2-dependent activity of calpain and melanoma cells migration. Hence, according to the present review together with our additional data, we believe this manuscript convincingly achieves our goal, namely to identify a new biomarker and potential therapeutic target for advanced melanoma, by placing TRPV2 mechanosensitive channel as a key regulator of metastatic melanoma cells migration.

PS: To facilitate the reading of our point-by-point responses, the points raised by the referees are in black italic and our reply is in blue.

Our revised manuscript has been fully reformatted according to EMBO Report guidelines and publication policies (e.g. M&M section included in the main manuscript, 3 levels for figures...)

To help reviewers track changes in the revised manuscript, amendments made in response to the referees are in green.

Reviewer #1:

***1)** how specific the calcium influx via TRPV2 contributes to the downstream signaling, such as calpain activation (as we know many routes of calcium influx can activate calpain);*

Other routes of Ca^{2+} influx, such as Store Operated Calcium Entry (SOCE), TRPM7 or PIEZOs, have indeed been described as potential activators of calpain, regulating cell adhesion (Chen *et al*, 2011; D'Souza *et al*, 2020; Liu *et al*, 2021; Pardo-Pastor *et al*, 2018; Su *et al*, 2006; Yang *et al*, 2009; Yao *et al*, 2020). It is now well-accepted that in the rear of migrating cells, Ca^{2+} activates calpain which catalyzes the cleavage of FA proteins, such as integrins, talin, vinculin and FAK (Glading *et al*, 2002), promoting the disassembly of the mature structures. As the SOCE signal is significantly impeded in invasive (compared to non-invasive) melanoma cells (Hooper *et al*, 2015), and since this Ca^{2+} entry is not defined as mechanosensitive and its local recruitment at adhesion sites is not clear, SOCE does not appear as a prime candidate in regulating nascent adhesion in advanced melanoma cells. Regarding TRPM7, its ion permeability is distinctive and allows the passage of Ca^{2+}, Mg^{2+} and other cations. This chanzyme has been described as a regulator of calpain-2 (m-calpain) activation in vinculin-containing FAs, but evidence suggest that this TRPM7 function is achieved neither by raising the global cytosolic Ca^{2+} concentration, nor through its kinase activity, but rather *via* increasing the intracellular Mg^{2+} concentration (Su *et al.*, 2006; Su *et al*, 2010; Su *et al*, 2011), leaving the regulation of the Ca^{2+} signal controlling calpain activity as an opened question. Finally, concerning PIEZO1, despite a clear role in regulating FA dynamics (assembly of mature adhesions and not disassembly) its precise function in melanoma migration remains undefined (Chen *et al*, 2018), specially since this channel has been reported as stabilizing adhesion in normal but not in transformed cells (Yao *et al.*, 2020). Importantly, the same study showed that PIEZO1 did not localize to paxillin-containing adhesions in A2058 melanoma cells. However, we do not exclude a cooperation between PIEZO1 and TRPV2 in migrating metastatic cells (which is further discussed below in answer to Reviewer#1's minor issue), that might be involved in the regulation of TRPV2 addressing to the plasma membrane. In any event, we can not rule out the participation of other Ca^{2+}-regulating actors into the modulation of calpain activity.

Importantly, to understand the role of each of these channels in cellular migration, it is necessary to consider the cellular model and context in which these studies are conceived. For instance, most of the above reports relate to mature focal adhesions, leaving the mechanistic basis for nascent adhesion dynamics extremely unsettled. In fibroblast, one report evokes the role of Ca^{2+} by showing that adhesion maturation and proper mechanosensing are regulated by the force-induced cleavage of Talin in early adhesions, which depends upon calpain and the TRPM4 channel (Saxena *et al*, 2017). Although TRPM4 is a Ca^{2+}- and voltage-activated channel, it is permeable only to monovalent cations and not to Ca^{2+}, leaving once again the question of the upstream Ca^{2+} signaling regulator unanswered. In parallel, it is noteworthy that numerous reports involving Ca^{2+} channels activity in cellular migration were based on pharmacological modulations, which can be misleading due to nonspecific/off-target effects. In addition, the activation of a Ca^{2+} channel by an agonist often triggers a massive and diffuse Ca^{2+} signal, likely different from local signals resulting from fine-tuned constitutive or endogenous channel activation, potentially leading to artifacts. Hence, we believe that pharmacological studies, although contributing to our understanding, should be balanced by several approaches to enable solid conclusions.

In our study, using TRPV2 gain or loss-of-function strategies, we showed that the modulation of TRPV2 expression and subsequently of its associated constitutive activity **(Figures 2C-E)** were inducing analogous changes in both calpain activity and the cleavage of its known substrate, Talin **(Figure 5 B-D)**. More precisely, the levels of modulations induced on TRPV2 activity, whether it was up- or down-regulated, were strictly comparable and proportional to the rate of alterations observed on calpain activity and Talin proteolysis. As a matter of fact, TRPV2 silencing prevented at least 50% and up to 80% of Talin cleavage in metastatic cells. The pharmacological activation of TRPV2 by its potent agonist (cannabidiol), in presence or absence of Ca^{2+}, further established the Ca^{2+} dependency of calpain-mediated cleavage of Talin **(Appendix Figure S6C and D)**. Altogether these data strongly support the conclusion that, at least part of the Ca^{2+}-activated proteolytic activity of calpain depends upon TRPV2 channel-mediated Ca^{2+} signal. As TRPV2 preferentially localized within nascent adhesion structures, we speculate that in advanced melanoma cells,

TRPV2 is regulating calpain-mediated cleavage of adhesion proteins in a spatiotemporally-regulated manner. However, we do not exclude the participation of other Ca^{2+} regulators in the cellular adhesion process. This point has been clarified in the discussion section of the manuscript **(page 16)**.

2) *except knock-down of TRPV2, is there any pharmacological inhibitors or pore-region mutation of V2 that can block the calcium influx via TRPV2? If such inhibitor or mutant are able to display the similar effects as knock-down of TRPV2 in cell migration assay, that would definitely add more definitive evidence for the role of TRPV2 in melanoma metastases.*

As discussed above in response to the first comment, we believe that pharmacological studies strengthen our understanding, but should be balanced with other approaches. Regarding TRPV2, the pharmacological tools available are highly controversial mainly because of specificity issues. Several compounds have been shown to affect TRPV2 function, however most are contentious compounds still under scrutiny (Iwata *et al*, 2020; Vriens *et al*, 2009). Among them, SKF96365 and Ruthenium Red have been identified as non-specific TRPV2 channel blockers. As an inhibitor, Tranilast remains the most used, although it has not been fully validated as a specific blocker of TRPV2. To consolidate the role of TRPV2-mediated Ca^{2+} influx during melanoma metastatic progression, we tested the effect of Tranilast on invasive melanoma cells, and could measure a significant reduction of their migration behavior assessed with wound healing assays. This data has been included in the present manuscript as a supplemental figure **(Appendix Fig S3A)**. Meanwhile, the inhibitory mechanism and the specificity of Tranilast towards human TRPV2 still need further clarifications.

*****Minor issue:*****

- For Fig. 6 B/D, is there any significant difference between ctrl and shTRPV2 group? It seems the error bar is huge, I doubt this will give you a solid answer for the claim "TRPV2 level determines the in vivo metastases potentials of melanoma cells";

We sincerely apologize for forgetting to include the statistical analysis of the *in vivo* and *ex vivo* data **Figure 6**, error which we have rectified.

When comparing the number of metastatic foci *ex vivo*, unpaired t-tests were applied, and the resulting *P* values were 0.0397 (*) in the 501mel-GFP *versus* -GFP-TRPV2 (Figure 6B), and 0.0691 in the 451Lu-shCtrl *versus* -shTRPV2 (Figure 6D). Hence, TRPV2 overexpression in the 501mel model showed a significant increase compared to the control in terms of the number of metastasis, while despite showing a clear trend, invalidation of TRPV2 in the 451Lu cells did not achieve statistical significance *ex vivo* (*P*=0.0691). As for ethical reasons, each group was limited to 6 to 7 mice, adding a couple of animals may have increased the statistical power. Importantly, in the same group of 451-Lu injected mice, *in vivo* imaging displayed a significant loss of photon flux (***P*=0.0064) upon TRPV2 silencing (Figure 6C). Additionally, to strengthen our conclusions, we took advantage of the xenografted zebrafish model, allowing a direct comparison of two cell lines with distinct invasive potentials in the same organism. In this *in vivo* model, the disseminating potential of human melanoma cells was substantially dependent on TRPV2. Hence, based on the results obtained in 3 different models, we concluded that TRPV2 was critical to promote the *in vivo* metastasis potential of human melanoma cells, and have adjusted accordingly the manuscript **(page 11: *TRPV2 expression is critical to the in vivo metastatic potential of melanoma tumor cells*)**, and **Figure 6** title (**page 39**) changed for ***TRPV2 expression level dictates the in vivo metastatic potential of human melanoma tumor cells xenografted in mice.***

- It seems piezo1 levels are equivalent high to TRPV2 from the data screening (Fig. 1A), at least the author should discuss about the possible link between these two channels, as they both mechanical sensitive and calcium permeable.

PIEZO1 is a stretch-activated cation channel, whose activation can be exclusively achieved *via* mechanical forces applied to the cell plasma membrane (PM) (Coste *et al*, 2010), leading to a short activation

phase that allows Ca^{2+} entry, which is followed by an inactivation phase that makes it insensitive to further stimulation (Poole *et al*, 2014). The role of PIEZO1 in cell migration is controversial, as some reports show that inhibition of PIEZO1 increases cell migration, such as in breast cancer, non-small cell lung carcinoma and neural crest cells (Canales Coutino & Mayor, 2021; Huang *et al*, 2019; Yu *et al*, 2021a), whereas others show inhibition in cell migration, such as in gastric cancer and glioma cells (Chen *et al.*, 2018; Zhang *et al*, 2018). Canales Coutino & Mayor suggested that PIEZO1 has a conserved role in regulating FA dynamics (Canales Coutino & Mayor, 2021), but whether this is translated into more or less migration depends on the cellular context.

In melanoma, we observed that Piezo1 is highly expressed in the SKCM tumors dataset **(Figure 1A)**, while Piezo2 expression remains low. Consistently, Piezo1 has been shown to be upregulated in primary and metastatic melanoma compared to normal skin in TCGA, and to correlate with a shorter overall survival of melanoma patients (zhang *et al*, 2022). In this study, PIEZO1 promoted proliferation, invasion and metastasis. However, another study showed that PIEZO1 was important for adhesion maturation in normal cells but did not affect adhesion formation in transformed cells. In normal cells, PIEZO1 was enriched within mature and retracting adhesions, where it was bound to a complex with specific integrins but not adhesion proteins, and this localization was dependent on continued contraction forces. Meanwhile, in the A2058 melanoma cells, PIEZO1 did not localize to paxillin adhesions, suggesting that PIEZO1 functions in mechanosensing processes are distinctively regulated in normal *versus* cancer cells (Yao *et al.*, 2020). In neural crest cells, PIEZO1 was further associated with FAs stabilization (Canales Coutino & Mayor, 2021).

Metastatic cells optimize their migratory potential by adapting their migration modes as they encounter different physical microenvironments. Efficient migration in confined spaces has been suggested to involve two independent confinement-sensing pathways: one mediated by PIEZO1/PDE1/PKA and the other by myosinII/Rac1, integrated through a complex feedback circuit (Hung *et al*, 2016). Upon physical confinement, the A375-SM invasive melanoma cancer cells optimize their motility notably *via* activating PIEZO1, which suppresses PKA and actomyosin contractility. In these melanoma cells, the depletion of PIEZO1 reduced only the confined migration. The cAMP-dependent protein kinase A (PKA), is known to play an important role in the migration of carcinoma cells and in the regulation of RhoA and Rac1 functions in several cooperative pathways (Newell-Litwa & Horwitz, 2011). Meanwhile PKA has also been shown to regulate TRPV2 trafficking to the PM (Stokes *et al*, 2004), and consequently its activation, suggesting that in a confined environment PIEZO1 could prevent TRPV2 translocation at the PM *via* the inhibition of PKA. Conversely, in migrating A375 melanoma cells, activation of Piezo1 increased the activation of the PI3K/AKT pathway (zhang *et al.*, 2022), which regulates TRPV2 translocation to the PM (Nagasawa & Kojima, 2015). Hence, PIEZO1 could regulate TRPV2 activity, by either addressing it to the PM through the activation of the PI3K/AKT pathway, or impeding its trafficking to the PM *via* PKA inhibition. We speculate that both mechanosensitive channels, TRPV2 and PIEZO1, play an important role in the migration and invasion potentials of metastatic melanoma cells. The activation of these channels could be interconnected, either in an alternative or coordinate fashion, depending on the microenvironment encountered by the cells. In this context, they could also engage cooperative functions, as TRPV2 is involved in nascent adhesion disassembly while PIEZO1 seems to preferentially stabilize mature FAs. Further investigations will determine whether, and how, these two mechanosensitive channels coordinate adhesion processes for metastatic melanoma cells to adopt the most appropriate migration mode, in order to achieve the fastest mobility. The potential connection between these two channels has now been mentioned in the discussion of the manuscript (**page 15**).

Reviewer #2:

*****Major points:*****

1) *The gene expression data and TMA staining suggest that high TRPV2 is associated with advanced melanoma. Is the TRPV2 locus altered at the genomic level (by mutation or copy-number alteration)? Could the authors investigate the mechanism of TRPV2 overexpression in melanoma compared to normal melanocytes or benign*

nevi? Knowing more about the regulatory elements in the TRPV2 promoter or enhancer regions and/or about the regulators of TRPV2 stability/cell surface localization would add considerable value to this study.

Referees cross-commenting
I think that the points raised by the other 2 reviewers are well taken. **My major point 1 could be disregarded if the other reviewers feel that it is beyond the scope of the paper.** [...]

We fully agree with Reviewer#2, understanding the regulation of TRPV2 at the genomic level, but also the regulation of its stability and subcellular localization, would be of considerable value.

To tackle this compelling point, we performed data set mining using cBioportal (https://www.cbioportal.org/) and determined whether the TRPV2 locus is altered at the genomic level (**Review additional figure 1A-C**). It appeared that mutations or copy-number (CN) alterations of the TRPV2 gene are rare events in melanoma, suggesting that TRPV2 overexpression in melanoma do not stem from CN-amplification. Hence, the exacerbated expression of TRPV2 in melanoma compared to benign nevi and to other cancer types, rather suggests that some melanocytic lineage-specific transcription factors may be at play (*e.g.* SOX10, MITF, PAX3...), in combination with transcriptional regulators and alterations that drive melanoma progression, especially those supporting a pro-invasive/pro-metastatic behavior such as BRN2 (see below response to reviewer#2's minor points). Favoring this hypothesis, TRPV2 expression in metastatic melanoma is positively correlated with both MITF and BRN2 (**Figures 1H and appendix S1D-E** and **Review additional Figure 2** below). Consistent with a potential regulation of TRPV2 expression by MITF is the fact that TRPV2 expression is induced following MITF overexpression in both the SK-MEL-28 and A375 melanoma cell lines (see Supplemental Data 1 in (Hoek *et al*, 2008b) and Supplementary file 1 in (Dilshat *et al*, 2021)). Yet, the opposite trend was not observed upon MITF invalidation or knock-down, suggesting an indirect regulation of TRPV2 *via* MITF. Mechanistically, it appears that neither MITF nor BRN2 may directly activate TRPV2 expression, as our analyses of the promoter region of TRPV2 using publically available ChIP-seq data did not show any potential binding sites for either transcription factor in melanoma cell lines.

However, we searched for melanoma cell lines with strong TRPV2 expression based on single cell RNAseq data (Wouters *et al*, 2020) and analyzed the corresponding H3k27ac tracks (Verfaillie *et al*, 2015) for predicted transcription factor binding sites (JASPAR database). This approach revealed multiple TFAP2A sites into the putative proximal TRPV2 enhancer region. The TFAP2A gene codes for the AP-2α transcription factor which controls neural crest differentiation and development. Interestingly, TFAP2A is strongly expressed in neural crest cells migrating from the cranial folds during neural tube closure and has been shown to cooperate with MITF to drive melanocyte differentiation (Seberg *et al*, 2017). In this context, Seberg *et al.* performed an integrated analysis of both TFAP2A and MITF ChIP-seq with H3K27ac ChIP-seq data from human melanocytes that predicted an active TFAP2A/MITF shared peak associated with the TRPV2 gene (see table S7 in (Seberg *et al.*, 2017)). TFAP2A was also identified as part of a core melanoma-specific molecular signature (Rambow *et al*, 2015) and, later on, showed to be a transcription factor enriched in melanocytic and "intermediate" states melanoma cells (Wouters *et al.*, 2020) consistent with the higher expression of TRPV2 observed in these two $MITF^{high}$ subpopulations (**Review additional Figure 2A)[Figures for referees not shown.]** .

Interestingly it has been recently shown that TFAP2A/AP-2α could facilitate melanoma metastasis formation (White *et al*, 2021) and that TFAP2A is one of the gene of the so-called "MITF program" overexpressed in the SMAD7^{low} signature that overcome the need for phenotype switching to promote metastatic progression (Tuncer *et al.*, 2019). Altogether this suggests that TRPV2 could be under the transcriptional control of TFAP2A and link TFAP2A to invasiveness. Yet, while gene correlation analyses show a positive correlation between TRPV2 and TFAP2A expression in both melanoma cell lines and tumors (**Review additional Figure 1D**)[Figures for referees not shown.] , the values of the Pearson's correlation coefficient are rather low and suggest that the regulation of TRPV2 is more complex and possibly dependent on additional factors, such as cofactor availability or chromatin accessibility.

Hence, we think that determining the exact transcription factors combination, epigenetic regulation, non-genomic regulation or tumor microenvironmental factors leading to high expression/activity of TRPV2 and promoting migration/invasion independently of the phenotype switching, while of great interest, is beyond the scope of this manuscript.

2) *To explain the effects of TRPV2 manipulation on melanoma cell migration and invasion, the authors propose a mechanism whereby TRPV2-dependent Ca2+ influx activates calpain, resulting in talin cleavage and dissolution of nascent focal adhesion. However, these changes observed upon TRPV2 overexpression or downregulation are not formally implicated in the regulation of melanoma cell motility. To prove a causal link between these correlative observations, the authors should i) use calpain inhibitors or express calpain-uncleavable talin and measure cell migration in the context of TRPV2 overexpression, and ii) express a constitutively active form of calpain or downregulate talin and measure cell migration in the context of TRPV2 downregulation. These experiments would provide a more complete demonstration of the proposed mechanism and would improve the manuscript.*

Reviewer #2: **Referees cross-commenting**
I think that the points raised by the other 2 reviewers are well taken. My major point 1 could be disregarded if the other reviewers feel that it is beyond the scope of the paper. However, I still feel that the paper could be stronger if the authors more formally proved the proposed mechanism of action of TRPV2 (major point 2).
Reviewer #3: **Referees cross-commenting**
I agree with point 2 raised by reviewer 2. It should not be a major burden to perform the suggested experiments (effects of calpain inhibitor or talin knockdown on migration) but will have a major impact on the strength of the paper. The rest of the suggestions made are cosmetic changes and clarifications.

We fully agree with this comment, and firmly believe that proving a causal link between the TRPV2-dependent activation of calpain and melanoma cells migration was needed to strengthen our conclusions. To that end, and as suggested by the reviewers, we undertook the following experiments:

1- In the context of TRPV2 overexpression, we measured the wound healing potential of 501mel melanoma cells upon the selective inhibition of calpains, using either calpeptin, blocking the active site of calpains, or the non-peptide PD150606 inhibitor interacting with the Ca^{2+} binding sites of the protease **(Figure EV2A-B and appendix S6E)**. While both inhibitors did not significantly affect the migration behavior of the non-invasive MOCK 501mel cells, they both hindered the migration speed of TRPV2 overexpressing 501mel cells. Interestingly, the migration capacities of these cells was severely delayed by the blocker of the calpains Ca^{2+} binding site, PD150606, evidencing the necessity of Ca^{2+} binding in this context.

2- Reciprocally, in the context of TRPV2 repression, we assessed the migration potential of WM266.4 cells upon overexpression of a constitutively active form of calpain-2 (calpain-2^{S50E}) **(Figure EV2C-D)**. Regarding the overexpression of a constitutively active form of calpain-2, several points had to be taken into account. Calpains activity has been involved in both early and engaged adhesions, at the leading and the rear edges of the cell, which means that boosting calpains activity could disrupt the adhesive properties of the cells. The proteases activity has also been involved in regulating proliferation and cell death, notably through apoptosis by activating caspases. To circumvent any interference of these effects on cell migration behavior, we measured transwell migration over a very short period of time (5 hours). Indeed, cells overexpressing active calpain-2 displayed adhesion weakness which precluded the use of wound-healing assays. As an alternative approach we use the modified boyden chamber assay (CIM plates) of the xCELLigence impedance-based system. In these assay, monitoring in real-time the migration speed of control WM266.4 cells, TRPV2-silenced or TRPV2-silenced overexpressing active calpain-2 cells, demonstrated that calpain-2 overactivation rescued the migration default observed in TRPV2-silenced cells.

Additionally, given the fact that talin is a substrate of calpain-2, and that we observed the TRPV2-dependent cleavage of talin in metastatic cells as well as a rescue of the migration potential of metastatic melanoma cells in the context of TRPV2 repression, we can presume that TRPV2-mediated Ca^{2+} influx activates ad minima calpain-2, to control cellular migration.
Invalidating Talin would further address the hypothesis that Talin is a major substrate of calpain involved in TRPV2-driven metastatic melanoma cells motility. Although in our study Talin proteolysis was initially used as a second-line readout of calpain activity, given its major role in early adhesion turnover, it is definitely a highly propitious target worthy of interest for a follow up study. However, Talin might not be the only substrate of calpain involved in melanoma cells migration, especially given the fact that calpains have a very large panel of substrates involved in the regulation of adhesions dynamics, including other cell motility modulators, such as Paxillin, Vinculin, or Filamin A (Lamsoul *et al*, 2020). This has been mentioned in the discussion (**Page 17**).

In any event, these additional experiments, showing a causal link between the changes in calpains activity upon the modulation of TRPV2 expression and the migration potential of melanoma cells (**Results page 10 and Figure EV2**), provide a complete demonstration of the proposed mechanistic model presented in the manuscript. They also set the basis for future investigations on the TRPV2-mediated spatiotemporal regulation of calpains activity in migrating melanoma cells. A very interesting study has indeed shown an asymmetrical distribution of calpain-2 in neutrophils, which is recruited at the leading edge of the cell during early pseudopod formation to promote directionality (Nuzzi *et al*, 2007).

*****Minor points:*****

The authors should mention the published literature on the role of TRPM1 in melanoma (especially progression to metastatic disease) in their introduction.

In order to give a better overview of the literature on the role of TRPM1, as well as other TRP members, in the specific context of melanoma progression, we mentioned their roles in the Introduction **(pages 3 and 4)**. More information can also be found in answer to the minor remark 1 of Reviewer#3.

Could the authors speculate on the apparent melanoma specificity of TRPV2 expression (figure 1)?

Several hypotheses can be raised regarding the predominant expression of TRPV2 in melanoma cells. For instance, it could result from **transcription factors** specifically expressed in the melanocytic lineage (such as MITF, SOX10, PAX3…) coupled to melanoma deregulated transcriptional pathways that would drive TRPV2 expression. This hypothesis is further discussed above, in response to the major point 1.
Another explanation for this apparent specificity of melanoma cells could be related to the **neural crest origin of the melanocytes**. Indeed, parallels exist between the highly invasive nature of metastatic melanoma and their neural crest/melanoblast precursors. Multiple studies have suggested that melanoma reactivates neural crest migration programs to drive plasticity and invasiveness (Diener & Sommer, 2021; Sommer, 2011). Intriguingly, TRPV2 transcripts are present in pre-migratory neural crest cells from embryos (Hutson *et al*, 2017), and was shown to regulate axon outgrowth through its activation by membrane stretch during development (Shibasaki *et al*, 2010). This hypothesis is not only linking TRPV2 expression to the neural origins of melanoma cells, but also to common functional features of these cells related to motility.
Finally, TRPV2-specificity in melanoma cells could also be associated with the specific type of migration these cells engage for **rapid plasticity**. As particularly invasive and disseminating cells, melanoma cells can adopt features of both mesenchymal and amoeboid migration (Gabbireddy *et al*, 2021). The mesenchymal-amoeboid transition (MAT) has been proposed as a mechanism for cancer cells to adapt rapidly their migration mode to their environment. This conversion to amoeboid, enabling a low-adhesive and energy-conserving migration strategy, has been described as involving calpain-mediated cleavage of talin in metastatic breast cancer and head and neck carcinoma cells, and therefore according to our mechanistic model might require TRPV2 activation (te Boekhorst *et al*, 2020). Note that this unique phenotypic switch

from mesenchymal to fast amoeboid migration also occurs in immune cells, where TRPV2 is actually highly expressed (Liu *et al*, 2015). More specifically, TRPV2 has been involved in migration and chemotaxis in neutrophils and monocytes/macrophages (Santoni *et al*, 2013).

These hypotheses are not mutually exclusive and could actually be intertwined. Our *in silico* and *in vitro* data **(appendix Figures S1D-E, S4** and **Review additional Figure 2)** are supporting an association of TRPV2 expression with a specific signature for cells endowed with unique plasticity potential involving amoeboid migration (de Winde *et al*, 2021; Rodriguez-Hernandez *et al*, 2020). This point is further developed in response to the comment below regarding MITF/AXL phenotypic signatures. To further elaborate the manuscript, this hypothesis was briefly mentioned in the discussion (**pages 14,16**). However, in-depth investigations would be required to bring a thorough answer to this compelling question.

Page 10 ***(now page 6)****, how was the invasiveness of melanoma cell lines measured? The authors should refrain from claiming that there is a "positive correlation between invasiveness and TRPV2 expression" (page 10) based on results from just 4 human melanoma cell lines. To support their claim, they would need to expand their panel of cell lines and/or use appropriate statistics (such as Pearson's correlation test).*

The invasiveness of the cell lines used in the present study were initially relying on reports from the literature, where their invasive potentials was assessed *in vitro*, or *in vivo* based on their ability to metastasize (Arozarena & Wellbrock, 2017; Herlyn *et al*, 1990; Juhasz *et al*, 1993; Tichet *et al*, 2015). Our own *in vitro* **(Figure 3A)** and *in vivo* **(Figure 6A-D)** analysis confirmed the strong migrative/invasive potentials of the WM266.4 and 451Lu cells, as compared to the poorly invasive 501mel. Invasiveness was further assessed by measuring the levels of POU3F2/BRN2 (**Figure 1H**) considered as a marker of invasiveness (Arozarena *et al*, 2011b; Thomson *et al*, 1995), and the expression of active (non-phospho) b-catenin (**appendix Figure S4**) described as a suppressor of invasion in melanoma (Arozarena *et al*, 2011a).
Moreover, and as suggested, we applied a Pearson's correlation test to the NHEM plus the 4 main cell lines used in this study (**Figure S1D**, $r_{pearson}=0.8946$, $P=0.0202$), as well as to the larger dataset of melanoma cell lines from the CCLE (**Figure S1E**, $r_{pearson}=0.3353$, $P=0.0019$), and showed a correlation between TRPV2 and POU3F2/BRN2 expressions.
For clarity purpose, we have modified the results section (**page 6**) as follow:
[While very low levels of TRPV2 mRNAs were present in normal human epithelial melanocytes (NHEM), a gradual increase of TRPV2 transcripts correlates with the rise of BRN2 expression in melanoma cells (ranging from the non-invasive 501mel, to the superficial spreading melanoma WM793, then to the metastatic melanoma WM266.4 and 451Lu (Arozarena et al., 2011a; Arozarena et al., 2011b; Arozarena & Wellbrock, 2017; Herlyn et al., 1990; Juhasz et al., 1993; Tichet et al., 2015) ***(Fig 1H and appendix S1D)****. In the broader CCLE melanoma cell lines dataset, the overall expressions of TRPV2 and POU3F2(BRN2) were also correlated* ***(appendix Fig S1E)****. In addition to TRPV2 mRNA expression, both TRPV2 protein levels* ***(Fig 2A)*** *and its functionality, assessed upon TRPV2 channel over-activation with the potent agonist cannabidiol (CBD) (Qin et al, 2008) (****appendix Figure S2A****), mimicked the expression of the BRN2 invasiveness marker. Altogether suggesting a correlation between the expression of functional TRPV2 channels and the BRN2-associated invasive phenotype of melanoma cell lines.]*

Could the authors examine the potential correlation between TRPV2 expression and MITF and AXL levels, which are well-accepted markers of the balance between growth and migration in melanoma?

To rapidly adapt to the tumor microenvironment, melanoma cells can switch their molecular and cellular phenotype, including in an epithelial-to-mesenchymal transition (EMT)-like manner. Conventionally, phenotypic changes were linked to the expression levels of MITF (microphthalmia-associated transcription factor) and AXL, in order to regulate differentiation, proliferation and metabolic rewiring. MITF is a melanocyte lineage-specific transcription factor, considered to be the ‘‘master regulator of melanocytes’’

because it is essential for melanoblast survival and melanocyte lineage commitment, and it regulates the expression of melanogenic enzymes. MITF has been proposed as a read-out of the phenotype switching model, with $MITF^{high}$ cells characterized as proliferative, and $MITF^{low}$ as invasive (Hoek *et al*, 2008a), acting as a rheostat where the levels of MITF would determine the phenotypic state (Rambow *et al*, 2019). During the invasion process, melanoma cells exhibit a de-differentiated phenotype, characterized by low pigmentation and reduced proliferation, whereas upon metastatic growth, cells switch back to a differentiated, pigmented and proliferative phenotype. These phenotypic switches, as well as MITF expression level changes are guided by the distinct tumor microenvironments.

Despite all the evidence suggesting that MITF must be down-regulated for melanoma progression, *MITF* amplification is frequent in melanoma and particularly common in metastatic forms of the disease where it is associated with poor survival (Garraway *et al*, 2005). The MITF gene is actually amplified in 10–15% of melanomas in which BRAF is mutated, as it is the case for the 451Lu invasive cell line (Garraway *et al.*, 2005; Wellbrock *et al*, 2008). Moreover, these reports showed that oncogenic BRAF stimulates MITF transcription in a BRN2-dependent manner, and further correlated BRN2 and MITF expression in human melanoma samples. The relationship between MITF and BRN2 seems complex, since BRN2 has also been described as expressed in a predominantly mutually exclusive pattern with MITF. Of note, BRN2 is expressed in melanoma but not in melanocytes. In line with the previous reports, while MITF was required to maintain proliferation *in vitro*, both MITF and BRN2 expression have been shown necessary for the development of melanoma metastasis *in vivo* (Simmons *et al*, 2017). Finally, a considerable fraction of circulating tumor cells isolated from invasive melanoma patients was MITF positive (Khoja *et al*, 2014), supporting the view that continued expression of MITF is essential in metastatic melanoma cells. Hence, the connection between MITF and melanoma invasiveness might not be as straightforward as anticipated.

We analyzed MITF/AXL expression levels in 4 subpopulations of melanoma cells issued from human biopsies, classified according to their differentiation states (Tsoi signatures). The 4 phenotypes, namely, undifferentiated, neural crest like, transitory and melanocytic, can be defined by the expression of a defined set of genes, notably the so-called "MITF/AXL rheostat" **(Review additional Figure 2A)**. As expected, undifferentiated and neural crest melanomas were $MITF^{low}/AXL^{high}$, whereas the opposite was seen in the 2 further differentiated subpopulations. Regarding TRPV2, although present in all 4 melanoma subtypes, its expression was slightly higher in the population with the melanocytic signature ($MITF^{high}/AXL^{low}$). Correlation analysis in the TCGA SKCM dataset further showed that TRPV2 expression was positively correlated to MITF expression, and rather negatively to AXL expression **(Review additional Figure 2B)**. As we mentioned in the discussion (**page 14**), while 501mel cells exhibit pseudo-epithelial markers (including MITF), and inversely WM266.4 display mesenchymal markers and a morphology evoking a complete mesenchymal transition, 451Lu cells were associated with an intermediate/partial phenotype, such as less viculin-containing adhesions, less N-cadherin and less vimentin, and a high MITF expression (**Figures 5A and appendix S4**). In line with this, it has been described that in cancer cells the amoeboid state is supported by both proliferative and pro-survival signaling (Graziani *et al*, 2022), with some amoeboid cells expressing concomitantly MITF and BRN2 (de Winde *et al.*, 2021). Interestingly, the highly invasive 451Lu cells, exhibiting a unique migratory phenotype with fewer engaged adhesions, are expressing extremely high levels of TRPV2. Sommer and colleagues further showed that in melanoma cells, NGFR overexpression promotes metastasis formation, but is associated with a gene expression program that is not overlapping with the previously established MITF/AXL expression signatures defining proliferative *versus* invasive cells (Restivo *et al*, 2017). They concluded that these processes were either independent of previously reported invasive transcriptional programs or were representative of transient states of cells undergoing dynamic switching. Later on, they reported that the down-regulation of SMAD7, a major negative regulator of the TGF-β signaling pathway, can overcome the need for the "phenotype switching" during melanoma tumor progression, since conditional *Smad7* deletion led to sustained melanoma growth coupled to metastasis formation (Tuncer *et al*, 2019). Importantly, low SMAD7 levels in patient tumors are associated with poor survival. Interestingly, our correlation analysis in the TCGA SKCM dataset revealed that SMAD7 expression is negatively correlated to both TRPV2 and MITF expressions **(Review additional Figure 2C)**.

Hence, contrary to the conventional "phenotype switching" model, melanoma progression can involve malignant melanoma cells simultaneously displaying proliferative and invasive properties, presenting unique migratory signatures, most likely accounting for the intrinsic cellular plasticity and heterogeneity of melanomas (Kemper *et al*, 2014; Tirosh *et al*, 2016). Conceivably, TRPV2 expression together with MITF expression may be associated with melanomas unique cellular plasticity.

How does TRPV2 overexpression in Figure 2C compare with normal expression levels in invasive cell lines?

When we compared TRPV2 expression at the protein level within the different cell lines, we observed a very similar level of expression between 501mel-TRPV2-GFP and the invasive 451Lu cells while 501mel-GFP were barely expressing TRPV2 (**appendix Figure S4**).

Could the authors also show talin and TRPV2 staining in Figure 4?

As stated above, although Talin is a central mechanosensitive adaptor protein in early integrin-mediated contacts, Talin cleavage was initially used as a second-line readout of calpain activity, and we unfortunately did not perform any Talin immunostaining.

Talin participates very early in adhesomes formation and is an important calpain-2 target to induce adhesion turnover (Critchley & Gingras, 2008; Klapholz & Brown, 2017). However, other calpain substrates than Talin, such as Paxillin and Filamin A, another cell motility modulator (Lamsoul *et al.*, 2020) could be concomitantly involved in melanoma cells migration. In order to deepen our understanding of the mechanistic events downstream of calpain activation, the analysis of several proteolytic targets of calpains should be considered. Analysis of their cleavage status, along with high-resolution microscopy approaches to define their precise subcellular localization, and whether these substrates belong to nascent adhesion sites, would be highly informative and we are planning on further exploring this mechanism in follow up studies.

Were the FA analyses presented in Figure 5 performed on fibronectin-coated plates? Please consider adding these details in the figure legends.

Analysis of mature FA numbers was indeed performed on low confluence melanoma cells seeded onto fibronectin-coated coverslips. We apologize for the oversight and amended the legend of **Figure 5 (page 38)** accordingly.

Could the authors provide more explicit statistical analyses on Figures 6 and 7D?

We apologize for the oversight and we have included statistical analyses on **Figures 6B,D** (unpaired t-test) and **7E** (previously 7D) (Anova multiple comparison). Please see also the above response to Reviewer#1's minor issues.

How do the authors explain that TRPV2 colocalizes with paxillin and not phospho-FAK (in contrast to what is depicted on Figure 7G)?

In the invasive melanoma models, we indeed observed clusters of TRPV2 and Paxillin (Pax), but could not detect any colocalization of the channel with pFAK.

Pax is one of the most important proteins for structure and function of integrin-mediated adhesions. As a scaffolding protein, Pax is recruited within early adhesomes at the cell front for the assembly of nascent adhesion complexes by integrin-activators such as Talin, to ultimately lead the adhesion dynamics and cytoskeleton reorganization (Lopez-Colome *et al*, 2017). Pax is also required for the disassembly of FAs at the rear end of the cell. Interactions of Pax with its binding partners are mostly regulated by its phosphorylation status at multiple Tyr and Ser residues. In adhesion sites, p-FAK preferentially complexes with phosphorylated Y31 and Y118-Pax in a periodic pattern (Choi *et al*, 2011; Digman *et al*, 2008), forming very dynamic, force-sensitive, confined and discrete clusters. The remaining adhesion area containing Pax organizes pFAK-independent signaling and adhesion (Bachmann *et al*, 2020). Hence, although FAK is recruited from the cytoplasm to adhesion sites by Pax, Pax interaction with and phosphorylation by FAK is not essential for Pax localization to adhesion sites (Brown *et al*, 1996). In fact, the binding site for FAK is

located at the N-term domain, whereas the sequence for localization at adhesions resides in the C-term region of Pax. Studies on the hierarchical assembly of adhesions have shown that the structure of the adhesive platforms may vary according to the distinct combinations of integrin receptors activating specific signaling pathways (Zaidel-Bar *et al*, 2003). Nevertheless, Pax recruitment to the leading edge of membrane protrusions is an early event in the adhesive process (Laukaitis *et al*, 2001; Petropoulos *et al*, 2016).
However we can not exclude that few engaged adhesion structures encompassing TRPV2-Pax-pFAK complexes, if present, were too discrete and dynamic to be detected without super-resolution microscopy techniques. This might explain the very sparse colocalization spots observed in the highly invasive 451Lu cells **(Figure 4Ah)**. Although FAK/p-FAK and Src/p-Src are highly expressed in both metastatic cell lines (WM266.4 and 451-Lu) as compared to the nonmetastatic 501mel cells, the modulation of TRPV2 expression (either overexpressed or silenced) did not affect both kinases expression (own unpublished data), further suggesting that, at least at this stage, Pax does not require a p-FAK-dependent signaling. Hence, based on the literature, we concluded that the co-clustering of TRPV2 and Pax observed in migrating melanoma cells occurs within nascent adhesion sites, which at this early stage does not involve a stable interaction with pFAK. The simplified schematic model in **Figure 8** has been modified accordingly.

The mechanosensitive nature of TRPV2 is not addressed experimentally in this manuscript. Please state published results more explicitly when referring to this aspect of TRPV2 activity regulation (for example on pages 13 and 19).

We apologize for this lack of clarity. The mechanosensitive properties of TRPV2, although well described in the literature as one of its specificity (among the main references: (Katanosaka *et al*, 2018; Nagasawa & Kojima, 2015; Sugio *et al*, 2017; Yu *et al*, 2021b), has indeed not been addressed here in the context of melanoma progression. This aspect is undoubtedly of great interest, but requires highly specific approaches. Hence, the text throughout the manuscript has been clarified accordingly.

__Reviewer #3:__

****Minor remarks:****

1. A description of the TRP family in the introduction would be helpful: How is the TRPV2 channel different from other family members? Which normal tissues is TRPV2 highly expressed? Most importantly, why did the authors decide to focus on TRPV2 since several other TRP proteins are highly expressed in melanomas including TRPM1 and TRPM7.

A brief presentation of the TRP channels superfamilies, as well as the known roles of TRP members, such as TRPM1 and TRPM7, in melanoma progression has been added to the Introduction **(pages 3 and 4)**. With regards to TRPV2 specificity towards the other members of the TRP family and its tissue-specific expression, we have added this information in the discussion of the manuscript **(page 13)**. However, to clarify the specific interest shown towards TRPV2, we believe it was necessary to further develop our rationale here. Indeed, in our analysis of Ca^{2+}-permeable channels mRNA levels in SKCM tumors **(Figure 1A)**, the expression of other TRP members, such as TRPM1 and TRPM7 were substantial, although lower than TRPV2 expression.

TRPM1 (where M stands for Melastatin), the founding member of the TRPM channels, is indeed described as important in melanocyte function and malignant melanoma pathophysiology. However TRPM1-expression correlates positively with the differentiation status of melanocytes and, inversely, with the aggressiveness and tumor thickness of malignant melanoma (even used as a good prognostic marker). TRPM1 expression was found to correlate with melanin content (Oancea *et al*, 2009) but downregulated in melanoma **(Figure 1B)**. In conjunction with TRPM1, a microRNA (miR-211) located within the TRPM1 gene is also described as playing an important role in tumor suppression (Boyle *et al*, 2011; Mazar *et al*, 2010). Taken together these data were not in favor of TRPM1 as a Ca^{2+}-regulating element of advanced melanoma progression.

Regarding TRPM7, it is the best studied TRP channel in the context of metastasis, and its overexpression has been observed in primary breast cancer tumors (Middelbeek *et al*, 2012), in ductal adenocarcinoma (Guilbert *et al*, 2009) and ER negative invasive ductal cancer (Guilbert *et al*, 2013), in malignant pancreatic tumors (Rybarczyk *et al*, 2017; Yee *et al*, 2015), in metastatic nasopharyngeal tumors (Chen *et al*, 2015) and in bladder cancer (Gao *et al*, 2017). Elevated TRPM7 expression was associated with poor prognosis and metastasis in most of these cancer types. Concerning melanoma tumors, in our study, a high expression of TRPM7 was indeed observed when querying tumors from the SKCM data set **(Figure 1A)**, yet the qPCR analysis on metastatic melanoma cell lines showed little expression of TRPM7 especially in comparison to TRPV2 expression (over 10 times less, **Figure 1C,D**). Consistently, TRPM7 expression has been shown overall steady in both melanocytes and melanoma cells, but most importantly did not increase together with the invasive phenotypes of melanoma cell lines (McNeill *et al*, 2007). Additionally, *in silico*, TRPM7 expression did not come out as a poor prognosis factor in melanoma tumors (data not shown). As tumor biopsies consist of cancer cells as well as immune cells, CAFibroblast and cells from the tumoral microenvironment, we hypothesized that TRPM7 was mostly overexpressed in other cell types than in melanoma cells, presumably in fibroblast. Moreover, as our initial goal was to identify a therapeutic target specific for the treatment of melanoma, we did not select TRPM7 as a potential candidate because of its ubiquitous expression. In any event, we firmly believe that studying the role played by TRPM7, as well as several other TRP family members, in advanced melanoma progression would be of great interest.

As for the reasons why we have focused on TRPV2, not only it was the most expressed channel in human SKCM tumors (**Figure 1A)**, and its overexpression was specific to melanoma lesions compared to nevi samples (A new panel containing a heatmap comparing TRP channels RNA expression levels in nevi and melanoma has been added in **Figure 1B** and a detailed analysis specific of TRPV2 expression is shown in **figure 7A)**, but also in metastatic melanoma cell lines TRPV2 expression was largely exceeding the expression of other TRP members (**Figure 1C-D)**, and finally its overexpression largely prevailed in melanoma in comparison to several other cancer types (**Figures 1E-F and appendix S1A-B**). Although the

mechanistic basis for TRPV2 mechanotransduction potential was up to now unexplained, the mechanosensitive properties of this channel are recognized as one of its specificity (Katanosaka *et al.*, 2018; Nagasawa & Kojima, 2015; Sugio *et al.*, 2017; Yu *et al.*, 2021b). The few studies reporting a role for TRPV2 in cancer progression were correlating its expression and translocation to the cell membrane (a regulatory mechanism that is specific to TRPV2 (Kojima & Nagasawa, 2014)) with tumor cells migration (Monet *et al*, 2009; Monet *et al*, 2010; Oulidi *et al*, 2013), making this mechanosensitive channel a good target for the acquisition of the metastatic potential in melanoma. Additionally, its closest phylogenetic family member, TRPV1, is described as a valuable therapeutic target in pain treatment, with specific pharmacological tools developed that have entered clinical trials, supporting a promising potential for the development of therapeutic treatment targeting TRPV2. Understanding the role of TRPV2 in regulating cell migration in the tumoral cell type exhibiting its highest level of expression, could eventually turn out beneficial for the treatment of the other cancer types involving TRPV2, including prostate and breast cancers.

2. Figure 2A: It would be better to include a cell surface protein as loading control on this Western blot.

A cell surface protein as a loading control would have indeed been ideal. Among the most common normalizing membrane proteins are beta-catenin and N-cadherin. However, as both proteins are involved in adhesion, their levels of expression are very different in the various melanoma cell lines we have tested **(appendix Figure S4)**. The Na^{+}/K^{+}-ATPase pump is another classically used plasma membrane loading control. While present in melanoma, its expression level is highly variable and depends on disease progression (Boukerche *et al*, 2004; Mathieu *et al*, 2009). Conceivably due to the heterogeneity of the melanoma cells, we have been unable to find an ubiquitous cell surface protein, whose expression level is consistent regardless of the cell types tested. This is why we have opted for Actin as loading and cell integrity control in these experiments where, as expected, actin is absent from the cell surface proteins fractions. Please note that the cell surface biotinylation assay is a robust technique to isolate and compare the amount of cell surface proteins from samples with an equivalent quantity of total protein and that the observations made with this method were confirmed using two alternative approaches namely, immunostaining and channel activity assays.

3. Figure 2A: Why is the upper band of different sizes? Has the TRPV2 channel been glycosylated differently in different cell lines? Or do these represent different splice forms?

According to the size of the multiple bands observed on TRPV2 immunoblots, the upper bands correspond to glycosylated forms of TRPV2. It has been reported that both high mannose-type glycosylation and complex glycosylation of TRPV2 occur (Jahnel *et al*, 2003). Overexpression recombinant TRPV2 in HEK cells further confirmed that anti-TRPV2 immunoreactivity appears as doublet, with similar size as those observed in metastatic melanoma cell lines **(appendix Figure S10A)**. In melanoma cells as well as in leukemia cells, TRPV2 upper band can be actually composed of several bands, likely corresponding to variable glycosylation patterns of TRPV2, differing according to the cell lines **(Figure 1G)**. Interestingly, studies have suggested that TRPV2 glycosylation plays a role in its trafficking towards the plasma membrane and would play an anchoring role (Jahnel *et al.*, 2003), which perfectly correlates with our cell-surface biotinylation assays where the upper glycosylated bands were majoritarily detected in the plasma membrane fraction **(Figure 2A)**.
Up to now, only short-splice variants of TRPV2, s-TRPV2, have been reported. These short variants have been found in normal human urothelial cells and bladder tissue specimens (where it was lost as an early event in bladder carcinogenesis) (Caprodossi *et al*, 2008), as well as in human macrophages (Nagasawa *et al*, 2007). They both lack the pore-forming region and the sixth transmembrane domains, where the glycosylation site is located. In our own unpublished data, we have observed that s-TRPV2 variants mRNAs are barely expressed in melanoma cells.

4. Statistical significance is not shown changes in figure S3. Thus, the claim on page 11 "Although, TRPV2 overexpression increased the growth rate of the non-invasive 501mel cells, TRPV2 silencing had no impact on the viability nor on ERK phosphorylation in either metastatic cell lines, suggesting that TRPV2 is dispensable for malignant melanoma proliferative/survival (Figures S3A-B)" is not validated.

We apologize for the oversight and we have now included statistical analyses (2way ANOVA) in **Figure EV1A (previously S3)**. A statistically significant difference was only seen when comparing the 501mel-GFP cells to their counterpart overexpressing GFP-TRPV2 (***P*=0.0038). As for the invasive 451Lu cells, the tenuous variations between the sh-Ctrl and both sh-TRPV2 expressing cells did not reach significance (*P*=0.9982 for sh-V2-1, and *P*=0.5755 for sh-V2-2). Regarding the WM266.4 cells, considering the rather slow growth curves initially presented we undertook additional experiments with optimized seeding density to improve the quality of these data. The manuscript has been updated accordingly, and shows no statistical difference between the sh-Ctrl *versus* the sh-V2-1 (*P*=0.8422), or *versus* the sh-V2-2 (*P*=0.9491). Moreover, we would like to highlight the fact that (as stated in the manuscript) no significant difference was observable in the size of the melanospheres formed by the WM266.4 cells, whether they were expressing TRPV2 or not **(Figure 3D)**.

5. Did OE and KD TRPV2 show changes in cell morphology?

Although we did not perform a thorough morphological analysis of the melanoma cell lines used in this study, we could observe very subtle alterations in their apparent shape upon either TRPV2 expression or silencing. For instance, when overexpressing TRPV2, the 501mel cells seem to display more protrusions (dendritic-like) compared to the control cells which have a more classical “flat polygonal” shape. But these changes have to be properly analyzed and quantified. Regarding the WM266.4 and 451Lu cells, despite both having highly invasive potential, they display distinct morphological features. WM266.4 are well spread polarized cells with protrusions and forming monolayers in culture, while 451Lu are very heterogeneous, some rounder, and with a propensity to grow in a clonal-type manner forming multilayers clusters, which is once again fitting with their intermediate mesenchymal-amoeboid phenotype (described in the discussion **page 14** and above in response to a couple of minor points issued by the Reviewer#2). While beyond the scope of this manuscript, an in-depth morphological analysis of melanoma cells, coupled to investigations of their morphologic and phenotypic migration features depending on the substrate, would definitely widen our understanding of the role played by TRPV2 in regulating melanoma metastatic migration.

6. The authors should discuss why the mice injected with GFP-TRPV2 overexpressing cells showed numerous metastatic foci in lungs, brain and bones, whereas, "TRPV2 repression prevented the extravasation of melanoma cells into the lungs" only?

When comparing the *in vivo* metastatic potentials of the 501mel cells overexpressing TRPV2-GFP with the 451Lu (endogenously highly expressing TRPV2), we indeed noticed that metastasis formation preferentially occurred in the lungs of 451Lu-injected mice, while mainly occurring in both the lungs and the bones of 501mel-TRPV2-GFP-injected mice **(Figures 6, EV4 and appendix S7-8)**.

Following the injection in the tail vein, cells are rapidly reaching the heart and then the lungs. At this stage, depending on their capacity to cross barriers and therefore invasive potentials, they may extravasate to form distant metastasis. Due to the experimental model, the lungs will therefore be a preferential site for extravasation and metastatic development. Moreover, we speculate that the 451Lu cells display a strong tropism for the lungs due to tumor-derived intrinsic properties of these cells. Indeed, 451Lu (where Lu stands for Lung) melanoma cells were selected from lung metastases of xenografted nude mice after several *in vivo* passages of human melanoma WM164 cells, previously isolated from a stage IV melanoma metastasis of a patient (Herlyn *et al.*, 1990). During the selection process while accumulating enhanced invasiveness, 451Lu cells might have also acquired a high tropism for the lung. Note that, although to a lower extent, 451Lu also metastasized in other organs of the mice.

By contrast, 501mel are melanoma cells of unknown primary histologic origins endowed with very low invasive potentials. Their intrinsic properties in terms of crossing endothelial barriers, intravasation,

extravasation and so forth are therefore likely limited. Among the 501mel-TRPV2-injected mice that developed metastasis, foci were localized in the lungs, but also in the bones and other organs. For instance, bones could provide a propitious microenvironnement for TRPV2-overexpressing cells to migrate to, due to a high extracellular Ca^{2+} concentration.

7. Page 15 : "The TCGA skin melanoma RNAseq dataset was first compared to the matched TCGA and GTEx normal datasets (Figure S13A)." The authors should clarify which subset of the GTEx normal dataset was used.

We apologize for the lack of clarity. The analyses presented in **appendix Figure S11A** (previously S13A) were done using the GEPIA online server (http://gepia2.cancer-pku.cn/), providing tools for differential expression analysis using TCGA tumor samples with paired adjacent TCGA normal samples and GTEx normal samples. To integrate the TCGA and GTEx data for comprehensive expression analysis, this server uses UCSC Xena project recomputed TCGA and GTEx raw RNA-Seq data, which makes the two datasets compatible. The normal skin GTEx dataset used by GEPIA includes RNA sequencing data of 557 samples from a non-specified subset, knowing that the GTEx Analysis Release V8 (dbGaP Accession phs000424.v8.p2) encompasses two distinct normal skin subsets: Not sun-exposed (suprapubic), n=604, and sun-exposed (lower leg) n=701. In any event, the median log2(TPM+1) values for TRPV2 expression in these two subsets were 2.86 and 3.11, respectively. Hence, despite a slight increase in sun-exposed *versus* not sun-exposed skin, TRPV2 expression level remains significantly higher in SKCM tumors (median log2(TPM+1)=6.3, **appendix Figure S11A**).
Since melanocytes represent only a small cellular fraction of normal skin, the choice of the "normal skin" dataset is of high importance and could definitely impact the conclusions. In order to strengthen our data, we have included analyses of TRPV2 expression based on the GSE46517 microarray gene expression dataset (**Figure 7A**) including human samples from normal skin, nevi, primary and metastatic melanoma tumors (Kabbarah *et al*, 2010). The results confirmed TRPV2 overexpression in melanomas compared to normal skin, but most importantly showed that in melanocytes-composed benign nevi, TRPV2 expression remained low, significantly below the level of expression observed in melanomas.

8. Page 25: Figure 1 legends: add T to the at the beginning of the sentence "The Ca2+- permeable channels plotted on the x-axis are grouped by family...."

We have meticulously revised the manuscript and all the following suggested modifications in point 8 and 9 have been included.

9. English needs to be improved throughout the manuscript. A few examples are listed below:

a.Abstract: „...is of primary importance (since being) AS IT IS the main cause..."

b.Abstract: „ Here, we (evidenced a) SHOW THE prominent expression...."

c.Introduction: „..The scope of this study was to identify AN ATYPICAL PROFILE among the numerous...."

d.Materials and Methods: Mice model should be Mouse models.

e.Results: „TRPV2 TRANSCRIPTS STOOD OUT AS THE MOST EXPRESSED among (most members of) the major Ca2+ permeable channels...."

f.„Importantly, the (utmost expression of) TRPV2 transcripts (was) WERE (also revealed) SHOWN TO BE HIGHLY EXPRESSED in SKCM tumours AS compared to...."

g.Page 12: The sentence: "In these define proximal clusters..." Needs to be rearranged or clarified. (Now page 9)

h.Discussion: „Nevertheless, modulating TRPV2 expression - either way - had no impact on THE EXPRESSION OF EMT markers.„ (Now page 14)

i.Discussion: „..the highly invasive WM266.4 cells (were) exhibitED antagonistic markers profiles...."

j.Discussion: „These observations were (in adequation) CONSISTENT with .....as well as with (their levels of) THE EXPRESSION OF THE BRN2 invasiveness marker."

k.The sentence „Therefore, globally impacting resting...." lacks an ending.

l.„(By being recruited) RECRUITMENT OF TRPV2 at the PM within paxillin-rich.....structures PLACES THIS mechanosensitive channel at...." (Now pages 16-17)

References :

Arozarena I, Bischof H, Gilby D, Belloni B, Dummer R, Wellbrock C (2011a) In melanoma, beta-catenin is a suppressor of invasion. *Oncogene* 30: 4531-4543

Arozarena I, Sanchez-Laorden B, Packer L, Hidalgo-Carcedo C, Hayward R, Viros A, Sahai E, Marais R (2011b) Oncogenic BRAF induces melanoma cell invasion by downregulating the cGMP-specific phosphodiesterase PDE5A. *Cancer Cell* 19: 45-57

Arozarena I, Wellbrock C (2017) Targeting invasive properties of melanoma cells. *FEBS J* 284: 2148-2162

Bachmann M, Skripka A, Wehrle-Haller B, Bastmeyer M (2020) Phosphorylated paxillin and FAK constitute subregions within focal adhesions. *bioRxiv*: 2020.2009.2024.311126

Boukerche H, Su ZZ, Kang DC, Fisher PB (2004) Identification and cloning of genes displaying elevated expression as a consequence of metastatic progression in human melanoma cells by rapid subtraction hybridization. *Gene* 343: 191-201

Boyle GM, Woods SL, Bonazzi VF, Stark MS, Hacker E, Aoude LG, Dutton-Regester K, Cook AL, Sturm RA, Hayward NK (2011) Melanoma cell invasiveness is regulated by miR-211 suppression of the BRN2 transcription factor. *Pigment Cell Melanoma Res* 24: 525-537

Brown MC, Perrotta JA, Turner CE (1996) Identification of LIM3 as the principal determinant of paxillin focal adhesion localization and characterization of a novel motif on paxillin directing vinculin and focal adhesion kinase binding. *J Cell Biol* 135: 1109-1123

Canales Coutino B, Mayor R (2021) The mechanosensitive channel Piezo1 cooperates with semaphorins to control neural crest migration. *Development* 148

Caprodossi S, Lucciarini R, Amantini C, Nabissi M, Canesin G, Ballarini P, Di Spilimbergo A, Cardarelli MA, Servi L, Mammana G *et al* (2008) Transient receptor potential vanilloid type 2 (TRPV2) expression in normal urothelium and in urothelial carcinoma of human bladder: correlation with the pathologic stage. *Eur Urol* 54: 612-620

Chen JP, Wang J, Luan Y, Wang CX, Li WH, Zhang JB, Sha D, Shen R, Cui YG, Zhang Z *et al* (2015) TRPM7 promotes the metastatic process in human nasopharyngeal carcinoma. *Cancer Lett* 356: 483-490

Chen X, Wanggou S, Bodalia A, Zhu M, Dong W, Fan JJ, Yin WC, Min HK, Hu M, Draghici D *et al* (2018) A Feedforward Mechanism Mediated by Mechanosensitive Ion Channel PIEZO1 and Tissue Mechanics Promotes Glioma Aggression. *Neuron* 100: 799-815 e797

Chen YF, Chiu WT, Chen YT, Lin PY, Huang HJ, Chou CY, Chang HC, Tang MJ, Shen MR (2011) Calcium store sensor stromal-interaction molecule 1-dependent signaling plays an important role in cervical cancer growth, migration, and angiogenesis. *Proc Natl Acad Sci U S A* 108: 15225-15230

Choi CK, Zareno J, Digman MA, Gratton E, Horwitz AR (2011) Cross-correlated fluctuation analysis reveals phosphorylation-regulated paxillin-FAK complexes in nascent adhesions. *Biophys J* 100: 583-592

Coste B, Mathur J, Schmidt M, Earley TJ, Ranade S, Petrus MJ, Dubin AE, Patapoutian A (2010) Piezo1 and Piezo2 are essential components of distinct mechanically activated cation channels. *Science* 330: 55-60

Critchley DR, Gingras AR (2008) Talin at a glance. *J Cell Sci* 121: 1345-1347

D'Souza RS, Lim JY, Turgut A, Servage K, Zhang J, Orth K, Sosale NG, Lazzara MJ, Allegood J, Casanova JE (2020) Calcium-stimulated disassembly of focal adhesions mediated by an ORP3/IQSec1 complex. *Elife* 9

de Winde CM, George SL, Crosas-Molist E, Hari-Gupta Y, Arp AB, Benjamin AC, Millward LJ, Makris S, Carver A, Imperatore V *et al* (2021) Podoplanin drives dedifferentiation and amoeboid invasion of melanoma. *iScience* 24: 102976

Diener J, Sommer L (2021) Reemergence of neural crest stem cell-like states in melanoma during disease progression and treatment. *Stem Cells Transl Med* 10: 522-533

Digman MA, Brown CM, Horwitz AR, Mantulin WW, Gratton E (2008) Paxillin dynamics measured during adhesion assembly and disassembly by correlation spectroscopy. *Biophys J* 94: 2819-2831

Dilshat R, Fock V, Kenny C, Gerritsen I, Lasseur RMJ, Travnickova J, Eichhoff OM, Cerny P, Moller K, Sigurbjornsdottir S *et al* (2021) MITF reprograms the extracellular matrix and focal adhesion in melanoma. *Elife* 10

Gabbireddy SR, Vosatka KW, Chung AJ, Logue JS (2021) Melanoma cells adopt features of both mesenchymal and amoeboid migration within confining channels. *Sci Rep* 11: 17804

Gao SL, Kong CZ, Zhang Z, Li ZL, Bi JB, Liu XK (2017) TRPM7 is overexpressed in bladder cancer and promotes proliferation, migration, invasion and tumor growth. *Oncol Rep* 38: 1967-1976

Garraway LA, Widlund HR, Rubin MA, Getz G, Berger AJ, Ramaswamy S, Beroukhim R, Milner DA, Granter SR, Du J *et al* (2005) Integrative genomic analyses identify MITF as a lineage survival oncogene amplified in malignant melanoma. *Nature* 436: 117-122

Glading A, Lauffenburger DA, Wells A (2002) Cutting to the chase: calpain proteases in cell motility. *Trends Cell Biol* 12: 46-54

Graziani V, Rodriguez-Hernandez I, Maiques O, Sanz-Moreno V (2022) The amoeboid state as part of the epithelial-to-mesenchymal transition programme. *Trends Cell Biol* 32: 228-242

Guilbert A, Gautier M, Dhennin-Duthille I, Haren N, Sevestre H, Ouadid-Ahidouch H (2009) Evidence that TRPM7 is required for breast cancer cell proliferation. *Am J Physiol Cell Physiol* 297: C493-502

Guilbert A, Gautier M, Dhennin-Duthille I, Rybarczyk P, Sahni J, Sevestre H, Scharenberg AM, Ouadid-Ahidouch H (2013) Transient receptor potential melastatin 7 is involved in oestrogen receptor-negative metastatic breast cancer cells migration through its kinase domain. *Eur J Cancer* 49: 3694-3707

Herlyn D, Iliopoulos D, Jensen PJ, Parmiter A, Baird J, Hotta H, Adachi K, Ross AH, Jambrosic J, Koprowski H *et al* (1990) In vitro properties of human melanoma cells metastatic in nude mice. *Cancer Res* 50: 2296-2302

Hoek KS, Eichhoff OM, Schlegel NC, Dobbeling U, Kobert N, Schaerer L, Hemmi S, Dummer R (2008a) In vivo switching of human melanoma cells between proliferative and invasive states. *Cancer Res* 68: 650-656

Hoek KS, Schlegel NC, Eichhoff OM, Widmer DS, Praetorius C, Einarsson SO, Valgeirsdottir S, Bergsteinsdottir K, Schepsky A, Dummer R *et al* (2008b) Novel MITF targets identified using a two-step DNA microarray strategy. *Pigment Cell Melanoma Res* 21: 665-676

Hooper R, Zhang X, Webster M, Go C, Kedra J, Marchbank K, Gill DL, Weeraratna AT, Trebak M, Soboloff J (2015) Novel Protein Kinase C-Mediated Control of Orai1 Function in Invasive Melanoma. *Mol Cell Biol* 35: 2790-2798

Huang Z, Sun Z, Zhang X, Niu K, Wang Y, Zheng J, Li H, Liu Y (2019) Loss of stretch-activated channels, PIEZOs, accelerates non-small cell lung cancer progression and cell migration. *Biosci Rep* 39

Hung WC, Yang JR, Yankaskas CL, Wong BS, Wu PH, Pardo-Pastor C, Serra SA, Chiang MJ, Gu Z, Wirtz D *et al* (2016) Confinement Sensing and Signal Optimization via Piezo1/PKA and Myosin II Pathways. *Cell Rep* 15: 1430-1441

Hutson MR, Keyte AL, Hernandez-Morales M, Gibbs E, Kupchinsky ZA, Argyridis I, Erwin KN, Pegram K, Kneifel M, Rosenberg PB *et al* (2017) Temperature-activated ion channels in neural crest cells confer maternal fever-associated birth defects. *Sci Signal* 10

Iwata Y, Ito S, Wakabayashi S, Kitakaze M (2020) TRPV2 channel as a possible drug target for the treatment of heart failure. *Lab Invest* 100: 207-217

Jahnel R, Bender O, Munter LM, Dreger M, Gillen C, Hucho F (2003) Dual expression of mouse and rat VRL-1 in the dorsal root ganglion derived cell line F-11 and biochemical analysis of VRL-1 after heterologous expression. *Eur J Biochem* 270: 4264-4271

Juhasz I, Albelda SM, Elder DE, Murphy GF, Adachi K, Herlyn D, Valyi-Nagy IT, Herlyn M (1993) Growth and invasion of human melanomas in human skin grafted to immunodeficient mice. *Am J Pathol* 143: 528-537

Kabbarah O, Nogueira C, Feng B, Nazarian RM, Bosenberg M, Wu M, Scott KL, Kwong LN, Xiao Y, Cordon-Cardo C *et al* (2010) Integrative genome comparison of primary and metastatic melanomas. *PLoS One* 5: e10770

Katanosaka K, Takatsu S, Mizumura K, Naruse K, Katanosaka Y (2018) TRPV2 is required for mechanical nociception and the stretch-evoked response of primary sensory neurons. *Sci Rep* 8: 16782

Kemper K, de Goeje PL, Peeper DS, van Amerongen R (2014) Phenotype switching: tumor cell plasticity as a resistance mechanism and target for therapy. *Cancer Res* 74: 5937-5941
Khoja L, Shenjere P, Hodgson C, Hodgetts J, Clack G, Hughes A, Lorigan P, Dive C (2014) Prevalence and heterogeneity of circulating tumour cells in metastatic cutaneous melanoma. *Melanoma Res* 24: 40-46
Klapholz B, Brown NH (2017) Talin - the master of integrin adhesions. *J Cell Sci* 130: 2435-2446
Kojima I, Nagasawa M (2014) Trpv2. *Handb Exp Pharmacol* 222: 247-272
Lamsoul I, Dupre L, Lutz PG (2020) Molecular Tuning of Filamin A Activities in the Context of Adhesion and Migration. *Front Cell Dev Biol* 8: 591323
Laukaitis CM, Webb DJ, Donais K, Horwitz AF (2001) Differential dynamics of alpha 5 integrin, paxillin, and alpha-actinin during formation and disassembly of adhesions in migrating cells. *J Cell Biol* 153: 1427-1440
Liu S, Xu X, Fang Z, Ning Y, Deng B, Pan X, He Y, Yang Z, Huang K, Li J (2021) Piezo1 impairs hepatocellular tumor growth via deregulation of the MAPK-mediated YAP signaling pathway. *Cell Calcium* 95: 102367
Liu YJ, Le Berre M, Lautenschlaeger F, Maiuri P, Callan-Jones A, Heuze M, Takaki T, Voituriez R, Piel M (2015) Confinement and low adhesion induce fast amoeboid migration of slow mesenchymal cells. *Cell* 160: 659-672
Lopez-Colome AM, Lee-Rivera I, Benavides-Hidalgo R, Lopez E (2017) Paxillin: a crossroad in pathological cell migration. *J Hematol Oncol* 10: 50
Mathieu V, Pirker C, Martin de Lassalle E, Vernier M, Mijatovic T, DeNeve N, Gaussin JF, Dehoux M, Lefranc F, Berger W *et al* (2009) The sodium pump alpha1 sub-unit: a disease progression-related target for metastatic melanoma treatment. *J Cell Mol Med* 13: 3960-3972
Mazar J, DeYoung K, Khaitan D, Meister E, Almodovar A, Goydos J, Ray A, Perera RJ (2010) The regulation of miRNA-211 expression and its role in melanoma cell invasiveness. *PLoS One* 5: e13779
McNeill MS, Paulsen J, Bonde G, Burnight E, Hsu MY, Cornell RA (2007) Cell death of melanophores in zebrafish trpm7 mutant embryos depends on melanin synthesis. *J Invest Dermatol* 127: 2020-2030
Middelbeek J, Kuipers AJ, Henneman L, Visser D, Eidhof I, van Horssen R, Wieringa B, Canisius SV, Zwart W, Wessels LF *et al* (2012) TRPM7 is required for breast tumor cell metastasis. *Cancer Res* 72: 4250-4261
Monet M, Gkika D, Lehen'kyi V, Pourtier A, Vanden Abeele F, Bidaux G, Juvin V, Rassendren F, Humez S, Prevarsakaya N (2009) Lysophospholipids stimulate prostate cancer cell migration via TRPV2 channel activation. *Biochim Biophys Acta* 1793: 528-539
Monet M, Lehen'kyi V, Gackiere F, Firlej V, Vandenberghe M, Roudbaraki M, Gkika D, Pourtier A, Bidaux G, Slomianny C *et al* (2010) Role of cationic channel TRPV2 in promoting prostate cancer migration and progression to androgen resistance. *Cancer Res* 70: 1225-1235
Nagasawa M, Kojima I (2015) Translocation of TRPV2 channel induced by focal administration of mechanical stress. *Physiol Rep* 3
Nagasawa M, Nakagawa Y, Tanaka S, Kojima I (2007) Chemotactic peptide fMetLeuPhe induces translocation of the TRPV2 channel in macrophages. *J Cell Physiol* 210: 692-702
Newell-Litwa KA, Horwitz AR (2011) Cell migration: PKA and RhoA set the pace. *Curr Biol* 21: R596-598
Nuzzi PA, Senetar MA, Huttenlocher A (2007) Asymmetric localization of calpain 2 during neutrophil chemotaxis. *Mol Biol Cell* 18: 795-805
Oancea E, Vriens J, Brauchi S, Jun J, Splawski I, Clapham DE (2009) TRPM1 forms ion channels associated with melanin content in melanocytes. *Sci Signal* 2: ra21
Oulidi A, Bokhobza A, Gkika D, Vanden Abeele F, Lehen'kyi V, Ouafik L, Mauroy B, Prevarskaya N (2013) TRPV2 mediates adrenomedullin stimulation of prostate and urothelial cancer cell adhesion, migration and invasion. *PLoS One* 8: e64885
Pardo-Pastor C, Rubio-Moscardo F, Vogel-Gonzalez M, Serra SA, Afthinos A, Mrkonjic S, Destaing O, Abenza JF, Fernandez-Fernandez JM, Trepat X *et al* (2018) Piezo2 channel regulates RhoA and actin cytoskeleton to promote cell mechanobiological responses. *Proc Natl Acad Sci U S A* 115: 1925-1930
Petropoulos C, Oddou C, Emadali A, Hiriart-Bryant E, Boyault C, Faurobert E, Vande Pol S, Kim-Kaneyama JR, Kraut A, Coute Y *et al* (2016) Roles of paxillin family members in adhesion and ECM degradation coupling at invadosomes. *J Cell Biol* 213: 585-599

Poole K, Herget R, Lapatsina L, Ngo HD, Lewin GR (2014) Tuning Piezo ion channels to detect molecular-scale movements relevant for fine touch. *Nat Commun* 5: 3520
Qin N, Neeper MP, Liu Y, Hutchinson TL, Lubin ML, Flores CM (2008) TRPV2 is activated by cannabidiol and mediates CGRP release in cultured rat dorsal root ganglion neurons. *J Neurosci* 28: 6231-6238
Rambow F, Job B, Petit V, Gesbert F, Delmas V, Seberg H, Meurice G, Van Otterloo E, Dessen P, Robert C *et al* (2015) New Functional Signatures for Understanding Melanoma Biology from Tumor Cell Lineage-Specific Analysis. *Cell Rep* 13: 840-853
Rambow F, Marine JC, Goding CR (2019) Melanoma plasticity and phenotypic diversity: therapeutic barriers and opportunities. *Genes Dev* 33: 1295-1318
Restivo G, Diener J, Cheng PF, Kiowski G, Bonalli M, Biedermann T, Reichmann E, Levesque MP, Dummer R, Sommer L (2017) low neurotrophin receptor CD271 regulates phenotype switching in melanoma. *Nat Commun* 8: 1988
Rodriguez-Hernandez I, Maiques O, Kohlhammer L, Cantelli G, Perdrix-Rosell A, Monger J, Fanshawe B, Bridgeman VL, Karagiannis SN, Penin RM *et al* (2020) WNT11-FZD7-DAAM1 signalling supports tumour initiating abilities and melanoma amoeboid invasion. *Nat Commun* 11: 5315
Rybarczyk P, Vanlaeys A, Brassart B, Dhennin-Duthille I, Chatelain D, Sevestre H, Ouadid-Ahidouch H, Gautier M (2017) The Transient Receptor Potential Melastatin 7 Channel Regulates Pancreatic Cancer Cell Invasion through the Hsp90alpha/uPA/MMP2 pathway. *Neoplasia* 19: 288-300
Santoni G, Farfariello V, Liberati S, Morelli MB, Nabissi M, Santoni M, Amantini C (2013) The role of transient receptor potential vanilloid type-2 ion channels in innate and adaptive immune responses. *Front Immunol* 4: 34
Saxena M, Changede R, Hone J, Wolfenson H, Sheetz MP (2017) Force-Induced Calpain Cleavage of Talin Is Critical for Growth, Adhesion Development, and Rigidity Sensing. *Nano Lett* 17: 7242-7251
Seberg HE, Van Otterloo E, Loftus SK, Liu H, Bonde G, Sompallae R, Gildea DE, Santana JF, Manak JR, Pavan WJ *et al* (2017) TFAP2 paralogs regulate melanocyte differentiation in parallel with MITF. *PLoS Genet* 13: e1006636
Shibasaki K, Murayama N, Ono K, Ishizaki Y, Tominaga M (2010) TRPV2 enhances axon outgrowth through its activation by membrane stretch in developing sensory and motor neurons. *J Neurosci* 30: 4601-4612
Simmons JL, Pierce CJ, Al-Ejeh F, Boyle GM (2017) MITF and BRN2 contribute to metastatic growth after dissemination of melanoma. *Sci Rep* 7: 10909
Sommer L (2011) Generation of melanocytes from neural crest cells. *Pigment Cell Melanoma Res* 24: 411-421
Stokes AJ, Shimoda LM, Koblan-Huberson M, Adra CN, Turner H (2004) A TRPV2-PKA signaling module for transduction of physical stimuli in mast cells. *J Exp Med* 200: 137-147
Su LT, Agapito MA, Li M, Simonson WT, Huttenlocher A, Habas R, Yue L, Runnels LW (2006) TRPM7 regulates cell adhesion by controlling the calcium-dependent protease calpain. *J Biol Chem* 281: 11260-11270
Su LT, Chen HC, Gonzalez-Pagan O, Overton JD, Xie J, Yue L, Runnels LW (2010) TRPM7 activates m-calpain by stress-dependent stimulation of p38 MAPK and c-Jun N-terminal kinase. *J Mol Biol* 396: 858-869
Su LT, Liu W, Chen HC, Gonzalez-Pagan O, Habas R, Runnels LW (2011) TRPM7 regulates polarized cell movements. *Biochem J* 434: 513-521
Sugio S, Nagasawa M, Kojima I, Ishizaki Y, Shibasaki K (2017) Transient receptor potential vanilloid 2 activation by focal mechanical stimulation requires interaction with the actin cytoskeleton and enhances growth cone motility. *FASEB J* 31: 1368-1381
te Boekhorst V, Jiang L, Mählen M, Meerlo M, Dunkel G, Durst FC, Yang Y, Levine H, Burgering BMT, Friedl P (2020) Calpain-2 regulates hypoxia/HIF-induced amoeboid reprogramming and metastasis. *bioRxiv*: 2020.2001.2006.892497
Thomson JA, Murphy K, Baker E, Sutherland GR, Parsons PG, Sturm RA, Thomson F (1995) The brn-2 gene regulates the melanocytic phenotype and tumorigenic potential of human melanoma cells. *Oncogene* 11: 691-700

Tichet M, Prod'Homme V, Fenouille N, Ambrosetti D, Mallavialle A, Cerezo M, Ohanna M, Audebert S, Rocchi S, Giacchero D *et al* (2015) Tumour-derived SPARC drives vascular permeability and extravasation through endothelial VCAM1 signalling to promote metastasis. *Nat Commun* 6: 6993

Tirosh I, Izar B, Prakadan SM, Wadsworth MH, 2nd, Treacy D, Trombetta JJ, Rotem A, Rodman C, Lian C, Murphy G *et al* (2016) Dissecting the multicellular ecosystem of metastatic melanoma by single-cell RNA-seq. *Science* 352: 189-196

Tuncer E, Calcada RR, Zingg D, Varum S, Cheng P, Freiberger SN, Deng CX, Kleiter I, Levesque MP, Dummer R *et al* (2019) SMAD signaling promotes melanoma metastasis independently of phenotype switching. *J Clin Invest* 129: 2702-2716

Verfaillie A, Imrichova H, Atak ZK, Dewaele M, Rambow F, Hulselmans G, Christiaens V, Svetlichnyy D, Luciani F, Van den Mooter L *et al* (2015) Decoding the regulatory landscape of melanoma reveals TEADS as regulators of the invasive cell state. *Nat Commun* 6: 6683

Vriens J, Appendino G, Nilius B (2009) Pharmacology of vanilloid transient receptor potential cation channels. *Mol Pharmacol* 75: 1262-1279

Wellbrock C, Rana S, Paterson H, Pickersgill H, Brummelkamp T, Marais R (2008) Oncogenic BRAF regulates melanoma proliferation through the lineage specific factor MITF. *PLoS One* 3: e2734

White JR, Thompson DT, Koch KE, Kiriazov BS, Beck AC, van der Heide DM, Grimm BG, Kulak MV, Weigel RJ (2021) AP-2alpha-Mediated Activation of E2F and EZH2 Drives Melanoma Metastasis. *Cancer Res* 81: 4455-4470

Wouters J, Kalender-Atak Z, Minnoye L, Spanier KI, De Waegeneer M, Bravo Gonzalez-Blas C, Mauduit D, Davie K, Hulselmans G, Najem A *et al* (2020) Robust gene expression programs underlie recurrent cell states and phenotype switching in melanoma. *Nat Cell Biol* 22: 986-998

Yang S, Zhang JJ, Huang XY (2009) Orai1 and STIM1 are critical for breast tumor cell migration and metastasis. *Cancer Cell* 15: 124-134

Yao M, Tijore A, Cox CD, Hariharan A, Van Nhieu GT, Martinac B, Sheetz M (2020) Force-dependent Piezo1 recruitment to focal adhesions regulates adhesion maturation and turnover specifically in non-transformed cells. *bioRxiv*: 2020.2003.2009.972307

Yee NS, Kazi AA, Li Q, Yang Z, Berg A, Yee RK (2015) Aberrant over-expression of TRPM7 ion channels in pancreatic cancer: required for cancer cell invasion and implicated in tumor growth and metastasis. *Biol Open* 4: 507-514

Yu Y, Wu X, Liu S, Zhao H, Li B, Zhao H, Feng X (2021a) Piezo1 regulates migration and invasion of breast cancer cells via modulating cell mechanobiological properties. *Acta Biochim Biophys Sin (Shanghai)* 53: 10-18

Yu ZH, Ji YC, Li K, Liang T, Liu B, Chen HL, Ni L, Luo ZP, Yang HL (2021b) Stiffness of the extracellular matrix affects apoptosis of nucleus pulposus cells by regulating the cytoskeleton and activating the TRPV2 channel protein. *Cell Signal* 84: 110005

Zaidel-Bar R, Ballestrem C, Kam Z, Geiger B (2003) Early molecular events in the assembly of matrix adhesions at the leading edge of migrating cells. *J Cell Sci* 116: 4605-4613

Zhang J, Zhou Y, Huang T, Wu F, Liu L, Kwan JSH, Cheng ASL, Yu J, To KF, Kang W (2018) PIEZO1 functions as a potential oncogene by promoting cell proliferation and migration in gastric carcinogenesis. *Mol Carcinog* 57: 1144-1155

zhang s, cao s, mengyuan g, wunai z, weifan z, zeen z, shuai w, yue s, lin w, yangyang y *et al* (2022) The Piezo1 Mechanosensitive Ion Channel Promotes Melanoma Malignant Progression by Activating PI3K-AKT Signaling. *PREPRINT (Version 1) available at Research Square*

Dear Dr. PENNA

Thank you for the submission of your revised manuscript to EMBO reports. We have now received the full set of referee reports that is copied below.

As you will see, the referees acknowledge that the study has been strengthened during the revision and referee 1 and 2 support publication with minor textual revisions. Referee 3 raises concerns regarding the conclusiveness of your in vivo data that TRPV2 promotes melanoma invasiveness. Upon further discussion with all three referees, we have decided that these concerns can be addressed in the text. Please discuss the limitations of your data and explain discrepancies. Please justify also why you chose the less widely-used WM266.4 and 451Lu cells to show that TRPV2 is essential for melanoma cell migration and invasion and not SK-Mel-2 and SK-Mel-28 , which show high expression of TRPV2 in Fig. 1F.

From the editorial side, there are also a few things that we need before we can proceed with the official acceptance of your study.

1) Regarding the Author Contributions, we now use CRediT to specify the contributions of each author in the journal submission system. CRediT replaces the author contribution section, which therefore needs to be removed from the manuscript text. You can use the free text box in our system if you wish to provide more detailed descriptions. See also guide to authors https://www.embopress.org/page/journal/14693178/authorguide#authorshipguidelines.

2) Please add callouts in the text to the following figure panels:
Fig 5D, EV1A&B, EV4A&B, Appendix Fig S5A&B, S8A-C, S9A&B, S10A&B.

3) Fig EV2C&D are called out before EV2A&B. We generally recommend arranging the figure panels so that they can be called out alphabetically. If possible, please rearrange.

4) Reagent and Tools table: Please remove the lines form the .xls table that describe how to fill the sheets (i.e., lines 2, 14, 23, 39, 43 etc). There is a typo in line 40 (appendix should be Appendix)

5) Appendix:
- Please add page numbers to the table of content and please use 'related to Figure X' instead of 'relative to'
- Please remove the headings from the figures (Supplemental Figure X)

- Appendix Fig. S1 (A): please define the red arrow. S1A-C: define the boxes, whiskers and central band of the box plot
- S2B: please provide a scale bar and define its size in the legend
- S2C: define the number of samples (n) and whether these are independent (biological) or technical replicates. Please define the error bars
- S3A: please define whether n = 5 refers to biological or technical replicates. Please define the statistical test used and the exact value of ****
- S3B: define the size of the scale bar
- S3C: define the error bars and n (biological, technical)
- S5A: please add scale bars to the small and large images and define their size in the figure legend. Please separate the individual images by adding lines. The term 'insets' seems imprecise as it could also refer to the small images to the left. Maybe use 'panels below'.
- S6A: please add a scale bar and define its size in the figure legend.
- S6C-E: please define the black and grey arrows on the TALIN IB
- S7: please define the boxes, whiskers and central bands of the box blot, the statistical test used and the exact value of **
- S9A: You use CM-DiL in the figure and CmDil in the legend.
- S9B: Please define the statistical test used the mean/average and the error bars.
- S10B: please provide scale bars for both samples and define their size in the legend.
- S11A+B: please define the boxes, whiskers and central band of the box plots and the exact value of *. Please define the abbreviation n(T) and n(N). Please change the legend for C) to (B)
- S11C: we need a scale bar and its size defined in the legend.

6) Figure EV2C displays data from 2 technical replicates as mean plus/minus SEM. Since the data are based on only 2 experiments, please display the individual datapoints instead of the mean and please remove the statistical analysis. Please note that we generally recommend showing individual datapoints in addition to the mean if the data are based n < 5.

7) I attach to this email a related manuscript file with comments by our data editors. Please address all comments and upload a revised file with tracked changes with your final manuscript submission.
- I have also moved your disclosure statement to a separate paragraph called 'Disclosure and competing interests statement' in the text (after the Author Contributions) and added some suggestions to the abstract.

8) Synopsis image: The text in the inset image is rather difficult to read and I suggest simplyfing the inset so that the individual components and the text are easier to interpret.

We look forward to seeing a final version of your manuscript as soon as possible.

Yours sincerely,

Martina Rembold, PhD
Senior Editor
EMBO reports

****

Referee #1:

The authors have addressed all my previous comments. On page 12 they claim that "TRPV2 intensity appeared to correlate with advanced lesions..." but have no statistical significance to back this up. This sentence needs to be removed or altered to reflect that fact that there is no statistical significance. No claim otherwise should be made.

On page 4: "..was to identify any atypical profile..." should be "was to identify atypial profiles....

On page 5: "...TRPV2 expression was exceeding the other non-voltage...." should be "TRPV2 expression exceeded the expression of the other non-voltage...."

The word "levels" is overused when talking about expression.

The sentence "The preferential distribution of TPRV2 within melanoma..." needs to be revisited.

The sentence "We therefore investigate TRPV2 expression versus melanoma invasiveness..." should be: "We therefore investigated TRPV2 expression with respect to melanoma invasiveness, by quantifying TRPV2 transcripts along with..."

Page 12: What is a "local hospital independent tumor biobank"? Why is this institute anonymous? Are there ethical approvals available?

Referee #2:

The authors have satisfactorily addressed all the points raised on the previous version of their manuscript. In particular, the data pertaining to the potential regulation of the TRPV2 locus by TFAP2A constitutes a valuable addition to this paper. The new results formally implicating calpain activity in the regulation of melanoma cell migration downstream of TRPV2 also consolidate the proposed mechanism. Overall, the revisions have strengthened this manuscript, which is suitable for publication in EMBO reports.

Referee #3:

According to the Human Protein Atlas, TRPV2 seems to be enriched in melanoma, suggesting a role of TRPV2 in melanoma metastasis. The authors performed protein profile analyses among various cancer cell lines and concluded that TRPV2 is a biomarker for melanoma metastasis. Additional experiments were performed to investigate the downstream molecular mechanisms. In their revised manuscript, the addition of discussions and new experimental results helped elaborate their conclusions. However, the overall experimental evidence to support their claim that "TRPV2 controls human melanoma invasiveness..." was not strong. The study remains superficial in terms of the underlying molecular mechanisms: TRPV2--Ca2+--calpain/talin--melanoma invasiveness. Several internal inconsistencies require explanations. For example, for the data set of tumor-bearing mice in Fig. 6A/B, no significant difference (ns.) was seen in vivo Day 64, yet significant difference (*) was seen in the Ex vivo necropsy data. On the contrary, significant difference (**) was seen for In vivo Day 35 in in Fig. 6C, while no significant difference (ns.) was seen for the Ex vivo necropsy data in Fig. 6D. In Fig.5C, in the cleavage of talin experiment, it appeared that no decrease of the upper band, which I assumed to be the full-length tarlin protein, was observed.

Reviewers' revision reports and point-by-point responses:

To facilitate the reading of our point-by-point responses, the points raised by the referees are in black italic and our answers are in blue.
To help track changes in the revised manuscript, amendments made in response to the first round of revision and during reformatting according to EMBO Report guidelines were highlighted in green. The amendments made in response to the new concerns raised by the referees are in red.

Referee #1:

The authors have addressed all my previous comments.

On page 12 they claim that "TRPV2 intensity appeared to correlate with advanced lesions..." but have no statistical significance to back this up. This sentence needs to be removed or altered to reflect that fact that there is no statistical significance. No claim otherwise should be made.

The sentence (**page 12**) has been changed to clearly indicate that there is no statistical significance to support this claim:
"A closer look at the results suggested a tendency towards a positive correlation between the progression of the disease and TRPV2 expression. However, the statistical analysis did not support these observations, likely due to interindividual variability as well as a limited number of high-grade lesions (4 grade III and 2 grade IV) (Fig 7C-D and appendix Fig S11B)."

On page 4: "..was to identify any atypical profile..." should be "was to identify atypial profiles.... *Changed*

On page 5: "...TRPV2 expression was exceeding the other non-voltage...." should be "TRPV2 expression exceeded the expression of the other non-voltage...." *Changed*

The word "levels" is overused when talking about expression.
We have revised our manuscript to avoid overusing the word "level".

The sentence "The preferential distribution of TPRV2 within melanoma..." needs to be revisited.

This sentence was changed (**page 5**) for:
"Consistently, among a large panel of cancer cell lines originating from different tissues, TRPV2 channel proteins were preferentially detected in melanoma cells (Fig 1F)."

The sentence "We therefore investigate TRPV2 expression versus melanoma invasiveness..." should be: "We therefore investigated TRPV2 expression with respect to melanoma invasiveness, by quantifying TRPV2 transcripts along with..." *Changed*

Page 12: What is a "local hospital independent tumor biobank"? Why is this institute anonymous? Are there ethical approvals available?

We apologize for the lack of clarity about the origin of these patient samples. Our initial goal was to specify that they were different from the TMA, but the phrasing was very clumsy. As now indicated in the manuscript (**page 12**) and in the legend of the **appendix Fig S11**, these melanoma samples were obtained from the Rennes University Hospital (CHU) tumor biobank (Centre de ressources biologiques humaines - CRB Santé **https://www.chu-rennes.fr/crb-sante.html).** This biological resources center manages and promotes human biological resources and associated bioclinical data (all obtained with patient approval), for research purposes.

aubin.penna@univ-poitiers.fr | +33 (0)5 49 45 39 96

Referee #2:

The authors have satisfactorily addressed all the points raised on the previous version of their manuscript. In particular, the data pertaining to the potential regulation of the TRPV2 locus by TFAP2A constitutes a valuable addition to this paper. The new results formally implicating calpain activity in the regulation of melanoma cell migration downstream of TRPV2 also consolidate the proposed mechanism. Overall, the revisions have strengthened this manuscript, which is suitable for publication in EMBO reports.

We are grateful for these positive and supportive comments, and we are thankful for acknowledging the added value brought by our thorough responses to the referees.

Referee #3:

According to the Human Protein Atlas, TRPV2 seems to be enriched in melanoma, suggesting a role of TRPV2 in melanoma metastasis. The authors performed protein profile analyses among various cancer cell lines and concluded that TRPV2 is a biomarker for melanoma metastasis. Additional experiments were performed to investigate the downstream molecular mechanisms. In their revised manuscript, the addition of discussions and new experimental results helped elaborate their conclusions. However, the overall experimental evidence to support their claim that "TRPV2 controls human melanoma invasiveness..." was not strong. The study remains superficial in terms of the underlying molecular mechanisms: TRPV2--Ca2+--calpain/talin--melanoma invasiveness. Several internal inconsistencies require explanations.

We are truly sorry to read that our study could be seen as "*not strong*" or "*superficial*" in terms of mechanisms. To support the statement that TRPV2 stands out as a valuable biomarker for malignant melanoma tumors, we not only showed that TRPV2 is enriched, at both the mRNA and the protein levels, in melanoma cell lines as well as in human metastatic melanoma samples, and most importantly that TRPV2 expression directly correlates to the aggressiveness of the tumor and to patient mortality in human melanoma biopsies. Moreover, the pivotal role of TRPV2 in the control of human melanoma invasiveness was substantiated by the fact that 1- *in vitro* (in 3 different cell lines) TRPV2 expression confers both migratory and invasive potentials, while its silencing prevents these malignant behaviors; and 2- *in vivo* (in 2 murine and 1 zebrafish models) TRPV2 expression directly correlates with melanoma cells metastatic potential. Regarding the molecular mechanisms underlying TRPV2 controls over melanoma cells invasiveness, we showed that TRPV2 is present in nascent adhesion structures and regulates the proteolytic activity of the Ca^{2+}-sensitive calpain. We further established a causal link between TRPV2, calpain/talin and melanoma cells migration. Although elements of this molecular pathway are still missing, the description of this malignant-specific molecular mechanism driven by TRPV2 is bringing new explanation on how the regulation of adhesion dynamics *via* the calpain system can be connected to the sensing of the tumoral microenvironment. We also would like to point out the fact that EMBO reports "*focuses on novelty and the physiological and/or functional significance of a finding, rather than the level of mechanistic detail reported*".
We hope that the above arguments will be convincing of the strength of our study, and that the following explanations will alleviate any inconsistencies.

"For example, for the data set of tumor-bearing mice in Fig. 6A/B, no significant difference (ns.) was seen in vivo Day 64, yet significant difference (*) was seen in the Ex vivo necropsy data. On the contrary, significant difference (**) was seen for In vivo Day 35 in in Fig. 6C, while no significant difference (ns.) was seen for the Ex vivo necropsy data in Fig. 6D."

Regarding the 501mel model overexpressing TRPV2 (see Fig 6A/B and EV4A/B): all our *in vivo* BLI measurements were done by using a rectangular ROI encompassing the thorax. The *ex vivo* analysis revealed that, in this model, the metastases were mainly found at the periphery of the animals, notably the bones in the limbs and the brain, with sometimes very small metastatic foci albeit abundant. We believe that the distribution coupled to the size/intensity of the metastases could be accountable for

aubin.penna@univ-poitiers.fr | +33 (0)5 49 45 39 96

the lack of detection of the luminescence in the entire animals. It is also noteworthy that the 501mel cell line is a poorly metastatic model in mice due to intrinsic cellular characteristics.

Conversely, in the highly metastatic 451Lu model (see Fig 6C/D and appendix Fig S8), most of the metastases developed in the control animals were found in the lungs and were very big and bright. In fact, to minimize saturation, the luminescence intensity at measurement had to be adjusted, which in turn led to a loss of detection of the small and dim foci. Ultimately, the number of foci quantified *ex vivo* were largely underestimated also due to the fact that individual metastases were hardly distinguishable. This was supported by the IHC staining of the metastases where higher numbers of foci of variable size could be detected (Appendix Fig S8). Finally, as shown in Fig 6C/D, the *in vivo* BLI signal as well as the ex vivo luminescence of the lungs were drastically less intense in the TRPV2-silenced metastases than in the control mice.

Although we believe that our *in vivo* murine experimental approach supports the conclusion that TRPV2 promotes human melanoma cells metastatic potential, it is likely that increasing the number of animals and refining the quantitative methods to be able to distinguish between individual metastatic foci would have enabled statistical significance to be reached in every conditions. Moreover, and as largely discussed in our first response to referee 3, it is important to keep in mind that the metastatic tropism of the 501mel and the 451Lu cells are very distinct (with the 451Lu preferentially metastasizing into the lungs). Finally, we would like to stress out once again the fact that we also undertook an *in vivo* approach in Zebrafish that showed similar results. Please refer to the manuscript **pages 11 and 12** for amendments.

"In Fig.5C, in the cleavage of talin experiment, it appeared that no decrease of the upper band, which I assumed to be the full-length tarlin protein, was observed."

It is true that in our study, as well as in many others using either the same 8d4 talin antibody (*Sigma-Aldrich*) as us (for example Fig 2 in (Nuzzi *et al*, 2007), Fig 2E in (Chantome *et al*, 2013), Fig 5A in (Bumba *et al*, 2010)), or using the TA205 clone anti-talin monoclonal antibody (Fig 5 & 6 in (Fong *et al*, 2021)), the increase of the talin cleaved form was not always correspondingly associated with a decrease of the full-length protein. As of now, we do not have any explanation for the steady detection of the full-length form, and whether it is a technical issue (resulting from the semi-quantitative western blotting method itself) or an unknown biological reason. However, we do believe the strong detection of the cleaved form only in TRPV2 expressing cells clearly supports our conclusion that in metastatic melanoma cells, TRPV2 regulates calpain activity and the resulting cleavage of talin.

aubin.penna@univ-poitiers.fr | +33 (0)5 49 45 39 96

REFERENCES

Arozarena I, Bischof H, Gilby D, Belloni B, Dummer R, Wellbrock C (2011a) In melanoma, beta-catenin is a suppressor of invasion. *Oncogene* 30: 4531-4543

Arozarena I, Sanchez-Laorden B, Packer L, Hidalgo-Carcedo C, Hayward R, Viros A, Sahai E, Marais R (2011b) Oncogenic BRAF induces melanoma cell invasion by downregulating the cGMP-specific phosphodiesterase PDE5A. *Cancer Cell* 19: 45-57

Bumba L, Masin J, Fiser R, Sebo P (2010) Bordetella adenylate cyclase toxin mobilizes its beta2 integrin receptor into lipid rafts to accomplish translocation across target cell membrane in two steps. *PLoS Pathog* 6: e1000901

Chantome A, Potier-Cartereau M, Clarysse L, Fromont G, Marionneau-Lambot S, Gueguinou M, Pages JC, Collin C, Oullier T, Girault A *et al* (2013) Pivotal role of the lipid Raft SK3-Orai1 complex in human cancer cell migration and bone metastases. *Cancer Res* 73: 4852-4861

Chapman A, Fernandez del Ama L, Ferguson J, Kamarashev J, Wellbrock C, Hurlstone A (2014) Heterogeneous tumor subpopulations cooperate to drive invasion. *Cell Rep* 8: 688-695

Fong KP, Molnar KS, Agard N, Litvinov RI, Kim OV, Wells JA, Weisel JW, DeGrado WF, Bennett JS (2021) Cleavage of talin by calpain promotes platelet-mediated fibrin clot contraction. *Blood Adv* 5: 4901-4909

Herlyn D, Iliopoulos D, Jensen PJ, Parmiter A, Baird J, Hotta H, Adachi K, Ross AH, Jambrosic J, Koprowski H *et al* (1990) In vitro properties of human melanoma cells metastatic in nude mice. *Cancer Res* 50: 2296-2302

Nuzzi PA, Senetar MA, Huttenlocher A (2007) Asymmetric localization of calpain 2 during neutrophil chemotaxis. *Mol Biol Cell* 18: 795-805

Rowling EJ, Miskolczi Z, Nagaraju R, Wilcock DJ, Wang P, Telfer B, Li Y, Lasheras-Otero I, Redondo-Munoz M, Sharrocks AD *et al* (2020) Cooperative behaviour and phenotype plasticity evolve during melanoma progression. *Pigment Cell Melanoma Res* 33: 695-708

aubin.penna@univ-poitiers.fr | *+33 (0)5 49 45 39 96*

2nd Revision - Editorial Decision **10th Jan 2023**

Dr. Aubin PENNA
Univ Poitiers, CNRS, 4CS
France

Dear Dr. Penna,

Thank you for sending the further modified manuscript file, which I have uploaded for you. I am now very pleased to accept your manuscript for publication in the next available issue of EMBO reports. Thank you for your contribution to our journal.

At the end of this email I include important information about how to proceed. Please ensure that you take the time to read the information and complete and return the necessary forms to allow us to publish your manuscript as quickly as possible.

As part of the EMBO publication's Transparent Editorial Process, EMBO reports publishes online a Review Process File to accompany accepted manuscripts. As you are aware, this File will be published in conjunction with your paper and will include the referee reports, your point-by-point response and all pertinent correspondence relating to the manuscript.

If you do NOT want this File to be published, please inform the editorial office within 2 days, if you have not done so already, otherwise the File will be published by default [contact: emboreports@embo.org]. If you do opt out, the Review Process File link will point to the following statement: "No Review Process File is available with this article, as the authors have chosen not to make the review process public in this case." Please note that the author checklist will still be published even if you opt out of the transparent process.

Thank you again for your contribution to EMBO reports and congratulations on a successful publication. Please consider us again in the future for your most exciting work.

Kind regards,

Martina Rembold

Martina Rembold, PhD
Senior Editor
EMBO reports

****

THINGS TO DO NOW:

Once your article has been received by Wiley for production, the corresponding author will receive an email from Wiley's Author Services system which will ask them to log in and will present them with the appropriate license for completion.

You will receive proofs by e-mail approximately 2-3 weeks after all relevant files have been sent to our Production Office; you should return your corrections within 2 days of receiving the proofs.

Please inform us if there is likely to be any difficulty in reaching you at the above address at that time. Failure to meet our deadlines may result in a delay of publication, or publication without your corrections.

All further communications concerning your paper should quote reference number EMBOR-2022-55069V3 and be addressed to emboreports@wiley.com.

Should you be planning a Press Release on your article, please get in contact with emboreports@wiley.com as early as possible, in order to coordinate publication and release dates.

EMBO Press Author Checklist

Corresponding Author Name: Aubin PENNA	
Journal Submitted to: EMBO Reports	
Manuscript Number: EMBOR-2022-55069V1	

USEFUL LINKS FOR COMPLETING THIS FORM
The EMBO Journal - Author Guidelines
EMBO Reports - Author Guidelines
Molecular Systems Biology - Author Guidelines
EMBO Molecular Medicine - Author Guidelines

Reporting Checklist for Life Science Articles (updated January 2022)
This checklist is adapted from Materials Design Analysis Reporting (MDAR) Checklist for Authors. MDAR establishes a minimum set of requirements in transparent reporting in the life sciences (see Statement of Task: 10.31222/osf.io/9sm4x). Please follow the journal's guidelines in preparing your manuscript.
Please note that a copy of this checklist will be published alongside your article.

Abridged guidelines for figures
1. Data
The data shown in figures should satisfy the following conditions:
- the data were obtained and processed according to the field's best practice and are presented to reflect the results of the experiments in an accurate and unbiased manner.
- ideally, figure panels should include only measurements that are directly comparable to each other and obtained with the same assay.
- plots include clearly labeled error bars for independent experiments and sample sizes. Unless justified, error bars should not be shown for technical replicates.
- if n<5, the individual data points from each experiment should be plotted. Any statistical test employed should be justified.
- Source Data should be included to report the data underlying figures according to the guidelines set out in the authorship guidelines on Data Presentation.

2. Captions
Each figure caption should contain the following information, for each panel where they are relevant:
- a specification of the experimental system investigated (eg cell line, species name).
- the assay(s) and method(s) used to carry out the reported observations and measurements.
- an explicit mention of the biological and chemical entity(ies) that are being measured.
- an explicit mention of the biological and chemical entity(ies) that are altered/varied/perturbed in a controlled manner.
- the exact sample size (n) for each experimental group/condition, given as a number, not a range;
- a description of the sample collection allowing the reader to understand whether the samples represent technical or biological replicates (including how many animals, litters, cultures, etc.).
- a statement of how many times the experiment shown was independently replicated in the laboratory.
- definitions of statistical methods and measures:
  - common tests, such as t-test (please specify whether paired vs. unpaired), simple χ2 tests, Wilcoxon and Mann-Whitney tests, can be unambiguously identified by name only, but more complex techniques should be described in the methods section;
  - are tests one-sided or two-sided?
  - are there adjustments for multiple comparisons?
  - exact statistical test results, e.g., P values = x but not P values < x;
  - definition of 'center values' as median or average;
  - definition of error bars as s.d. or s.e.m.

Please complete ALL of the questions below.
Select "Not Applicable" only when the requested information is not relevant for your study.

Materials

Newly Created Materials	Information included in the manuscript?	In which section is the information available? (Reagents and Tools Table, Materials and Methods, Figures, Data Availability Section)
New materials and reagents need to be available; do any restrictions apply?	Yes	Data Availability section

Antibodies	Information included in the manuscript?	In which section is the information available? (Reagents and Tools Table, Materials and Methods, Figures, Data Availability Section)
For **antibodies** provide the following information:<br>- Commercial antibodies: RRID (if possible) or supplier name, catalogue number and or/clone number<br>- Non-commercial: RRID or citation	Yes	Material and Methods and Reagents and Tools Table

DNA and RNA sequences	Information included in the manuscript?	In which section is the information available? (Reagents and Tools Table, Materials and Methods, Figures, Data Availability Section)
**Short novel DNA or RNA including primers, probes**: provide the sequences.	Yes	appendix Table S1

Cell materials	Information included in the manuscript?	In which section is the information available? (Reagents and Tools Table, Materials and Methods, Figures, Data Availability Section)
**Cell lines:** Provide species information, strain. Provide accession number in repository **OR** supplier name, catalog number, clone number, and/**OR** RRID.	Yes	Material and Methods and Reagents and Tools Table
**Primary cultures:** Provide species, strain, sex of origin, genetic modification status.	Not Applicable	
Report if the cell lines were recently **authenticated** (e.g., by STR profiling) and tested for mycoplasma contamination.	Yes	Material and Methods

Experimental animals	Information included in the manuscript?	In which section is the information available? (Reagents and Tools Table, Materials and Methods, Figures, Data Availability Section)
**Laboratory animals or Model organisms:** Provide species, strain, sex, age, genetic modification status. Provide accession number in repository **OR** supplier name, catalog number, clone number, **OR** RRID.	Yes	Material and Methods and Reagents and Tools Table
**Animal observed in or captured from the field:** Provide species, sex, and age where possible.	Not Applicable	
Please detail **housing and husbandry conditions**.	Yes	Material and Methods: Mouse models and In vivo experiments.

Plants and microbes	Information included in the manuscript?	In which section is the information available? (Reagents and Tools Table, Materials and Methods, Figures, Data Availability Section)
**Plants:** provide species and strain, ecotype and cultivar where relevant, unique accession number if available, and source (including location for collected wild specimens).	Not Applicable	
**Microbes:** provide species and strain, unique accession number if available, and source.	Not Applicable	

Human research participants	Information included in the manuscript?	In which section is the information available? (Reagents and Tools Table, Materials and Methods, Figures, Data Availability Section)
If collected and within the bounds of privacy constraints report on age, sex and gender or ethnicity for all study participants.	Not Applicable	

Core facilities	Information included in the manuscript?	In which section is the information available? (Reagents and Tools Table, Materials and Methods, Figures, Data Availability Section)
If your work benefited from core facilities, was their service mentioned in the acknowledgments section?	Yes	Acknowledgments section

Design

Study protocol	Information included in the manuscript?	In which section is the information available? (Reagents and Tools Table, Materials and Methods, Figures, Data Availability Section)
If study protocol has been **pre-registered, provide DOI in the manuscript**. For clinical trials, provide the trial registration number **OR** cite DOI.	Not Applicable	
Report the **clinical trial registration number** (at ClinicalTrials.gov or equivalent), where applicable.	Not Applicable	

Laboratory protocol	Information included in the manuscript?	In which section is the information available? (Reagents and Tools Table, Materials and Methods, Figures, Data Availability Section)
Provide DOI OR other citation details if **external detailed step-by-step protocols** are available.	Yes	Material and Methods

Experimental study design and statistics	Information included in the manuscript?	In which section is the information available? (Reagents and Tools Table, Materials and Methods, Figures, Data Availability Section)
Include a statement about **sample size** estimate even if no statistical methods were used.	Yes	Figure and Figure legends
Were any steps taken to minimize the effects of subjective bias when allocating animals/samples to treatment (e.g. **randomization procedure**)? If yes, have they been described?	Not Applicable	
Include a statement about **blinding** even if no blinding was done.	Not Applicable	
Describe **inclusion/exclusion criteria** if samples or animals were excluded from the analysis. Were the criteria pre-established?<br><br>If sample or data points were omitted from analysis, report if this was due to attrition or intentional exclusion and provide justification.	Not Applicable	
For every figure, are **statistical tests** justified as appropriate? Do the data meet the assumptions of the tests (e.g., normal distribution)? Describe any methods used to assess it. Is there an estimate of variation within each group of data? Is the variance similar between the groups that are being statistically compared?	Yes	Figure legends

Sample definition and in-laboratory replication	Information included in the manuscript?	In which section is the information available? (Reagents and Tools Table, Materials and Methods, Figures, Data Availability Section)
In the figure legends: state number of times the experiment was **replicated** in laboratory.	Yes	Figure legends
In the figure legends: define whether data describe **technical or biological replicates**.	Yes	Figure legends

Ethics

Ethics	Information included in the manuscript?	In which section is the information available? (Reagents and Tools Table, Materials and Methods, Figures, Data Availability Section)
Studies involving **human participants**: State details of **authority granting ethics approval** (IRB or equivalent committee(s), provide reference number for approval.	Not Applicable	
Studies involving **human participants**: Include a statement confirming that **informed consent** was obtained from all subjects and that the experiments conformed to the principles set out in the WMA Declaration of Helsinki and the Department of Health and Human Services Belmont Report.	Not Applicable	
Studies involving **human participants:** For publication of **patient photos**, include a statement confirming that consent to publish was obtained.	Not Applicable	
Studies involving experimental **animals**: State details of **authority granting ethics approval** (IRB or equivalent committee(s), provide reference number for approval. Include a statement of compliance with ethical regulations.	Yes	Material and Methods
Studies involving **specimen and field samples:** State if relevant **permits** obtained, provide details of authority approving study; if none were required, explain why.	Not Applicable	

Dual Use Research of Concern (DURC)	Information included in the manuscript?	In which section is the information available? (Reagents and Tools Table, Materials and Methods, Figures, Data Availability Section)
Could your study fall under dual use research restrictions? Please check biosecurity documents and list of **select agents and toxins** (CDC): https://www.selectagents.gov/sat/list.htm	Not Applicable	
If you used a select agent, is the security level of the lab appropriate and reported in the manuscript?	Not Applicable	
If a study is subject to dual use research of concern regulations, is the name of the **authority granting approval and reference number** for the regulatory approval provided in the manuscript?	Not Applicable	

Reporting

The MDAR framework recommends adoption of discipline-specific guidelines, established and endorsed through community initiatives. Journals have their own policy about requiring specific guidelines and recommendations to complement MDAR.

Adherence to community standards	Information included in the manuscript?	In which section is the information available? (Reagents and Tools Table, Materials and Methods, Figures, Data Availability Section)
State if relevant guidelines or checklists (e.g., **ICMJE, MIBBI, ARRIVE, PRISMA**) have been followed or provided.	Not Applicable	
For **tumor marker prognostic studies**, we recommend that you follow the **REMARK** reporting guidelines (see link list at top right). See author guidelines, under 'Reporting Guidelines'. Please confirm you have followed these guidelines.	Not Applicable	
For **phase II and III randomized controlled trials**, please refer to the **CONSORT** flow diagram (see link list at top right) and submit the CONSORT checklist (see link list at top right) with your submission. See author guidelines, under 'Reporting Guidelines'. Please confirm you have submitted this list.	Not Applicable	

Data Availability

Data availability	Information included in the manuscript?	In which section is the information available? (Reagents and Tools Table, Materials and Methods, Figures, Data Availability Section)
Have **primary datasets** been deposited according to the journal's guidelines (see 'Data Deposition' section) and the respective accession numbers provided in the Data Availability Section?	Not Applicable	
Were **human clinical and genomic datasets** deposited in a public access-controlled repository in accordance to ethical obligations to the patients and to the applicable consent agreement?	Not Applicable	
Are **computational models** that are central and integral to a study available without restrictions in a machine-readable form? Were the relevant accession numbers or links provided?	Not Applicable	
If publicly available data were reused, provide the respective **data citations in the reference list**.	Yes	Materials and Methods, Figure legends
